# G10 is a direct activator of human STING

**DOI:** 10.1371/journal.pone.0237743

**Published:** 2020-09-10

**Authors:** Monali Banerjee, Sandip Middya, Ritesh Shrivastava, Sourav Basu, Rajib Ghosh, David C. Pryde, Dharmendra B. Yadav, Arjun Surya

**Affiliations:** 1 Curadev Pharma Pvt. Ltd., Noida, Uttar Pradesh, India; 2 Curadev Pharma Ltd., Sandwich, Kent, United Kingdom; University of St Andrews, UNITED KINGDOM

## Abstract

The cGAS/STING pathway initiates an innate immune response when DNA is detected in the cytosol. DNA bound cGAS synthesizes cyclic dinucleotides which bind and activate the adaptor STING, leading to downstream secretion of Type I interferons and other pro-inflammatory NFκB pathway cytokines. In the mouse, the STING driven innate immune response is central to immune based clearance of various tumors and this has triggered a significant effort focused on the discovery of human STING agonists for the treatment of cancer. This report uses an in vitro kinase assay to show that G10, a previously identified STING pathway activator is actually a weak but direct STING agonist and identifies other more potent leads.

## Introduction

STING (STimulator of INterferon Genes) is a transmembrane adaptor protein expressed on mitochondrial and endoplasmic reticulum membranes that serves as a transducer of innate immune signalling leading to the secretion of cytokines and the maturation and activation of proximal antigen presenting cells (APCs) [1,2]. The innate immune system senses and responds to danger from external pathogens or internal neoplasms through an array of pattern recognition proteins that are present in most tissues and stimulate the secretion of inflammatory cytokines [3,4]. One such danger signal is cytosolic DNA which is sensed by the enzyme cyclic GMP AMP synthase (cGAS) which responds by synthesizing the cyclic dinucleotide (CDN) cyclic guanine adenine monophosphate (cGAMP), a potent agonist of STING [5,6]. Agonist binding causes a large conformational change in STING that eventually leads to gene transcription and secretion of Type I interferons and pro-inflammatory cytokines of the NFκB lineage. An early step in this cascade is the activation of Tank Binding Kinase 1 (TBK1) by cGAMP bound STING [7,8]. TBK1 phosphorylates the transcription factor IRF3 and also activates NFκB to initiate the STING driven pro-inflammatory response [9].

The maturation of various antigen presenting cells (APCs) is driven by STING activation as part of a critical sequence of events that leads to an amplified immune response where APCs activate cytotoxic T cells from adjacent lymph nodes. In pre-clinical models, activation of APCs by STING has led to tumor regression of non-inflamed or “cold” tumors that lack a T cell infiltrate [10,11]. The innate immune response is muted in several cancers [12,13] and so the demonstration that the activation of STING by CDNs or the small molecule DMXAA leads to significant tumor regression in murine models has led to a flurry of activity directed towards the discovery of STING agonists [14]. STING is polymorphic in humans with five variants [15]–R232 (58% of population), HAQ (20%), H232 (13%), AQ (7%) and Q (2%) that account for greater than 99% of the STING alleles found in humans. STING agonists described in the literature are either CDN-based that typically activate STING from most species [16] or non-CDN small molecules that tend to be species specific with some exceptions. [17]. Currently, the most clinically advanced chemotypes are the CDNs ADU-S100 [18] (Aduro Biotech/Novartis) and MK-1474 [19] (Merck). Both are close analogs of cGAMP and are administered to patients by the intra-tumoral (IT) route. The clinical application of CDNs may be limited by their polar, macrocyclic, multi-chiral structure which affects their pharmacokinetic properties and creates hurdles for large scale synthesis. For these and allied reasons, there is an active effort to identify classical small molecules that activate STING. Unfortunately, DMXAA does not activate any variant of human STING [20].

The quest for small molecule STING agonists has certain challenges. STING, which has only recently been described, is a transmembrane adaptor protein located on mitochondrial, endoplasmic reticulum and plasma membranes [21]. Wide clinical application with uniform doses will require the agonist to activate multiple human protein variants with similar potency. As an innate immune transducer, STING is expressed in several cell types, both immune and non-immune, and the sequence of STING activation and the identity of its signaling partners may depend on the cell type [22]. The larger and more polar CDNs (mw ~700–1000 amu) have multiple binding interactions with STING causing a large and complex change in conformation [23]. Mimicking this with small molecules is not trivial. Computational approaches such as docking, which are based on the interaction of a single ligand with its target, are confounded by the fact that STING forms a homodimer and small molecules are likely to bind in pairs, similar to the mode of binding of DMXAA with murine STING which was determined from crystal structures [23]. Finally, unlike enzymes and receptors for which established techniques can demonstrate ligand-protein interaction, STING is an adaptor. Conclusive demonstration that a compound has a *direct* action on STING and does not stimulate IRF3/NFκB driven transcription by indirect means through ancillary factors requires a combination of biochemical and biophysical approaches.

**Fig 1:**
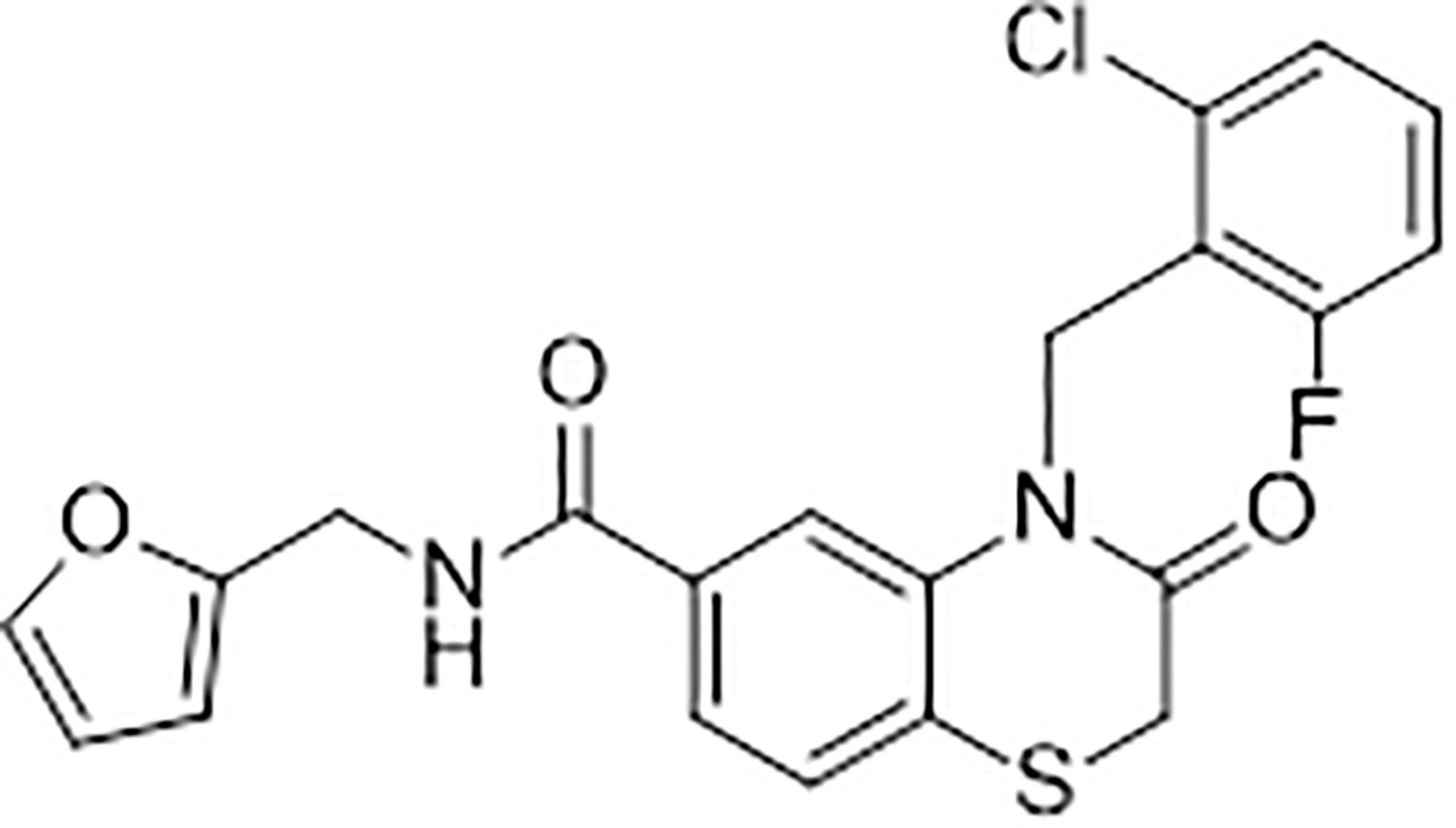
G10. Chemical structure of G10.

Reports of small molecule human STING agonists are relatively recent in the literature. An early report was from Sali et al. [24] in 2015, who used a high throughput screen designed to detect IRF3 activation in immortalized human fibroblasts and identified a weak activator of STING which they named G10 (Fig 1). G10 activity was STING dependent since the compound was inactive in STING knockout fibroblasts. G10 also induced several pro-inflammatory genes including those from the NFκB pathway in human PBMCs and umbilical endothelial cells but unexpectedly did not activate the NFκB pathway in the immortalized human fibroblasts. Apart from its inability to stimulate NFκB in human fibroblasts, G10 was also unable to activate either IRF3 or NFκB linked reporters in the human monocytic cell line THP1. Further, there was no evidence of direct engagement of G10 with the ligand binding domain of the R232 variant of STING in a differential scanning fluorimetry (DSF) assay. From these results, the authors concluded that IRF3 activation and IRF3-dependent transcriptional activity in response to G10 occurred via a STING-dependent pathway but *not* through the direct activation of STING by G10.

Intrigued by this report, a fresh analysis of G10 was undertaken at Curadev as part of a quest for small molecule leads for its internal STING agonist program. In the current report we use human cell lines overexpressing the common R232 variant of STING and cell-free two-protein in vitro kinase assays to demonstrate that G10 is indeed a direct STING agonist. We also describe our initial efforts to improve the potency, pan-isoform selectivity and physicochemical properties of the G10 pharmacophore against human STING.

## Materials and methods

### Reagents

All reagents were procured from Sigma Aldrich unless otherwise mentioned.

### Test and control compounds

All test compounds were synthesised by Curadev. Full synthetic details are provided in the Supplementary Information section. DMXAA (5,6 dimethyl xanthenone 4-acetic acid) was purchased from TCI Chemicals (Cat# D5235) and ADU-S100 was purchased from Medchem Express (Cat# HY12885B).

### Cell lines

HEK293T cells were procured from ATCC. THP-1 and THP-1-Dual KI-hSTING-R232 cells which contained the R232 version of STING was knocked in to THP1 cells in which the HAQ version was ablated, were procured from Invivogen, CA, USA.

### Stable cell line generation

a) **Stable STING expressing cells.** Stable HEK293T STING-expressing cell lines were generated using plasmids purchased from Invivogen, CA, USA, that contain STING cDNA cloned into the pUNO-1 vector under the hEF1-HTLV promoter as well as the Blasticidin selection cassette. The human STING plasmids corresponding to the R232, H232 and HAQ variants were directly procured from Invivogen while the AQ and Q variants were created by mismatch PCR using cDNA from HAQ.STING and R232.STING variants respectively. These vectors were individually transfected into HEK293T cells using Lipofectamine (Invitrogen) and transfected cells were expanded under Blasticidin selection. These cells were further subjected to clonal selection using the limiting dilution method to obtain clonally pure populations of HEK293T cells expressing each of the five human STING variants. Clones with minimal ligand independent activation of STING were chosen for cellular experiments.
b) **N-His R232.hSTING HEK293T cells.** HEK293T cells stably expressing N-His R232.hSTING were generated by the following described method. N-His R232.hSTING.pcDNA Myc B plasmid was transfected into HEK293T cells using Lipofectamine reagent (Invitrogen) and the transfected cells were selected under Hygromycin. Stably STING expressing HEK293T cells thus selected were further subjected to single cell clonal selection using the limiting dilution method to obtain a clonally pure population of HEK293T cells expressing the human R232 STING variant.

### Luciferase assay in recombinant HEK-293T cells

5 x 10^5^ clonally selected HEK293T-hSTING-Luciferase cells or control HEK293T-Luciferase cells were seeded in 384-well plates in growth medium and stimulated with known STING agonists or novel compounds. Supernatants were removed after 20 hours of treatment and secretory reporter gene activity was measured using the Quanti-Luc detection system (Invivogen) on a Spectramax i3X luminometer.

### Western blot assay

5 x 10^5^ cells were seeded in 24-well plates in 500 μl growth medium and stimulated with novel compounds at 10μM. After 2 hours of treatment cells were harvested by centrifugation and cell pellets were lysed in RIPA buffer (20mM Tris-Cl, 150mM NaCl, 0.5mM EDTA, 1% NP40, 0.05% SDS) containing 1x phosphatase inhibitor cocktail 3 (Sigma) and 1x protease inhibitor (Roche) to extract the soluble fraction of protein. 10 μg of extracted protein was electrophoresed in 10% SDS-PAGE gels and transferred onto Immobilon-P membranes (Millipore). Blots were incubated with antibodies specific for phosphorylated STING (Ser366), phosphorylated IRF3 (Ser396), total STING, Actin (Cell Signaling) and IRF3 (Abcam) as shown in Table 1. Anti-rabbit HRP label secondary antibody (Abcam) and Clarity Max^™^ western ECL substrate (Biorad—cat# 1705062) were used for visualization of bands using a BioRad XRS *plus* imager.

**Table 1:** Antibodies used in the immunoblotting experiments.

Antibodies	Vendor	Cat #	Raised in	Dilution used	Diluent
1° Ab–human pSTING^Ser366^	Cell signalling (CST)	19781	Rabbit	1:2500	5% BSA
1° Ab–mouse pSTING^Ser365^	CST	72971	Rabbit	1:2500	5% milk
1° Ab–STING	CST	136475	Rabbit	1:5000	5% BSA
1° Ab–pIRF3^Ser396^	CST	4947S	Rabbit	1:5000	5% BSA
1° Ab–pIRF3	Abcam	ab68481	Rabbit	1:5000	5% milk
1° Ab–ACTIN	CST	8H10D10	Rabbit	1:5000	5% milk
Anti-Rabbit 2° Ab—HRP	Abcam	ab97200	Goat	1:10000	5% milk

The primary antibodies were incubated either at RT for 1 h or at 4°C overnight. The secondary antibody was incubated at RT for 1 h.

### Isolation of ER (endoplasmic reticulum) from cells

100 million N-His R232.hSTING HEK293T or R232.hSTING cells were resuspended in 4 ml chilled hypotonic homogenization buffer (final composition: 10 mM Tris-HCl, 10 mM KCl, 1.5 mM MgCl_2_, 0.5mM EGTA, 1 mM Na_3_VO_4_, 1X protease inhibitor cocktail (Roche), 1X Phosphatase cocktail3 (Sigma), pH 7.5) and kept on ice for 15 min. This was followed by homogenization 3 times with a 30 sec each pulse and 1 min cooling between each pulse. Homogenates were centrifuged at 5000g for 10 min at 4 °C to remove nuclei, mitochondria and cell membrane from the cytosolic fraction. Supernatant was further centrifuged at 100000g for 1 h at 4 °C to fractionate the ER containing cytosolic fraction. The pellet thus obtained was resuspended in homogenization buffer and washed by centrifugation at 100000g twice. Post washing, the ER fraction containing pellet was resuspended in ER buffer (final composition: 20 mM Tris-HCl, 100 mM NaCl, 5 mM MgCl_2_, 5 mM MnCl_2_, 20 mM beta Glycerophosphate, 0.2 mM Na_3_VO_4_, 1X protease inhibitor cocktail (Roche), pH 7.5) using a syringe fitted with a 26G needle passing through it several times till homogeneity. The protein content of the ER homogenate was estimated by the BCA method and aliquots were stored at -80 °C for further use.

### Ligand induced change in STING melting temperature

Two sources of STING (ER fraction of N-His R232.hSTING HEK293T and R232.hSTING HEK293T whole cells) were used for melting temperature experiments. (a) 5 μl of isolated ER (8mg/ml) was stimulated with serially diluted concentration of agonist in a 15 μl reaction volume. After 1 h of stimulation, the reaction mixture was transferred into 0.2 ml PCR-tubes and heated in a gradient thermocycler (Biometra) for 3 min. (b) 5 x 10^5^ R232.hSTING cells were seeded in 24-well plates in 500 μl growth medium and stimulated with serially diluted concentrations of agonist. After 2hr of stimulation cells were harvested through centrifugation and 20 μl cells suspensions were transferred into 0.2 ml PCR-tubes and heated as above. All procedures were identical post-heating of samples. After a subsequent incubation of 5 min. at room temperature, the ER mixture was solubilized by adding 30 μl of 1.5X solubilization buffer (final composition: 50 mM Tris-HCl, 100 mM NaCl, 0.2% NP-40, 5% glycerol, 1.5 mM MgCl_2_, 25 mM NaF, 1 mM Na_3_VO_4_, 1 mM phenylmethylsulfonyl fluoride, 1 mM dithiothreitol (DTT), 1X protease inhibitor cocktail (Roche), 1X Phosphatase cocktail3 (Sigma), pH 7.5). Precipitated proteins were separated from the soluble fraction by centrifugation at 21000g for 20 min at 4 °C. Extracted soluble protein was electrophoresed in 10% SDS-PAGE gels and transferred onto Immobilon-P membranes (Millipore). Blots were incubated with antibodies specific for total STING (Cell Signaling). Anti-rabbit HRP label secondary antibody (Abcam) and Clarity Max^™^ western ECL substrate (Biorad—cat# 1705062) were used for visualization of bands with a BioRad XRS *plus* imager.

### In vitro kinase assay

50ng of recombinant STING protein (Cayman Chemicals Cat# 22816) corresponding to the soluble domain of STING (138 to 379 aa) protein or 1 μl of ER fraction (8mg/ml) containing full length STING from R232.hSTING was incubated with 20 ng of recombinant full length TBK1 protein (Invitrogen, Cat# A31514) in the presence/absence of test compound in 20 μl reaction buffer (50mM Tris-HCl of pH 7.4, 100mM NaCl, 5mM MgCl_2_, 5mM MnCl_2_, 10% glycerol, 0.2mM Na_3_VO_4_, 20mM β-glycero-PO_4_, 0.5mM ATP) and 0.01% BSA at 30 °C for 45 min. The reaction was quenched by addition of 2 μl of 10X EDTA to a final concentration of 50 μM. Samples were treated with 4x gel loading dye followed by electrophoresis in 10% SDS-PAGE gels and transferred to Immobilon-P membranes (Millipore). Blots were incubated with antibodies specific for phosphorylated STING (Ser366) and total STING (Cell Signaling). Anti-rabbit HRP labelled secondary antibody (Abcam) and Clarity Max^™^ western ECL substrate (Biorad, Cat# 1705062) were used for visualization of bands using a BioRad XRS *plus* imager.

## Results

**Fig 2:**
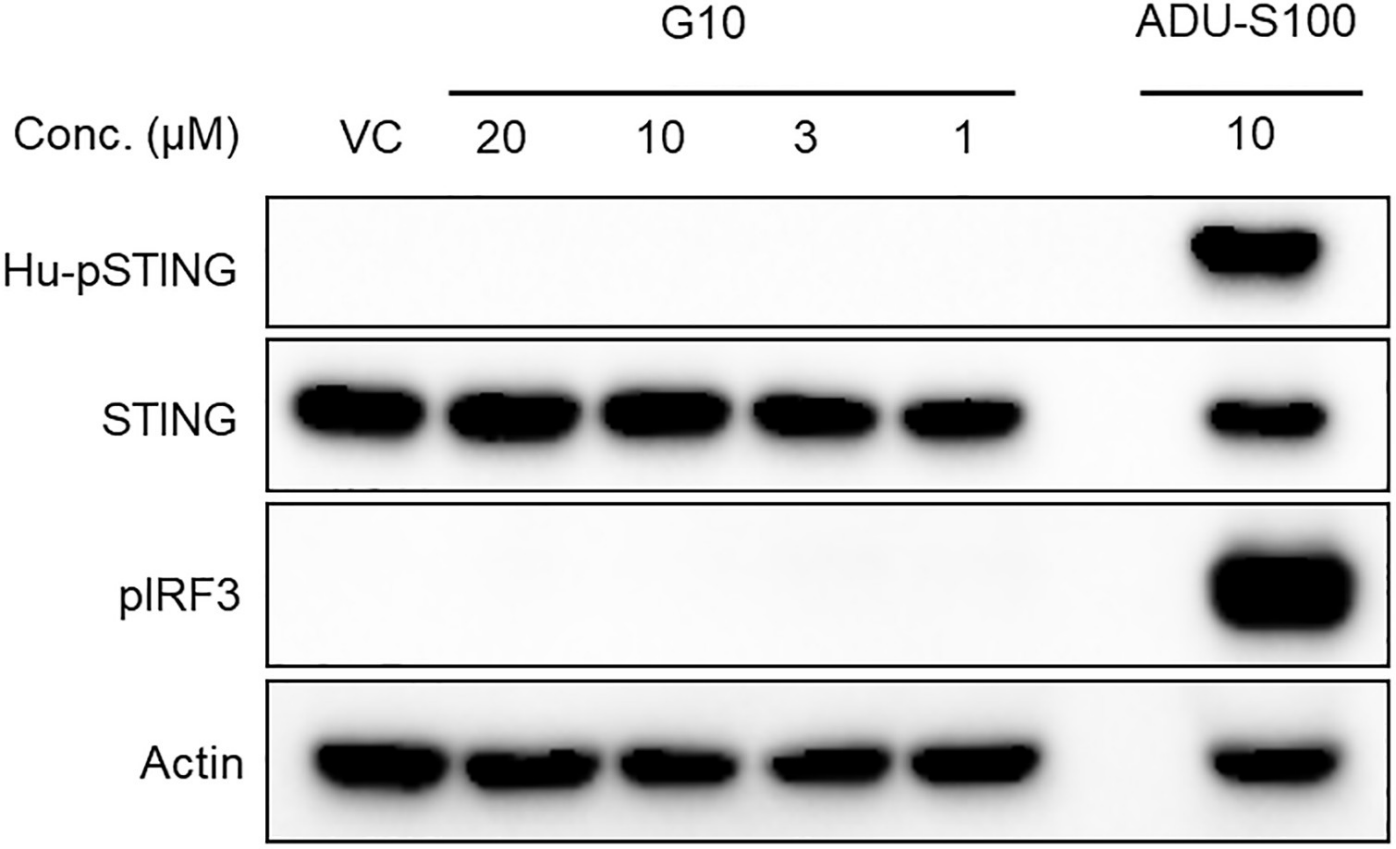
G10 does not promote phosphorylation of HAQ STING or IRF3 in THP-1 cells. Cells were incubated with G10 for 2 hours and then proteins harvested and analyzed by immunoblotting using human pSTING, STING, pIRF3 and actin antibodies. ADU-S100 was used as a positive control.

The structure of G10 was related to some flavone chemotypes we had previously designed and synthesized as potential STING agonists in which aryl groups were attached to a bicyclic core with similar orientation vectors. We first tested for the ability of G10 to activate either the IRF3 or the NFκB pathway in the human monocytic THP1 cell line by monitoring the formation of phosphorylated STING or IRF3 in these cells. Confirming the findings of Sali et al. who used reporter based functional assays, G10 did not activate IRF3 or NFκB signalling in THP1 cells. High concentrations of G10 had been used in that report but G10 had a maximum solubility of 20μM under our assay conditions in which we too were unable to detect either pSTING or pIRF3 in THP1 cells (Fig 2).

**Fig 3:**
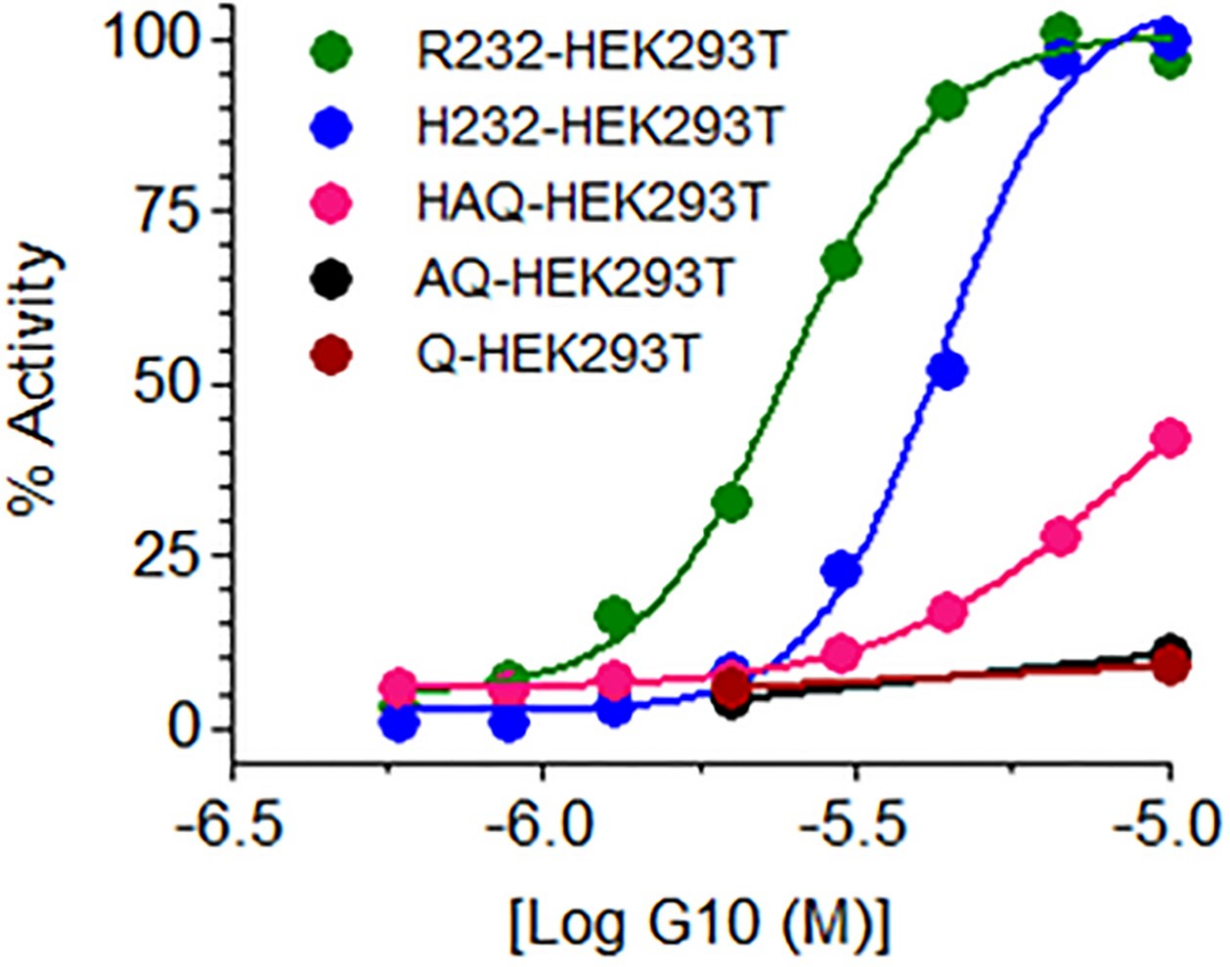
G10 activity is STING variant dependent. G10 activates the luciferase coupled ISRE.ISG54 reporter system in R232, H232, HAQ variants of human STING to varying degrees in stably transfected HEK293T cells. Cells were incubated with G10 for 18 hours and luciferase activity was estimated.

THP1 cells constitutively express the HAQ variant of human STING (~20% allele frequency). The inactivity could be because (a) G10 is not a STING agonist, (b) G10 does not activate the HAQ STING variant or (c) that THP1 cells are somehow resistant to G10. Since G10 had been shown to activate STING in human fibroblasts, we decided to test G10 against other variants of human STING in stable cell lines overexpressing these variants. Cells overexpressing human STING variants were anticipated to be more sensitive to agonists than cell lines with constitutive levels of STING such as THP1. The human embryonic kidney cell line (HEK293T) was chosen as a template to assess STING activation since it lacks functional human STING and does not respond to CDNs. Further, these cells lack other DNA adaptor/sensors such as DAI and IFI16 and cannot synthesize CDNs since they do not express a functional cGAS [25]. As anticipated, parent HEK293T cells were unresponsive to CDNs and G10 (S1 Fig). We then prepared and tested HEK293T cell lines expressing each of five human STING variants and a luciferase reporter driven by an ISRE.ISG promoter and found that G10 activated IRF3 with potency that was variant dependent (Fig 3). G10 was more potent against the R232 and H232 variants with an estimated EC_50_ = 2.5μM and 4.3μM respectively but was less active against the HAQ variant which is carried by THP1 cells and barely active against the Q and AQ variants of STING.

**Fig 4:**
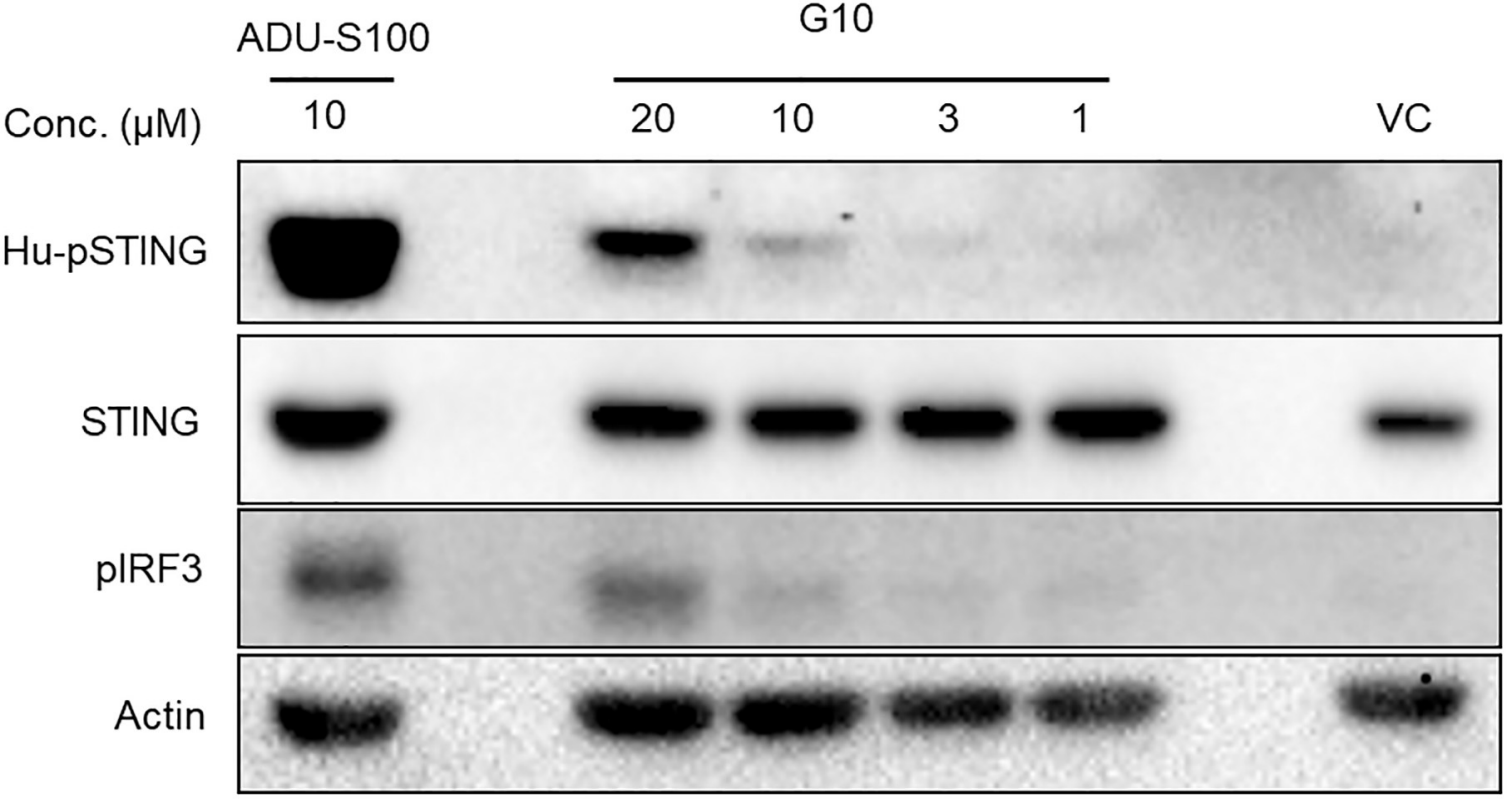
G10 promotes phosphorylation of STING pathway proteins in THP1-R232 cells. G10 treatment leads to phosphorylation of the R232 variant of human STING in THP1 knock in cells. Cells were incubated with G10 for 2 hours and then proteins harvested and analyzed by immunoblotting using human pSTING, STING, pIRF3 and actin antibodies. ADU-S100 was used as a positive control.

The weak activation of HEK293T cells stably transfected with HAQ STING was investigated since G10 did not activate the THP-1 line which expresses HAQ STING constitutively. One clear difference in these lines is the amount of STING, which is far greater in the transfected HEK293T cells. In fact, when we used a commercially available THP-1 cell line (Invivogen) that overexpressed R232 STING in an HAQ knockout background we found that G10 treatment led to phosphorylation of STING and IRF3 (Fig 4). Taken together, these experiments indicated that G10 activity was directly caused by interaction with STING, though G10 was not able to activate all human STING variants.

**Fig 5:**
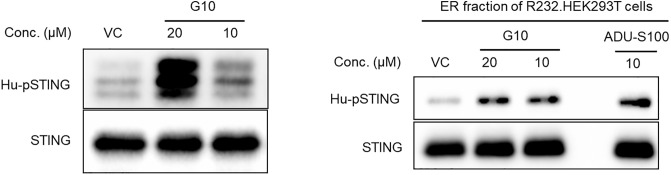
Cell free phosphorylation of CTT domain of STING. (Left panel) Immunoblot analysis of the cell free in vitro kinase assay conducted in ATP containing kinase buffer with recombinant CTT domain of R232 STING and recombinant hTBK1 enzyme. Addition of G10 leads to phosphorylation of the CTT domain by TBK1. (Right panel) Similar experiment carried out using STING protein isolated from the ER fraction of R232-HEK293T cells.

We then sought evidence of direct and productive interaction between R232.STING and G10 by monitoring phosphorylation of R232 STING by recombinant TBK1 in the presence of G10. A simplified three component assay [26] with STING, TBK1 and G10 was designed to demonstrate that agonist activated STING is phosphorylated by TBK1. This assay was based on the demonstration of in vitro phosphorylation of STING by TBK1, using ADU-S100 as a positive control (S2 Fig). Two separate sources of STING were tested in this assay. The first was the recombinant CTT domain of R232 STING (Cayman Chemical) and the second was the ER fraction from R232.STING transfected HEK293T cells which contained full length STING. Each of these STING preparations was incubated with recombinant TBK1 in the presence of G10 and ATP and the reaction was then electrophoresed by SDS-PAGE followed by western blotting to detect pSTING. Both sources of STING were activated by G10 resulting in the formation of pSTING by TBK1 (Fig 5). The possibility that G10 was a direct activator of TBK1 was tested and ruled out by carrying out a TBK1 kinase assay in the presence of G10 (S1 Table). Detection of pSTING by western blotting in this in vitro enzyme assay provided proof of a functionally productive agonist driven interaction between STING and TBK1 and decisively identified G10 as an agonist of the most common human variant of STING. Contrary to the conclusions of the earlier report [24], we here establish that G10 is a direct STING agonist.

**Fig 6:**
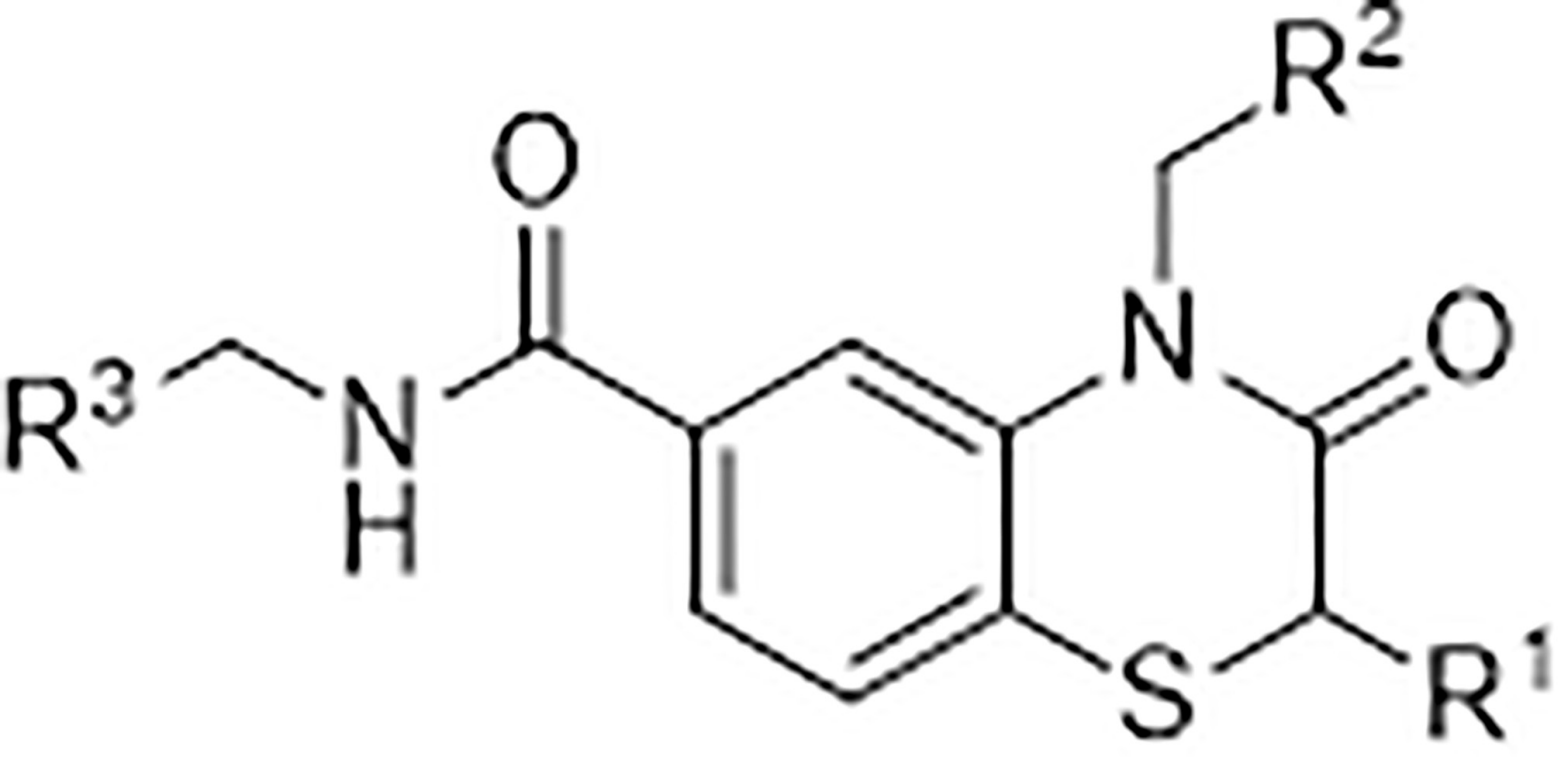
Sites of G10 modification. Structural analogues were designed and synthesised to increase potency in the benzothiazinone core at the R groups indicated.

We immediately initiated a chemical exploration of the G10 pharmacophore and focused on the R^1^, R^2^ and R^3^ regions of the molecule (Fig 6).

The first replacements were at R^2^ where we found that very close analogs of G10 (Compounds 1–3) with minor modifications of R^2^ maintained weak activity against the R232 variant of STING and did not show activation of THP1 cells (Table 2).

**Table 2:** Close analogs of G10 activate R232 STING.

Compound ID	R^1^	R^2^	R^3^	HEK.R232 EC_50_ (nM)	THP-1 EC_50_ (nM)
**G10**	H	2-Cl-6-FPh	2-furan	2490	Nil @ 30uM
**1**	H	2,6-diClPh	2-furan	2160	Nil @ 30uM
**2**	H	2,6-diFPh	2-furan	3970	Nil @ 30uM
**3**	H	Ph	2-furan	9130	Nil @ 30uM

The sites of structural modification refer to those depicted in Fig 6.

We then varied the R^3^ functionality and found that replacing the furan heterocycle with fluorophenyl groups caused a significant change in potency against R232.hSTING. Interestingly we also observed very weak activation of the THP1 line (Table 3, Compounds 4 and 5). Further alterations of the R^2^ functionality to 2,6-diF-4-OMe-phenyl and 2,6-diF-4-OH-phenyl in conjunction with R^3^ as 2,4,6-trifluorophenyl led to sharp increases in activity in the R232 assay (Compounds 6 and 7) and in the latter case significant activity was now observed in the THP1 cell assay.

**Table 3:** Structural analogues potently activate R232 STING and activate HAQ STING.

Compound ID	R^1^	R^2^	R^3^	HEK.R232 EC_50_ (nM)	THP-1 EC_50_ (nM)
**4**	H	2-Cl-6-FPh	2,4-diFPh	292	> 30000
**5**	H	2-Cl-6-FPh	2,4,6-triFPh	188	> 10000
**6**	H	2,6-diF-4-OMePh	2,4,6-triFPh	159.7	> 10000
**7**	H	2,6-diF-4-OHPh	2,4,6-triFPh	71.6	1380

The sites of structural modification refer to those depicted in Fig 6.

We then substituted a single methyl group at the R^1^ position and created variants around R^2^ and R^3^ (Table 4). These compounds were racemic mixtures except for Compounds 10 and 11 which were separated as chirally pure enantiomers, though the absolute configuration was not determined. Compounds 8, 9 and 11 showed sub-micromolar activity in both the R232 and THP1 cellular assays. The two enantiomers, Compound 10 and Compound 11, varied significantly in potency suggesting a critical role of R^1^ in the interaction with STING.

**Table 4:** Structural analogues potently activate R232 and HAQ STING.

Compound ID	R^1^	R^2^	R^3^	HEK.R232 EC_50_ (nM)	THP-1 EC_50_ (nM)
***rac* 8**	Me	2-Cl-6-FPh	2,4,6-triFPh	122	944
***rac* 9**	Me	2-Cl-6-F-3-OHPh	2,4,6-triFPh	96	412
**10- *enant 1***	Me	3,5-diFPh	2,4,6-triFPh	421	~6000*
**11- *enant 2***	Me	3,5-diFPh	2,4,6-triFPh	66	967
***rac* 12**	Me	3,5-diFPh	2-benzofuran	91	~8000*

The sites of structural modification refer to those depicted in Fig 6.

*Low compound solubility did not allow measurement of a full dose response curve.

**Fig 7:**
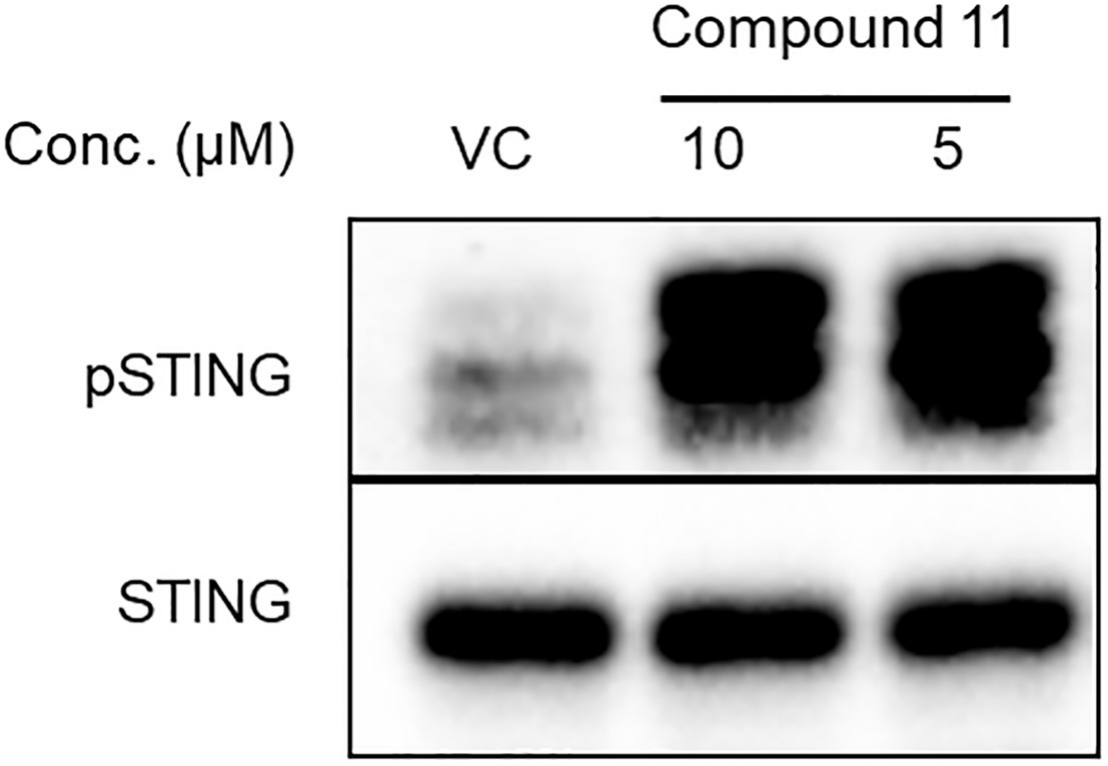
Compound 11 is a potent STING agonist in the cell free pSTING assay. Compound 11 is a direct STING agonist. Immunoblots showing Compound 11 dependent phosphorylation of recombinant CTT domain of STING by TBK1 (Lane 1: No agonist, Lanes 2, 3: Compound 11 at 10 and 5μM respectively).

To confirm that the functional activities observed were due to agonism of the STING protein, the cell free two-component kinase assay was carried out using Compound 11 with both recombinant CTT domain of R232.hSTING and endoplasmic reticulum membranes of transfected cells that contained full length R232.hSTING. Compound 11 showed significantly enhanced potency compared with G10 (Fig 7) in both these assays. Results with this more potent analog confirmed that G10 is an agonist of the most common human variant of STING.

We subsequently confirmed activity of a second example compound (Compound 8) in the cell-free assay (S3 Fig). This compound also activated multiple STING variants overexpressed in HEK293T cells measured by luciferase reporter assay or phosphorylation of STING (S4 and S5 Figs), and phosphorylated STING in human PBMCs from multiple donors and human tumor lines (S6 and S7 Figs).

**Fig 8:**
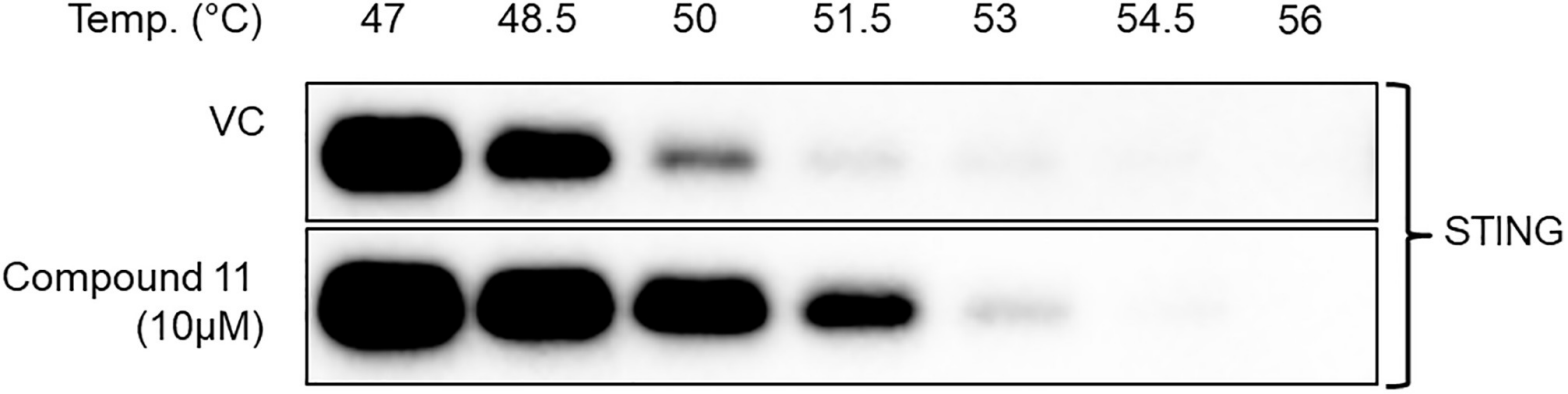
Compound 11 binding leads to STING stabilization in ER. Ligand induced change in STING melting temperature was monitored in N-His.R232.hSTING endoplasmic reticulum (ER) extracted from HEK293T cells overexpressing N-His.R232.hSTING. ER was treated with 10 μM Compound 11 for 30 minutes and then subjected to heating in a gradient thermal cycler at various temperatures for 3 minutes followed by solubilization and immunoblotting.

Further evidence of the direct interaction of STING with Compound 11 was obtained by showing that Compound 11 binding induces changes in STING stability. This was done using ER membranes as a source of STING and by monitoring STING stability as a function of temperature in the presence of compound. Compound 11 caused a discernable shift in the thermal stability of STING providing further evidence of an interaction with STING (Fig 8).

G10 did not demonstrate a significant ligand induced change in STING melting temperature when incubated with R232 STING transfected cells (S8 and S9 Figs). This corroborates the reported failure to detect direct binding of this compound by DSF.

**Fig 9:**
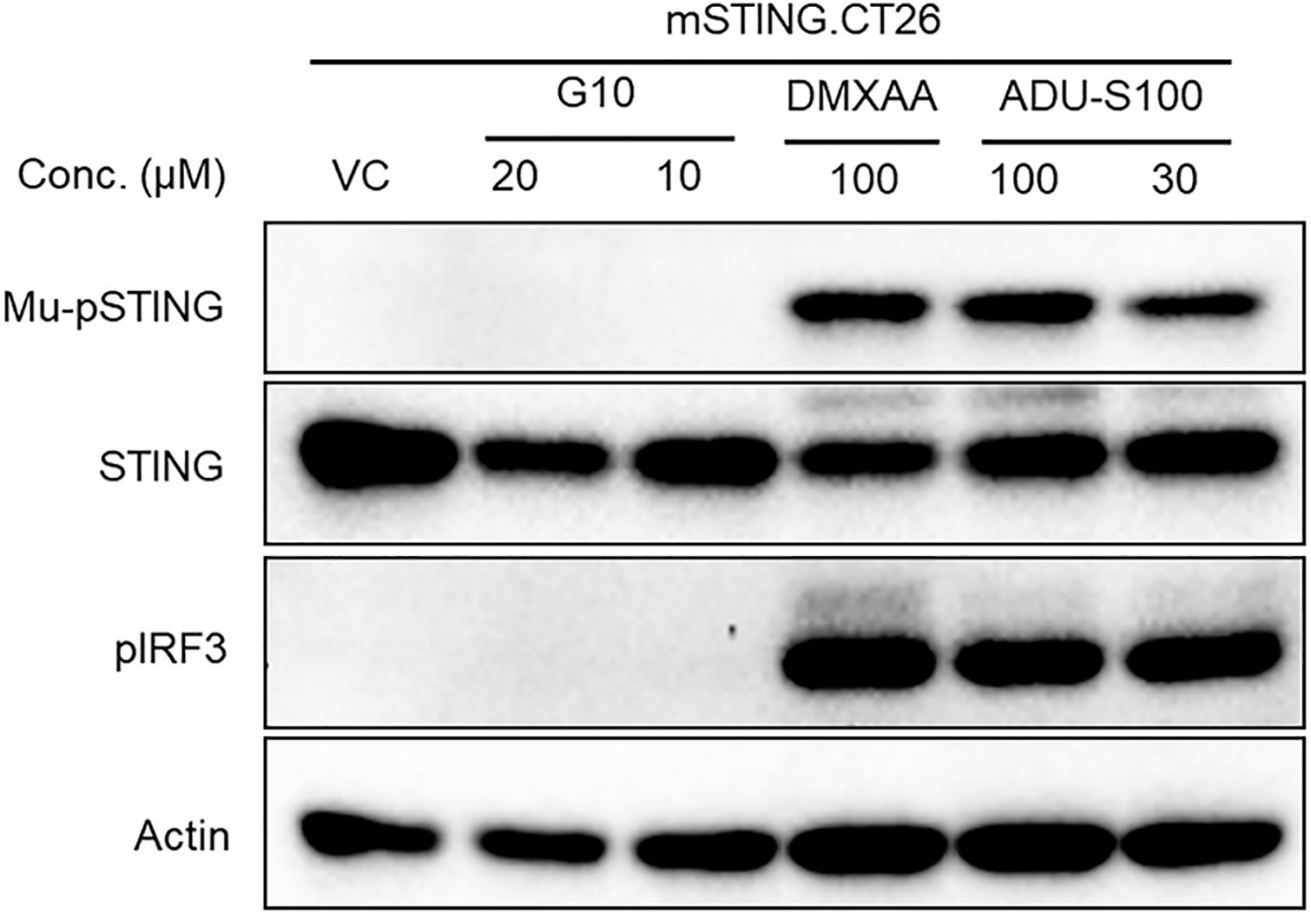
Murine STING pathway proteins are not phosphorylated by G10 treatment. Murine STING and IRF3 in CT26 cells that stably overexpress mSTING are not phosphorylated after G10 treatment. Cells were incubated with G10 for 2 hours and then proteins harvested and analyzed by immunoblotting using murine pSTING and STING and pIRF3 antibodies, with DMXAA (mouse specific) and ADU-S100 (pan STING) as positive controls.

It was postulated that G10 possibly activated STING by indirect means with the assistance of an unidentified factor distinct from STING [24]. To show that G10 activated STING directly and not by interacting with an ancillary human cellular factor, G10 activity was assessed in a murine cell line expressing human STING so that the only human protein in these cells would be hSTING. We chose the CT26 murine line for this experiment since it carries very low background quantities of murine STING and created an R232.hSTING.CT26 cell line by stable transfection. As expected G10 treatment did not lead to phosphorylation of mSTING or mIRF3 in either the parent CT26 line (S10 Fig) or in CT26 cells overexpressing mSTING (Fig 9).

**Fig 10:**
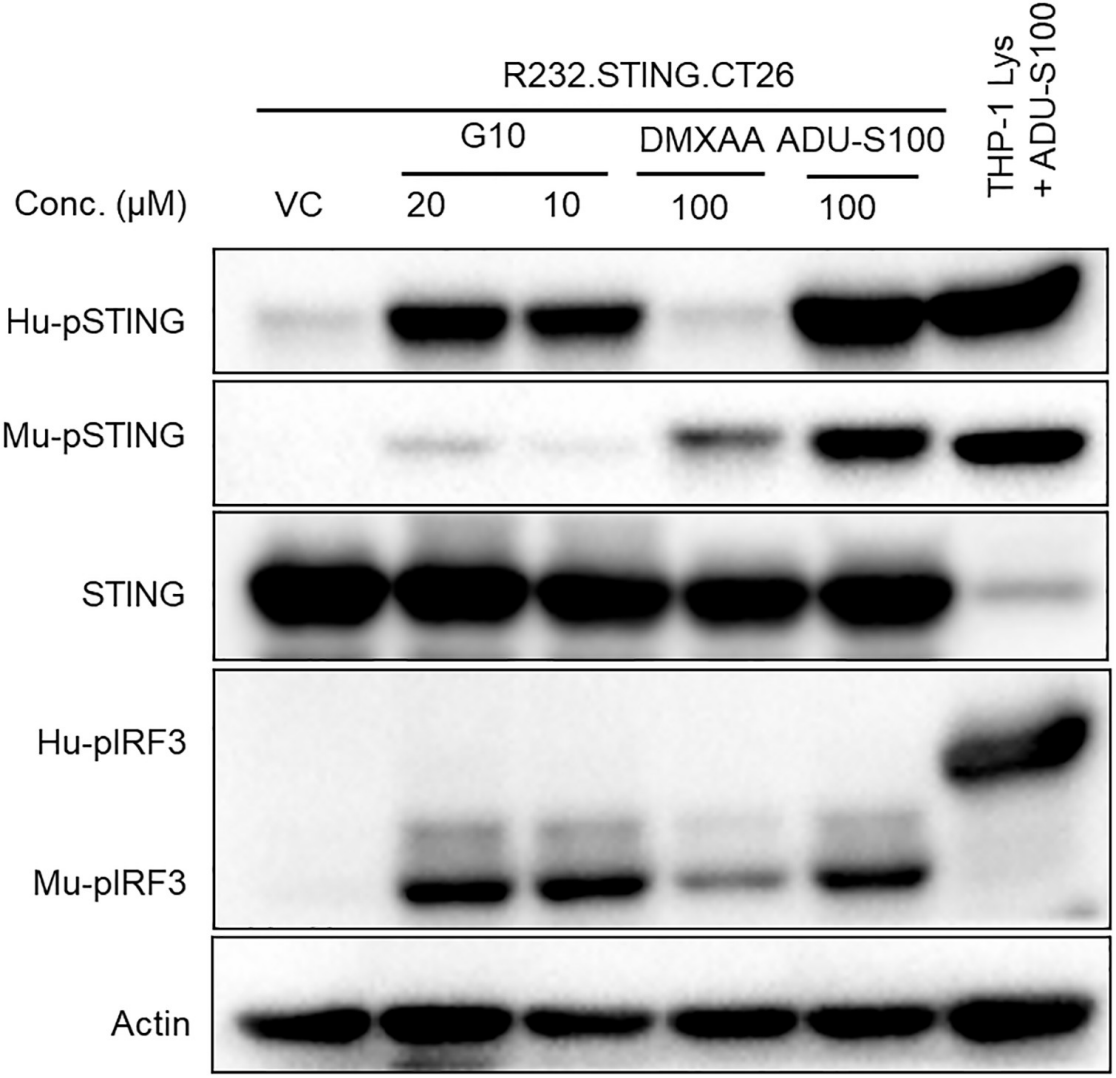
Human STING is phosphorylated on G10 treatment and couples to mouse IRF3. R232.hSTING.CT26 cells were incubated with G10 for 2 hours and then proteins harvested and analyzed by immunoblotting using antibodies againstHu- pSTING, Mu- pSTING, STING and pIRF3(both cross-reactive). ADU-S100 and DMXAA were used as a positive control and a mouse specific control respectively. Left to right: Lane 1: Vehicle control; Lane 2, 3: G10 increases Hu-pSTING and Mu-pIRF3 levels. Lane 4: DMXAA activates endogenous Mu-STING in CT26 (see also Fig 9); Lane 5: ADU-S100 phosphorylates both endogenous Mu-STING and overexpressed human STING; Lane 6: ADU-S100 phosphorylates STING and IRF3 in Hu-THP1 cells. Note that the faint Mu-pSTING band at 20μM G10 (Lane 2) is due to weak cross-reactivity of the Mu-pSTING antibody with Hu-pSTING. This can be seen in lane 6 where the Mu-pSTING antibody recognizes Hu-pSTING.

In contrast, significant amounts of phosphorylated hSTING and mIRF3 were detected when CT26 cells overexpressing R232.hSTING were treated with G10 (Fig 10). This result ruled out the possibility that G10 activates STING by interacting with a factor from human cells in a STING dependent manner and not with STING directly since the R232.hSTING.CT26 cells contained no human proteins other than STING. It confirmed what was already evident from the kinase assay. G10 works directly through STING and not through an ancillary factor in cells. Interestingly, this experiment also provided evidence that human STING can activate mouse TBK-1 to phosphorylate mouse IRF3, which is in agreement with previous findings [27].

## Discussion

### Therapeutic potential

Cyclic di-nucleotide based STING agonists are currently under clinical investigation for the treatment of a variety of cancers [18,19]. The administration of STING agonists with minimal triggering of non-tumor innate immune responses is a therapeutic goal. This can be achieved by direct IT injection to limit systemic exposure or by the use of tumor targeting delivery systems such as antibody drug conjugates (ADCs). Though CDNs currently under investigation have been delivered by the IT route, their polar nature causes them to leak out of the tumor into the plasma potentially limiting the doses at which they can be administered [28]. Further, the multi-chiral macrocyclic structures of CDNs provide manufacturing challenges. For these reasons there is a need for small molecule STING agonists that have low tumor leakage so that they can be administered either IT or by tumor targeting approaches.

### Proving direct binding of a STING agonist

The binding of cGAMP leads to post-translational modifications and translocation of STING to the Golgi where it interacts with TBK1 which phosphorylates itself, STING and IRF3 [29]. However, TBK1 can be activated in a STING independent manner [30] and so proving that a functional agonist works by directly activating STING and not through an associated protein requires additional confirmation. It is not possible to confirm STING binding by cellular functional assays in isolation or even in conjunction with knockout lines and so cell-free techniques need to be used to conclusively demonstrate the interaction of STING with an agonist. Biophysical measures of protein-ligand interaction such as differential scanning fluorimetry (DSF) or isothermal calorimetry (ITC) can be useful but cannot be performed with full length STING. Moreover, while these assays can confirm the presence of binding they cannot be used to determine whether binding leads to a productive functional activation of STING. Conversely, the absence of binding cannot be reliably inferred from the absence of a DSF or ITC signal [31]. One way of providing an unequivocal demonstration of productive binding by a STING agonist is the in vitro demonstration of agonist dependent phosphorylation of STING by TBK1 [26]. To this end we designed a STING-TBK1 kinase assay that monitors STING phosphorylation in the presence of an agonist and in the absence of other cellular components.

### G10 is a STING agonist

G10 was originally identified as a human STING dependent activator of the IRF3 pathway whose direct action was on a yet unknown factor distinct from human STING [24]. The molecule was non-polar in nature and structurally distinct from DMXAA, a murine specific small molecule STING agonist [20].

We decided to re-examine G10 since its structure had some similarities to pharmacophores that we were designing for an internal STING agonist program. We first reproduced G10’s failure to function in THP1 cells that carried the HAQ variant of STING by directly monitoring the phosphorylation of STING and IRF3. In contrast, G10 did activate R232.STING in stably transfected HEK293T cells, a result similar to one obtained in human fibroblasts [24]. Suspecting that G10 might exhibit differential potency against the various human STING variants, we prepared a panel of cell lines each carrying stably integrated variants of STING. In these cell lines STING was expressed under the viral hEF1-HTLV promoter and so levels of STING were significantly higher than in cell lines such as THP1. Using these cells as a sensitive tool we determined that G10 activated the R232 and H232 variants of STING with low micromolar potency but had substantially reduced ability to activate the HAQ variant of STING, which incidentally is found in THP-1 cells. G10 also caused the phosphorylation of IRF3 and STING in the THP-1 STING knockout cell line engineered to overexpress R232.hSTING. This strongly indicated that G10 was a STING agonist.

To rule out the possibility that an ancillary factor in human cells mediated the action of G10 on STING, we demonstrated that G10 was able to activate human STING when it was overexpressed in murine CT26 cells. In order to prove direct interaction we used a cell free in vitro kinase assay to demonstrate that G10 phosphorylated STING in the presence of TBK1. No other protein or cellular component was used in this assay providing incontrovertible evidence of STING agonism through direct interaction with G10.

Finally, we used G10 as a starting point for a conservative synthesis effort to identify structurally similar G10 analogs that had greater potency against both the R232 and HAQ variants. We were indeed able to identify such compounds in which potency against the HAQ variant in particular was enhanced by more than 1000 fold. One of these molecules, Compound 11, caused STING phosphorylation in the STING-TBK1 kinase assay proving it was a direct STING agonist.

## Conclusions

The work presented in this report lays the foundation for identifying potent, pan-isoform, non-CDN small molecule STING agonists.

## Supporting information

S1 Fig G10 does not stimulate reporter gene expression in HEK293T cells that do not express STING. (TIF)

S2 Fig Cell free TBK-1 kinase assay with ADU-S100 as positive control. (TIF)

S3 Fig Compound 8 phosphorylates STING in a cell free pSTING assay. (TIF)

S4 Fig Reporter gene activation of STING variants by Compound 8. (TIF)

S5 Fig Human STING variants are phosphorylated on Compound 8 treatment. (TIF)

S6 Fig STING in PBMCs isolated from multiple human donors is phosphorylated after treatment with Compound 8. (TIF)

S7 Fig STING in human cell lines is phosphorylated after treatment with Compound 8. (TIF)

S8 Fig G10 does not change the melting temperature of STING in R232-HEK293T cells. (TIF)

S9 Fig ADU-S100 binding causes a significant change in the melting temperature of STING in R232-HEK293T cells. (TIF)

S10 Fig STING pathway proteins in wild type murine CT26 cells are not phosphorylated upon G10 treatment. (TIF)

S1 Table G10 does not inhibit or activate TBK-1 enzyme. (TIF)

S2 Table STING alleles in human cell lines used for assessment. (TIF)

S1 File Materials and methods for compound synthesis. Full synthetic details of Compounds 1–12 and characterisation data are provided.
(DOCX)

S2 File Spectra. Chemical characterisation spectra for Compounds 1–12.
(PDF)

S1 Raw images Raw blot images used to generate figures. (PDF)
