# Supplementary figures and images for: G10 is a direct activator of human STING
Source: PLoS One. 2020 Sep 10;15(9):e0237743. doi: 10.1371/journal.pone.0237743 (PMC7482845; doi:10.1371/journal.pone.0237743)

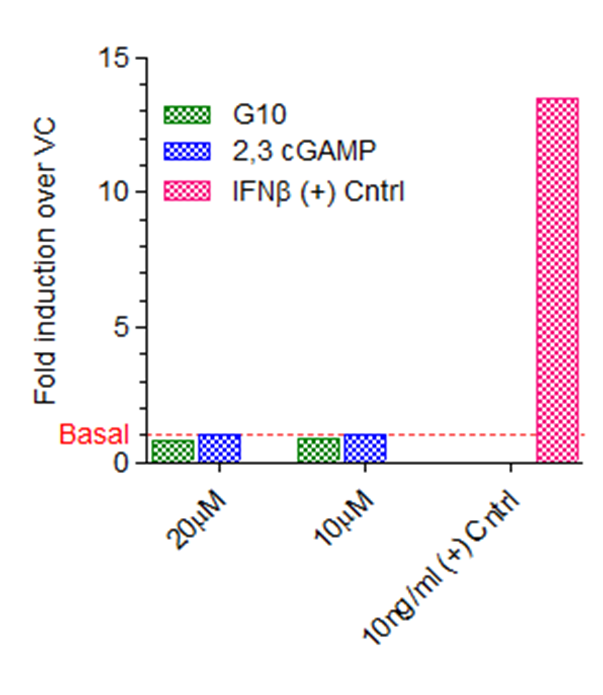

Supplement: S1 Fig — (TIF) [file pone.0237743.s001.tif]

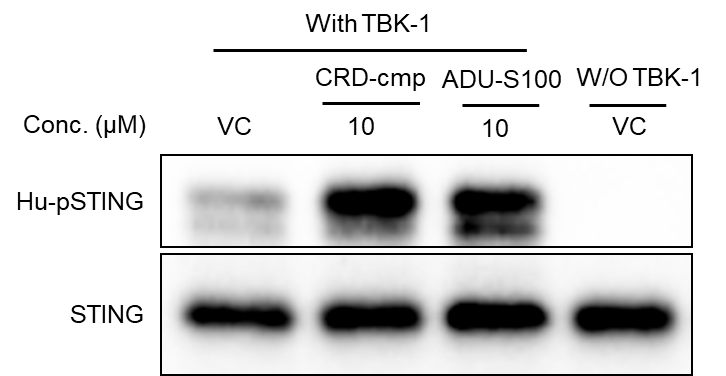

Supplement: S2 Fig — (TIF) [file pone.0237743.s002.tif]

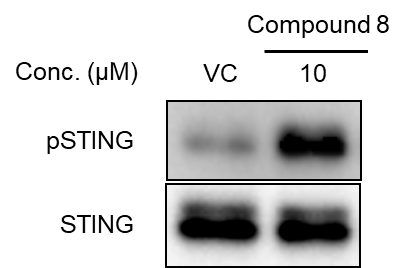

Supplement: S3 Fig — (TIF) [file pone.0237743.s003.tif]

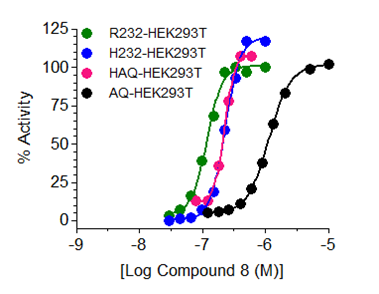

Supplement: S4 Fig — (TIF) [file pone.0237743.s004.tif]

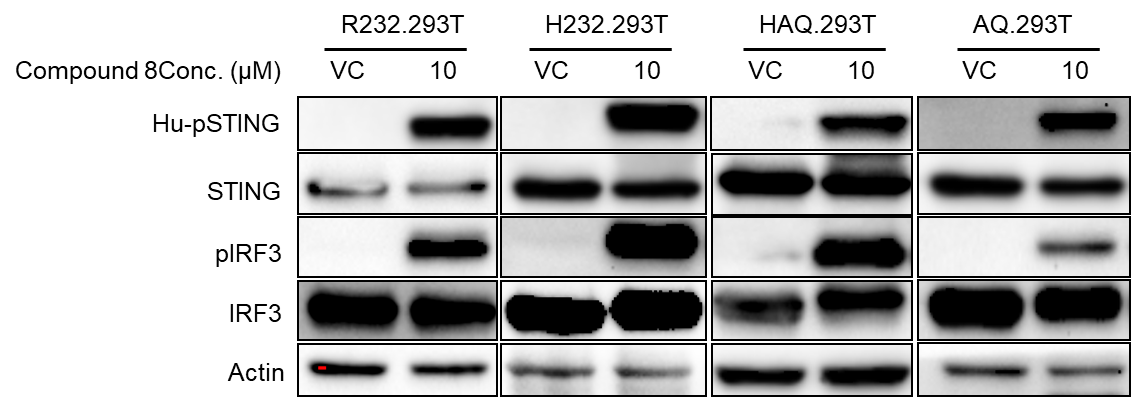

Supplement: S5 Fig — (TIF) [file pone.0237743.s005.tif]

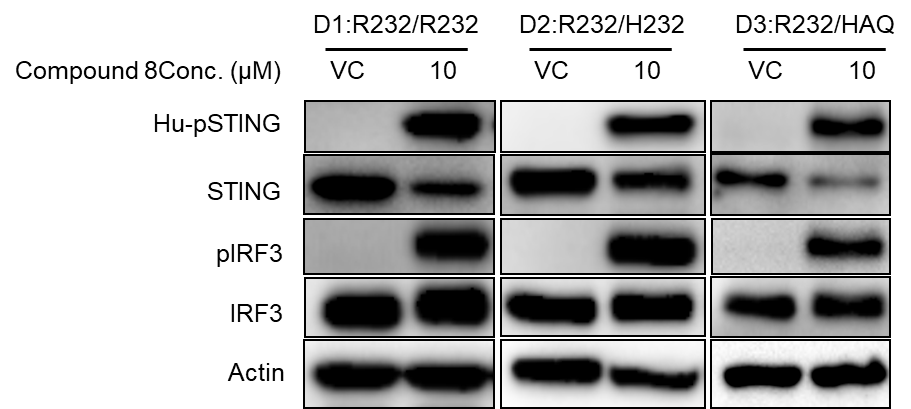

Supplement: S6 Fig — (TIF) [file pone.0237743.s006.tif]

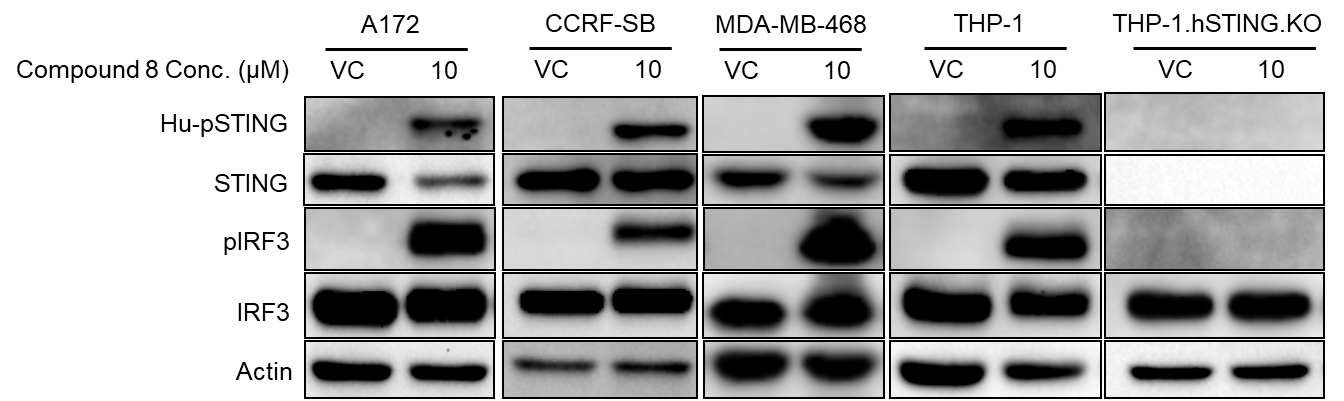

Supplement: S7 Fig — (TIF) [file pone.0237743.s007.tif]

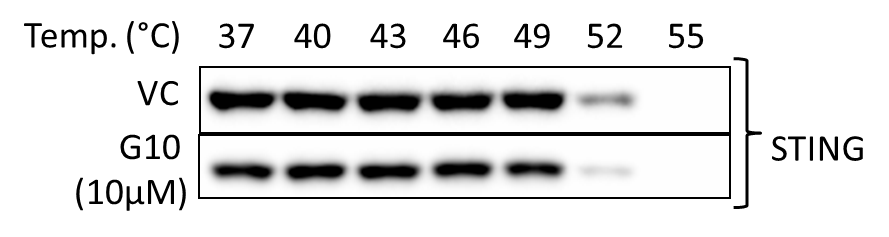

Supplement: S8 Fig — (TIF) [file pone.0237743.s008.tif]

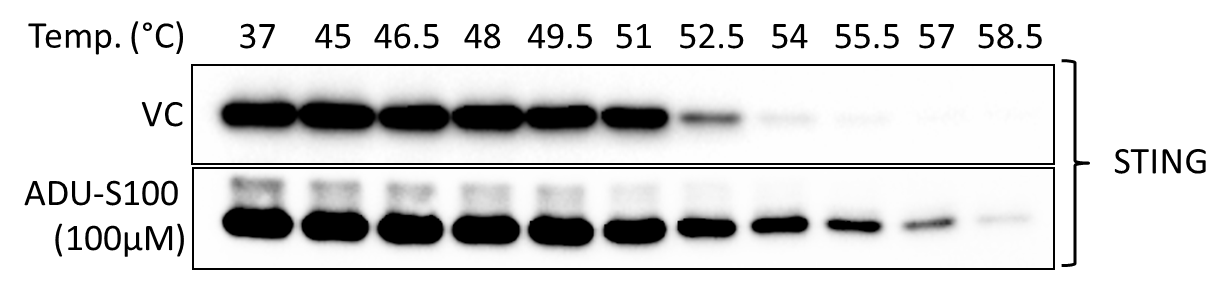

Supplement: S9 Fig — (TIF) [file pone.0237743.s009.tif]

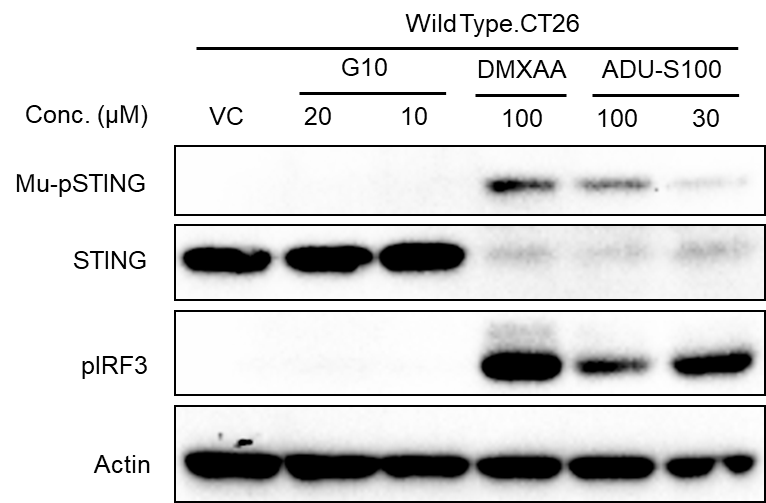

Supplement: S10 Fig — (TIF) [file pone.0237743.s010.tif]

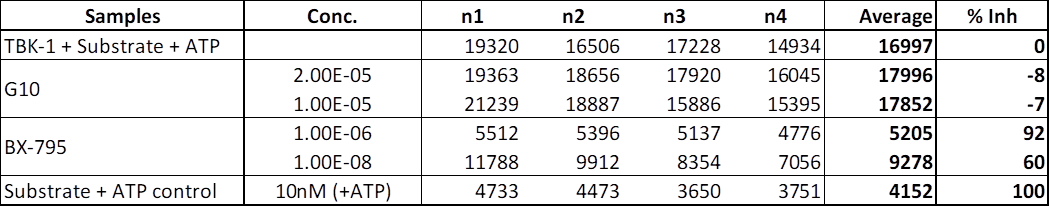

Supplement: S1 Table — (TIF) [file pone.0237743.s011.tif]

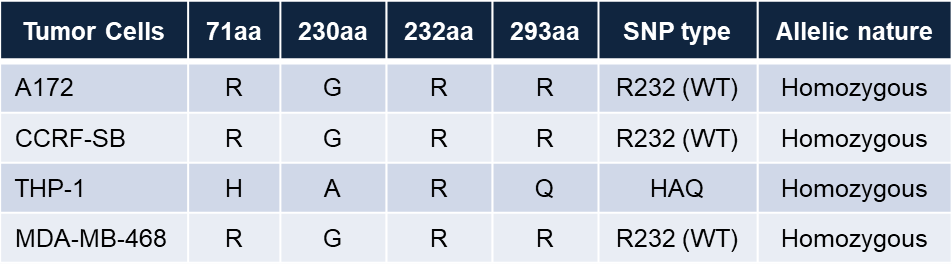

Supplement: S2 Table — (TIF) [file pone.0237743.s012.tif]
