# Supplementary material for: G10 is a direct activator of human STING
Source: PLoS One. 2020 Sep 10;15(9):e0237743. doi: 10.1371/journal.pone.0237743 (PMC7482845; doi:10.1371/journal.pone.0237743)
Supplement: S1 File — Full synthetic details of Compounds 1–12 and characterisation data are provided. (DOCX) [file pone.0237743.s013.docx]

**S1 File. Materials and methods for compound synthesis**

All chemicals, reagents and solvents were purchased from commercial sources and used without further purification. All reactions were performed under an atmosphere of nitrogen unless otherwise noted.

Nuclear magnetic resonance (NMR) spectra were in all cases consistent with the proposed structures. Characteristic chemical shifts (δ) are given in parts-per-million downfield from tetramethylsilane using conventional abbreviations for designation of major peaks: e.g. s, singlet; d, doublet; t, triplet; q, quartet; m, multiplet; br, broad. Spectra were recorded in deuterodimethylsulphoxide; d_6_-DMSO.

Mass spectra, MS (m/z), were recorded using electrospray ionisation (ESI). Where relevant and unless otherwise stated the m/z data provided are for isotopes ^19^F, ^35^Cl, ^79^Br and ^127^I.

Thin layer chromatography (TLC) was carried out on Merck silica gel 60 plates (5729). All final compounds were >95% pure as judged by the LCMS or UPLC analysis methods described in the General Purification and Analytical Methods below unless otherwise stated. Flash column chromatography was carried out using pre-packed silica gel cartridges in a Combi-Flash platform. Prep-HPLC purification was carried out according to the General Purification and Analytical Methods described below.

**General Purification and Analytical Methods**

All final compounds were purified by either Combi-flash or prep-HPLC purification, and analysed for purity and product identity by UPLC or LCMS according to one of the below conditions.

**Prep-HPLC**

Preparative HPLC was carried out on a Waters auto purification instrument using either a YMC Triart C18 column (250 x 20 mm, 5 µm) or a Phenyl Hexyl column (250 x 21.2 mm, 5 µm) operating at between ambient temperature and 50 °C with a flow rate of 16.0 – 50.0 mL/min.

Mobile phase 1: A = 20mM Ammonium Bicarbonate in water, B = Acetonitrile; Gradient Profile: Mobile phase initial composition of 80% A and 20% B, then to 60% A and 40% B after 3 min., then to 30% A and 70% B after 20 min., then to 5% A and 95% B after 21 min., held at this composition for 1 min. for column washing, then returned to initial composition for 3 min.

Mobile phase 2: A = 10mM Ammonium Acetate in water, B = Acetonitrile; Gradient Profile: Mobile phase initial composition of 90% A and 10% B, then to 70% A and 30% B after 2 min., then to 20% A and 80% B after 20 min., then to 5% A and 95% B after 21 min., held at this composition for 1 min. for column washing, then returned to initial composition for 3 min.

**LCMS method**

General 5 min method: Zorbax Extend C18 column (50 x 4.6 mm, 5µm) operating at ambient temperature and a flow rate of 1.2 mL/min. Mobile phase: A = 10 mM Ammonium Acetate in water, B = Acetonitrile; Gradient profile: from 90 % A and 10 % B to 70 % A and 30 B in 1.5 min, and then to 10 % A and 90 % B in 3.0 min, held at this composition for 1.0 min, and finally back to initial composition for 2.0 min.

**UPLC method**

UPLC was carried out on a Waters auto purification instrument using a Zorbax Extend C18 column (50 x 4.6 mm, 5µm) at ambient temperature and a flow rate of 1.5ml/min.

**Method A**

Mobile phase 1: A = 1-5 mM Ammonium Acetate in water, B = 1-5 mM Ammonium Acetate in 90:10 Acetonitrile/water; Gradient profile from 95% A and 5% B to 65% A and 35% B in 2 min., then to 10% A and 90% B in 3.0 min., held at this composition for 4.0 min. and finally back to the initial composition for 5.0 min.

**Method B**

Mobile phase 2: A = 0.1 % formic acid in water, B = Acetonitrile; Gradient profile from 98 % A and 2 % B over 1 min., then 90 % A and 10 % B for 1 min., then 2 % A and 98 % B for 2 min. and then back to the initial composition for 3 min.

**Abbreviations**

AcOH acetic acid

MeCN/CAN acetonitrile

MeOH methanol

TEA triethylamine

DIPEA diisopropylethylamine

EtOAc ethyl acetate

HATU **1-[bis(dimethylamino)methylene]-1*H*-1,2,3-triazolo[4,5-*b*]pyridinium-3-oxide hexafluorophosphate**

HBTU *N,N,N′,N′*-tetramethyl-*O-*(1*H*-benzotriazol-1-yl)uronium hexafluorophosphate,

HOBt 1-hydroxybenzotriazole

THF tetrahydrofuran

DMF dimethylformamide

DCM dichloromethane

DBU [1,8-diazabicyclo[5.4.0]undec-7-ene](https://www.sigmaaldrich.com/catalog/substance/18diazabicyclo540undec7ene15224667422211)

TLC thin layer chromatography

LCMS liquid chromatography-mass spectrometry

HPLC/UPLC high performance liquid chromatography/ultra performance liquid chromatography

**Compound Synthesis Experimental**

**Schematic of synthesis of test compounds**

**General Synthetic Procedures**

**General procedure for the preparation of 3**

To a stirred solution of **1** (1.0 eq.) in MeCN (4 mL/mmol) was added **2** (1.2 eq.) followed by TEA or DBU (1.5 eq.) and the whole stirred at RT for 1 h. After completion of the reaction (monitored by TLC and LCMS) the solvent was evaporated, diluted with water and extracted with EtOAc. The organic layer was dried over anhydrous Na_2_SO_4_, filtered and concentrated *in vacuo* to give **3** (90-100% yield) as a crude solid which was used in the next step without any further purification.

**General procedure for the preparation of 4**

To a stirred solution of **3** (1.0 eq. ) in AcOH (5 mL/mmol) was added iron powder (4.0 eq.) and the mixture stirred at 80-82 °C for 2 h. After completion of the reaction (monitored by TLC and LCMS), the reaction mixture was poured into crushed ice and the precipitated solid was filtered, washed with water and dried to afford **4** (85-88%) as a crude solid which was used in the next step without any further purification.

**General procedure for the preparation of 6**

To a stirred solution of **4** (1.0 eq.) in DMF (4 mL/mmol) was added NaH (1.0 eq., 60% suspension in mineral oil) followed by addition of **5** (1.05 eq.) at 0-5 ^o^C and the mixture allowed to stir at RT for 0.5-1 h. The reaction was monitored by TLC. After completion of the reaction the reaction mixture was quenched with saturated NH_4_Cl solution and extracted with EtOAc. The organic layers were washed with brine and dried over anhydrous Na_2_SO_4._ The organics were evaporated under reduced pressure to obtain the crude product which was purified by Combi-flash using mixtures of EtOAc in hexanes as eluent to afford **6** (70-80% yield) as a pale yellow solid.

**General procedure for the preparation of 7**

To a stirred solution of ester **6** (1 eq.) in a mixture of solvents MeOH:THF:H_2_O (12 mL/mmol, 1:2:1) was added LiOH.H_2_O (4.0 eq.) at RT and the resulting reaction mixture was stirred at RT for 2 h. TLC showed complete consumption of the ester **6**. The solvents were evaporated under reduced pressure and the resulting residue was dissolved in water and acidified with 1N HCl to pH 2-4, which resulted in the formation of a precipitate, which was filtered and washed with water followed by hexane. The obtained solid was dried by azeotropic distillation with MeCN to afford the desired carboxylic acid **7** (70-85% yield) as an off white solid.

**General procedure for the preparation of 9**

To a stirred solution of a carboxylic acid **7** (1 eq.) in DCM/THF/DMF (10 mL/mmol) was added TEA/DIPEA (2 eq.) followed by HATU/HBTU (1.2 eq.) at 0-5 ^o^C and the whole stirred for 5-10 min. then amine **8** (1.1 eq.) was added. The resulting reaction mixture was brought to RT and allowed to stir at RT for 2-3 h. When UPLC or TLC showed completion of the reaction, the reaction mixture was diluted with water and extracted with EtOAc. The combined organic layers were washed with aqueous NaHCO_3_ solution followed by dilute aqueous HCl and finally with brine, and then dried over anhydrous Na_2_SO_4_. The solvent was evaporated under reduced pressure to obtain the crude material which was purified by Combi-flash using mixtures of EtOAc in hexanes as an eluent to afford **9** (20-80% yield) as a pale yellow solid.

Preparation of **9a**: 4-(2-Chloro-6-fluorobenzyl)-N-(furan-2-ylmethyl)-3-oxo-3,4-dihydro-2H-benzo[b][1,4]thiazine-6-carboxamide (G10)

Reaction time 2 h; yield 62%; white solid; purification by prep-HPLC; purity 99.09% by UPLC (Method B); ^1^H NMR: (500 MHz; DMSO-d_6_): δ 3.58 (s, 2H), 4.45 (d, *J* = 5.55 Hz, 2H), 5.38 (s, 2H), 6.26 (d, *J* = 2.5 Hz, 1H), 6.41 (t, *J* = 1.5 Hz, 1H), 7.10-7.13 (m, 1H), 7.23-7.31 (m, 2H), 7.47-7.51 (m, 2H), 7.59 (s, 1H), 7.79 (s, 1H), 8.94 (t, *J* = 5.6 Hz, 1H); UPLC-MS m/z: 431.05 [M+H]; HRMS (ESI) m/z: Calcd. for C_21_H_16_ClFN_2_O_3_S [M+H]^+^ = 431.0632, found 431.0625.

Preparation of **9b**: 4-(2,6-Dichlorobenzyl)-N-(furan-2-ylmethyl)-3-oxo-3,4-dihydro-2H-benzo[b][1,4]thiazine-6-carboxamide (Compound 1)

Reaction time 4 h; yield 60%; white solid; purification by prep-HPLC; purity 100% by LCMS and 99.63% by UPLC (Method B); ^1^H NMR: (400 MHz; DMSO-d_6_): δ 3.56 (s, 2H), 4.45 (d, *J* = 5.56 Hz, 2H), 5.42 (s, 2H), 6.26 (d, *J* = 2.68 Hz, 1H), 6.41 (dd, *J’* = 1.84 Hz, *J”* = 3.12 Hz, 1H), 7.22-7.26 (m, 1H), 7.36 (d, *J* = 7.84 Hz, 2H), 7.48 (s, 2H), 7.59 (t, *J* = 0.68 Hz, 1H), 7.77 (s, 1H), 8.92 (t, *J* = 5.64 Hz, 1H); ^13^C NMR: (100 MHz; DMSO-d_6_): δ 31.0, 35.9, 44.0, 106.8, 110.4, 117.6, 122.0, 128.1, 128.3, 128.6 (2xCH), 129.7, 131.4, 132.6, 135.1 (2xC), 138.7, 142.0, 152.1, 164.9, 165.7; LCMS m/z: 447 [M+H]; HRMS (ESI) m/z: Calcd. for C_21_H_16_Cl_2_N_2_O_3_S [M+H]^+^ = 447.0337, found 447.0329.Preparation of **9c**: 4-(2,6-Difluorobenzyl)-N-(furan-2-ylmethyl)-3-oxo-3,4-dihydro-2H-benzo[b][1,4]thiazine-6-carboxamide (Compound 2)

Reaction time 4 h; yield 65%; white solid; purification by prep-HPLC; purity 100% by LCMS and 99.66% by UPLC (Method B); ^1^H NMR: (400 MHz; DMSO-d_6_): δ 3.58 (s, 2H), 4.46 (d, *J* = 5.6 Hz, 2H), 5.36 (s, 2H), 6.25 (d, *J* = 2.44 Hz, 1H), 4.41 (dd, *J’* = 1.84 Hz, *J”* = 3.12 Hz, 1H), 6.97-7.02 (m, 2H), 7.29-7.33 (m, 1H), 7.46-7.59 (m, 2H), 7.59 (s, 1H), 7.83 (s, 1H), 8.96 (t, *J* = 5.52 Hz, 1H); ^13^C NMR: (100 MHz; DMSO-d_6_): δ 30.4, 36.0, 36.4, 106.7, 110.4, 111.5 (dd, *J’* = 5.92 Hz, *J”* = 18.76 Hz, 2xCH), 112.2 (t, *J* = 17.66 Hz, 1xC), 117.2, 121.9, 127.8, 128.0, 129.9 (t, *J* = 10.3 Hz, 1xCH), 132.6, 138.4, 142.0, 152.2, 159.5 (d, *J* = 7.8 Hz, 1xC), 162.0 (d, *J* = 7.86 Hz, 1xC), 164.8, 164.9; LCMS m/z: 415 [M+H]; HRMS (ESI) m/z: calcd. for C_21_H_16_F_2_N_2_O_3_S [M+H]^+^ = 415.0928, found 415.0918

Preparation of **9d**: 1-Benzyl-N-(furan-2-ylmethyl)-2-oxo-2,3-dihydro-1H-benzo[b][1,4]thiazine-7-carboxamide (Compound 3)

Reaction time 2 h; yield 20%; white solid; purification by prep-HPLC; purity 99.29% by UPLC (Method A); ^1^H NMR: (500 MHz; DMSO-d_6_): δ 3.72 (s, 2H), 4.42 (d, *J* = 5.5 Hz, 2H), 5.28 (s, 2H), 6.22 (d, *J* = 2.8 Hz, 1H), 6.39 (s, 1H), 7.20-7.24 (m, 3H), 7.29-7.32 (m, 2H), 7.51-7.54 (m, 2H), 7.57 (s, 1H), 7.65 (s, 1H), 8.99 (t, *J* = 5.45 Hz, 1H); UPLC-MS m/z: 379.18 [M+H]; HRMS (ESI) m/z: calcd. for C_21_H_18_N_2_O_3_S [M+H]^+^ = 379.1116, found 379.1107

Preparation of **9e**: 4-(2-Chloro-6-fluorobenzyl)-N-(2,4-difluorobenzyl)-3-oxo-3,4-dihydro-2H-benzo[b][1,4]thiazine-6-carboxamide (Compound 4)

Reaction time 3 h; yield 76.5%; white solid; purification by combi-flash; purity 98.54% by UPLC (Method A); ^1^H NMR: (500 MHz; DMSO-d_6_): δ 3.60 (s, 2H), 4.46 (d, *J* = 5.5 Hz, 2H), 5.39 (s, 2H), 7.08-7.14 (m, 2H), 7.24-7.32 (m, 3H), 7.35-7.40 (m, 1H), 7.49 (s, 2H), 7.78 (s, 1H), 9.00 (t, *J* = 5.65 Hz, 1H); ^13^C NMR: (100 MHz; DMSO-d_6_): δ 30.7 (2xCH_2_), 36.0, 103.6 (t, *J* = 25.85 Hz, 1xCH), 111.2 (d, *J* = 21.03 Hz, 1xCH), 114.7 (d, *J* = 22.47 Hz, 1xCH), 117.4, 121.7-122.4 (m, 1xC), 121.9, 125.6, 128.0, 128.2, 130.1 (d, *J* = 9.26 Hz, 1xCH), 130.6 (dd, *J’* = 6.24 Hz, *J”* = 9.15 Hz, 1xCH), 132.5, 134.0 (d, *J* = 4.62 Hz, 1xC), 138.4, 158.7 (d, *J* = 12.13 Hz, 1xC), 159.9-160.1 (m, 1xC), 161.1 (d, *J* = 12.27 Hz, 1xC), 162.4-162.6 (m, 1xC), 165.2, 165.3; UPLC-MS m/z: 477.35 [M+H]; HRMS (ESI) m/z: calcd. for C_23_H_16_ClF_3_N_2_O_2_S [M+H]^+^ = 477.0651, found 477.0644

Preparation of **9f**: 4-(2-Chloro-6-fluorobenzyl)-3-oxo-N-(2,4,6-trifluorobenzyl)-3,4-dihydro-2H-benzo[b][1,4]thiazine-6-carboxamide (Compound 5)

Reaction time 4 h; yield 57%; white solid; purification by prep-HPLC; purity 99.46% by UPLC (Method B); ^1^H NMR: (500 MHz; DMSO-d_6_): δ 3.58 (s, 2H), 4.45 (d, *J* = 4.9 Hz, 2H), 5.36 (s, 2H), 7.10 (t, *J* = 9.2 Hz, 1H), 7.20-7.31 (m, 4H), 7.42-7.47 (m, 2H), 7.73 (s, 1H), 8.85 (t, *J* = 5.1 Hz, 1H); ^13^C NMR: (100 MHz; DMSO-d_6_): δ 30.7 (2xCH_2_), 31.0, 100.3 (t, *J* = 27.68 Hz, 2xCH), 110.8 (t, *J* = 19.39 Hz,1xC), 114.43 (d, *J* = 22.81 Hz, 1xCH), 117.35, 121.7, 121.8, 121.9, 125.5, 127.9, 128.0, 130.0 (d, *J* = 9.68 Hz, 1xCH), 132.5, 134.0 (d, *J* = 4.74 Hz, 1xC), 138.3, 159.9-160.3 (m, 2xC), 162.3-162.6 (m, 1xC), 164.8, 165.3; UPLC-MS m/z: 495.24 [M+H]; HRMS (ESI) m/z: calcd. for C_23_H_15_ClF_4_N_2_O_2_S [M+H]^+^ = 495.0557, found 495.0547.

Preparation of **9g** *rac*: 4-(2-Chloro-6-fluorobenzyl)-2-methyl-3-oxo-N-(2,4,6-trifluorobenzyl)-3,4-dihydro-2H-benzo[b][1,4]thiazine-6-carboxamide (Compound 8)

Reaction time 3 h; yield 48%; white solid; purification by prep-HPLC; purity 99.38% by UPLC (Method B); ^1^H NMR: (500 MHz; DMSO-d_6_): δ 1.31 (d, *J* = 6.95 Hz, 3H), 3.72-3.76 (q, *J* = 6.35 Hz, 1H), 4.45 (d, *J* = 4.7 Hz, 2H), 5.29 (d, *J* = 15.7 Hz, 1H), 5.45 (d, *J* = 15.75 Hz, 1H), 7.10 (t, *J* = 9.5 Hz, 1H), 7.20-7.30 (m, 4H), 7.46 (s, 2H), 7.72 (s, 1H), 8.86 (bs, 1H); ^13^C NMR: (100 MHz; DMSO-d_6_): δ 14.8, 31.0, 37.2, 40.7, 100.4 (t, *J* = 27.79 Hz, 2xCH), 110.6-111.1 (m, 1xC), 114.5 (d, *J* = 22.84 Hz, 1xCH), 117.2, 121.8, 122.0, 122.1, 125.6, 126.6, 128.3, 130.0 (d, *J* = 10.16 Hz, 1xCH), 132.6, 134.0 (d, J = 4.64 Hz, 1xC), 137.9, 159.8-160.2 (m, 2xC), 162.3-162.6 (m, 1xC), 164.9, 167.3; UPLC-MS m/z: 509.32 [M+H]; HRMS (ESI) m/z: calcd. for C_24_H_17_ClF_4_N_2_O_2_S [M+H]^+^ = 509.0714, found 509.0704.

Preparation of **9h** *rac*: 4-(3,5-Difluorobenzyl)-2-methyl-3-oxo-N-(2,4,6-trifluorobenzyl)-3,4-dihydro-2H-benzo[b][1,4]thiazine-6-carboxamide (Compound 13)

Reaction time 3 h; yield 66%; white solid; purification by combi-flash; purity 96.54% by UPLC (Method B), 99.83% by UPLC (Method A); ^1^H NMR: (500 MHz; DMSO-d_6_): δ 1.39 (d, *J* = 6.95 Hz, 3H), 3.97 (d, J = 6.95 Hz, 1H), 4.42 (s, 2H), 5.20 (d, *J* = 17.05 Hz, 1H), 5.34 (d, *J* = 17.05 Hz, 1H), 6.89 (d, *J* = 6.7 Hz, 2H), 7.14-7.18 (m, 3H), 7.53 (s, 3H), 8.94 (t, *J* = 5.1 Hz, 1H); UPLC-MS m/z: 493.29 [M+H]; HRMS (ESI) m/z: calcd. for C_24_H_17_F_5_N_2_O_2_S [M+H]^+^ = 493.1009, found 493.1004.

**Chiral separation of 9h *rac***

Racemic 4-(3,5-difluorobenzyl)-2-methyl-3-oxo-N-(2,4,6-trifluorobenzyl)-3,4-dihydro-2H-benzo[b][1,4]thiazine-6-carboxamide (**9h** *rac*) (55 mg) was subjected to chiral separation to afford two enantiomers;

Enantiomer 1, **9i** (Compound 10) (25.0 mg, purity 100%, chiral purity 100% ee)

Enantiomer 2, **9j** (Compound 11) (22.0 mg, purity 99.02%, chiral purity 98.04% ee)

**Chiral Separation Methods**

Chiral separation was carried out using an Agilent HPLC (1200 series) under the following conditions:

**Column**               :    Chiralcel OD-H (4.6 x 250 mm), 5μm
**Mobile phase**    :    Hexane/EtOH/DEA : 80/20/0.1
**Flow rate**            :    1.0 ml/min
**Run time**      :    20 min
**Wave length**     :    254 nm
**Solubility**           :    MeOH

Preparation of **9i** enantiomer 1: 4-(3,5-Difluorobenzyl)-2-methyl-3-oxo-N-(2,4,6-trifluorobenzyl)-3,4-dihydro-2H-benzo[b][1,4]thiazine-6-carboxamide (Compound 10)

Yield 45.5%; white solid; purity 100% by HPLC (chiral separation method); ^1^H NMR: (500 MHz; DMSO-d_6_): δ 1.39 (d, *J* = 6.8 Hz, 3H), 3.98-3.94 (m, 1H), 4.42 (s, 2H), 5.35-5.19 (m, 2H), 6.89 (d, *J* = 6.9 Hz, 2H), 7.18-7.13 (m, 3H), 7.53 (s, 3H), 8.92 (s, 1H); ^13^C NMR: (100 MHz; DMSO-d_6_): δ 14.4, 31.1, 36.3, 46.9, 100.3 (t, *J* = 28.15 Hz, 2xCH), 102.5 (t, *J* = 25.64 Hz, 1xCH), 109.22 (dd, *J’* = 6.93 Hz, *J”* = 18.61 Hz, 2xCH), 110.6-111.0 (m, 1xC), 116.7, 122.2, 126.0, 128.1, 132.9, 138.5, 141.9 (t, *J* = 9.07 Hz, 1xC), 159.7-160.3 (m, 2xC), 161.2 (d, *J* = 13.09 Hz, 1xC), 162.2-163.0 (m, 1xC), 163.7 (d, *J* = 13.11 Hz, 1xC), 164.8, 167.3; LCMS m/z: 493.1 [M+H]; HRMS (ESI) m/z: calcd. for C_24_H_17_F_5_N_2_O_2_S [M+H]^+^ = 493.1009, found 493.1001.

Preparation of **9j** enantiomer 2: 4-(3,5-Difluorobenzyl)-2-methyl-3-oxo-N-(2,4,6-trifluorobenzyl)-3,4-dihydro-2H-benzo[b][1,4]thiazine-6-carboxamide (Compound 11)

Yield 40%; white solid; purity 99.02% by HPLC (chiral separation method); ^1^H NMR: (500 MHz; DMSO-d_6_): δ 1.39 (d, *J* = 6.8 Hz, 3H), 3.98-3.94 (m, 1H), 4.42 (s, 2H), 5.35-5.19 (m, 2H), 6.89 (d, *J* = 6.8 Hz, 2H), 7.18-7.13 (m, 3H), 7.53 (s, 3H), 8.92 (s, 1H); ^13^C NMR: (100 MHz; DMSO-d_6_): δ 14.5, 31.1, 36.3, 46.9, 100.3 (t, *J* = 27.86 Hz, 2xCH), 102.5 (t, *J* = 25.69 Hz, 1xCH), 109.21 (dd, *J’* = 6.63 Hz, *J”* = 18.89 Hz, 2xCH), 110.6-111.1 (m, 1xC), 116.8, 122.2, 126.1, 128.1, 132.9, 138.5, 141.9 (t, *J* = 8.85 Hz, 1xC), 159.8-160.3 (m, 2xC), 161.3 (d, *J* = 13.0 Hz, 1xC), 162.2-162.6 (m, 1xC), 163.7 (d, *J* = 13.1 Hz, 1xC), 164.9, 167.3; LCMS m/z: 493.1 [M+H]; HRMS (ESI) m/z: calcd. for C_24_H_17_F_5_N_2_O_2_S [M+H]^+^ = 493.1009, found 493.1000.

Preparation of **9k** *rac*: N-(Benzofuran-2-ylmethyl)-1-(3,5-difluorobenzyl)-3-methyl-2-oxo-2,3-dihydro-1H-pyrido[2,3-b][1,4]thiazine-7-carboxamide (Compound 12)

Reaction time 3 h; yield 21%; white solid; purification by prep-HPLC; purity 99.79% by UPLC (Method B); ^1^H NMR: (500 MHz; DMSO-d_6_): δ 1.46 (d, *J* = 7.0 Hz, 3H), 4.23-4.19 (m, 1H), 4.64 (d, *J* = 5.5 Hz, 2H), 5.36-5.19 (m, 2H), 6.75 (s, 1H), 6.94 (d, *J* = 6.6 Hz, 2H), 7.29-7.13 (m, 3H), 7.52 (d, *J* = 8.0 Hz, 1H), 7.58 (d, *J* = 7.5 Hz, 1H), 7.84 (s, 1H), 8.66 (d, *J* = 1.2 Hz, 1H), 9.33 (t, *J* = 5.5 Hz, 1H); ^13^C NMR: (100 MHz; DMSO-d_6_): δ 14.7, 35.9, 36.5, 46.8, 102.7 (t, *J* = 25.85 Hz, 1xC), 103.6, 109.3 (dd, *J’* = 6.79 Hz, *J”* = 18.87 Hz, 2xCH), 110.8, 120.7, 122.8, 123.2, 123.9, 127.6, 127.9, 134.9, 141.4 (t, *J* = 9.12 Hz, 1xC), 142.2, 148.8, 154.0, 155.1, 161.3 (d, *J* = 12.82 Hz, 1xC), 163.7, 163.8, 166.7; UPLC-MS m/z: 480.36 [M+H]; HRMS (ESI) m/z: calcd. for C_25_H_19_F_2_N_3_O_3_S [M+H]^+^ = 480.1193, found 480.1188.

Preparation of **9l**: 4-(2,6-Difluoro-4-methoxybenzyl)-3-oxo-N-(2,4,6-trifluorobenzyl)-3,4-dihydro-2H-benzo[b][1,4]thiazine-6-carboxamide (Compound 6)

Reaction time 1 h; yield 45%; white solid; purification by prep-HPLC; purity 99.53% by UPLC (Method A); ^1^H NMR: (500 MHz; DMSO-d_6_): δ 3.57 (s, 2H), 3.71 (s, 3H), 4.46 (d, *J* = 4.05 Hz, 2H), 5.26 (s, 2H), 6.61 (d, *J* = 9.85 Hz, 2H), 7.21 (t, *J* = 8.4 Hz, 2H), 7.45 (s, 2H), 7.78 (s, 1H), 8.85 (bs, 1H); ^13^C NMR: (100 MHz; DMSO-d_6_): δ 30.5, 31.1 (t, *J* = 2.95 Hz, 1xCH_2_), 35.5, 55.8, 98.0 (dd, *J’* = 7.69 Hz, *J”* = 21.32 Hz, 2xCH), 100.3 (t, *J* = 28.08 Hz, 2xCH), 103.8 (t, *J* = 18.87 Hz, 1xC), 110.6-111.1 (m, 1xC), 117.3, 121.9, 127.9, 127.9, 132.4, 138.1, 159.8-160.3 (m, 5xC), 162.3-162.8 (m, 1xC), 164.8, 164.8; UPLC-MS m/z: 509.16 [M+H]; HRMS (ESI) m/z: calcd. for C_24_H_17_F_5_N_2_O_3_S [M+H]^+^ = 509.0958, found 509.0951.

Preparation of **9m** *rac*: 4-(2-Chloro-6-fluoro-3-methoxybenzyl)-2-methyl-3-oxo-N-(2,4,6-trifluorobenzyl)-3,4-dihydro-2H-benzo[b][1,4]thiazine-6-carboxamide (Compound 14)

Reaction time 1 h; yield 80%; white solid; purification by trituration with diethyl ether and n-pentane; purity 94.52% by UPLC (Method A), 98.6% by UPLC (Method B); ^1^H NMR: (400 MHz; DMSO-d_6_): δ 1.31 (d, *J* = 7.04 Hz, 3H), 3.72 (d, *J* = 7.04 Hz, 1H), 3.78 (s, 3H), 4.44 (d, *J* = 4.96 Hz, 2H), 5.29 (d, *J* = 15.72 Hz, 1H), 5.42 (d, *J* = 15.72 Hz, 1H), 7.02-7.06 (m, 2H), 7.17-7.21 (m, 2H), 7.46 (s, 2H), 7.70 (s, 1H), 8.84 (d, *J* = 5.04 Hz, 1H); UPLC-MS m/z: 539.15 [M+H]; HRMS (ESI) m/z: calcd. for C_25_H_19_ClF_4_N_2_O_3_S [M+H]^+^ = 539.0819, found 539.0811.

**General procedure for the preparation of 10**

To a stirred solution of **9** (1.0 eq.) in anhydrous DCM (15 mL/mmol) was added BBr_3_ (2.5-5 eq., 1M sol. In DCM) under a nitrogen atmosphere at 0-5 °C, and then the reaction was stirred at RT for 2-4 h. Progress of the reaction was monitored by TLC and LCMS and after completion the reaction mixture was quenched over ice cold water and the pH of the reaction was adjusted to basic using a saturated solution of NaHCO_3_ and then extracted with DCM. The organic layer was separated, washed with brine, dried over anhydrous Na_2_SO_4_ and distilled *in* *vacuo* to give a crude product which was purified by prep-HPLC to afford **10** (40-42%) as a white solid.

Preparation of **10a**: 4-(2,6-Difluoro-4-hydroxybenzyl)-3-oxo-N-(2,4,6-trifluorobenzyl)-3,4-dihydro-2H-benzo[b][1,4]thiazine-6-carboxamide (Compound 7)

Reaction time 4 h; BBr_3_ (5 eq.); yield 41%; white solid; purification by prep-HPLC; purity 98.53% by UPLC (Method B); ^1^H NMR: (500 MHz; DMSO-d_6_): δ 3.56 (s, 2H), 4.46 (d, *J* = 4.3 Hz, 2H), 5.22 (s, 2H), 6.31 (d, *J* = 9.75 Hz, 2H), 7.20 (t, *J* = 8.4 Hz, 2H), 7.44 (s, 2H), 7.76 (s, 1H), 8.84 (bs, 1H), 10.32 (s, 1H); ^13^C NMR: (100 MHz; DMSO-d_6_): δ 30.5, 31.0, 35.4, 99.0 (dd, *J’* = 7.25 Hz, *J”* = 20.03 Hz, 2xCH), 100.0-100.6 (m, 2xCH), 101.9 (t, *J* = 19.24 Hz, 1xC), 110.6-111.1 (m, 1xC), 117.4, 121.8, 127.8, 127.9, 132.4, 138.1, 158.6 (t, *J* = 15.31 Hz, 1xC), 159.8-160.3 (m, 4xC), 162.3-162.8 (m, 1xC), 164.7, 164.9; UPLC-MS m/z: 495.16 [M+H]; HRMS (ESI) m/z: calcd for C_23_H_15_F_5_N_2_O_3_S [M+H]^+^ = 495.0802, found 495.0792.

Preparation of **10b *rac***: 4-(2-Chloro-6-fluoro-3-hydroxybenzyl)-2-methyl-3-oxo-N-(2,4,6-trifluorobenzyl)-3,4-dihydro-2H-benzo[b][1,4]thiazine-6-carboxamide (Compound 9)

Reaction time 2 h; BBr_3_ (2.5 eq.); yield 41.5%; white solid; purification by prep-HPLC; purity 98.94% by UPLC (Method A); ^1^H NMR: (500 MHz; DMSO-d_6_): δ 1.31 (d, *J* = 6.85 Hz, 3H), 3.72 (d, *J* = 6.9 Hz, 1H), 4.44 (d, *J* = 4.25 Hz, 2H), 5.26 (d, *J* = 15.65 Hz, 1H), 5.40 (d, *J* = 15.60 Hz, 1H), 6.81-6.92 (m, 2H), 7.18-7.21 (m, 2H), 7.46 (s, 2H), 7.69 (s, 1H), 8.38 (s, 1H), 10.09 (s, 1H);^13^C NMR: (100 MHz; DMSO-d_6_): δ 14.9, 31.0, 37.3, 41.0, 100.4 (t, *J* = 27.4 Hz, 2xCH), 110.7 (d, *J* = 18.9 Hz, 1xC), 114.1 (d, *J* = 24.2 Hz, 1xCH), 115.4 (d, *J* = 8.9 Hz, 1xCH), 117.2, 120.1, 122.0, 122.1, 126.5, 128.3, 132.6, 137.9, 149.7, 152.7, 155.1, 156.0, 162.4, 164.9, 167.2; UPLC-MS m/z: 525.13 [M+H]; HRMS (ESI) m/z: calcd. for C_24_H_17_ClF_4_N_2_O_3_S [M+H]^+^ = 525.0663, found 525.0653.
