# Supplementary material for: G10 is a direct activator of human STING
Source: PLoS One. 2020 Sep 10;15(9):e0237743. doi: 10.1371/journal.pone.0237743 (PMC7482845; doi:10.1371/journal.pone.0237743)
Supplement: S2 File — Chemical characterisation spectra for Compounds 1–12. (PDF) [file pone.0237743.s014.pdf]

NP-CA150-98-A2

Compound G10 (9a)

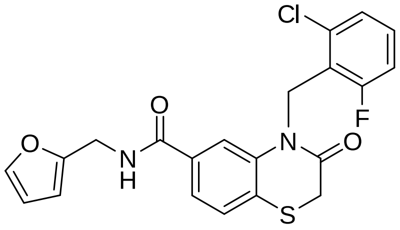

Compound G10 (9a)

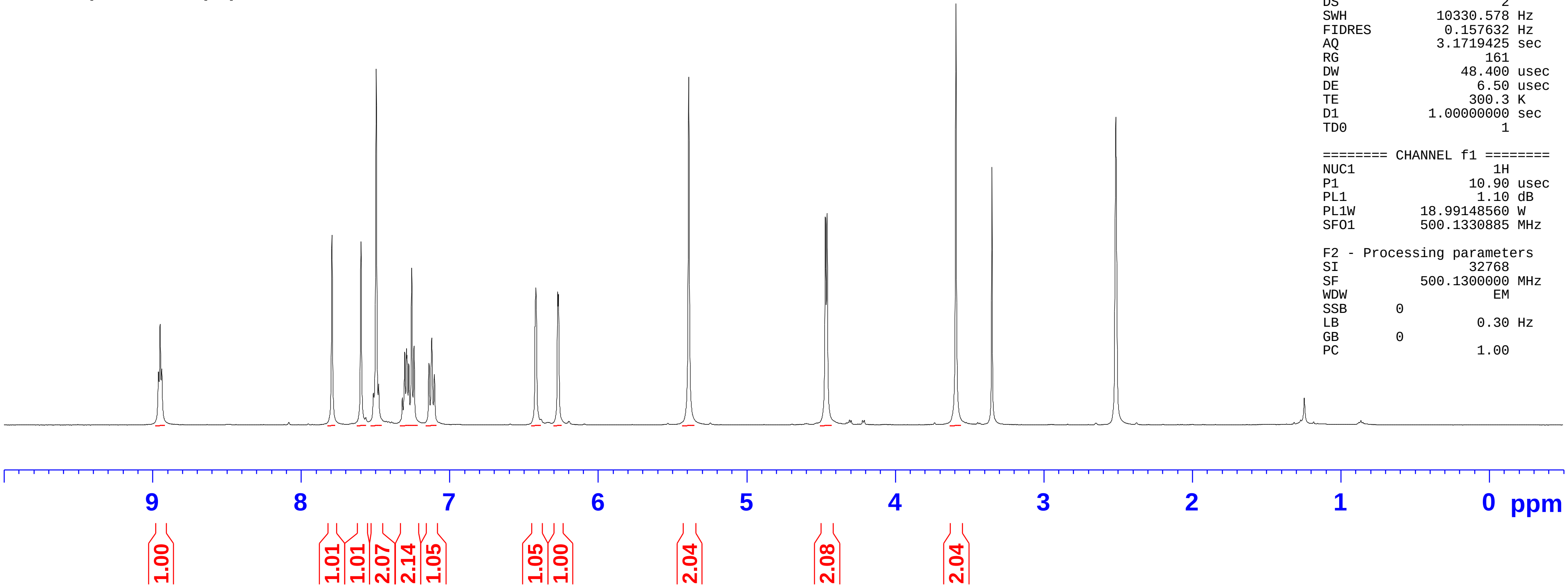

Current Data Parameters
NAME 113-132
EXPNO 123
PROCNO 1

F2 - Acquisition Parameters
Date_ 20160701
Time 3.45
INSTRUM spect
PROBHD 5 mm PABBO BB-
PULPROG zg30
TD 65536
SOLVENT DMSO
NS 12
DS 2
SWH 10330.578 Hz
FIDRES 0.157632 Hz
AQ 3.1719425 sec
RG 161
DW 48.400 usec
DE 6.50 usec
TE 300.3 K
D1 1.00000000 sec
TD0 1

======== CHANNEL f1 ========
NUC1 1H
P1 10.90 usec
PL1 1.10 dB
PL1W 18.99148560 W
SFO1 500.1330885 MHz

F2 - Processing parameters
SI 32768
SF 500.1300000 MHz
WDW EM
SSB 0
LB 0.30 Hz
GB 0
PC 1.00

Compound G10 (9a)

SAMPLE INFORMATION

Sample Name:	NP_CA150_98_A2	Acquired By:	UPLC_MS_01 System
Vial:	1:C,5	Sample Set Name:	CRD_FINAL
Injection #:	1	Acq. Method Set:	UPLC_FA_C18_6min_N
Injection Volume:	0.40 ul	Processing Method:	MASS
Run Time:	6.0 Minutes	Channel Name:	431.0Da
Date Acquired:	28-06-2016 18:01:17 IST	Date Processed:	30-06-2016 16:08:46 IST

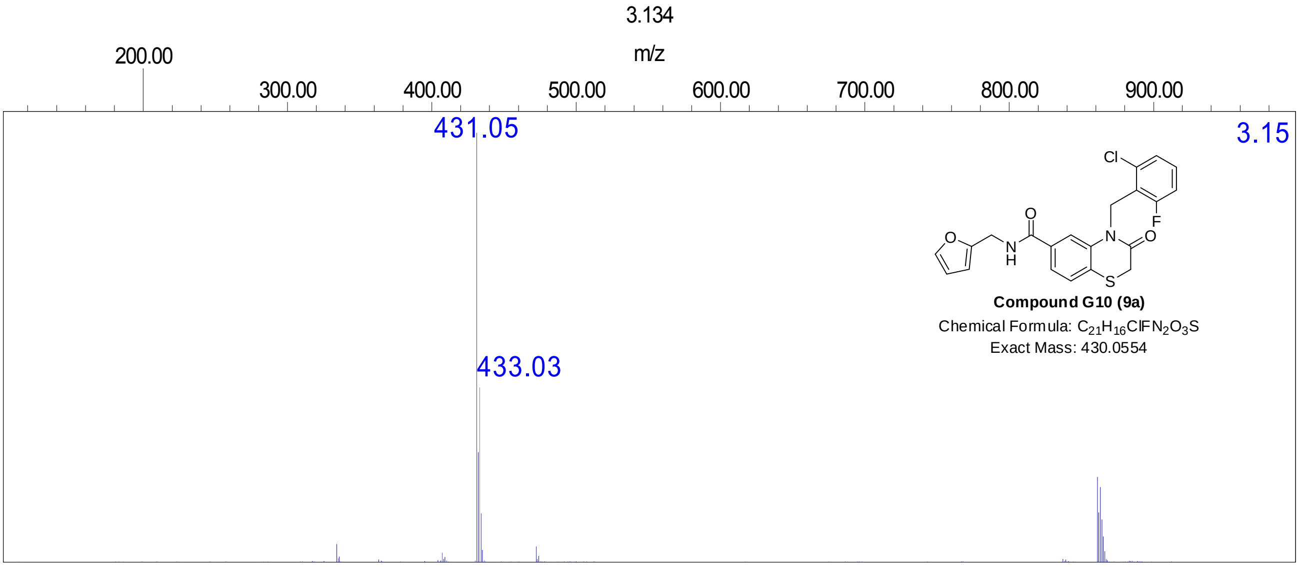

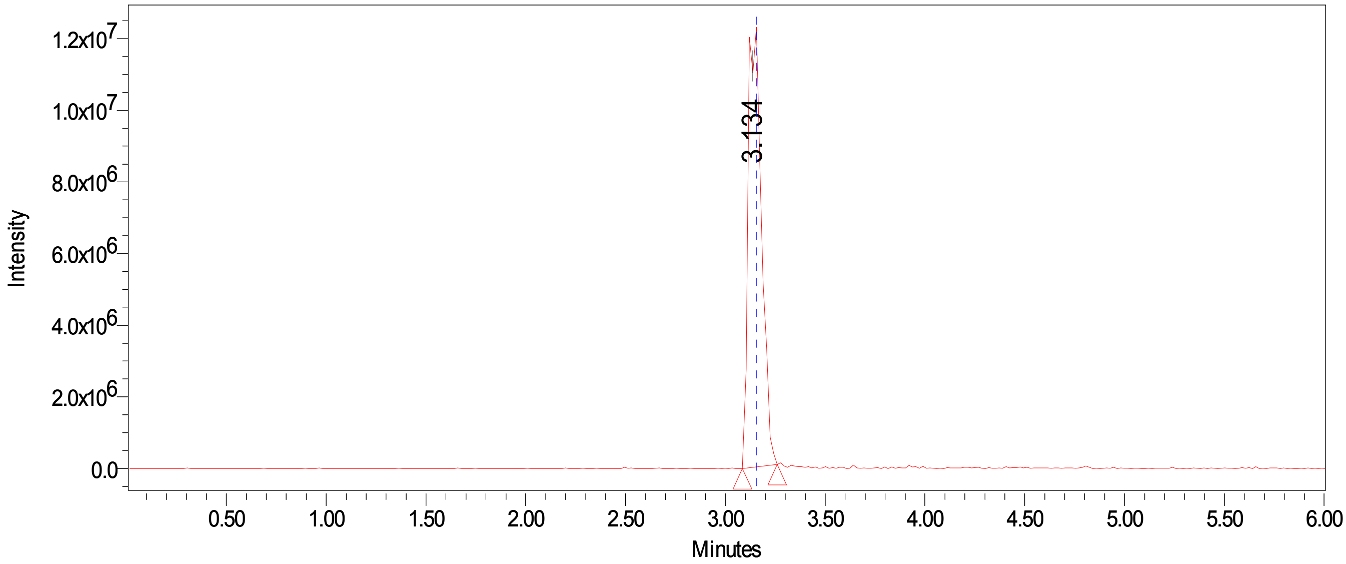

Channel Description 1: 100.00-1000.00 ES+, Centroid, CV=Tune; Processed Channel Descr. W3100 1: MS Scan MS 431.00 m/z Peak Separation: 1.0000 (1: 100.00-1000.00 ES+, Centroid, CV=Tune)

Qualitative Analysis Report

Compound G10 (9a)

**Data Filename**	AS-CRD-2070.d	**Sample Name**	AS-CRD-2070
**Sample Type**	Sample	**Position**	Vial 33
**Instrument Name**	Instrument 1	**User Name**	
**Acq Method**	Direct Mass-2017.m	**Acquired Time**	6/30/2020 9:24:55 PM
**IRM Calibration Status**	Some Ions Missed	**DA Method**	Default.m
**Comment**			

Sample Group **Info.**

Acquisition SW Version 6200 series TOF/6500 series Q-TOF B.05.00 (B5042.0)

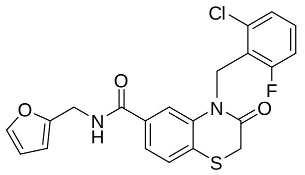

Compound G10 (9a)
Chemical Formula: $C_{21}H_{16}ClFN_2O_3S$
Exact Mass: 430.0554

User Chromatograms

Fragmentor Voltage 118 **Collision Energy** 0 **Ionization Mode** ESI

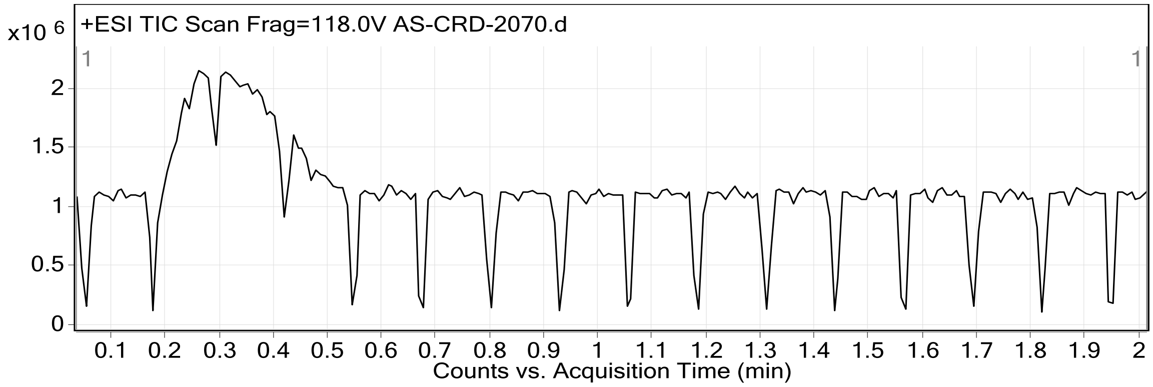

User Spectra

**Fragmentor Voltage**	**Collision Energy**	**Ionization Mode**
118	0	ESI

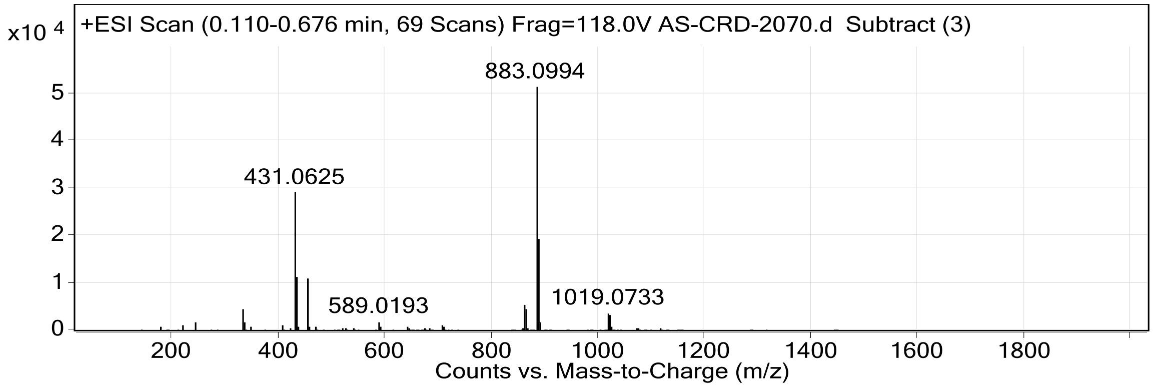

Peak List

m/z	z	Abund
431.0625	1	29191.83
432.0657	1	7183.13
433.0603	1	11390.19
453.0444	1	11051.55
861.1184	1	5594.88
883.0994	1	51582.91

Qualitative Analysis Report

884.1024	1	24595.97
885.0978	1	43934.43
886.0998	1	19285.84
887.0966	1	12729.88

Compound G10 (9a)

Compounds

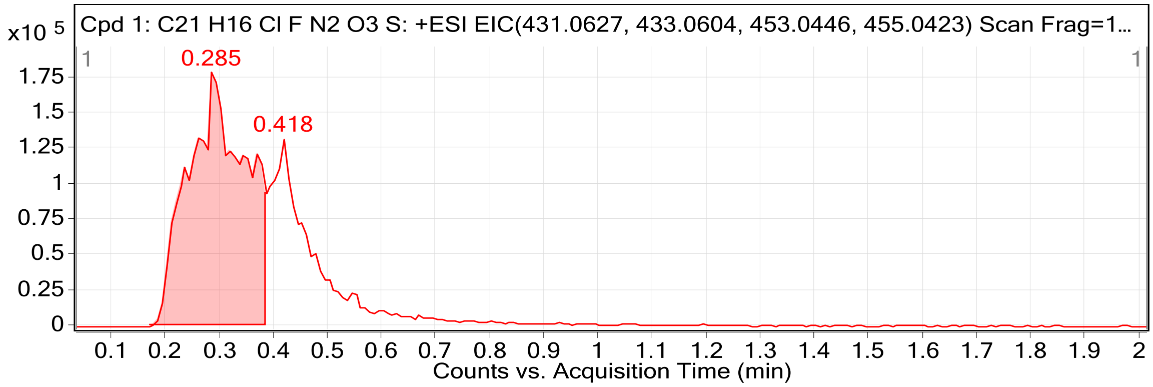

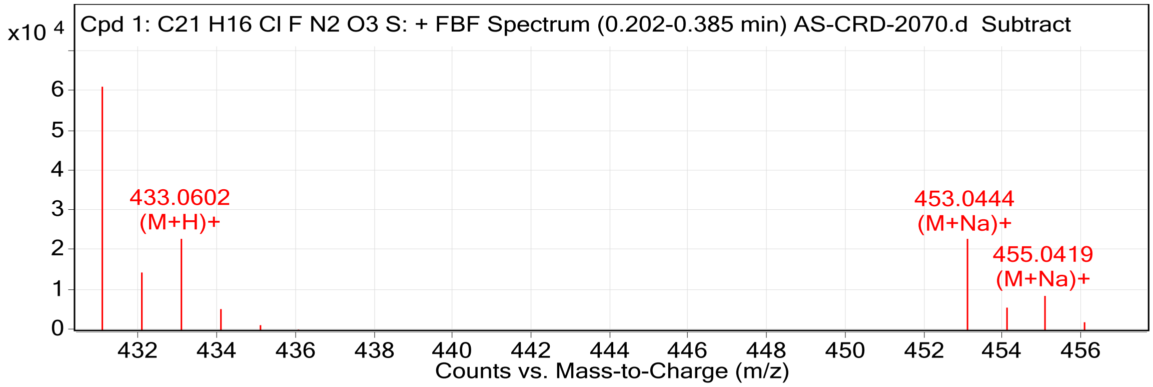

Peak List

m/z	z	Abund	Formula	Ion
431.0625	1	61318.65	C21H17ClFN2O3S	(M+H)+
432.0656	1	14634.2	C21H17ClFN2O3S	(M+H)+
433.0602	1	23251.8	C21H17ClFN2O3S	(M+H)+
434.0625	1	5441.59	C21H17ClFN2O3S	(M+H)+
435.0604	1	1580.29	C21H17ClFN2O3S	(M+H)+
436.0613	1	339.73	C21H17ClFN2O3S	(M+H)+
453.0444	1	22937.07	C21H16ClFN2NaO3S	(M+Na)+
454.0475	1	5791.19	C21H16ClFN2NaO3S	(M+Na)+
455.0419	1	8946.24	C21H16ClFN2NaO3S	(M+Na)+
456.0445	1	2092.86	C21H16ClFN2NaO3S	(M+Na)+

Compound G10 (9a)

SAMPLE INFORMATION

Sample Name:	NP_CA150_98_A2	Acquired By:	UPLC_MS_01 System
Vial:	1:C,5	Sample Set Name:	CRD_FINAL
Injection #:	1	Acq. Method Set:	UPLC_FA_C18_6min_N
Injection Volume:	0.40 ul	Processing Method:	UPLC
Run Time:	6.0 Minutes	Channel Name:	240.0nm
Date Acquired:	28-06-2016 18:01:17 IST	Date Processed:	30-06-2016 16:08:11 IST
Column	KINETEX EVO C18 (2.1x100mm)	Mobile Phase	0.1% Formic Acid in Water/ACN

Auto-Scaled Chromatogram

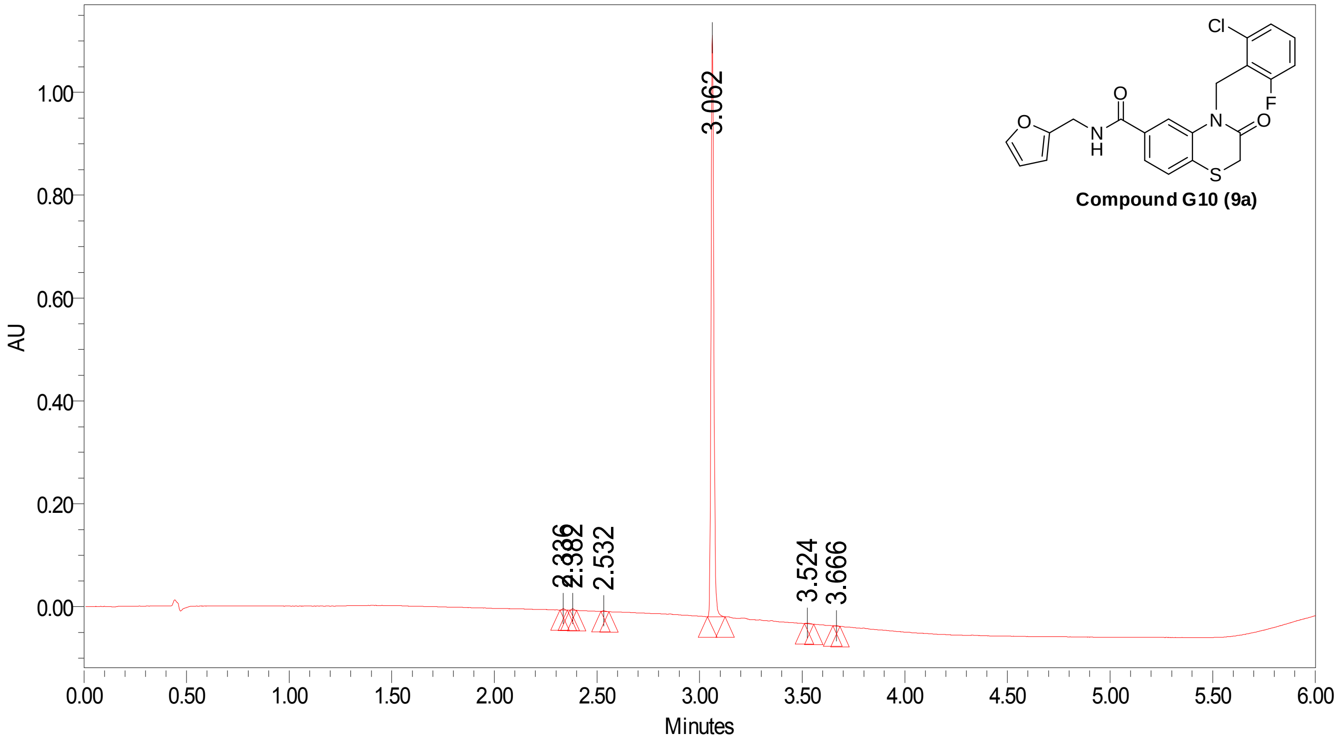

Processed Channel Descr. PDA 240.0 nm (PDA Spectrum (210-400)nm)

Peak Results

	Name	RT	Area	% Area	Height
1		2.336	2483	0.23	2948
2		2.382	2807	0.26	3312
3		2.532	1553	0.14	1600
4		3.062	1064838	99.09	1132739
5		3.524	2044	0.19	1477
6		3.666	941	0.09	947

GS-CA283-56

Compound 1 (9b)

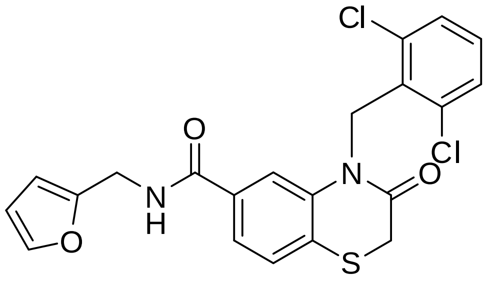

Chemical Formula: $C_{21}H_{16}Cl_2N_2O_3S$

Exact Mass: 446.03

Molecular Weight: 447.33

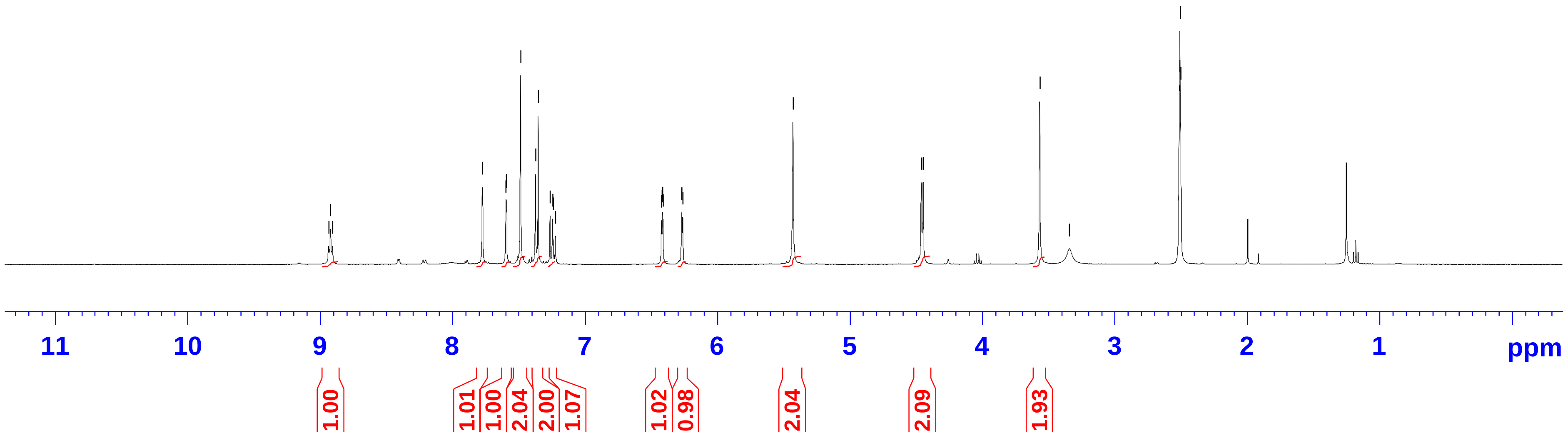


```
Current Data Parameters
NAME          10-06-2019
EXPNO                 80
PROCNO                 1

F2 - Acquisition Parameters
Date_           20190610
Time               14.47
INSTRUM            spect
PROBHD   5 mm PABBO BB/
PULPROG             zg30
TD                 65536
SOLVENT             DMSO
NS                    16
DS                     2
SWH             8012.820 Hz
FIDRES          0.122266 Hz
AQ             4.0894465 sec
RG                200.06
DW                62.400 usec
DE                  6.50 usec
TE                 296.4 K
D1            1.00000000 sec
TD0                    1

======== CHANNEL f1 ========
SFO1         400.2464717 MHz
NUC1                  1H
P1                 12.85 usec
PLW1         15.00000000 W

F2 - Processing parameters
SI                 65536
SF           400.2440000 MHz
WDW                   EM
SSB      0
LB                  0.30 Hz
GB       0
PC                  1.00
```

Compound 1 (9b)

TCG Lifesciences Private Limited
Kolkata

CRD2926B2 IN DMSO-13C

TCGLS/ARD/NMR02/K02

NAME	CRD2926B2
EXPNO	60
PROCNO	1
Date_	20200704
Time	17.07
INSTRUM	spect
PROBHD	5 mm PABBO BB-
PULPROG	zgpg30
TD	65536
SOLVENT	DMSO
NS	20000
DS	4
SWH	25252.525 Hz
FIDRES	0.385323 Hz
AQ	1.2976629 sec
RG	2050
DW	19.800 usec
DE	6.50 usec
TE	298.4 K
D1	2.00000000 sec
D11	0.03000000 sec
TD0	1

======== CHANNEL f1 ========

NUC1	13C
P1	6.75 usec
PL1	0.00 dB
SFO1	100.6404331 MHz

======== CHANNEL f2 ========

CPDPRG2	waltz16
NUC2	1H
PCPD2	80.00 usec
PL2	0.00 dB
PL12	13.45 dB
PL13	17.00 dB
SFO2	400.2016008 MHz
SI	32768
SF	100.6304292 MHz
WDW	EM
SSB	0
LB	1.00 Hz
GB	0
PC	1.40

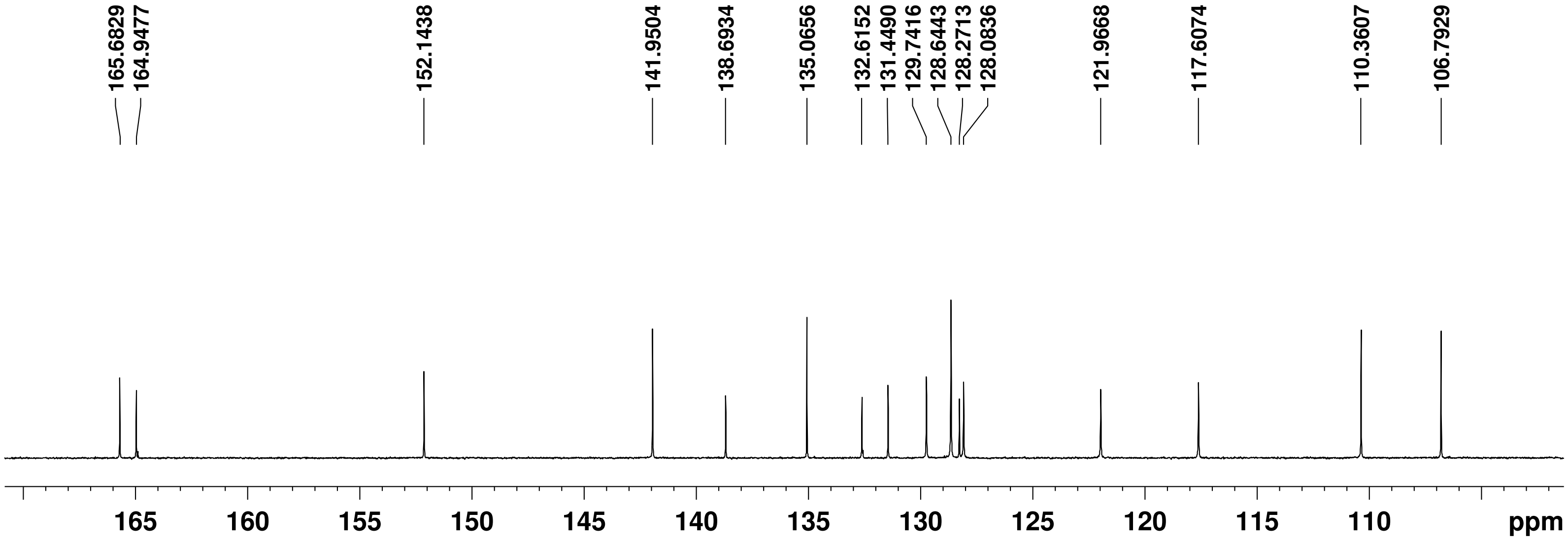

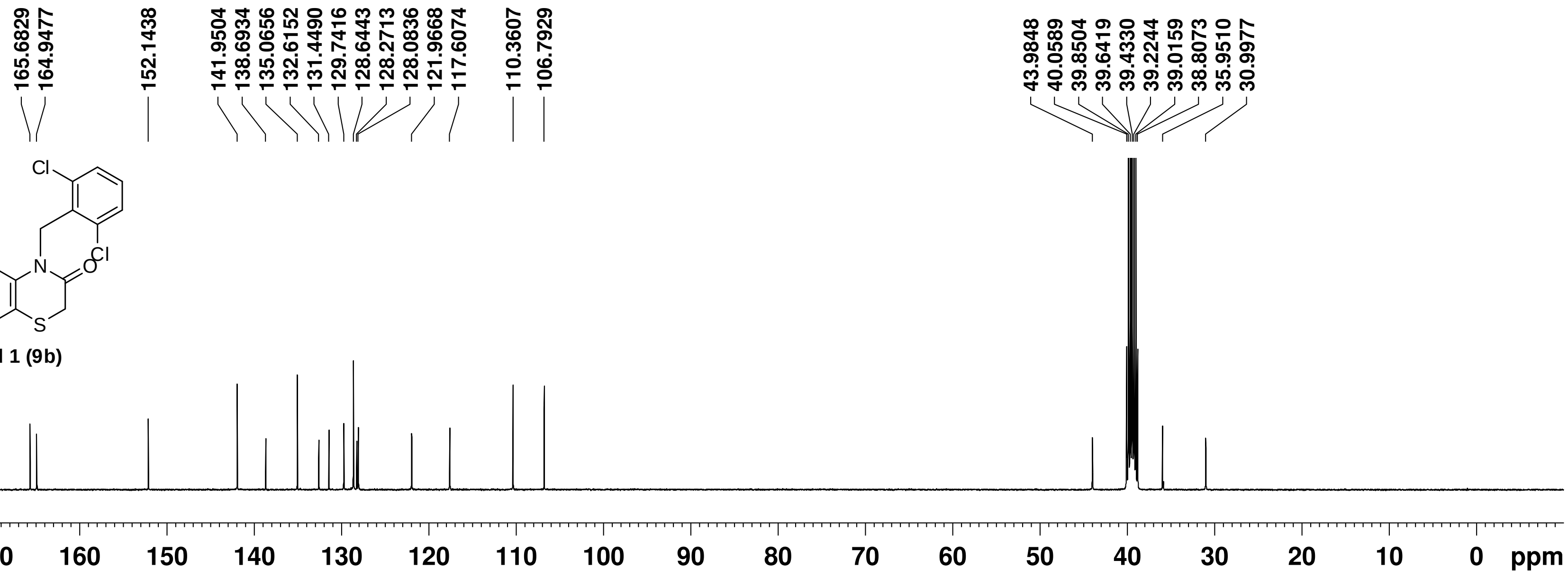

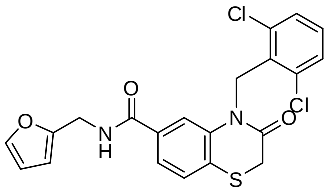

Compound 1 (9b)

Compound 1 (9b)

TCG Lifesciences Private Limited
Kolkata

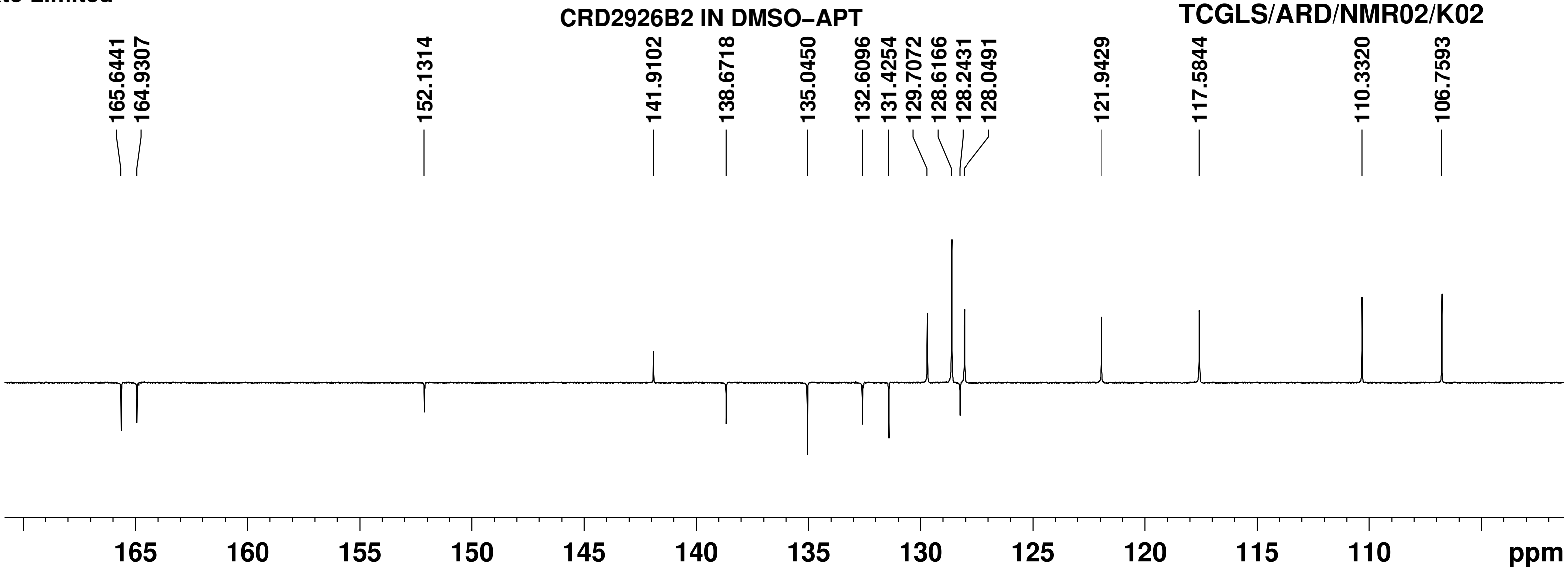

NAME	CRD2926B2
EXPNO	61
PROCNO	1
Date_	20200705
Time	20.58
INSTRUM	spect
PROBHD	5 mm PABBO BB–
PULPROG	jmod
TD	65536
SOLVENT	DMSO
NS	10000
DS	4
SWH	25252.525 Hz
FIDRES	0.385323 Hz
AQ	1.2976629 sec
RG	2050
DW	19.800 usec
DE	6.50 usec
TE	300.6 K
CNST2	145.0000000
CNST11	1.0000000
D1	2.00000000 sec
D20	0.00689655 sec
TD0	1
======== CHANNEL f1 ========	
NUC1	13C
P1	6.75 usec
P2	13.50 usec
PL1	0.00 dB
SFO1	100.6404331 MHz
======== CHANNEL f2 ========	
CPDPRG2	waltz16
NUC2	1H
PCPD2	80.00 usec
PL2	0.00 dB
PL12	13.45 dB
SFO2	400.2016008 MHz
SI	32768
SF	100.6304317 MHz
WDW	EM
SSB	0
LB	1.00 Hz
GB	0
PC	1.40

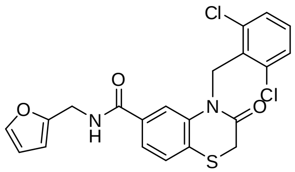

Compound 1 (9b)

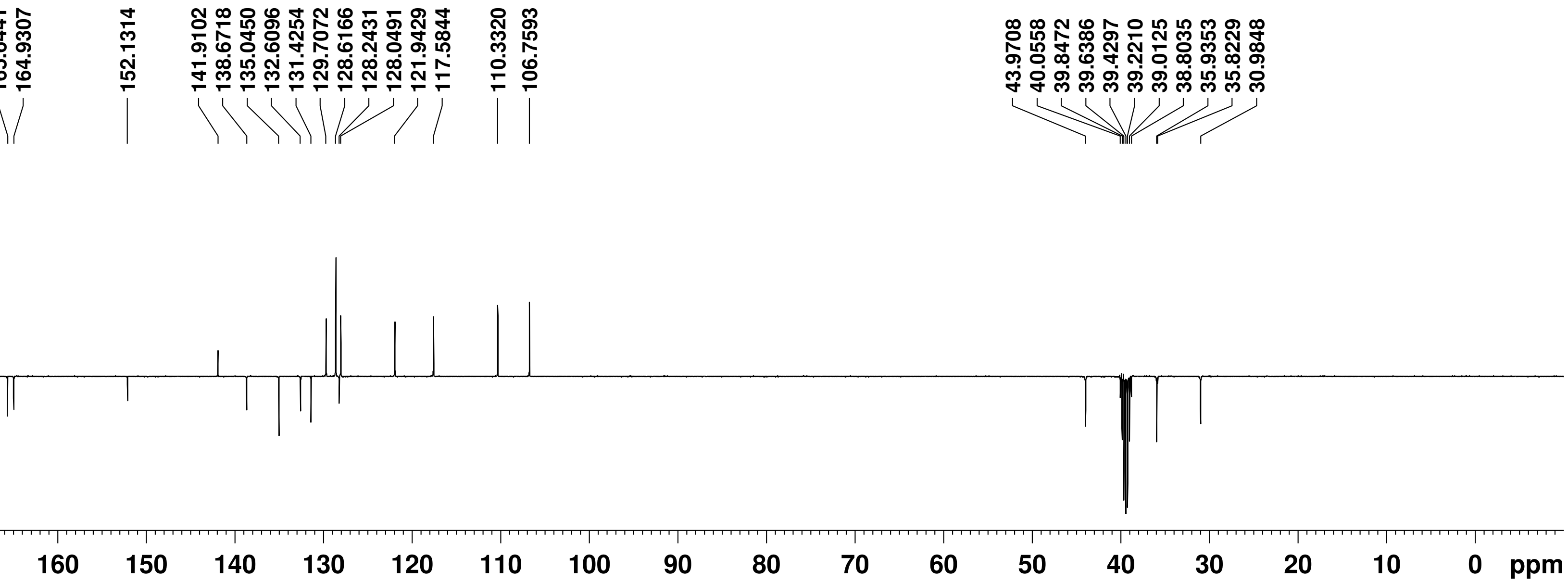

Qualitative Analysis Report

Compound 1 (9b)

**Data Filename**	AS-CRD-2926.d	**Sample Name**	AS-CRD-2926
**Sample Type**	Sample	**Position**	Vial 61
**Instrument Name**	Instrument 1	**User Name**	
**Acq Method**	Direct Mass-2017.m	**Acquired Time**	6/16/2020 11:59:08 AM
**IRM Calibration Status**	Some Ions Missed	**DA Method**	Default.m
**Comment**			

Sample Group **Info.**

Acquisition SW Version 6200 series TOF/6500 series Q-TOF B.05.00 (B5042.0)

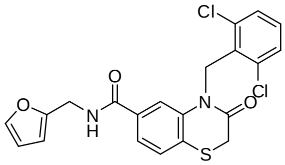

Compound 1 (9b)
Chemical Formula: $C_{21}H_{16}Cl_2N_2O_3S$
Exact Mass: 446.0259

User Chromatograms

Fragmentor Voltage 118 **Collision Energy** 0 **Ionization Mode** ESI

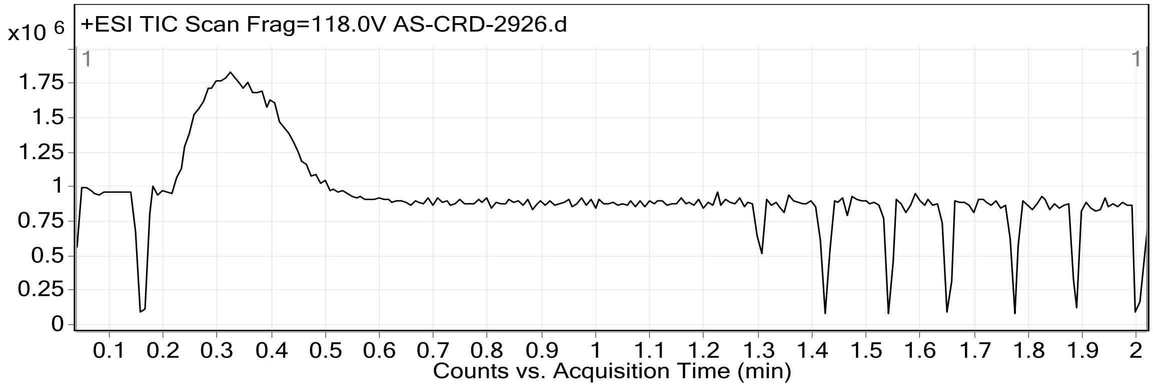

User Spectra

**Fragmentor Voltage**	**Collision Energy**	**Ionization Mode**
118	0	ESI

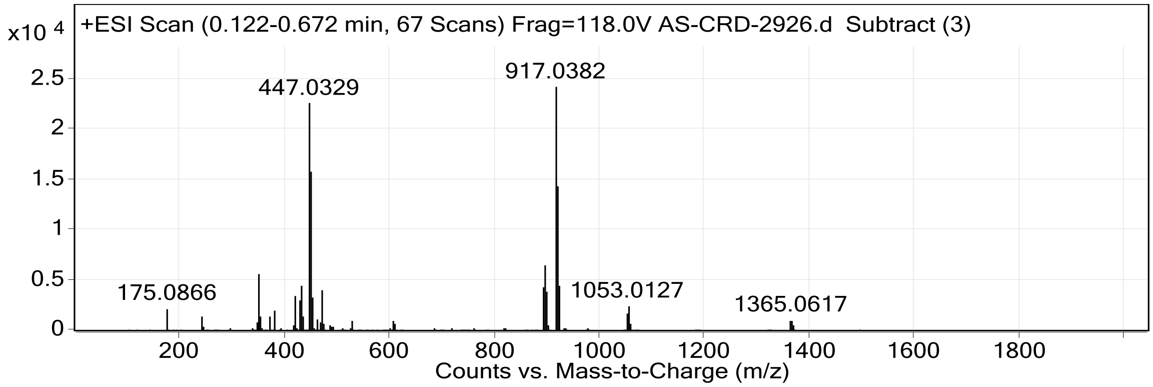

Peak List

m/z	z	Abund
348.1341	1	5626.17
447.0329	1	22615.31
449.0302	1	15801.02
895.0562	1	6505.04
915.0405	1	15727.88
916.0435	1	7853.19

917.0382	1	24260.55
918.0405	1	11010.44
919.0362	1	14438
920.038	1	6245.78

Compound 1 (9b)

Compounds

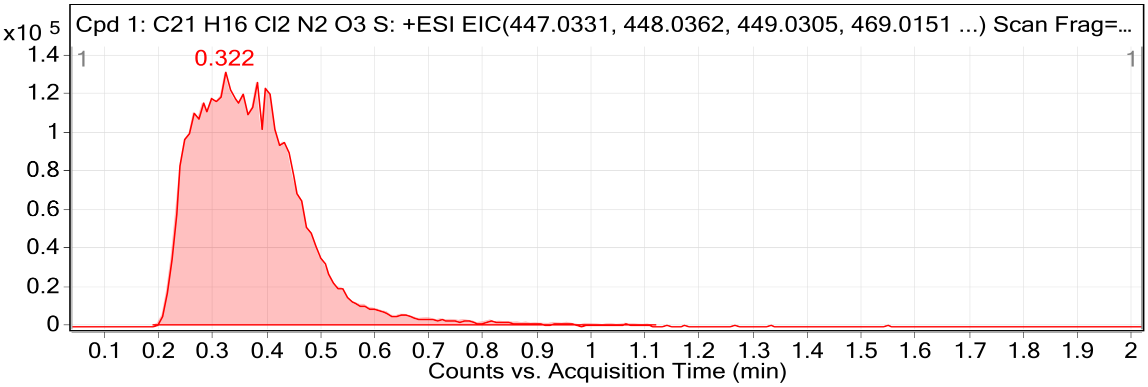

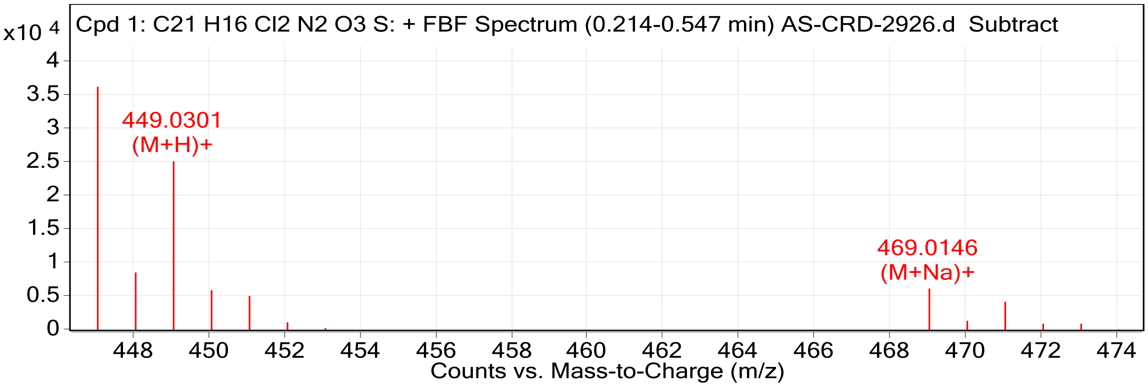

Peak List

m/z	z	Abund	Formula	Ion
447.0329	1	36405.9	C21H17Cl2N2O3S	(M+H)+
448.0358	1	8672.74	C21H17Cl2N2O3S	(M+H)+
449.0301	1	25345.13	C21H17Cl2N2O3S	(M+H)+
450.0331	1	6087.02	C21H17Cl2N2O3S	(M+H)+
451.0278	1	5250.98	C21H17Cl2N2O3S	(M+H)+
452.0299	1	1214.7	C21H17Cl2N2O3S	(M+H)+
469.0146	1	6292.84	C21H16Cl2N2NaO3S	(M+Na)+
470.0178	1	1565.95	C21H16Cl2N2NaO3S	(M+Na)+
471.0122	1	4458.86	C21H16Cl2N2NaO3S	(M+Na)+
472.0152	1	1154.67	C21H16Cl2N2NaO3S	(M+Na)+

Compound 1 (9b)

Batch No.: **CR240FFSL-Lib2-1-16-NEW-1**

CHEMBIOTEK TCG LIFESCIENCES ENTERPRISE
KOLKATA, INDIA

Production_ID:

INSTRUMENT No.:LCMS07/K09

09-Feb-201712:56:30

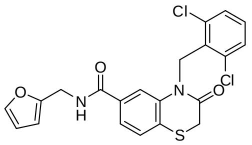

Compound 1 (9b)
Chemical Formula: $C_{21}H_{16}Cl_2N_2O_3S$
Exact Mass: 446.0259

09FEB17_RES_FINAL_FA_49_A

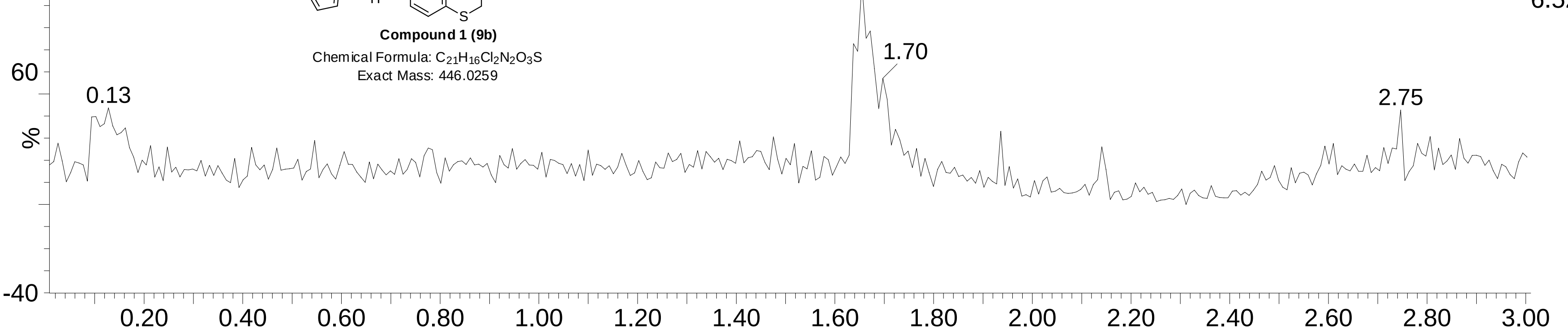

09FEB17_RES_FINAL_FA_49_A

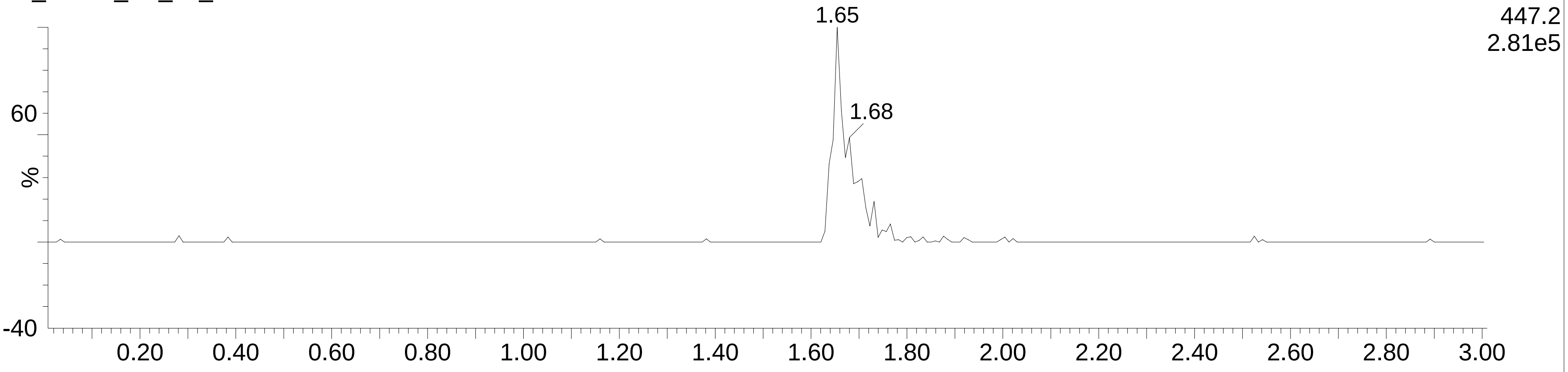

09FEB17_RES_FINAL_FA_49_A

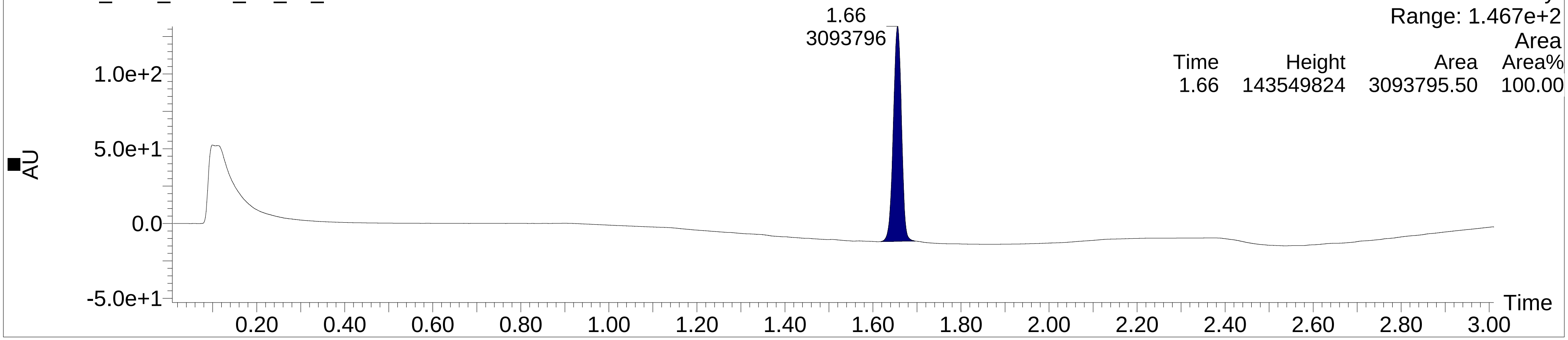

Compound 1 (9b)

Batch No.:
CR240FFSL-Lib2-1-16-NEW-1

CHEMBIOTEK TCG LIFESCIENCES ENTERPRISE
KOLKATA, INDIA

Production_ID:

INSTRUMENT No.:LCMS07/K09

09-Feb-201712:56:30

09FEB17_RES_FINAL_FA_49_A 194 (1.655)

1: Scan ES+
9.36e4

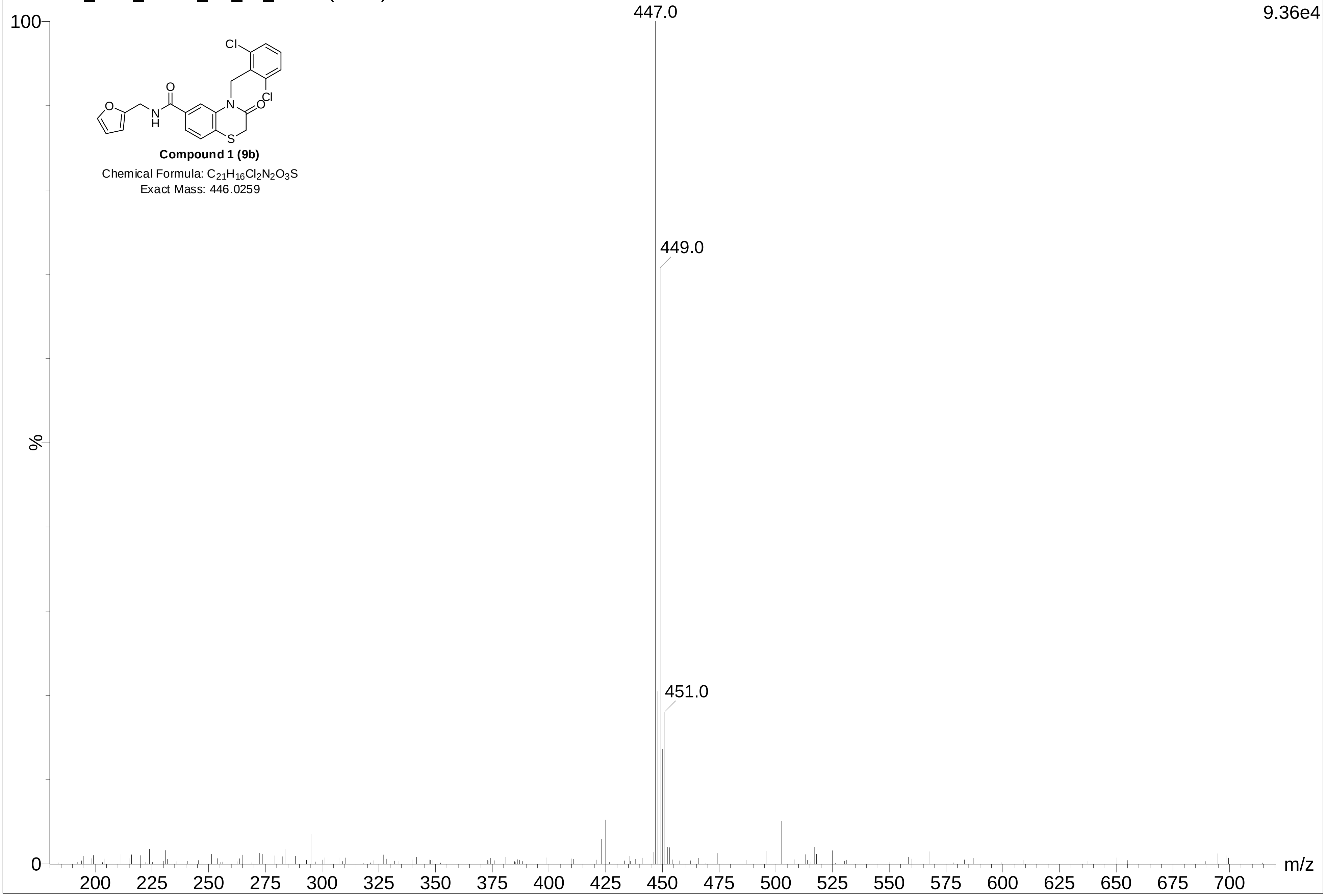

Compound 1 (9b)

SAMPLE INFORMATION

Sample Name:	GS-CA327-06	Acquired By:	UPLC_MS_01 System
Vial:	1:E,2	Sample Set Name:	SAMPLE_FA
Injection #:	1	Acq. Method Set:	FA_C18_6min_N
Injection Volume:	1.20 ul	Processing Method:	UPLC1
Run Time:	6.0 Minutes	Channel Name:	220.0nm@2
Date Acquired:	11-06-2020 15:57:19 IST	Date Processed:	11-06-2020 17:25:14 IST
Column	KINETEX_EVO_C-18	Mobile Phase	0.1% FA in Water/ACN

Auto-Scaled Chromatogram

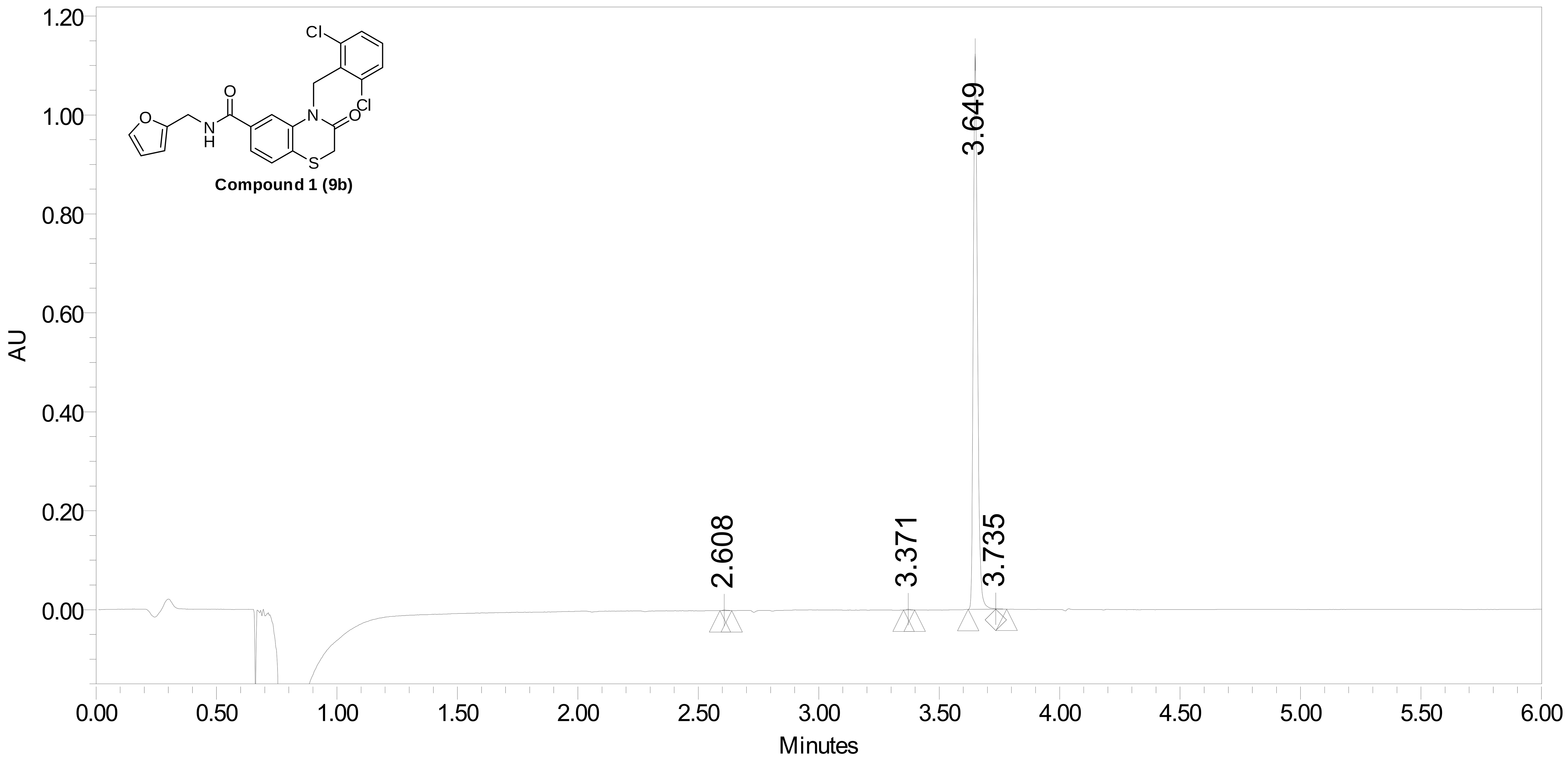

Processed Channel Descr. PDA 220.0 nm (PDA Spectrum (210-400)nm) Blank Subtracted from BLANK_DMSO, Vial 1:F,7 Inj. 1

Peak Results

	Name	RT	Area	% Area	Height
1		2.608	1860	0.14	1339
2		3.371	1882	0.14	1787
3		3.649	1364993	99.63	1122776
4		3.735	1278	0.09	1108

VK/CA287/07

Compound 2 (9c)

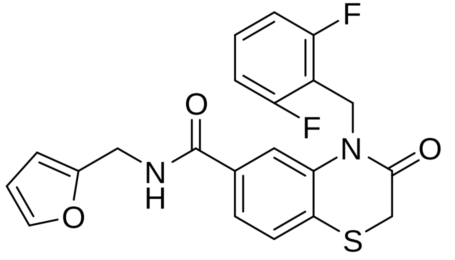

Chemical Formula: $C_{21}H_{16}F_2N_2O_3S$

Exact Mass: 414.08

Molecular Weight: 414.43

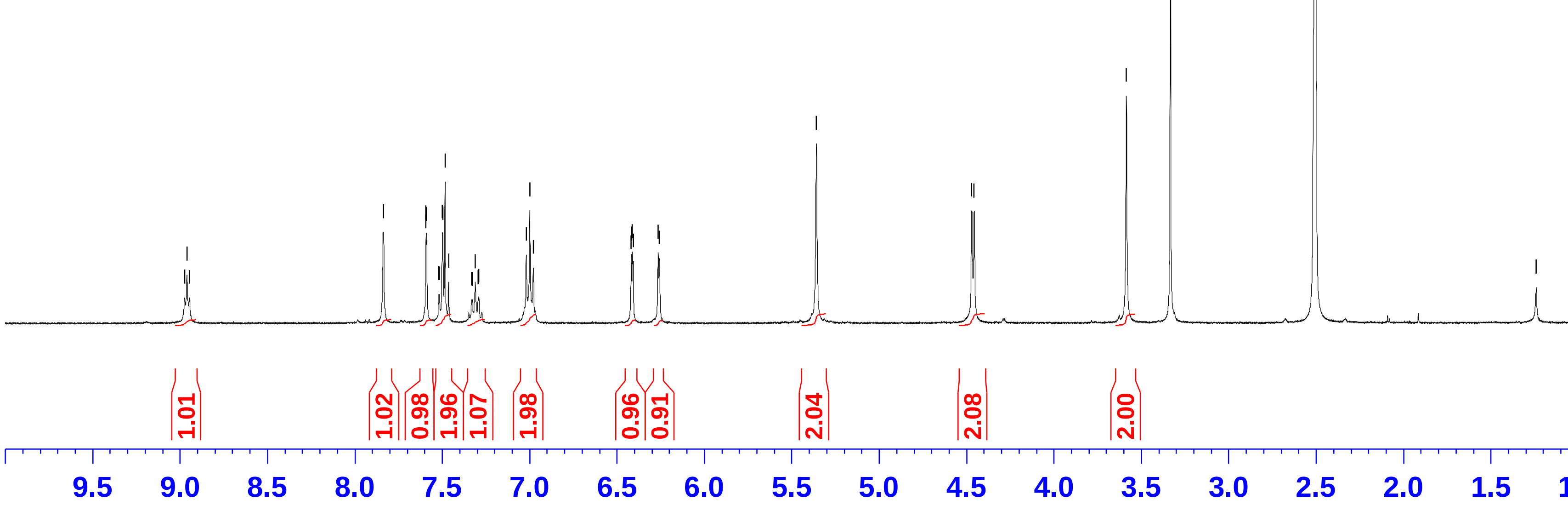


```
Current Data Parameters
NAME           10-06-2019
EXPNO                 130
PROCNO                  1

F2 - Acquisition Parameters
Date_            20190610
Time                15.28
INSTRUM             spect
PROBHD   5 mm PABBO BB/
PULPROG              zg30
TD                  65536
SOLVENT              DMSO
NS                     16
DS                      2
SWH              8012.820 Hz
FIDRES           0.122266 Hz
AQ              4.0894465 sec
RG                 200.06
DW                 62.400 usec
DE                   6.50 usec
TE                  296.5 K
D1             1.00000000 sec
TD0                     1

======== CHANNEL f1 ========
SFO1          400.2464717 MHz
NUC1                   1H
P1                  12.85 usec
PLW1          15.00000000 W

F2 - Processing parameters
SI                  65536
SF            400.2440000 MHz
WDW                    no
SSB      0
LB       0 Hz
GB       0
PC                   1.00
```

Compound 2 (9c)

TCG Lifesciences Private Limited

Kolkata

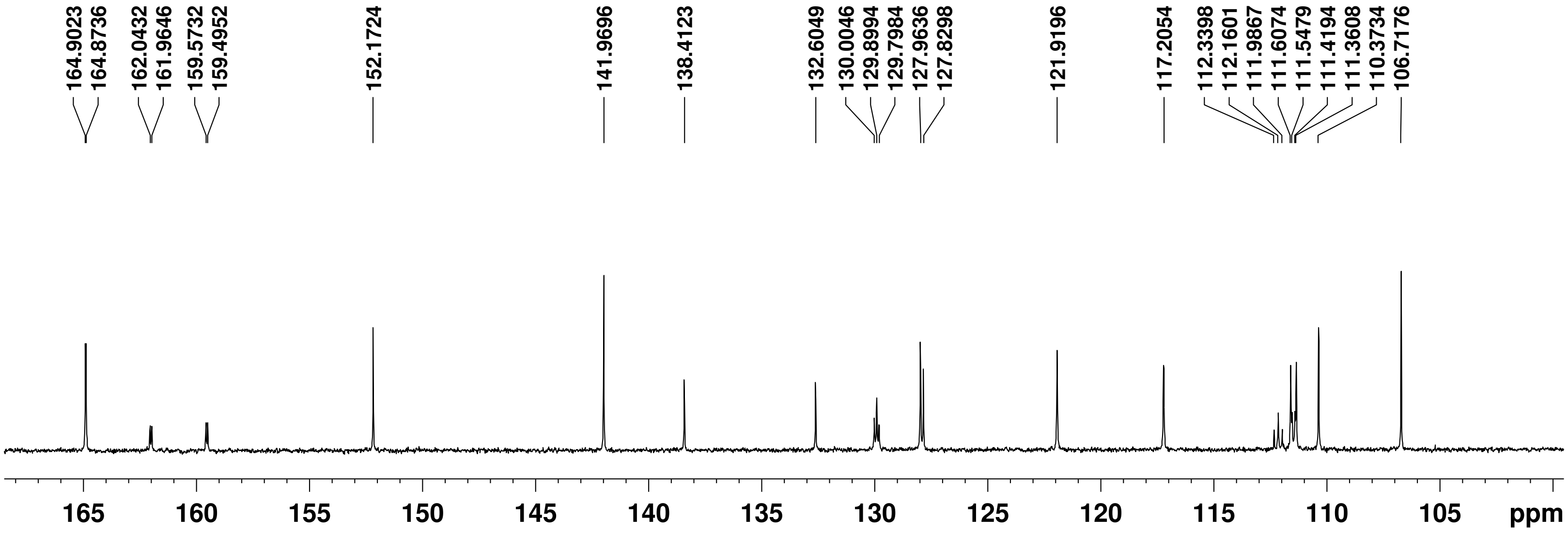

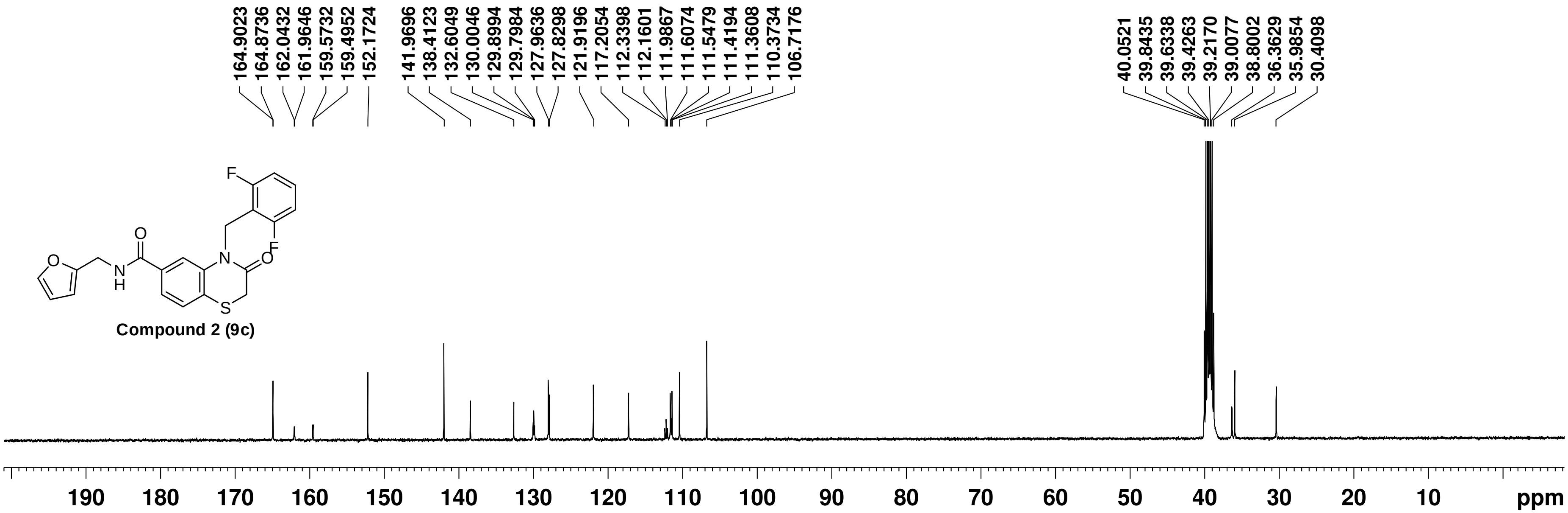

Compound 2 (9c)

Compound 2 (9c)

TCG Lifesciences Private Limited

Kolkata

CRD2882B2 IN DMSO-APT

TCGLS/ARD/NMR02/K02

NAME	CRD2882B2
EXPNO	61
PROCNO	1
Date_	20200708
Time	6.18 h
INSTRUM	spect
PROBHD	Z8246_0048 (PH
PULPROG	jmod
TD	32768
SOLVENT	DMSO
NS	4042
DS	4
SWH	25252.525 Hz
FIDRES	1.541292 Hz
AQ	0.6488564 sec
RG	64
DW	19.800 usec
DE	6.50 usec
TE	297.2 K
CNST2	145.0000000
CNST11	1.0000000
D1	2.00000000 sec
D20	0.00689655 sec
TD0	1
SFO1	100.6152855 MHz
NUC1	13C
P1	9.10 usec
P2	18.20 usec
SI	16384
SF	100.6052824 MHz
WDW	EM
SSB	0
LB	1.00 Hz
GB	0
PC	1.40

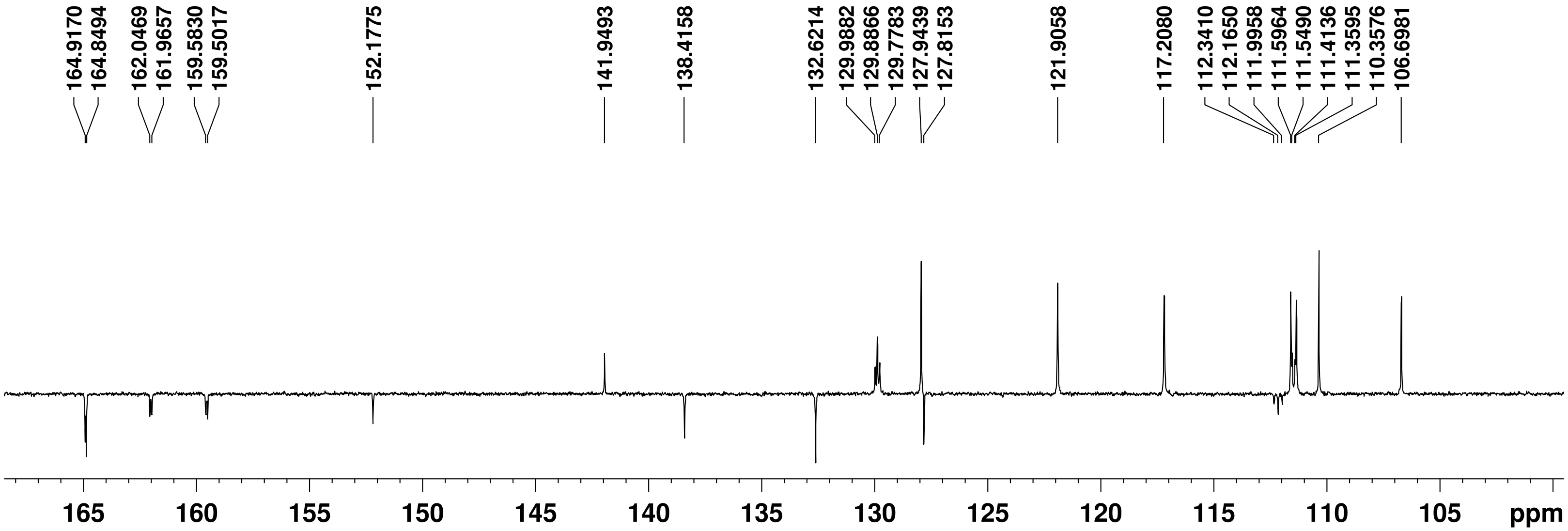

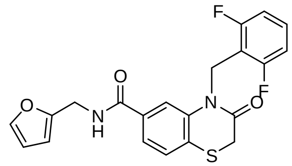

Compound 2 (9c)

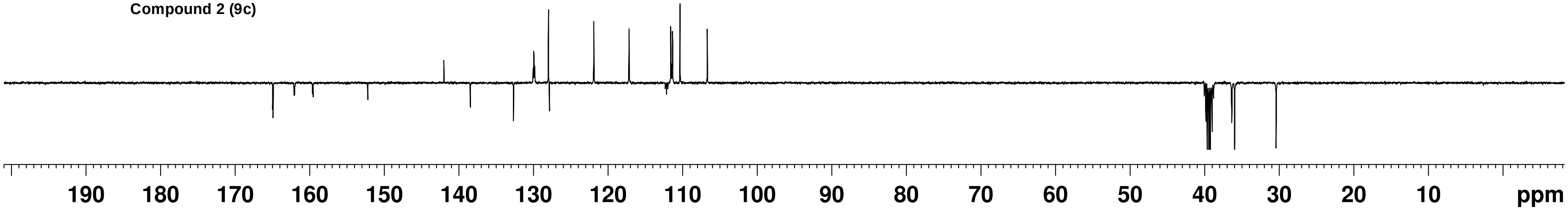

Qualitative Analysis Report

Compound 2 (9c)

**Data Filename**	AS-CRD-2882.d	**Sample Name**	AS-CRD-2882
**Sample Type**	Sample	**Position**	Vial 62
**Instrument Name**	Instrument 1	**User Name**	
**Acq Method**	Direct Mass-2017.m	**Acquired Time**	6/16/2020 12:02:43 PM
**IRM Calibration Status**	Some Ions Missed	**DA Method**	Default.m
**Comment**			

Sample Group **Info.**

Acquisition SW Version 6200 series TOF/6500 series Q-TOF B.05.00 (B5042.0)

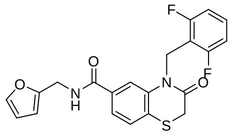

Compound 2 (9c)

Chemical Formula: $C_{21}H_{16}F_2N_2O_3S$

Exact Mass: 414.0850

User Chromatograms

Fragmentor Voltage 118 **Collision Energy** 0 **Ionization Mode** ESI

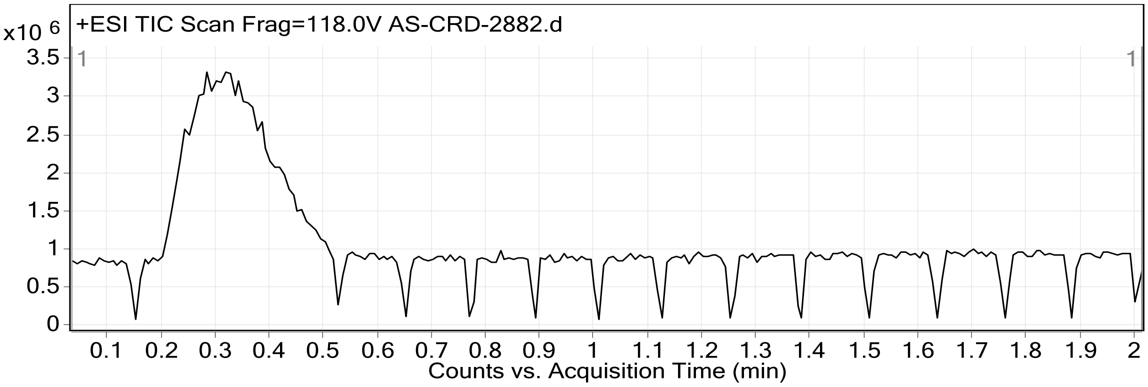

User Spectra

Fragmentor Voltage	Collision Energy	Ionization Mode
118	0	ESI

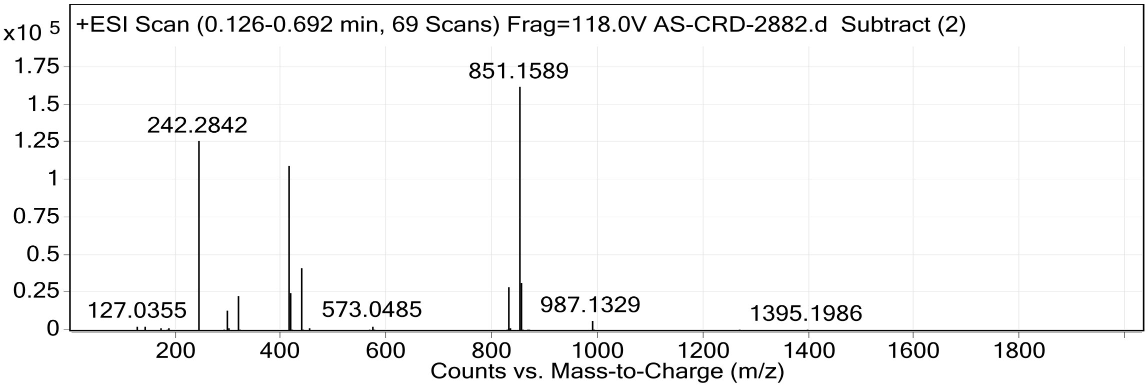

Peak List

m/z	z	Abund
242.2842	1	126409.03
243.2873	1	21239.85
318.0393	1	23733.13
415.0921	1	109862.52
416.0949	1	24931.8
437.0738	1	41918.22

829.1766	1	28693.16
851.1589	1	162583.67
852.1617	1	76317.07
853.1605	1	32298.24

Compound 2 (9c)

Compounds

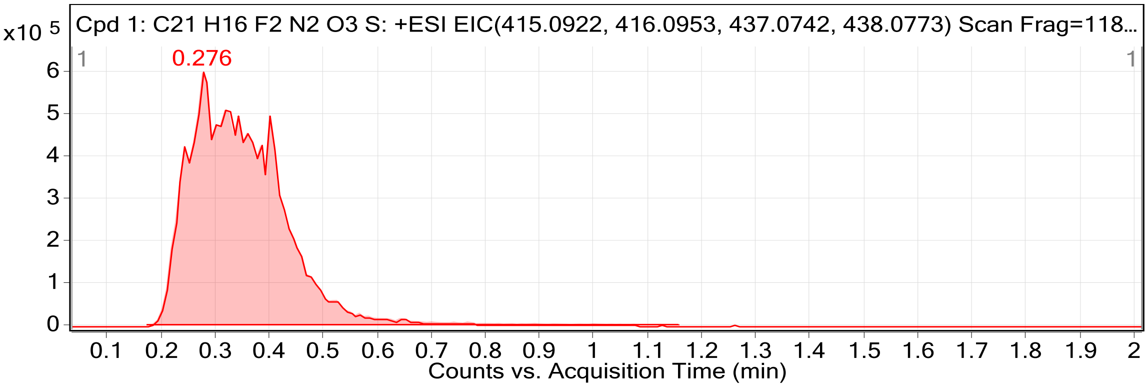

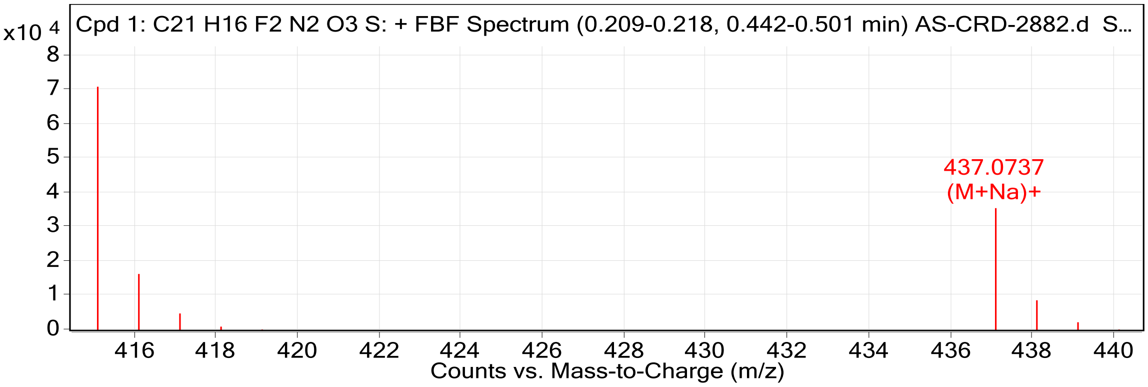

Peak List

m/z	z	Abund	Formula	Ion
415.0918	1	71108.46	C21H17F2N2O3S	(M+H)+
416.0947	1	16713.54	C21H17F2N2O3S	(M+H)+
417.0911	1	4954.84	C21H17F2N2O3S	(M+H)+
418.0931	1	1154.74	C21H17F2N2O3S	(M+H)+
419.1052	1	245.19	C21H17F2N2O3S	(M+H)+
437.0737	1	35785.96	C21H16F2N2NaO3S	(M+Na)+
438.0763	1	8810.1	C21H16F2N2NaO3S	(M+Na)+
439.074	1	2704.59	C21H16F2N2NaO3S	(M+Na)+
440.0746	1	631.06	C21H16F2N2NaO3S	(M+Na)+

Compound 2 (9c)

BATCH NO: **CHEMBIOTEK TCG LIFESCIENCES ENTERPRISE** **18-Jan-201717:07:42**

CR240FFSL-Lib2-1-34-NEW **KOLKATA- INDIA**

INSTRUMENT NO:TCGLS/ARD/LCMS12/K58

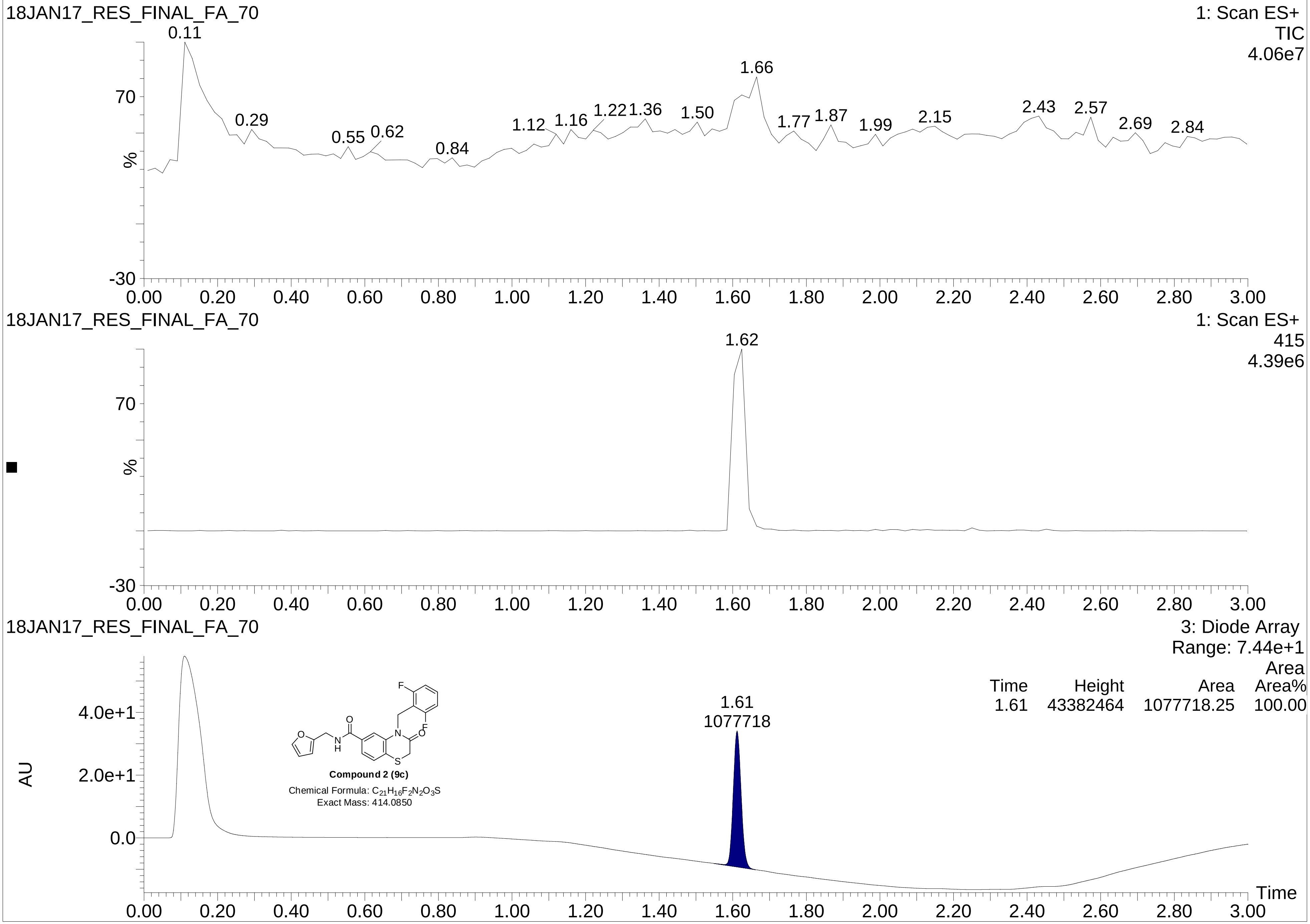

Time	Height	Area	Area Area%
1.61	43382464	1077718.25	100.00

Compound 2 (9c)

BATCH NO: **CR240FFSL-Lib2-1-34-NEW**
INSTRUMENT NO:TCGLS/ARD/LCMS12/K58

CHEMBIOTEK TCG LIFESCIENCES ENTERPRISE
KOLKATA- INDIA

18-Jan-201717:07:42

18JAN17_RES_FINAL_FA_70 81 (1.625)

1: Scan ES+
3.81e6

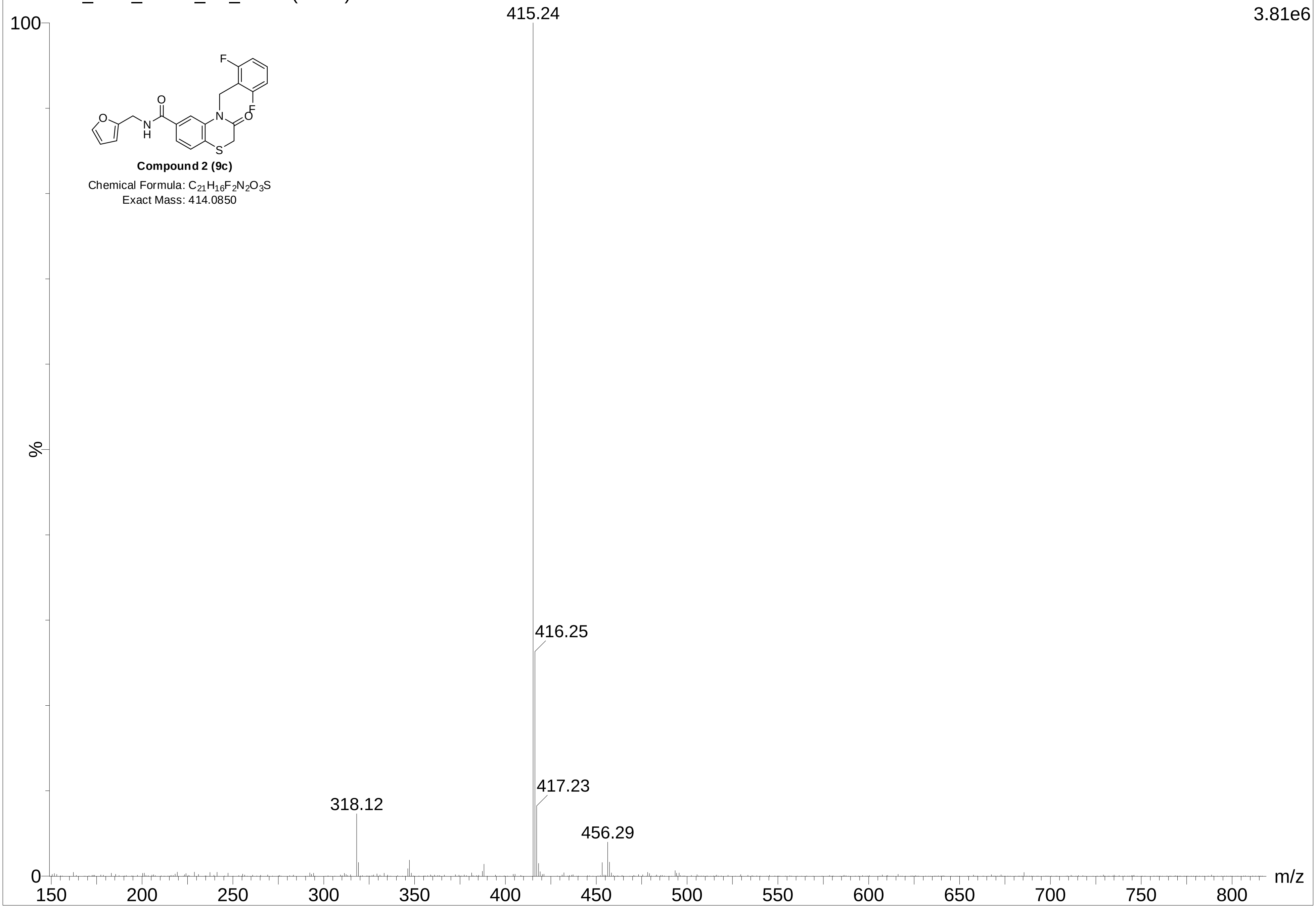

Compound 2 (9c)
Chemical Formula: $C_{21}H_{16}F_2N_2O_3S$
Exact Mass: 414.0850

Empower 3 SOFTWARE

Compound 2 (9c)

SAMPLE INFORMATION

Sample Name:	VK-CA298-132	Acquired By:	UPLC_MS_01 System
Vial:	1:A,4	Sample Set Name:	SAMPLE_FA
Injection #:	2	Acq. Method Set:	FA_C18_6min_N
Injection Volume:	2.00 ul	Processing Method:	UPLC1
Run Time:	6.0 Minutes	Channel Name:	220.0nm@1
Date Acquired:	09-06-2020 16:01:32 IST	Date Processed:	10-06-2020 11:18:06 IST
Column	KINETEX_EVO_C-18	Mobile Phase	0.1% FA in Water/ACN

Auto-Scaled Chromatogram

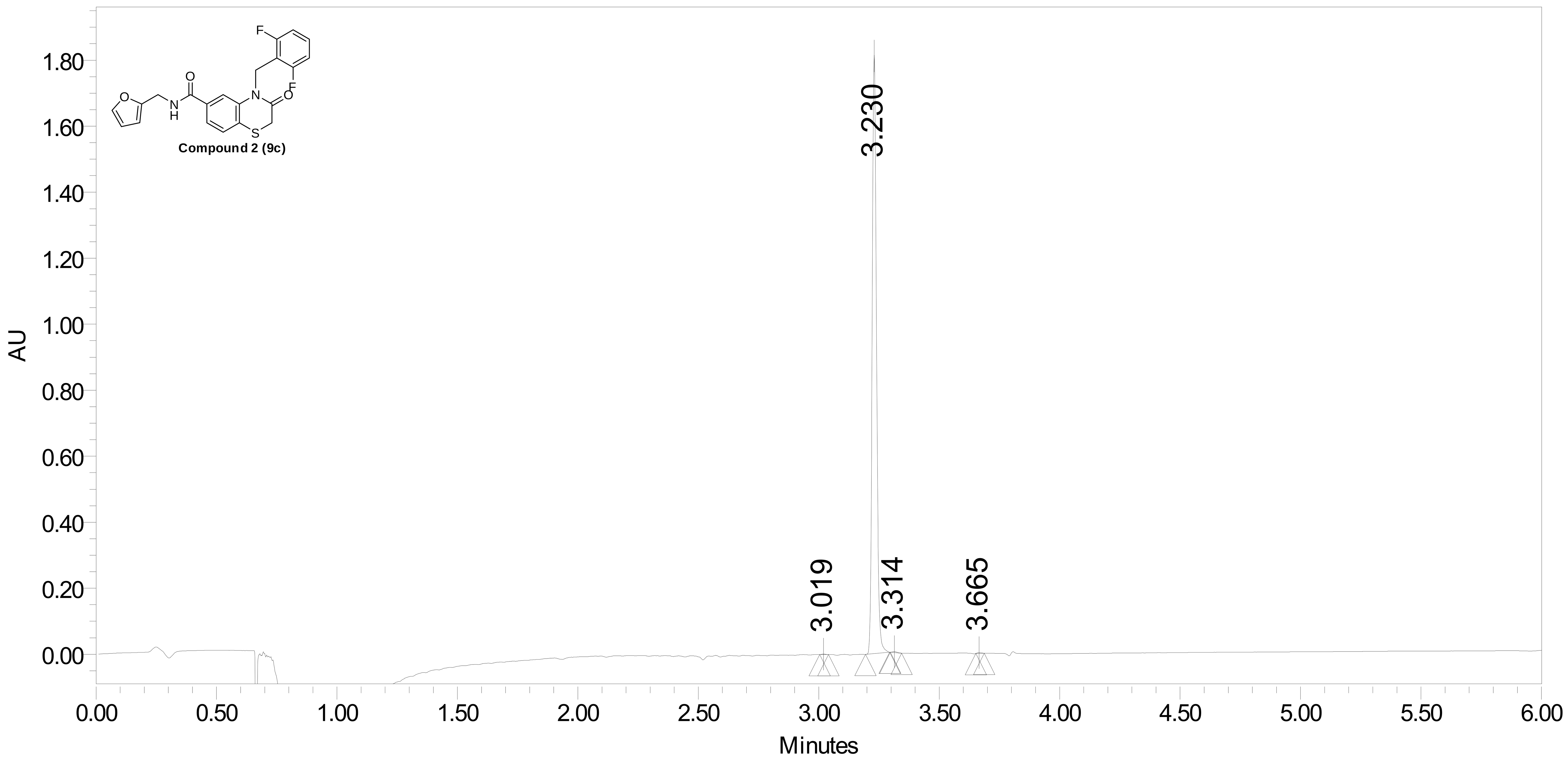

Processed Channel Descr. PDA 220.0 nm (PDA Spectrum (210-400)nm) Blank Subtracted from BLANK_DMSO, Vial 1:F,7 Inj. 1

Peak Results

	Name	RT	Area	% Area	Height
1		3.019	1393	0.06	1474
2		3.230	2263617	99.66	1813780
3		3.314	4064	0.18	3267
4		3.665	2194	0.10	1951

Compound 3 (9d)

SD-CA195-103

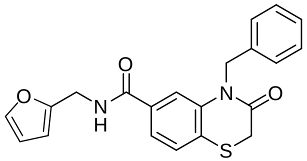

Compound 3 (9d)

Chemical Formula: $C_{21}H_{18}N_2O_3S$

Exact Mass: 378.1038

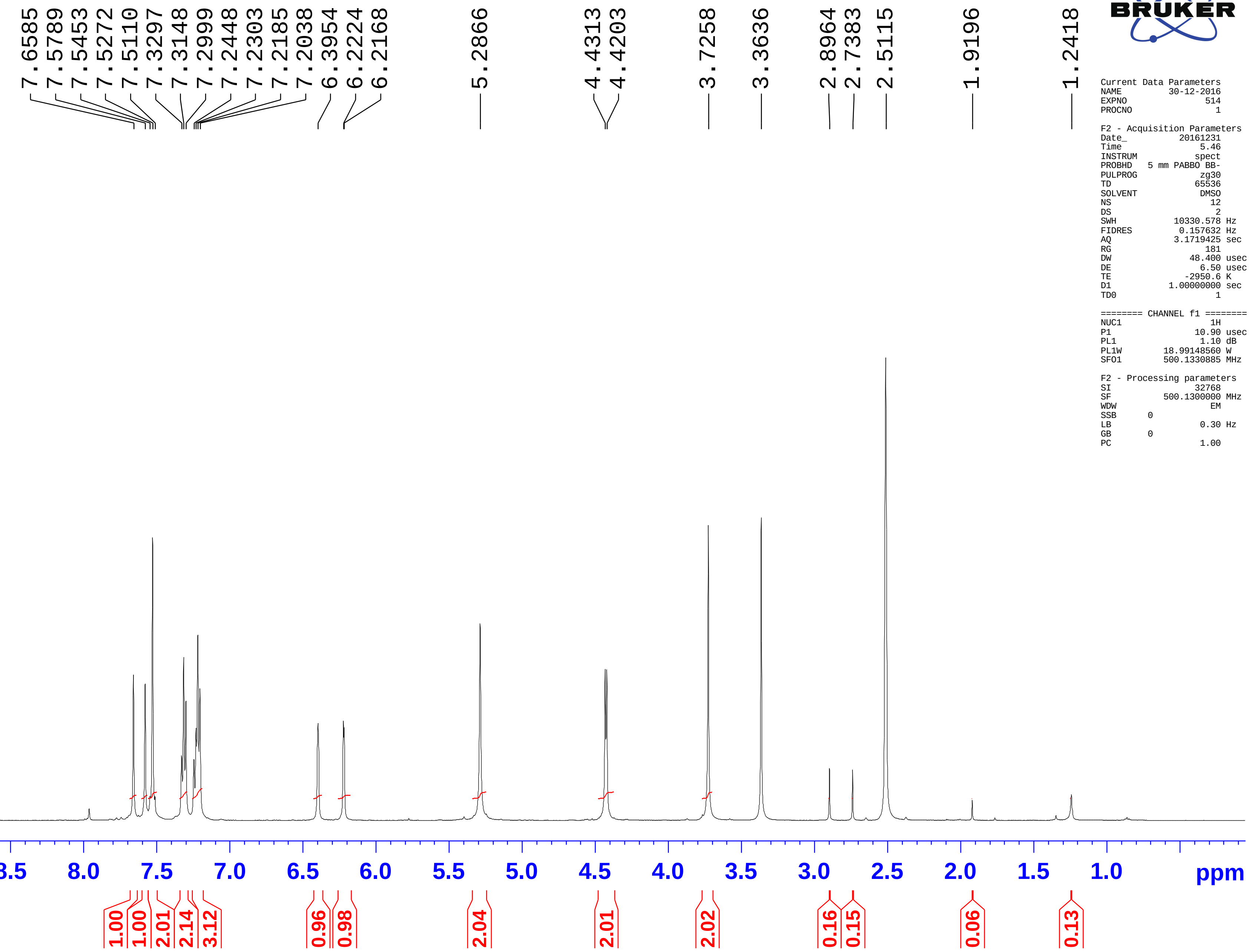


```
Current Data Parameters
NAME            30-12-2016
EXPNO                  514
PROCNO                   1

F2 - Acquisition Parameters
Date_             20161231
Time                  5.46
INSTRUM              spect
PROBHD   5 mm PABBO BB-
PULPROG               zg30
TD                   65536
SOLVENT               DMSO
NS                      12
DS                       2
SWH              10330.578 Hz
FIDRES            0.157632 Hz
AQ               3.1719425 sec
RG                     181
DW                  48.400 usec
DE                    6.50 usec
TE                 -2950.6 K
D1              1.00000000 sec
TD0                      1

======== CHANNEL f1 ========
NUC1                    1H
P1                   10.90 usec
PL1                   1.10 dB
PL1W           18.99148560 W
SFO1           500.1330885 MHz

F2 - Processing parameters
SI                   32768
SF             500.1300000 MHz
WDW                     EM
SSB      0
LB                    0.30 Hz
GB       0
PC                    1.00
```

Qualitative Analysis Report

Compound 3 (9d)

**Data Filename**	AS-CRD-2688.d	**Sample Name**	AS-CRD-2688
**Sample Type**	Sample	**Position**	Vial 63
**Instrument Name**	Instrument 1	**User Name**	
**Acq Method**	Direct Mass-2017.m	**Acquired Time**	6/16/2020 12:06:24 PM
**IRM Calibration Status**	Some Ions Missed	**DA Method**	Default.m
**Comment**			

**Sample Group**		**Info.**
**Acquisition SW Version**	6200 series TOF/6500 series Q-TOF B.05.00 (B5042.0)	

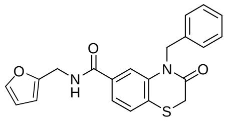

Compound 3 (9d)
Chemical Formula: $C_{21}H_{18}N_2O_3S$
Exact Mass: 378.1038

User Chromatograms

Fragmentor Voltage 118 **Collision Energy** 0 **Ionization Mode** ESI

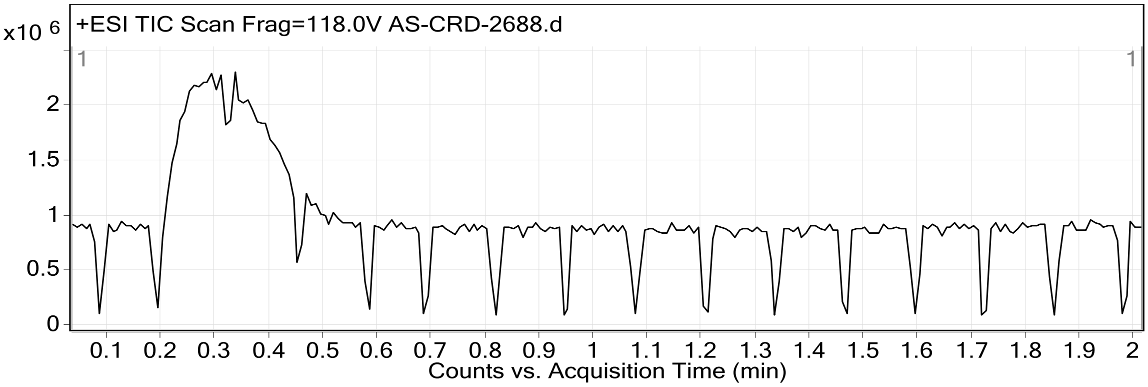

User Spectra

Fragmentor Voltage	Collision Energy	Ionization Mode
118	0	ESI

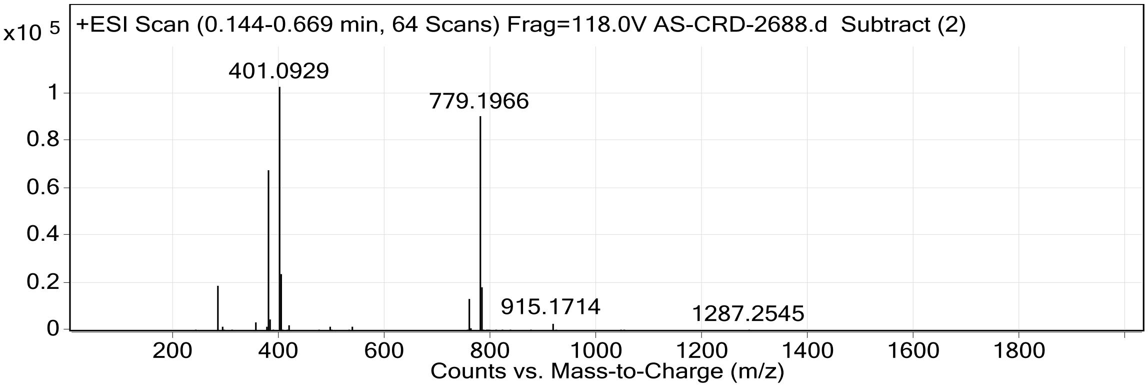

Peak List

m/z	z	Abund
282.0582	1	18929.74
379.1109	1	67596.25
380.1139	1	15572.55
401.0929	1	102996.95
402.0956	1	24087.47
403.0929	1	7014.55

757.2145	1	13780.17
779.1966	1	90267.3
780.1995	1	43069.45
781.1983	1	18508.49

Compound 3 (9d)

Compounds

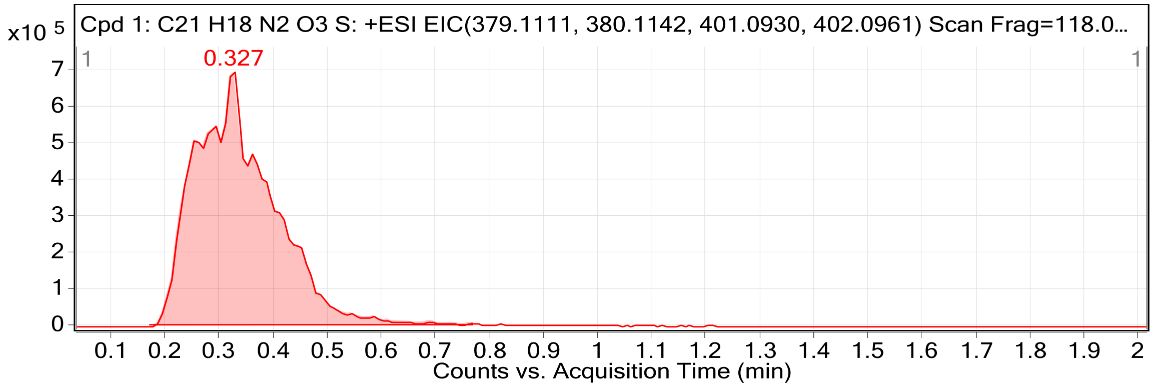

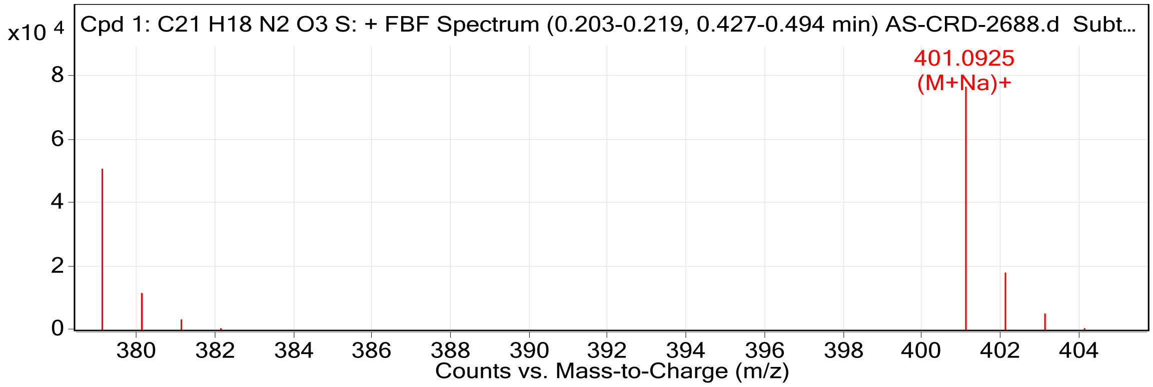

Peak List

m/z	z	Abund	Formula	Ion
379.1107	1	51026.58	C21H19N2O3S	(M+H)+
380.1136	1	11902.92	C21H19N2O3S	(M+H)+
381.1112	1	3597.4	C21H19N2O3S	(M+H)+
382.1118	1	725.45	C21H19N2O3S	(M+H)+
383.1215	1	61.19	C21H19N2O3S	(M+H)+
401.0925	1	76901.88	C21H18N2NaO3S	(M+Na)+
402.0954	1	18556.66	C21H18N2NaO3S	(M+Na)+
403.0925	1	5436.07	C21H18N2NaO3S	(M+Na)+
404.0936	1	1077.1	C21H18N2NaO3S	(M+Na)+
405.0949	1	228.57	C21H18N2NaO3S	(M+Na)+

Compound 3 (9d) — SAMPLE INFORMATION

Sample Name:	SD-CA195-103	Acquired By:	UPLC_MS_01 System
Vial:	1:E,6	Sample Set Name:	FINAL_AA
Injection #:	1	Acq. Method Set:	AA_C18_6min_N
Injection Volume:	0.30 ul	Processing Method:	MASS
Run Time:	6.0 Minutes	Channel Name:	379.2Da
Date Acquired:	15-12-2016 11:05:39 IST	Date Processed:	15-12-2016 11:50:56 IST

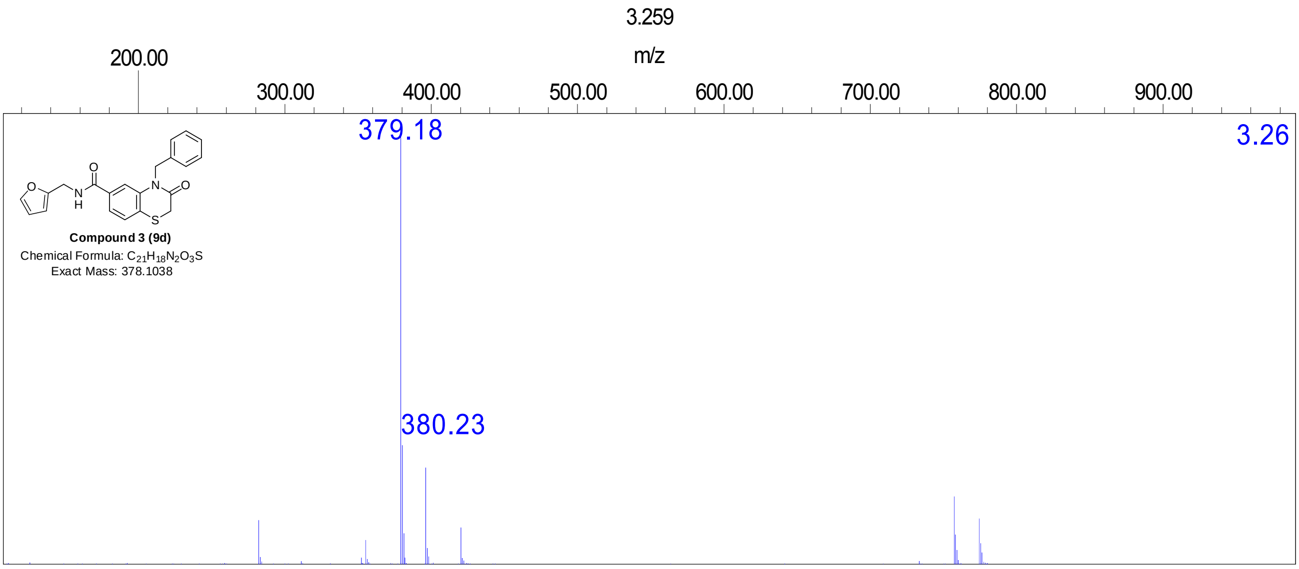

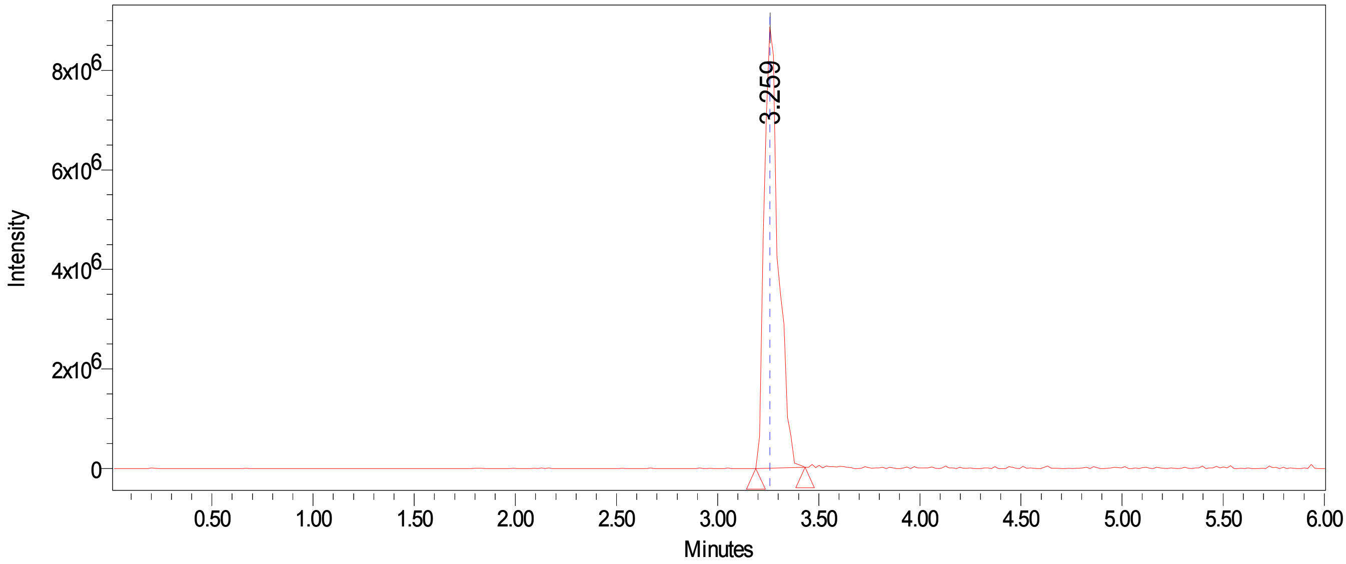

Channel Description 1: 100.00-1000.00 ES+, Centroid, CV=Tune; Processed Channel Descr. W3100 1: MS Scan MS 379.19 m/z Peak Separation: 1.0000 (1: 100.00-1000.00 ES+, Centroid, CV=Tune)

Compound 3 (9d) **SAMPLE INFORMATION**

Sample Name:	SD-CA195-103	Acquired By:	UPLC_MS_01 System
Vial:	1:E,6	Sample Set Name:	FINAL_AA
Injection #:	1	Acq. Method Set:	AA_C18_6min_N
Injection Volume:	0.30 ul	Processing Method:	UPLC
Run Time:	6.0 Minutes	Channel Name:	220.0nm
Date Acquired:	15-12-2016 11:05:39 IST	Date Processed:	15-12-2016 11:50:27 IST
Column	KINETEX EVO C18 (2.1x100mm)	Mobile Phase	5mM AA in Water/ACN

Auto-Scaled Chromatogram

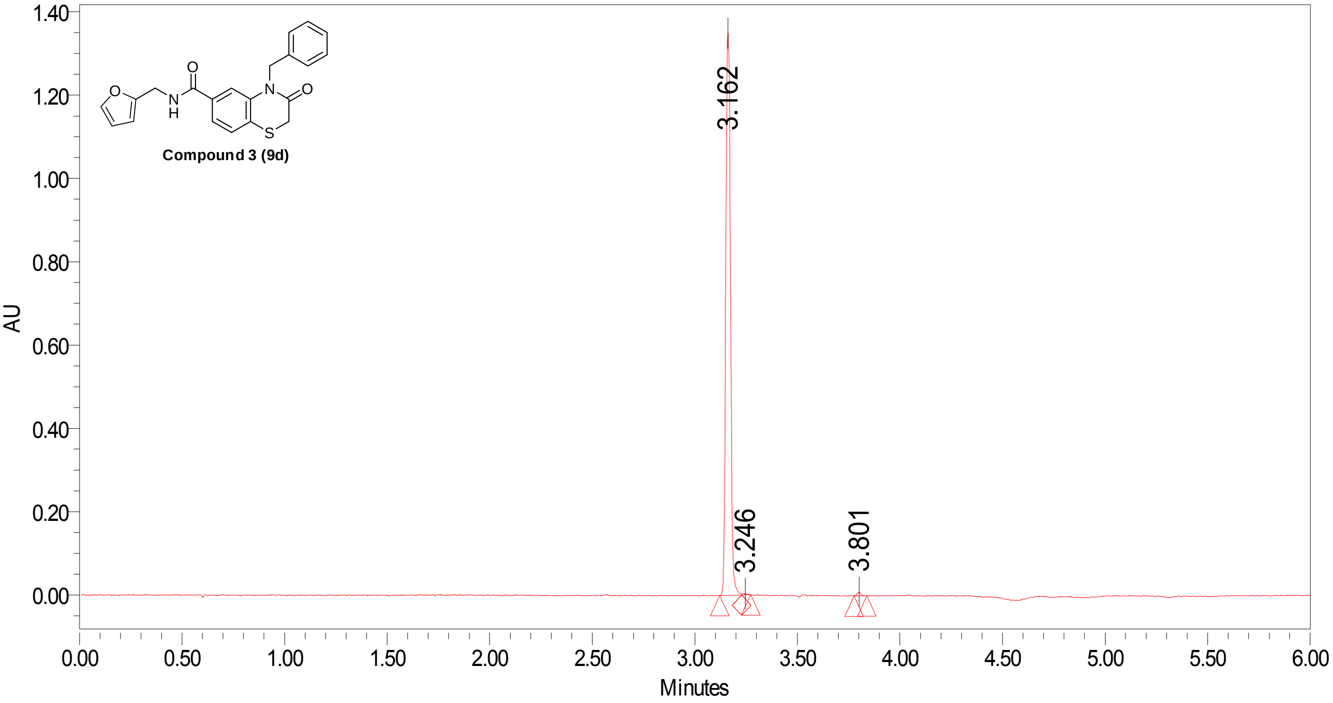

Processed Channel Descr. PDA 220.0 nm (PDA Spectrum (210-400)nm) Blank Subtracted from BLANK_N, Vial 1:F,8 Inj. 1

Peak Results

	Name	RT	Area	% Area	Height
1		3.162	2138421	99.29	1349253
2		3.246	4564	0.21	3394
3		3.801	10645	0.49	8404

Compound 4 (9e)

ND-CA201-80

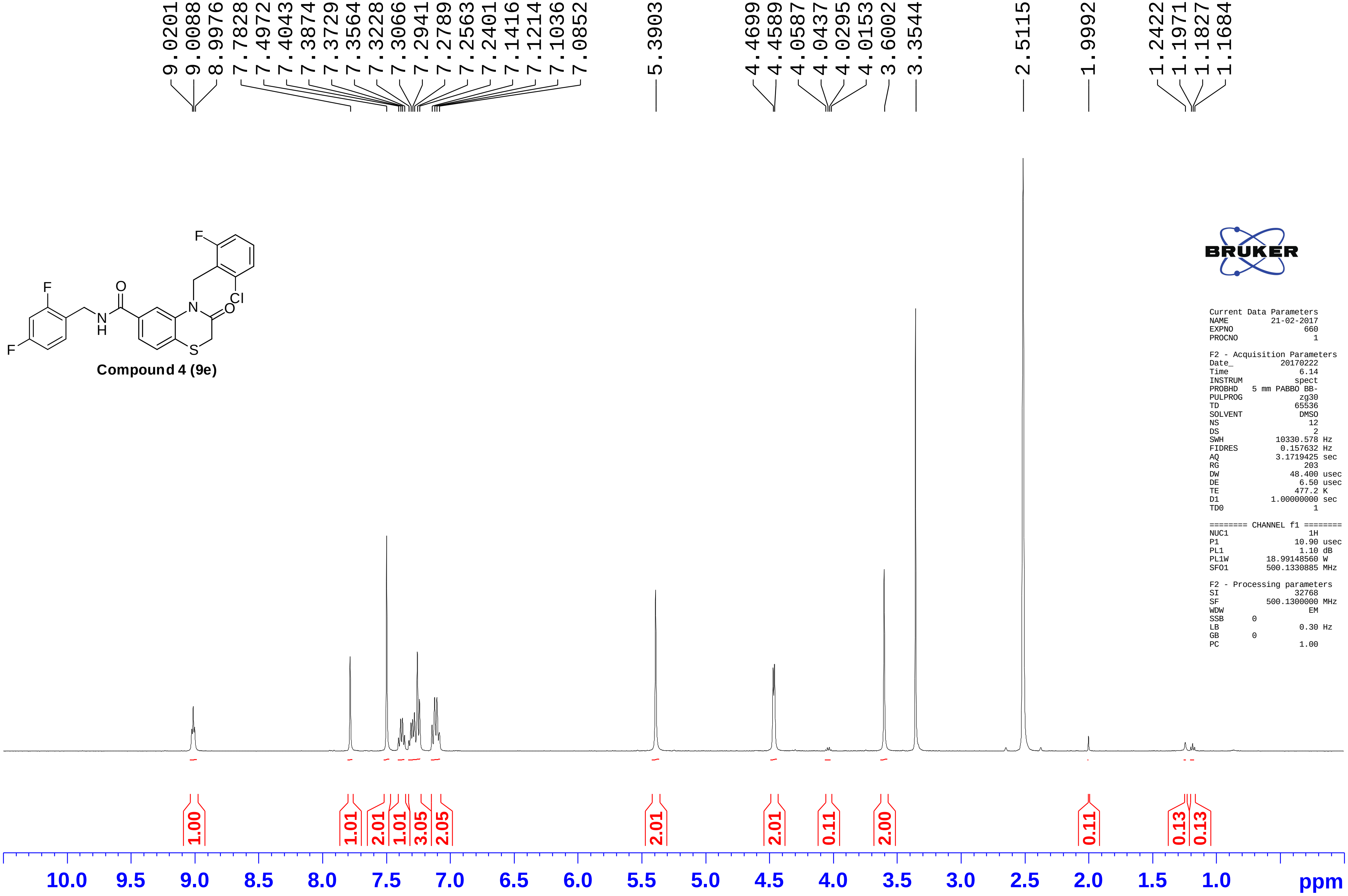

Compound 4 (9e)

Compound 4 (9e)

TCG Lifesciences Private Limited
Kolkata

CRD−2947 IN DMSO−13C

TCGLS/ARD/NMR02/K02

NAME	CRD−2947
EXPNO	60
PROCNO	1
Date_	20200621
Time	11.53 h
INSTRUM	spect
PROBHD	Z8246_0048 (PH
PULPROG	zgpg30
TD	32768
SOLVENT	DMSO
NS	25000
DS	2
SWH	25252.525 Hz
FIDRES	1.541292 Hz
AQ	0.6488564 sec
RG	64
DW	19.800 usec
DE	6.50 usec
TE	297.2 K
D1	2.00000000 sec
D11	0.03000000 sec
TD0	1
SFO1	100.6152855 MHz
NUC1	13C
P0	3.03 usec
P1	9.10 usec
SI	16384
SF	100.6052821 MHz
WDW	EM
SSB	0
LB	1.00 Hz
GB	0
PC	1.40

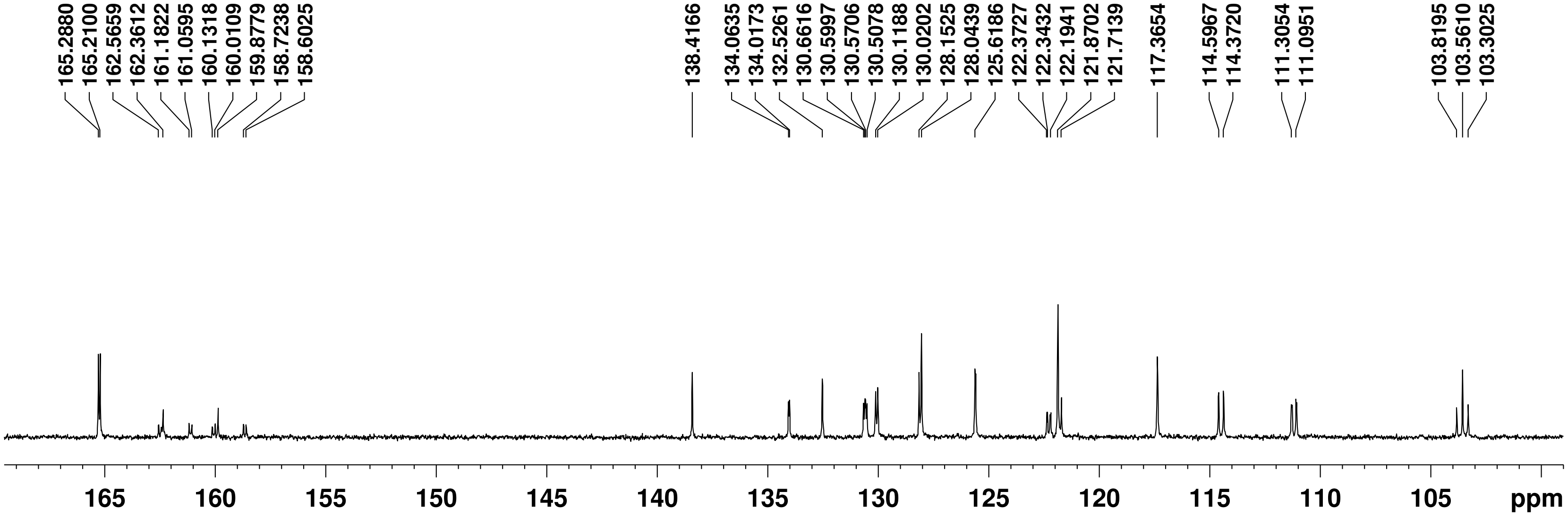

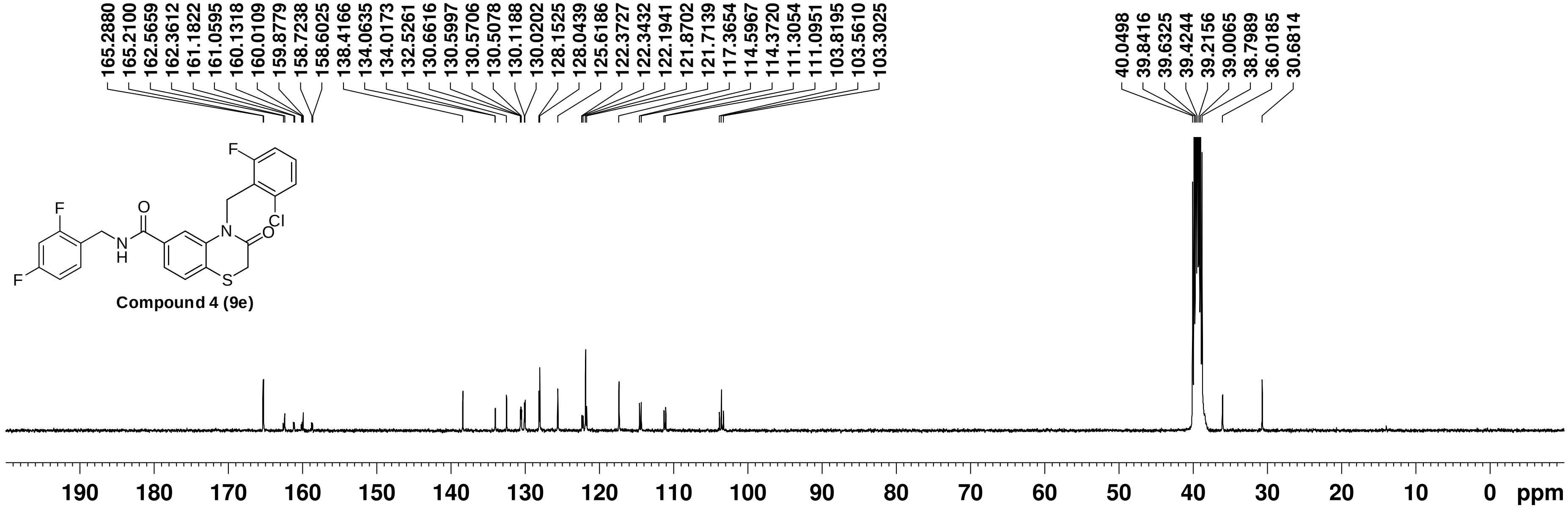

Compound 4 (9e)

Compound 4 (9e)

TCG Lifesciences Private Limited

Kolkata

NAME CRD−2947
EXPNO 61
PROCNO 1
Date_ 20200621
Time 23.07 h
INSTRUM spect
PROBHD Z8246_0048 (PH
PULPROG jmod
TD 32768
SOLVENT DMSO
NS 15000
DS 4
SWH 25252.525 Hz
FIDRES 1.541292 Hz
AQ 0.6488564 sec
RG 64
DW 19.800 usec
DE 6.50 usec
TE 297.2 K
CNST2 145.0000000
CNST11 1.0000000
D1 2.00000000 sec
D20 0.00689655 sec
TD0 1
SFO1 100.6152855 MHz
NUC1 13C
P1 9.10 usec
P2 18.20 usec
SI 16384
SF 100.6052820 MHz
WDW EM
SSB 0
LB 1.00 Hz
GB 0
PC 1.00

CRD−2947 IN DMSO−APT

TCGLS/ARD/NMR02/K02

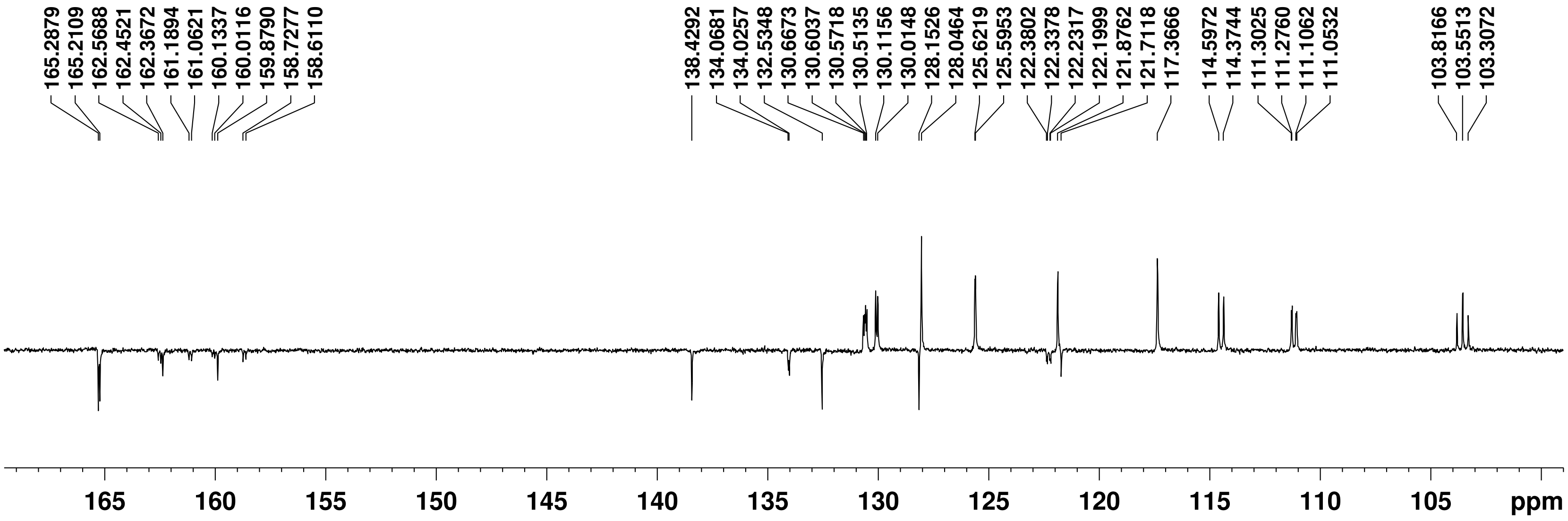

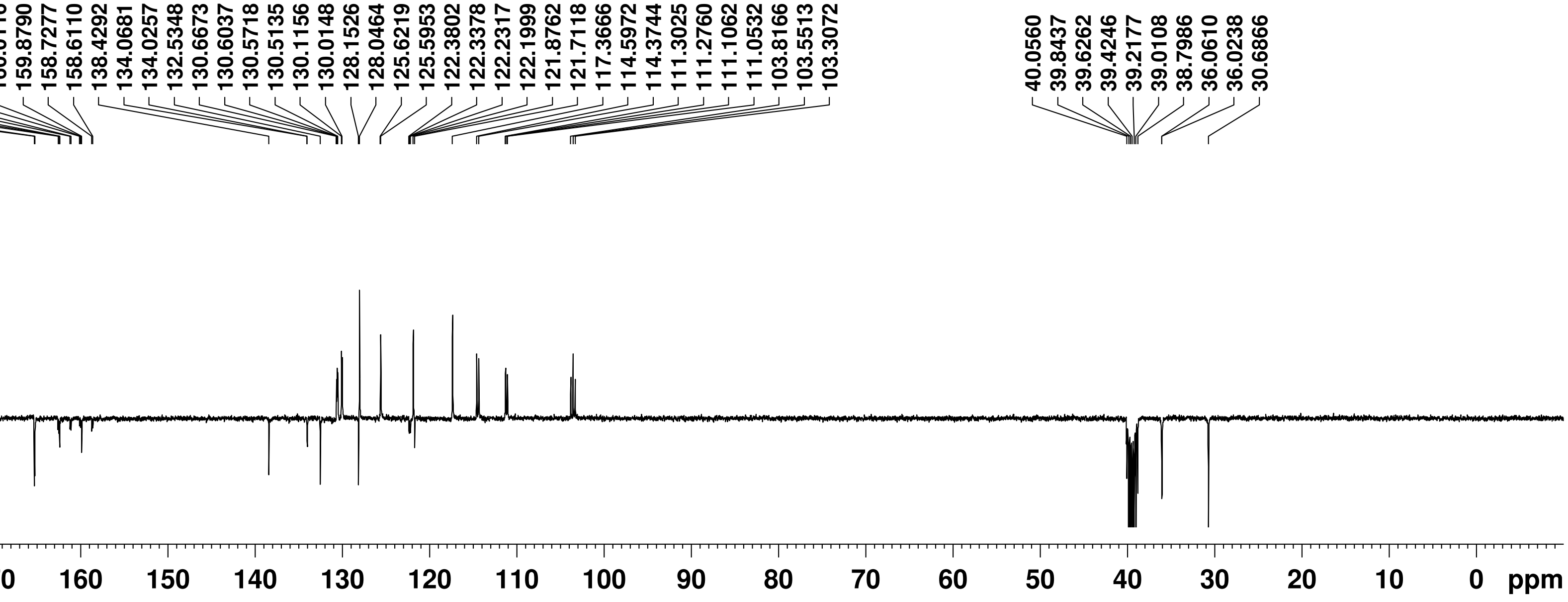

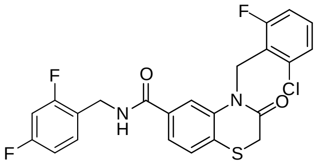

Compound 4 (9e)

Chemical Formula: $C_{23}H_{16}ClF_3N_2O_2S$

Exact Mass: 476.0573

Qualitative Analysis Report

Compound 4 (9e)

**Data Filename**	AS-CRD-2947.d	**Sample Name**	AS-CRD-2947
**Sample Type**	Sample	**Position**	Vial 64
**Instrument Name**	Instrument 1	**User Name**	
**Acq Method**	Direct Mass-2017.m	**Acquired Time**	6/16/2020 12:10:00 PM
**IRM Calibration Status**	Some Ions Missed	**DA Method**	Default.m
**Comment**			

Sample Group **Info.**

Acquisition SW Version 6200 series TOF/6500 series Q-TOF B.05.00 (B5042.0)

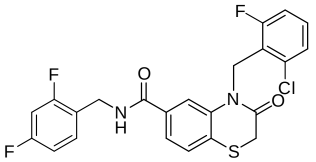

Compound 4 (9e)

Chemical Formula: $C_{23}H_{16}ClF_3N_2O_2S$

Exact Mass: 476.0573

User Chromatograms

Fragmentor Voltage 118 **Collision Energy** 0 **Ionization Mode** ESI

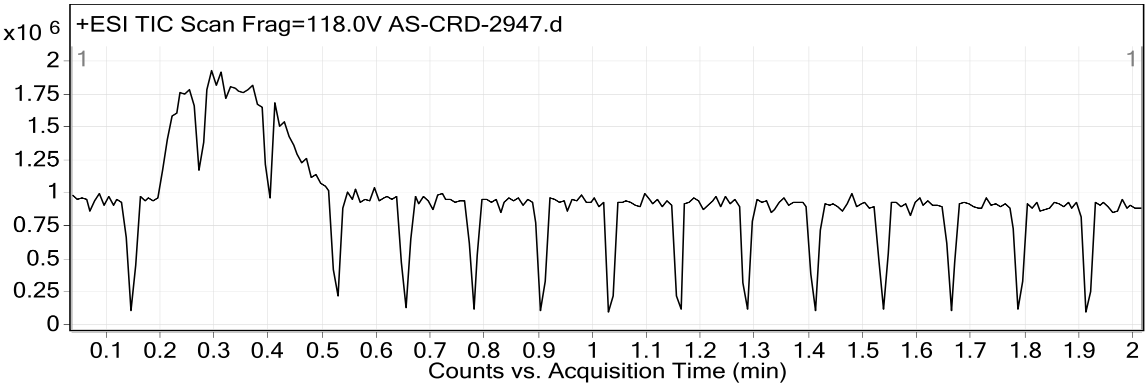

User Spectra

**Fragmentor Voltage**	**Collision Energy**	**Ionization Mode**
118	0	ESI

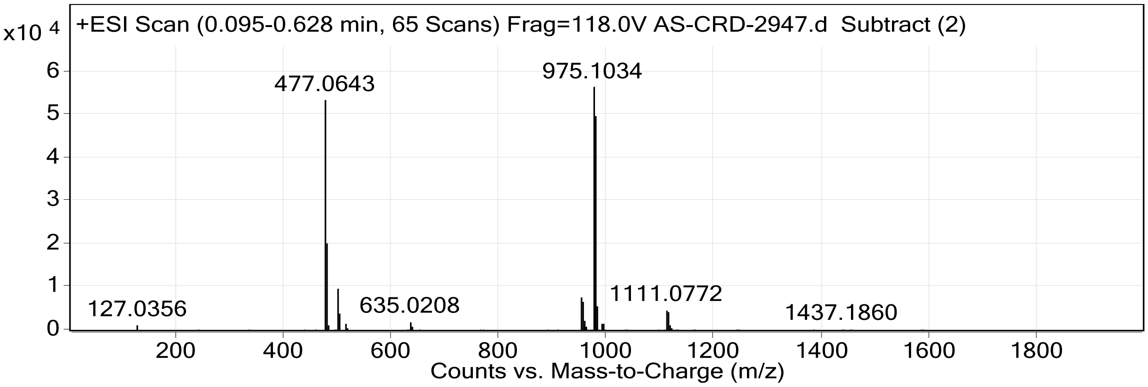

Peak List

m/z	z	Abund
477.0643	1	53563.73
478.0672	1	13651.37
479.062	1	20309.12
499.0463	1	9858.47
953.1212	1	7703.67
975.1034	1	56528.74

976.1064	1	29849.19
977.1019	1	49587.28
978.1041	1	23260
979.1007	1	15051.92

Compound 4 (9e)

Compounds

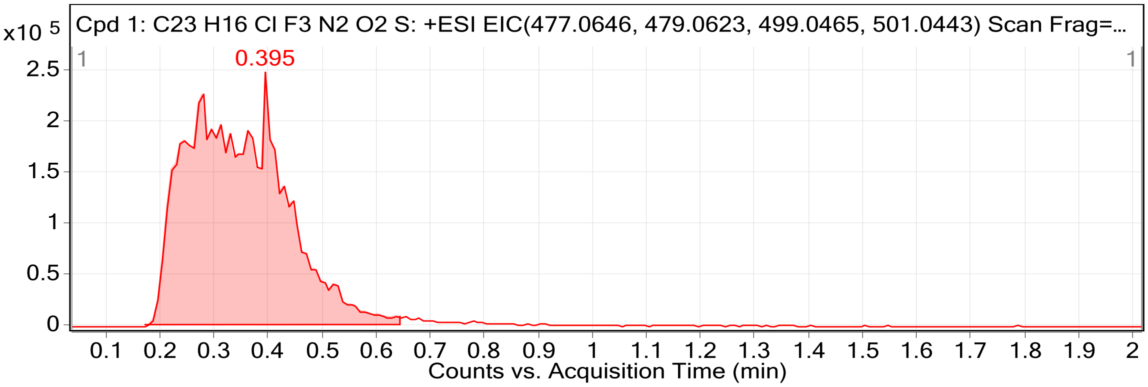

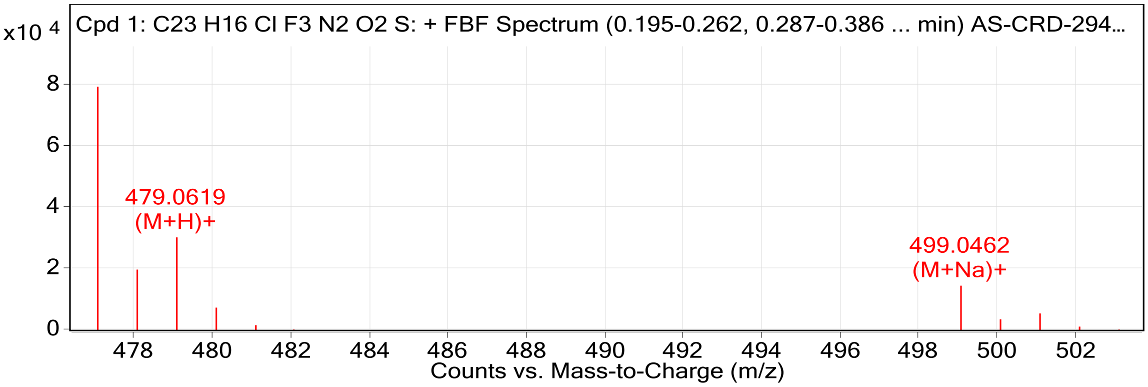

Peak List

m/z	z	Abund	Formula	Ion
477.0642	1	79670.77	C23H17ClF3N2O2S	(M+H)+
478.0671	1	20192.89	C23H17ClF3N2O2S	(M+H)+
479.0619	1	30370.38	C23H17ClF3N2O2S	(M+H)+
480.0643	1	7617.63	C23H17ClF3N2O2S	(M+H)+
481.0624	1	2102.09	C23H17ClF3N2O2S	(M+H)+
482.0616	1	501.34	C23H17ClF3N2O2S	(M+H)+
499.0462	1	14604.52	C23H16ClF3N2NaO2S	(M+Na)+
500.0494	1	3926.52	C23H16ClF3N2NaO2S	(M+Na)+
501.0438	1	5857.42	C23H16ClF3N2NaO2S	(M+Na)+
502.0464	1	1491.2	C23H16ClF3N2NaO2S	(M+Na)+

Compound 4 (9e) **S A M P L E   I N F O R M A T I O N**

Sample Name:	ND-CA201-80	Acquired By:	UPLC_MS_01 System
Vial:	2:D,5	Sample Set Name:	FA
Injection #:	1	Acq. Method Set:	PH HEX_FA_6min
Injection Volume:	0.10 ul	Processing Method:	MASS
Run Time:	6.0 Minutes	Channel Name:	477.3Da
Date Acquired:	17-02-2017 12:52:51 IST	Date Processed:	17-02-2017 14:44:06 IST

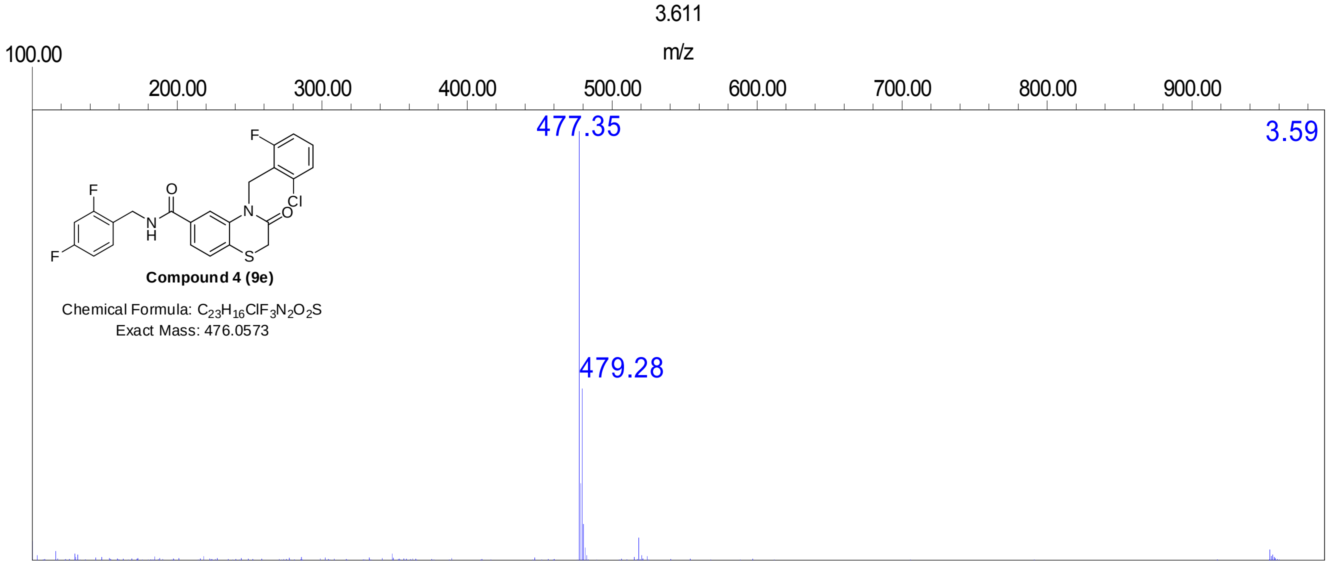

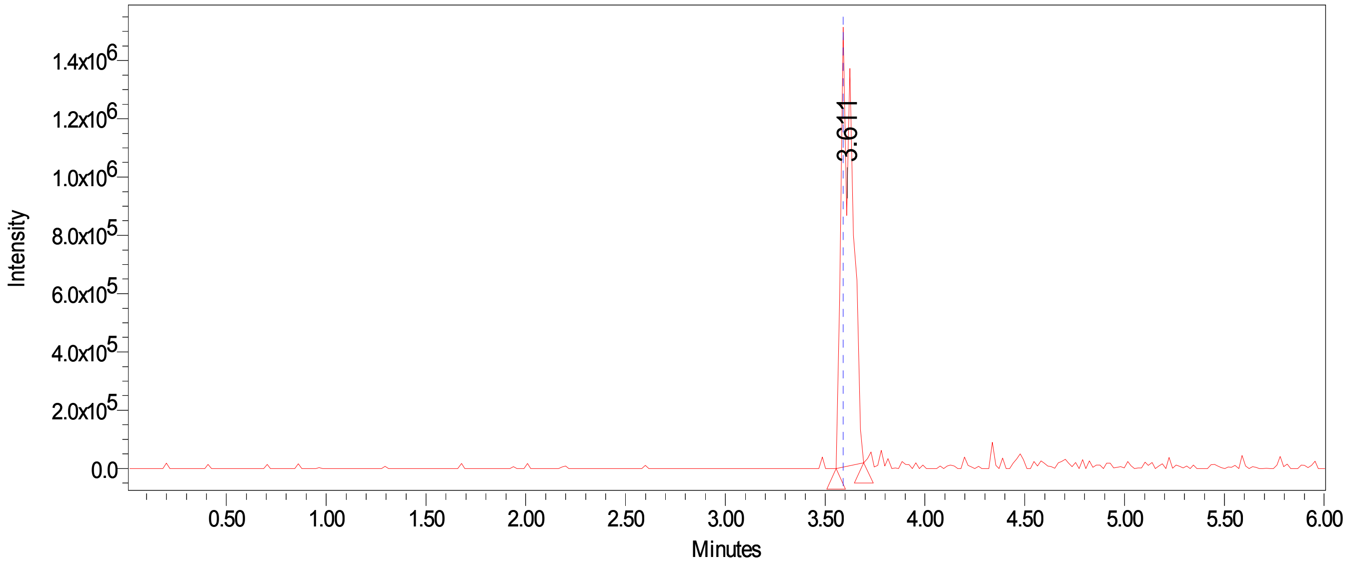

Channel Description 1: 100.00-1000.00 ES+, Centroid, CV=Tune; Processed Channel Descr. W3100 1: MS Scan MS 477.33 m/z Peak Separation: 1.0000 (1: 100.00-1000.00 ES+, Centroid, CV=Tune)

Compound 4 (9e)

SAMPLE INFORMATION

Sample Name:	ND-CA201-80	Acquired By:	UPLC_MS_01 System
Vial:	2:D,5	Sample Set Name:	FA
Injection #:	1	Acq. Method Set:	PH HEX_FA_6min
Injection Volume:	0.10 ul	Processing Method:	UPLC
Run Time:	6.0 Minutes	Channel Name:	240.0nm
Date Acquired:	17-02-2017 12:52:51 IST	Date Processed:	17-02-2017 14:43:27 IST
Column	PHENYL HEXYL (2.1x100mm)	Mobile Phase	0.1% FA in Water/ACN

Auto-Scaled Chromatogram

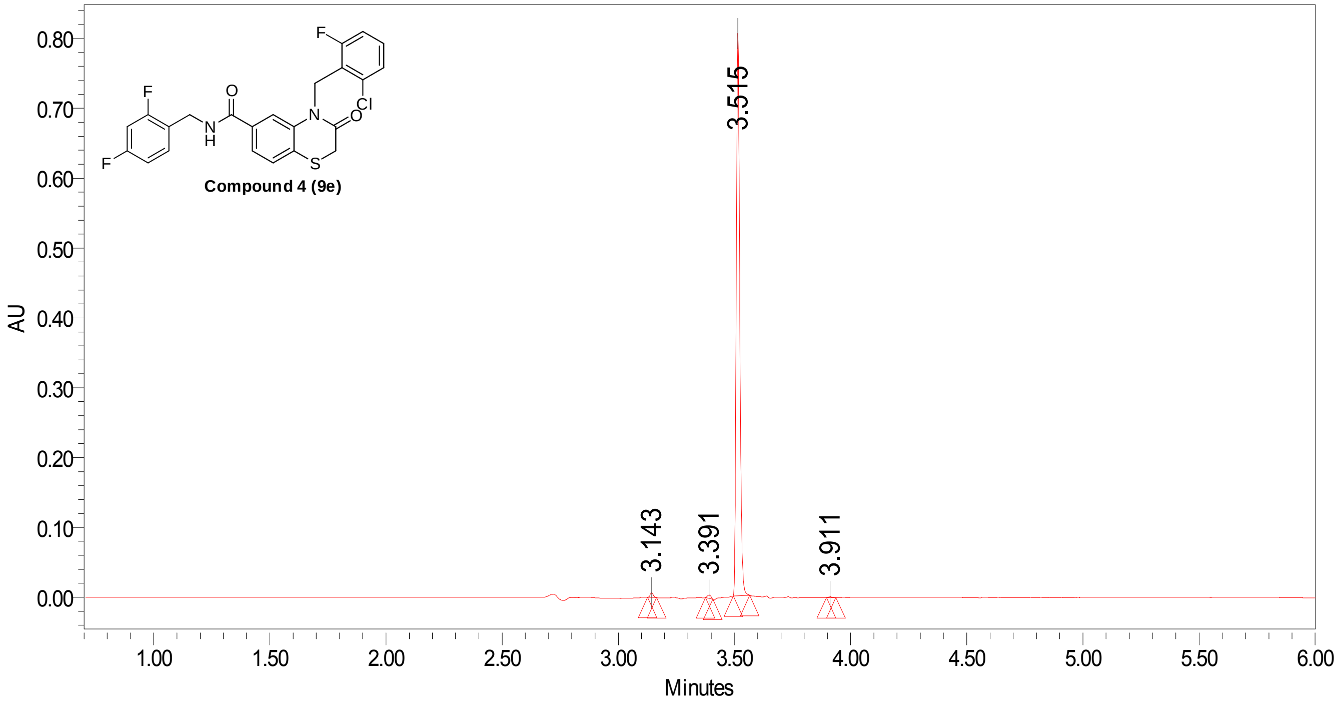

Compound 4 (9e)

Processed Channel Descr. PDA 240.0 nm (PDA Spectrum (210-400)nm) Blank Subtracted from BLANK, Vial 1:F,8 Inj. 1

Peak Results

	Name	RT	Area	% Area	Height
1		3.143	6166	0.72	6578
2		3.391	5294	0.62	4840
3		3.515	843206	98.54	806478
4		3.911	1014	0.12	962

Compound 5 (9f)

SN/CA202/82

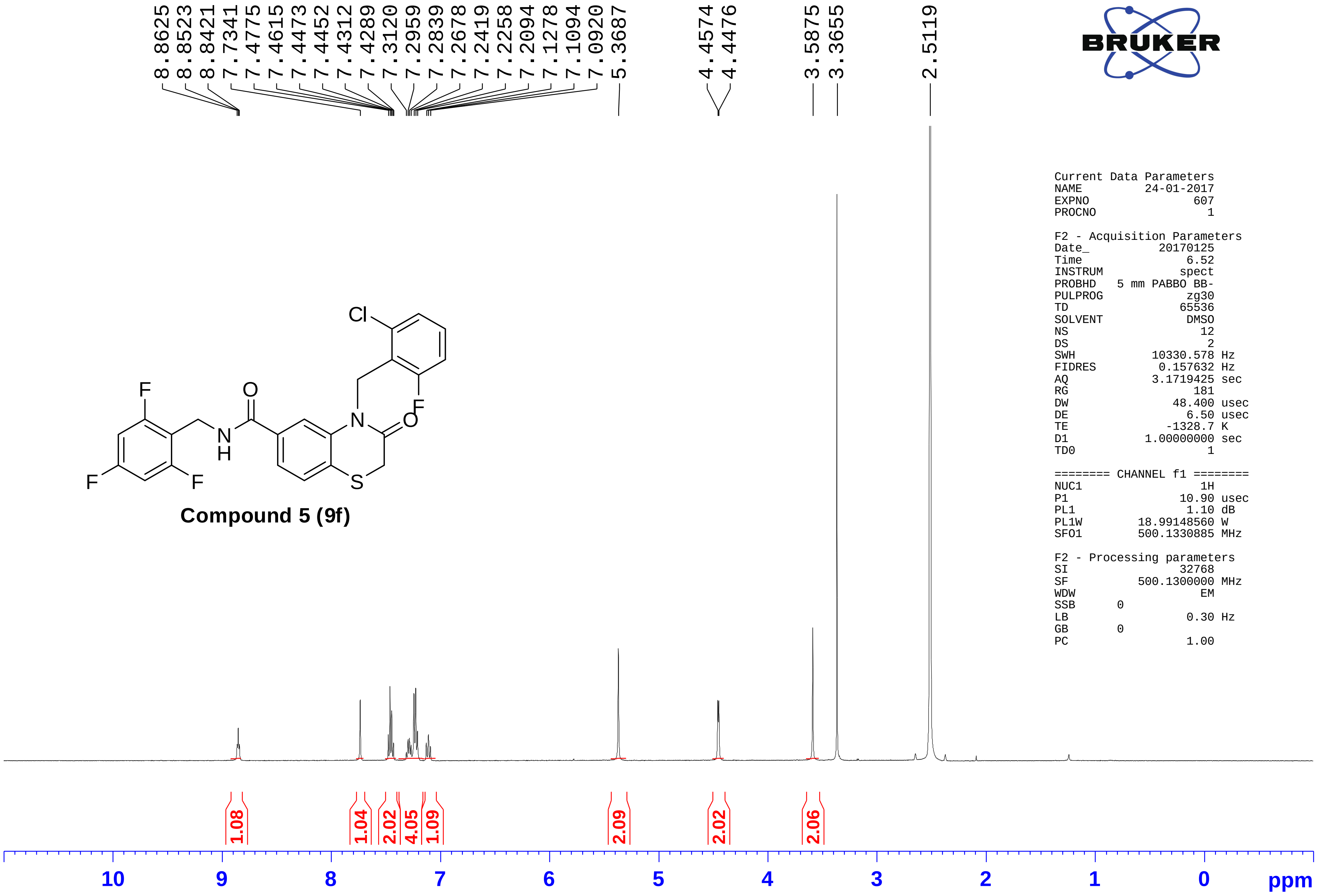

Compound 5 (9f)

```
Current Data Parameters
NAME               24-01-2017
EXPNO                     607
PROCNO                      1

F2 - Acquisition Parameters
Date_                20170125
Time                     6.52
INSTRUM                 spect
PROBHD   5 mm PABBO BB-
PULPROG                  zg30
TD                      65536
SOLVENT                  DMSO
NS                         12
DS                          2
SWH                 10330.578 Hz
FIDRES               0.157632 Hz
AQ                  3.1719425 sec
RG                        181
DW                     48.400 usec
DE                       6.50 usec
TE                    -1328.7 K
D1                 1.00000000 sec
TD0                         1

======== CHANNEL f1 ========
NUC1                       1H
P1                      10.90 usec
PL1                      1.10 dB
PL1W              18.99148560 W
SFO1              500.1330885 MHz

F2 - Processing parameters
SI                      32768
SF                500.1300000 MHz
WDW                        EM
SSB      0
LB                       0.30 Hz
GB       0
PC                       1.00
```

Compound 5 (9f)

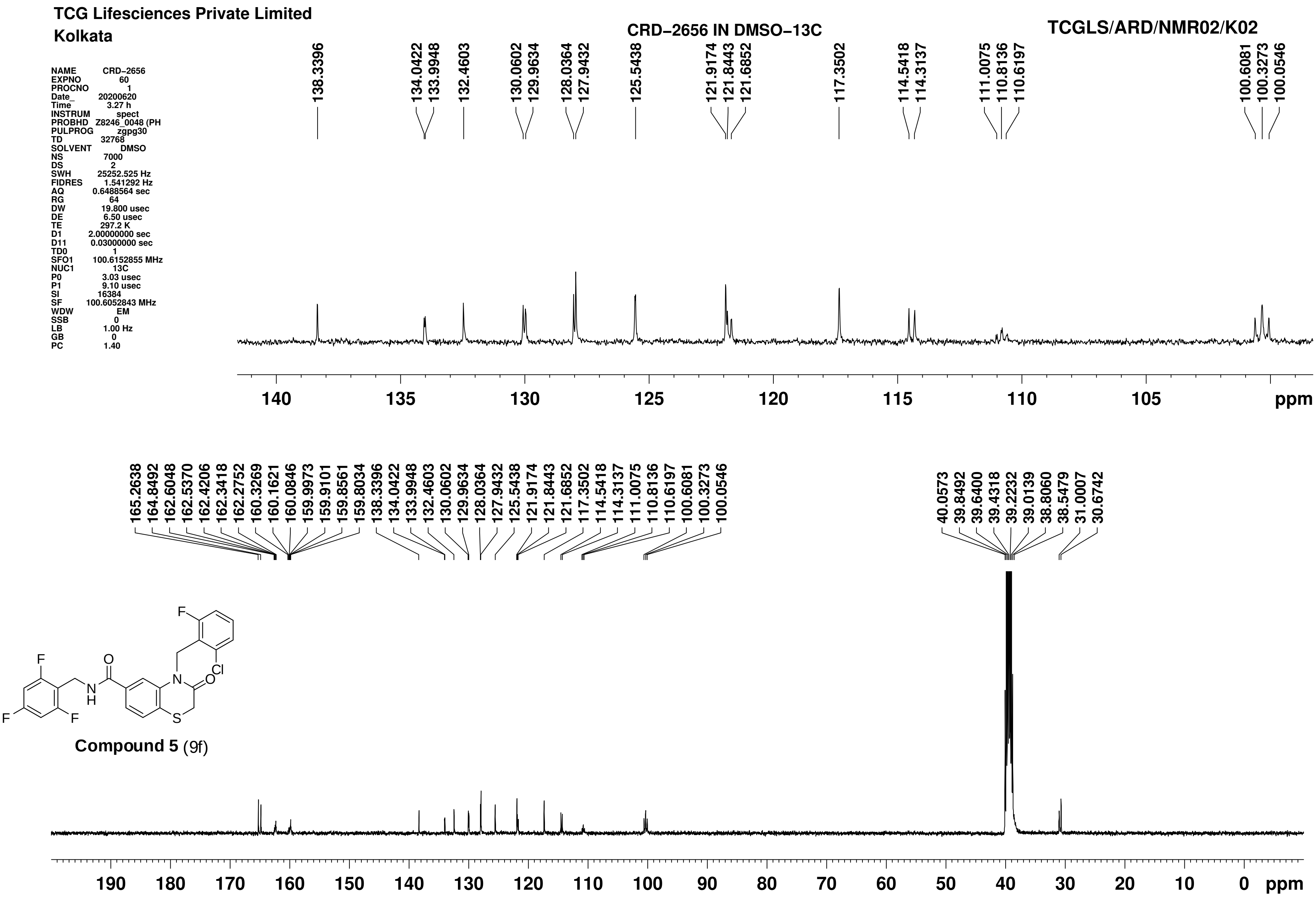

Qualitative Analysis Report

Compound 5 (9f)

**Data Filename**	AS-CRD-2656.d	**Sample Name**	AS-CRD-2656
**Sample Type**	Sample	**Position**	Vial 65
**Instrument Name**	Instrument 1	**User Name**	
**Acq Method**	Direct Mass-2017.m	**Acquired Time**	6/16/2020 12:13:33 PM
**IRM Calibration Status**	Some Ions Missed	**DA Method**	Default.m
**Comment**			

Sample Group **Info.**
Acquisition SW Version 6200 series TOF/6500 series Q-TOF B.05.00 (B5042.0)

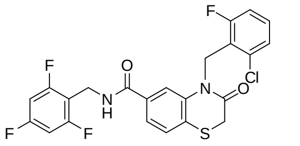

Compound 5 (9f)
Chemical Formula: $C_{23}H_{15}ClF_4N_2O_2S$
Exact Mass: 494.0479
Molecular Weight: 494.8890

User Chromatograms

Fragmentor Voltage 118 **Collision Energy** 0 **Ionization Mode** ESI

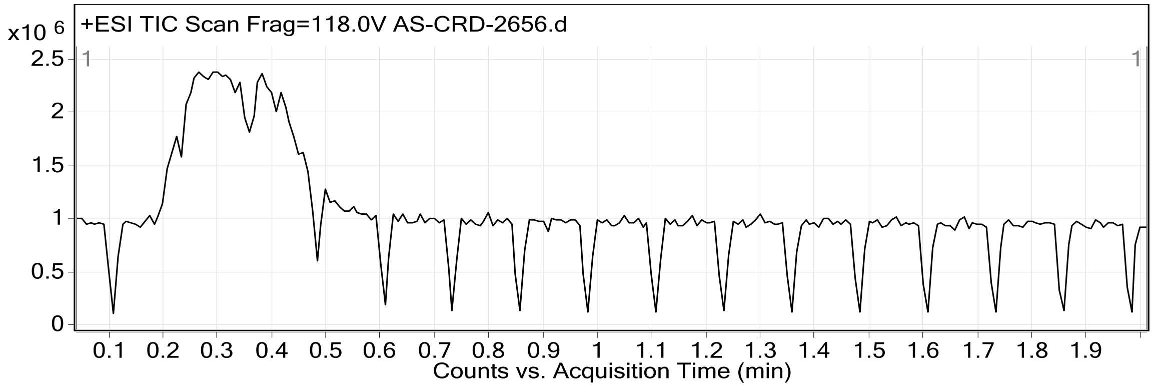

User Spectra

Fragmentor Voltage	Collision Energy	Ionization Mode
118	0	ESI

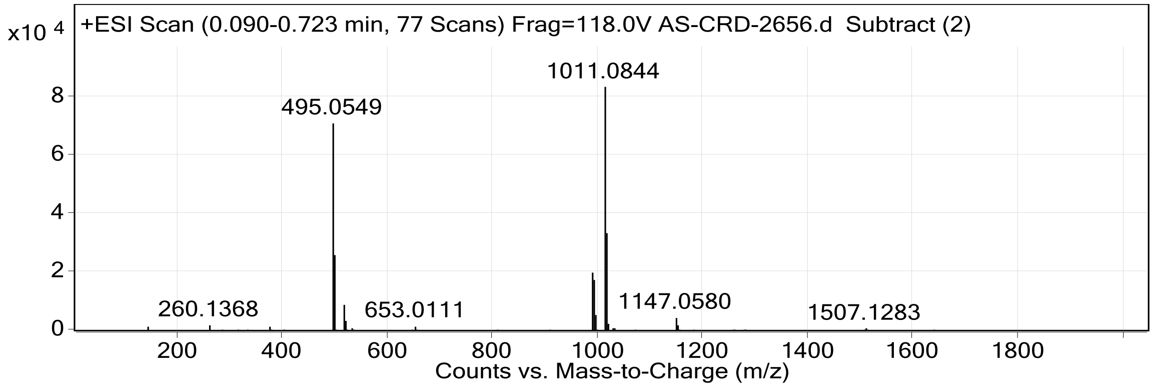

Peak List

m/z	z	Abund
495.0549	1	71253.82
496.0579	1	17502.47
497.0526	1	26163.22
989.1024	1	20095.96
991.1008	1	17365.02
1011.0844	1	83626.32

1012.0875	1	43295.54
1013.0829	1	72868.48
1014.0849	1	33505.16
1015.0818	1	21269.29

Compound 5 (9f)

Compounds

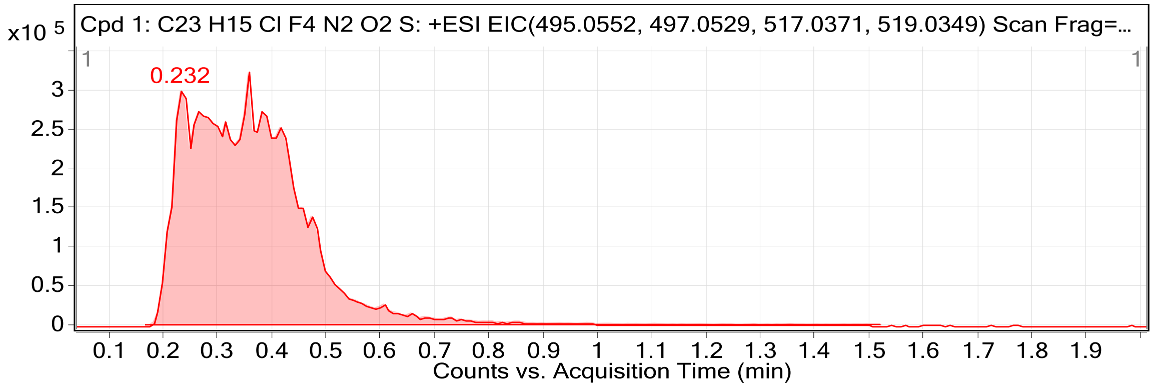

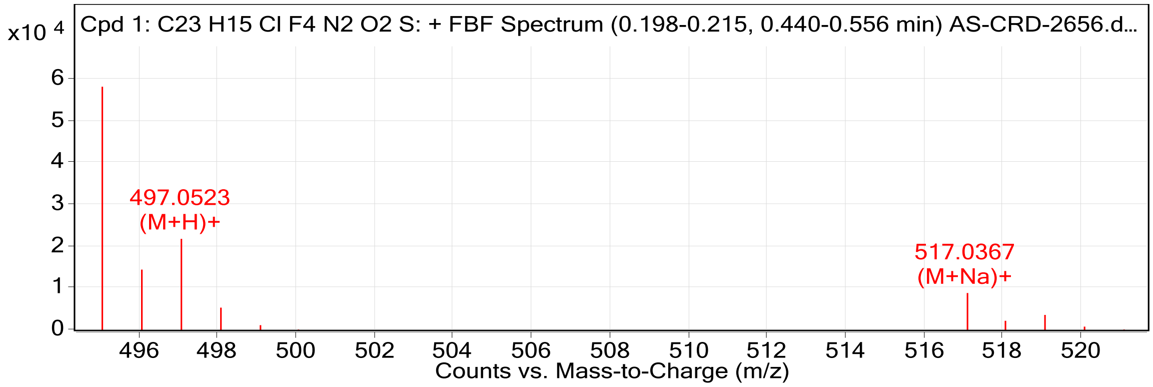

Peak List

m/z	z	Abund	Formula	Ion
495.0547	1	58302.96	C23H16ClF4N2O2S	(M+H)+
496.0577	1	14584.56	C23H16ClF4N2O2S	(M+H)+
497.0523	1	22080.4	C23H16ClF4N2O2S	(M+H)+
498.0547	1	5538.29	C23H16ClF4N2O2S	(M+H)+
499.0524	1	1499.86	C23H16ClF4N2O2S	(M+H)+
517.0367	1	9093.44	C23H15ClF4N2NaO2S	(M+Na)+
518.039	1	2514.48	C23H15ClF4N2NaO2S	(M+Na)+
519.0344	1	3713.65	C23H15ClF4N2NaO2S	(M+Na)+
520.0367	1	1054.49	C23H15ClF4N2NaO2S	(M+Na)+
521.0333	1	335.91	C23H15ClF4N2NaO2S	(M+Na)+

Compound 5 (9f)

SAMPLE INFORMATION

Sample Name:	SN-CA202-82	Acquired By:	UPLC_MS_01 System
Vial:	1:D,2	Sample Set Name:	SAMPLE_FA
Injection #:	1	Acq. Method Set:	PH HEX_FA_6min
Injection Volume:	0.50 ul	Processing Method:	MASS
Run Time:	6.0 Minutes	Channel Name:	495.2Da
Date Acquired:	23-01-2017 14:10:37 IST	Date Processed:	23-01-2017 14:52:07 IST

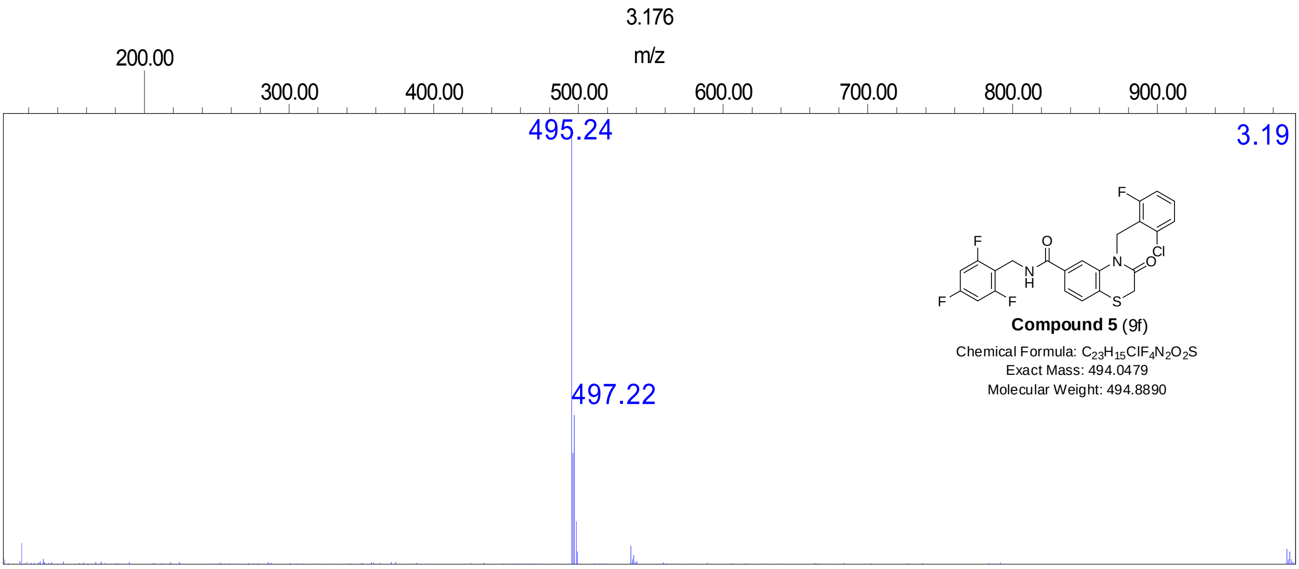

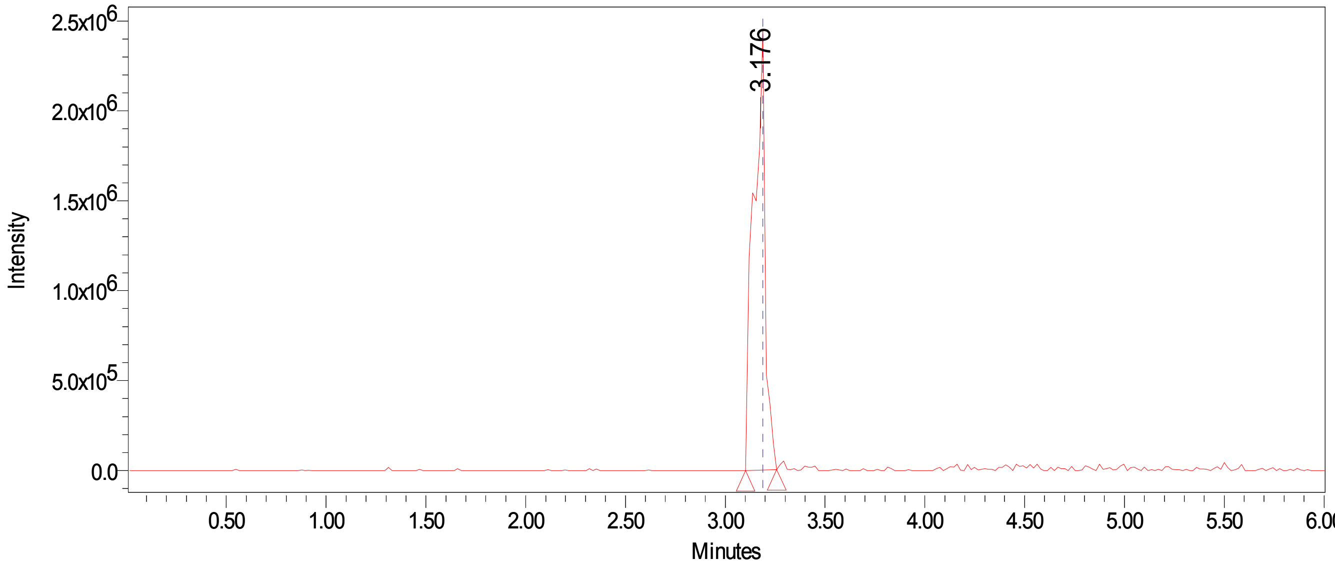

Channel Description 1: 100.00-1000.00 ES+, Centroid, CV=Tune; Processed Channel Descr. W3100 1: MS Scan MS 495.24 m/z Peak Separation: 1.0000 (1: 100.00-1000.00 ES+, Centroid, CV=Tune)

Compound 5 (9f)

SAMPLE INFORMATION

Sample Name:	SN-CA202-82	Acquired By:	UPLC_MS_01 System
Vial:	1:D,2	Sample Set Name:	SAMPLE_FA
Injection #:	1	Acq. Method Set:	PH HEX_FA_6min
Injection Volume:	0.50 ul	Processing Method:	UPLC
Run Time:	6.0 Minutes	Channel Name:	230.0nm@2
Date Acquired:	23-01-2017 14:10:37 IST	Date Processed:	23-01-2017 14:51:26 IST
Column	PHENYL HEXYL (2.1x100mm)	Mobile Phase	0.1% FA in Water/ACN

Auto-Scaled Chromatogram

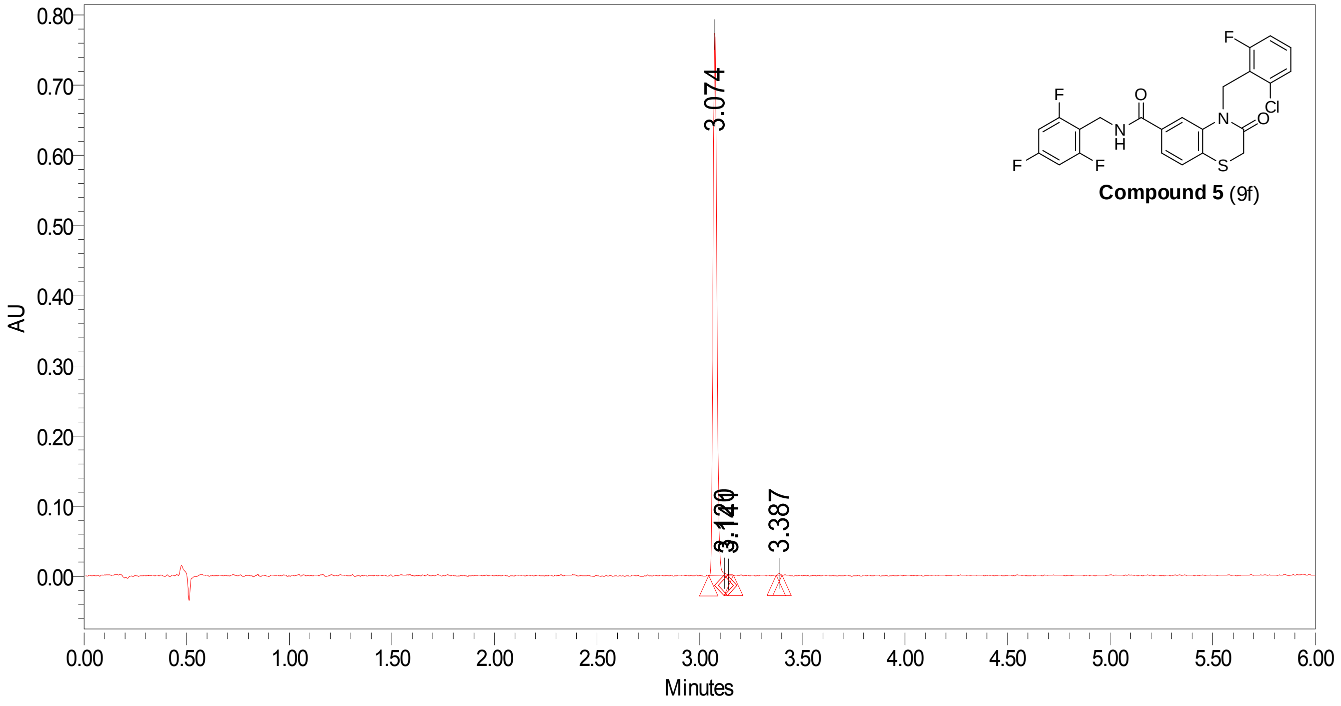

Processed Channel Descr. PDA 230.0 nm (PDA Spectrum (210-400)nm) Blank Subtracted from BLANK_FA, Vial 1:F,8 Inj. 1

Peak Results

	Name	RT	Area	% Area	Height
1		3.074	996020	99.46	773961
2		3.120	2114	0.21	2704
3		3.141	1301	0.13	1186
4		3.387	1968	0.20	2322

Compound 6 (9l)

ND-CA262-51

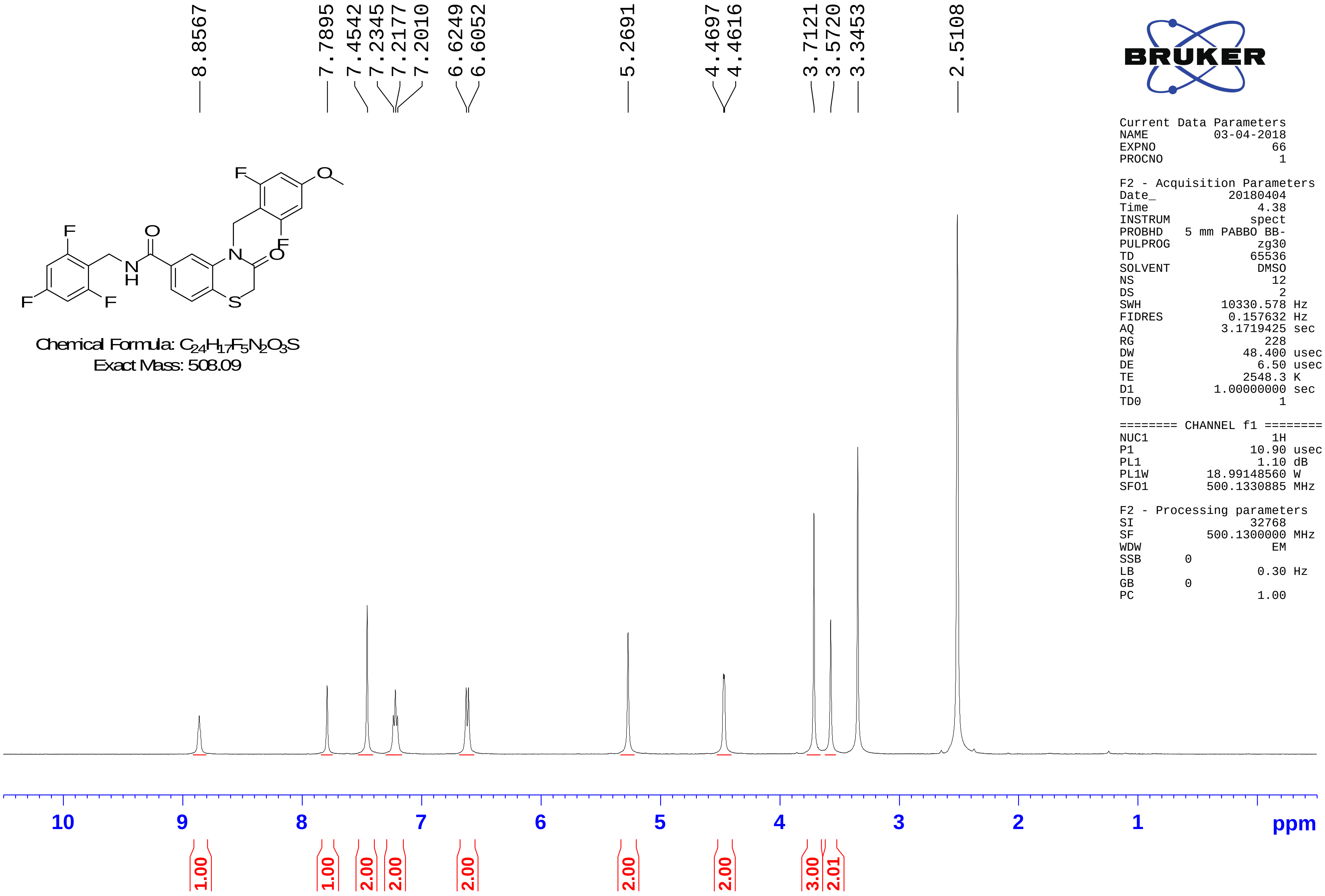


```
Current Data Parameters
NAME          03-04-2018
EXPNO                 66
PROCNO                 1

F2 - Acquisition Parameters
Date_           20180404
Time                4.38
INSTRUM            spect
PROBHD   5 mm PABBO BB-
PULPROG             zg30
TD                 65536
SOLVENT             DMSO
NS                    12
DS                     2
SWH           10330.578 Hz
FIDRES         0.157632 Hz
AQ            3.1719425 sec
RG                   228
DW                48.400 usec
DE                  6.50 usec
TE                2548.3 K
D1            1.00000000 sec
TD0                    1

======== CHANNEL f1 ========
NUC1                  1H
P1                 10.90 usec
PL1                 1.10 dB
PL1W         18.99148560 W
SFO1         500.1330885 MHz

F2 - Processing parameters
SI                 32768
SF           500.1300000 MHz
WDW                   EM
SSB      0
LB                  0.30 Hz
GB       0
PC                  1.00
```

Compound 6 (9l)

TCG Lifesciences Private Limited
Kolkata

CRD−3969 IN DMSO−13C

TCGLS/ARD/NMR02/K02

NAME	CRD−3969
EXPNO	60
PROCNO	1
Date_	20200620
Time	17.29
INSTRUM	spect
PROBHD	5 mm PABBO BB−
PULPROG	zgpg30
TD	65536
SOLVENT	DMSO
NS	25000
DS	4
SWH	25252.525 Hz
FIDRES	0.385323 Hz
AQ	1.2976629 sec
RG	2050
DW	19.800 usec
DE	6.50 usec
TE	299.3 K
D1	2.00000000 sec
D11	0.03000000 sec
TD0	1
======== CHANNEL f1 ========	
NUC1	13C
P1	6.75 usec
PL1	0.00 dB
SFO1	100.6404331 MHz
======== CHANNEL f2 ========	
CPDPRG2	waltz16
NUC2	1H
PCPD2	80.00 usec
PL2	0.00 dB
PL12	13.45 dB
PL13	17.00 dB
SFO2	400.2016008 MHz
SI	32768
SF	100.6304307 MHz
WDW	EM
SSB	0
LB	1.00 Hz
GB	0
PC	1.40

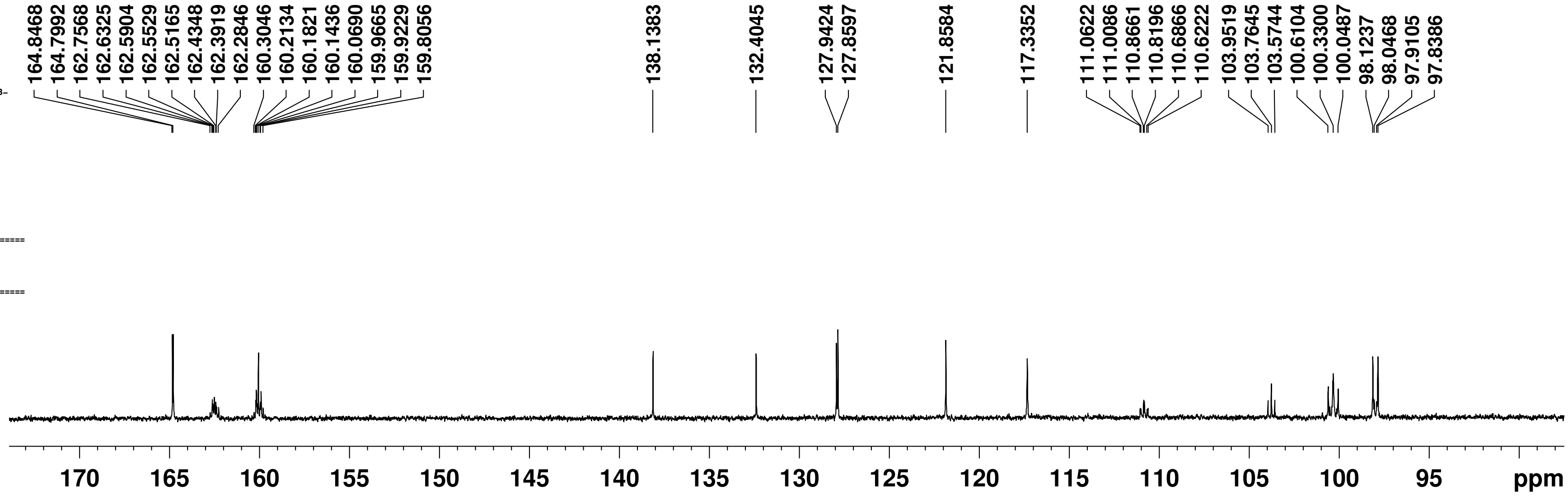

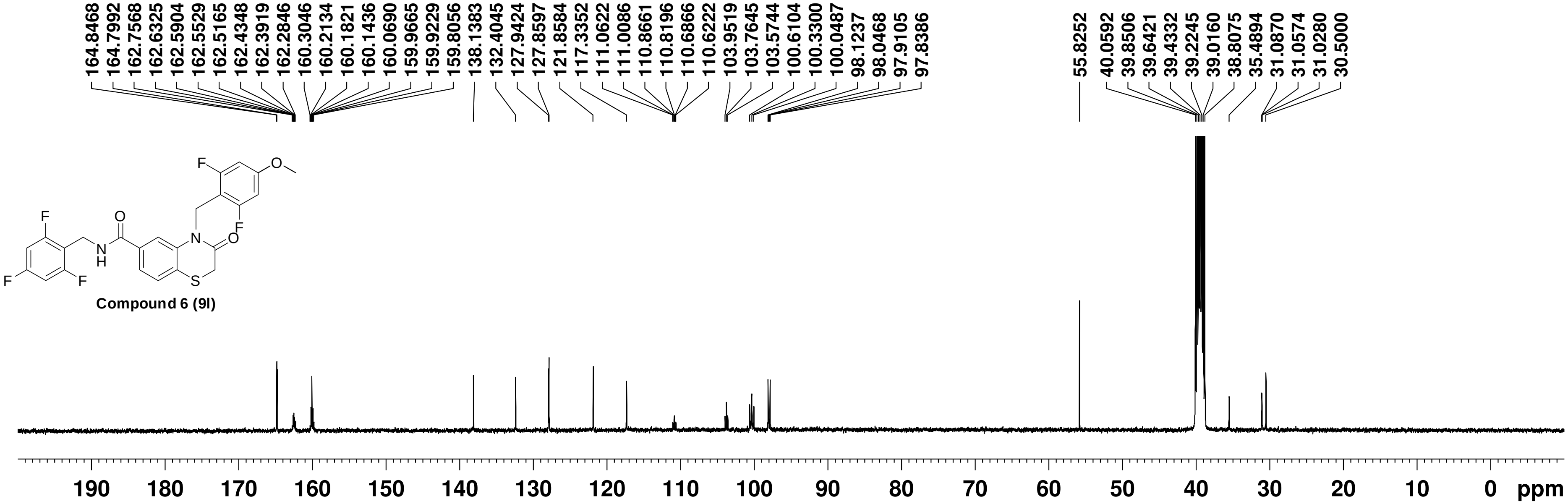

Compound 6 (9l)

Compound 6 (9l)

TCG Lifesciences Private Limited
Kolkata

CRD−3969 IN DMSO−APT

TCGLS/ARD/NMR02/K02

NAME	CRD−3969
EXPNO	61
PROCNO	1
Date_	20200622
Time	1.51
INSTRUM	spect
PROBHD	5 mm PABBO BB−
PULPROG	jmod
TD	65536
SOLVENT	DMSO
NS	10000
DS	4
SWH	25252.525 Hz
FIDRES	0.385323 Hz
AQ	1.2976629 sec
RG	2050
DW	19.800 usec
DE	6.50 usec
TE	299.4 K
CNST2	145.0000000
CNST11	1.0000000
D1	2.00000000 sec
D20	0.00689655 sec
TD0	1

======== CHANNEL f1 ========

NUC1	13C
P1	6.75 usec
P2	13.50 usec
PL1	0.00 dB
SFO1	100.6404331 MHz

======== CHANNEL f2 ========

CPDPRG2	waltz16
NUC2	1H
PCPD2	80.00 usec
PL2	0.00 dB
PL12	13.45 dB
SFO2	400.2016008 MHz
SI	32768
SF	100.6304315 MHz
WDW	EM
SSB	0
LB	1.00 Hz
GB	0
PC	1.40

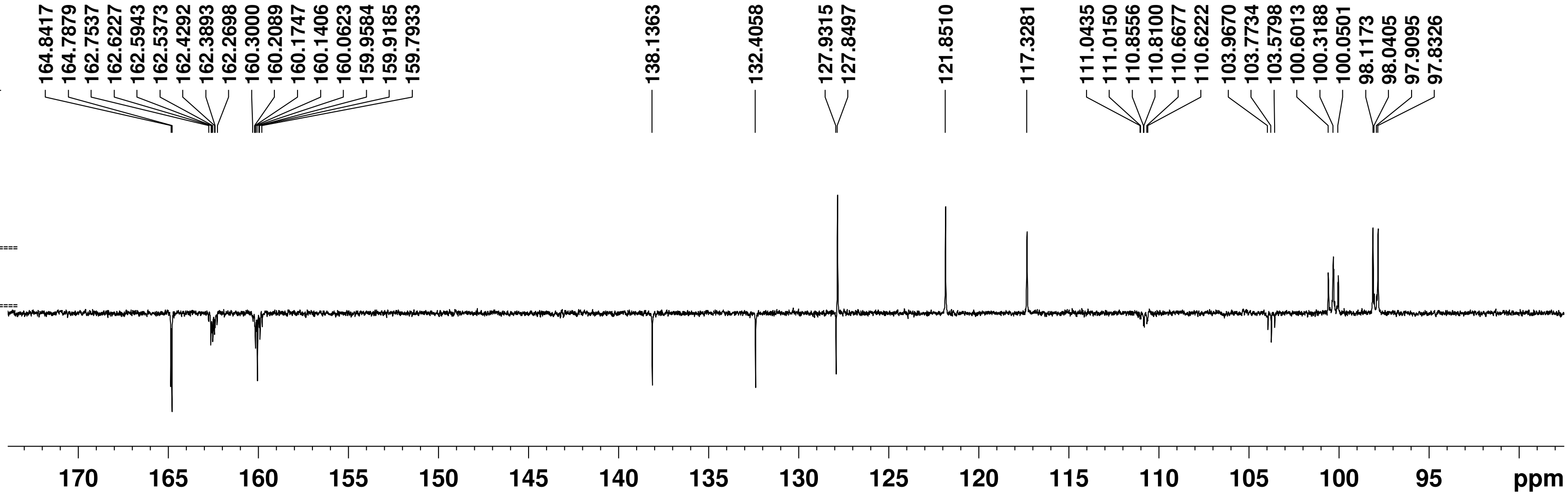

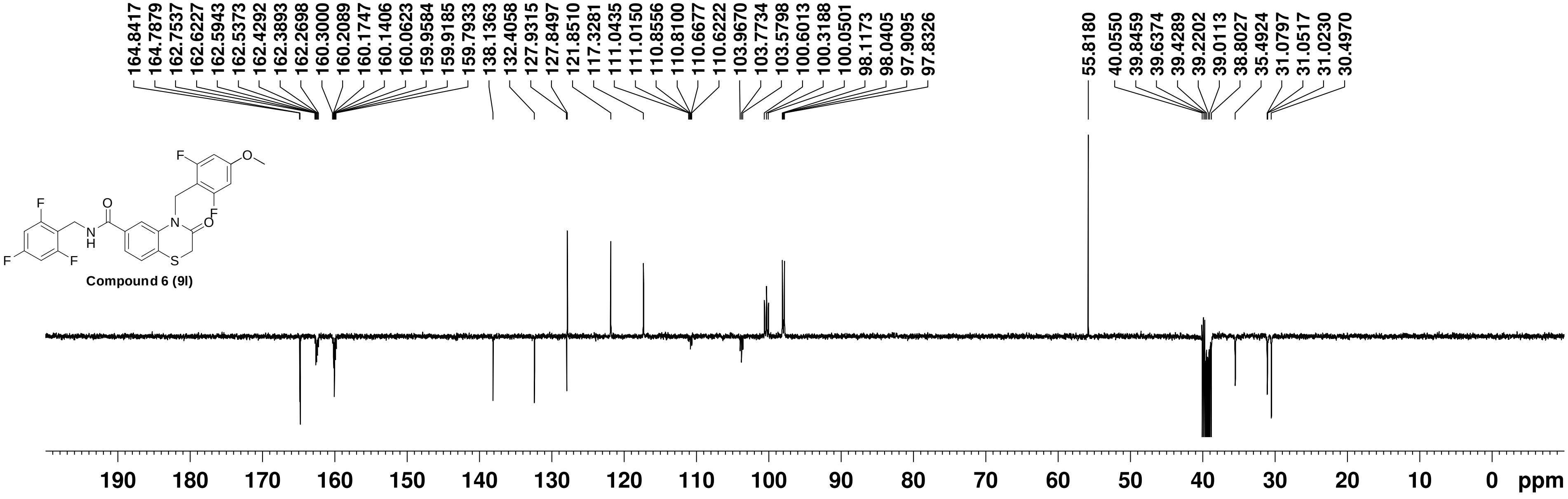

Compound 6 (9l)

Qualitative Analysis Report

Compound 6 (9l)

**Data Filename**	AS-CRD-3969.d	**Sample Name**	AS-CRD-3969
**Sample Type**	Sample	**Position**	Vial 69
**Instrument Name**	Instrument 1	**User Name**	
**Acq Method**	Direct Mass-2017.m	**Acquired Time**	6/16/2020 12:27:55 PM
**IRM Calibration Status**	Some Ions Missed	**DA Method**	Default.m
**Comment**			

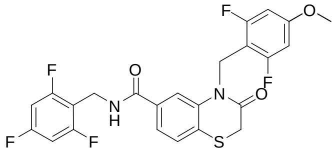

Compound 6 (9l)

Chemical Formula: $C_{24}H_{17}F_5N_2O_3S$

Exact Mass: 508.0880

**Sample Group**		**Info.**
**Acquisition SW Version**	6200 series TOF/6500 series Q-TOF B.05.00 (B5042.0)	

User Chromatograms

Fragmentor Voltage 118 **Collision Energy** 0 **Ionization Mode** ESI

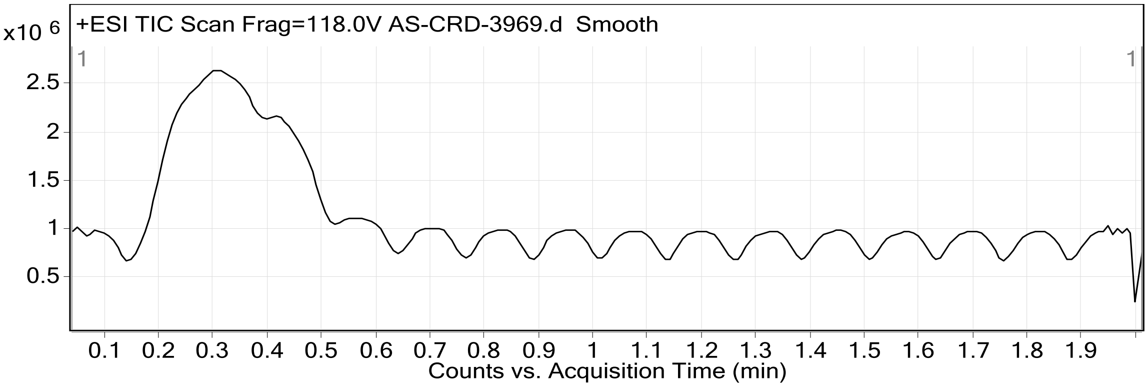

User Spectra

Fragmentor Voltage	Collision Energy	Ionization Mode
118	0	ESI

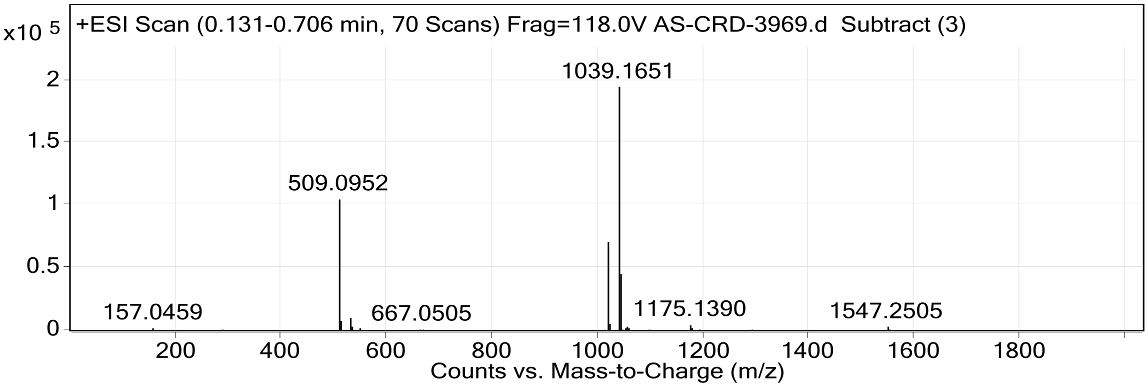

Peak List

m/z	z	Abund
509.0952	1	105298
510.098	1	26787.38
531.0768	1	10680.39
1017.1827	1	71631.29
1018.1857	1	39153.14
1019.1851	1	17428.08

1039.1651	1	194971.7
1040.1677	1	103962.05
1041.1671	1	46054.39
1042.1672	1	15300.82

Compound 6 (9l)

Compounds

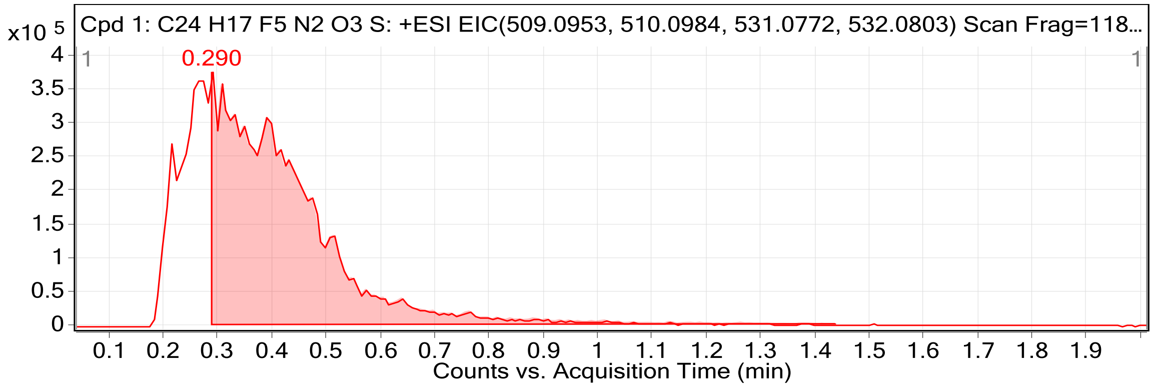

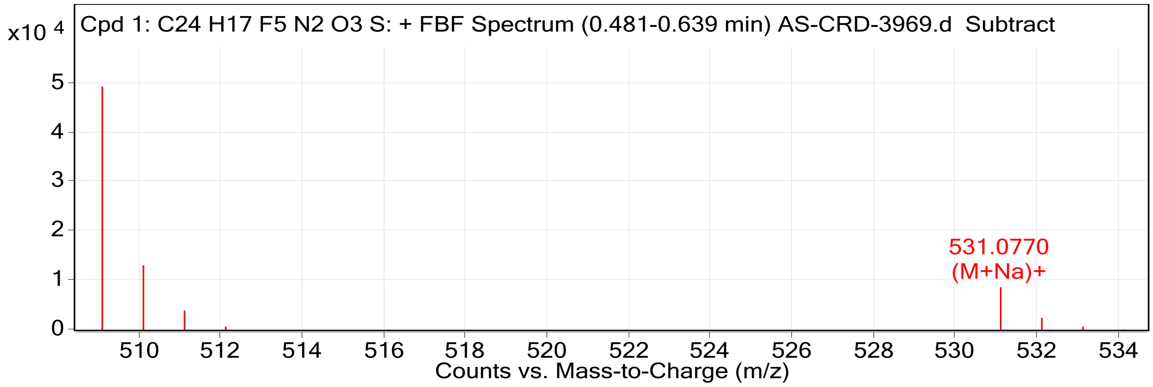

Peak List

m/z	z	Abund	Formula	Ion
509.0951	1	49411.3	C24H18F5N2O3S	(M+H)+
510.0979	1	13258.35	C24H18F5N2O3S	(M+H)+
511.0956	1	4078.82	C24H18F5N2O3S	(M+H)+
512.0965	1	849.94	C24H18F5N2O3S	(M+H)+
513.1003	1	129.74	C24H18F5N2O3S	(M+H)+
531.077	1	8730.64	C24H17F5N2NaO3S	(M+Na)+
532.0793	1	2595.69	C24H17F5N2NaO3S	(M+Na)+
533.078	1	819.23	C24H17F5N2NaO3S	(M+Na)+
534.0799	1	148.96	C24H17F5N2NaO3S	(M+Na)+

Compound 6 (9l)

SAMPLE INFORMATION

Sample Name:	ND-CA262-51	Acquired By:	UPLC_MS_01 System
Vial:	1:A,2	Sample Set Name:	AA
Injection #:	1	Acq. Method Set:	AA_C18_6min_N
Injection Volume:	0.50 ul	Processing Method:	MASS
Run Time:	6.0 Minutes	Channel Name:	509.2Da@5
Date Acquired:	31-03-2018 18:40:56 IST	Date Processed:	02-04-2018 14:59:02 IST

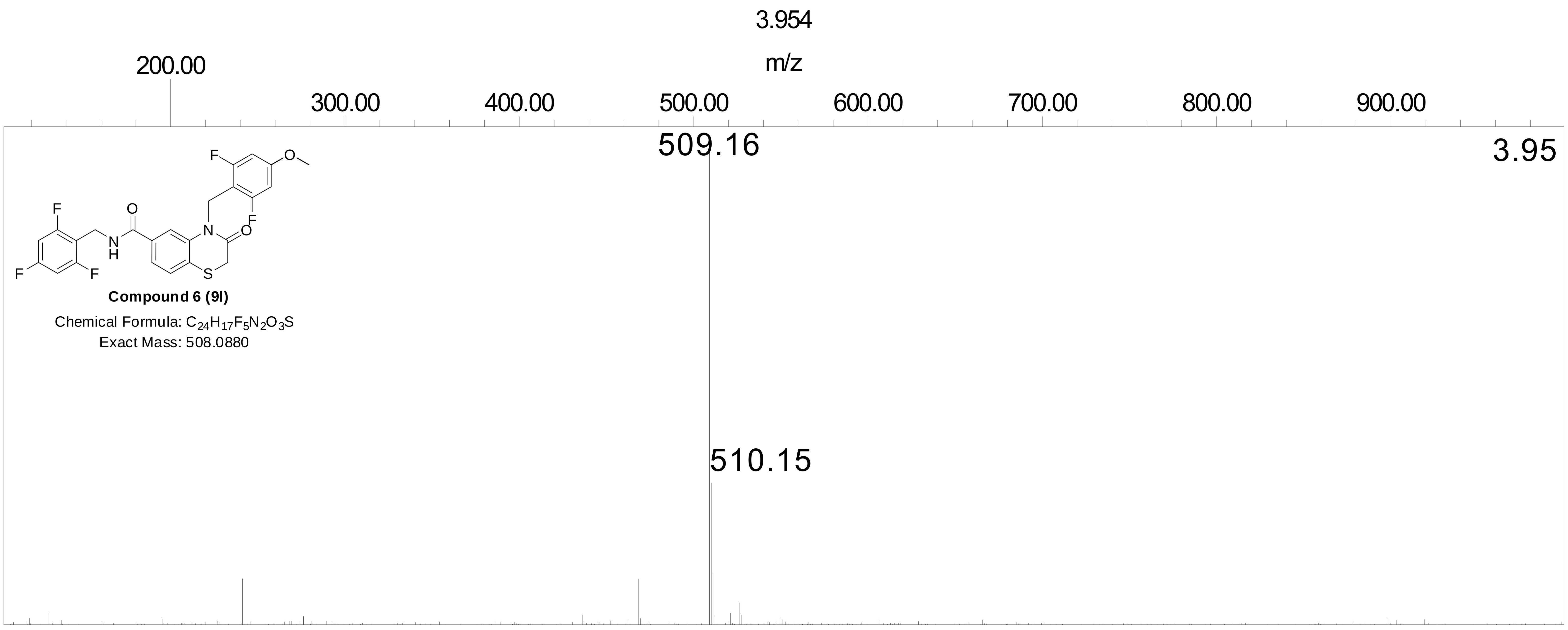

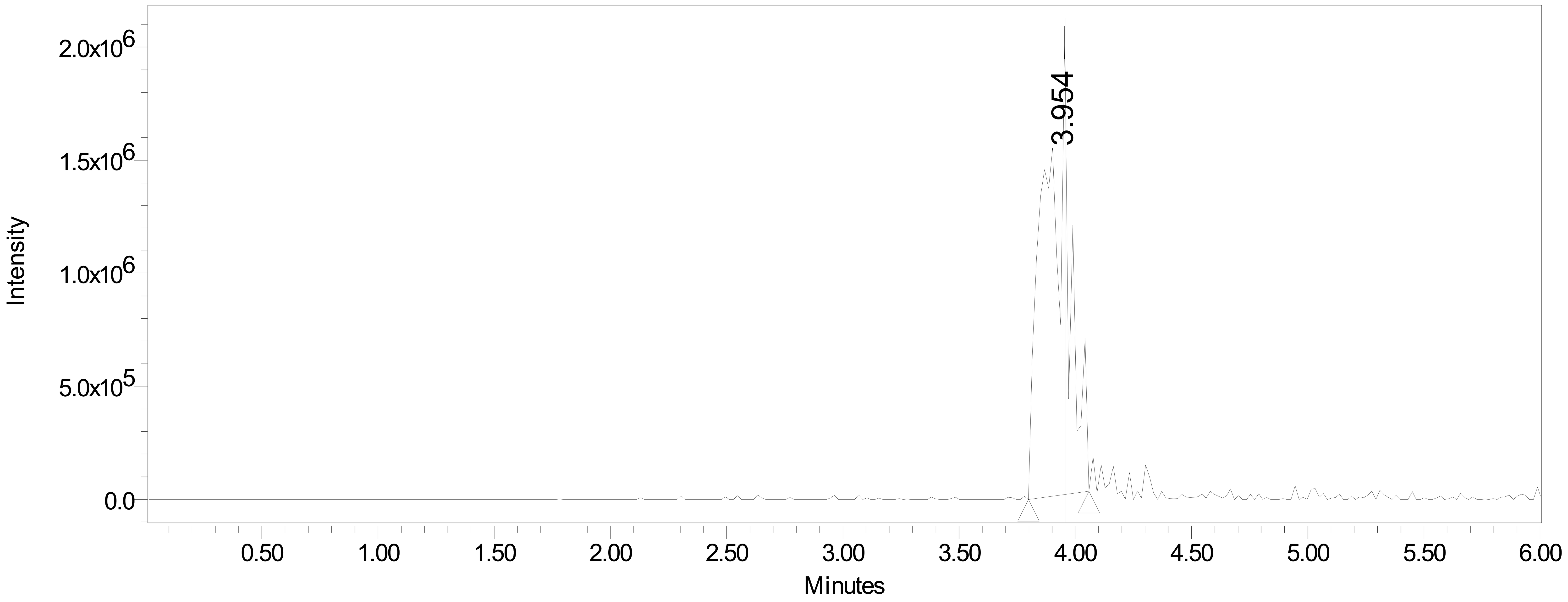

Channel Description 1: 100.00-1000.00 ES+, Centroid, CV=Tune; Processed Channel Descr. W3100 1: MS Scan MS 509.16 m/z Peak Separation: 1.0000 (1: 100.00-1000.00 ES+, Centroid, CV=Tune)

Compound 6 (9l)

SAMPLE INFORMATION

Sample Name:	ND-CA262-51	Acquired By:	UPLC_MS_01 System
Vial:	1:A,2	Sample Set Name:	AA
Injection #:	1	Acq. Method Set:	AA_C18_6min_N
Injection Volume:	0.50 ul	Processing Method:	UPLC
Run Time:	6.0 Minutes	Channel Name:	240.0nm
Date Acquired:	31-03-2018 18:40:56 IST	Date Processed:	02-04-2018 14:57:34 IST
Column	KINETEX_EVO_C-18	Mobile Phase	1mM A.A. in Water/ACN

Auto-Scaled Chromatogram

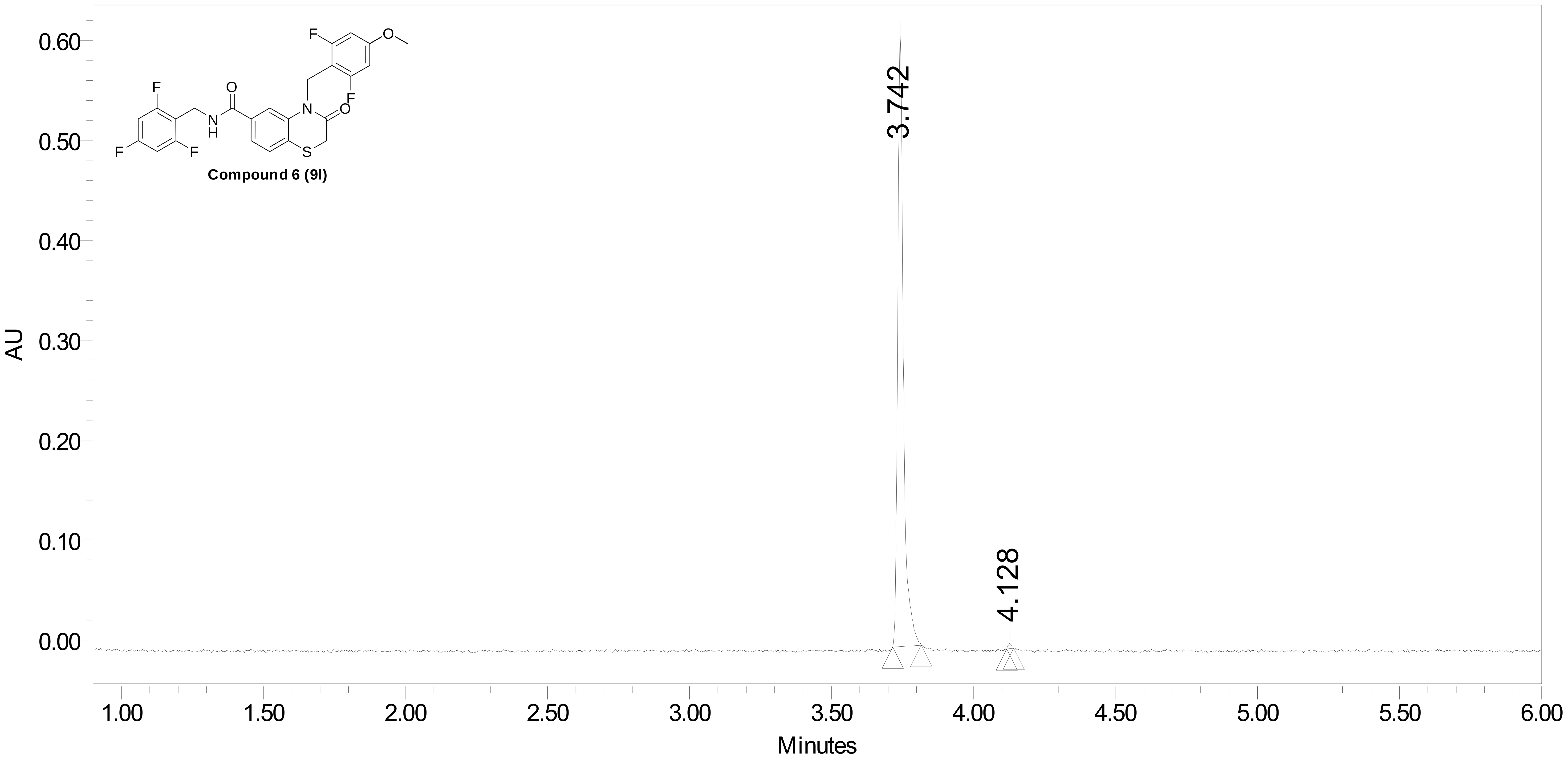

Processed Channel Descr. PDA 240.0 nm (PDA Spectrum (210-400)nm) Blank Subtracted from BLANK, Vial 1:F,8 Inj. 1

Peak Results

	Name	RT	Area	% Area	Height
1		3.742	866033	99.53	610893
2		4.128	4053	0.47	5329

Compound 7 (10a)

ND-CA262-53

Chemical Formula: $C_{23}H_{15}F_5N_2O_3S$

Exact Mass: 494.07

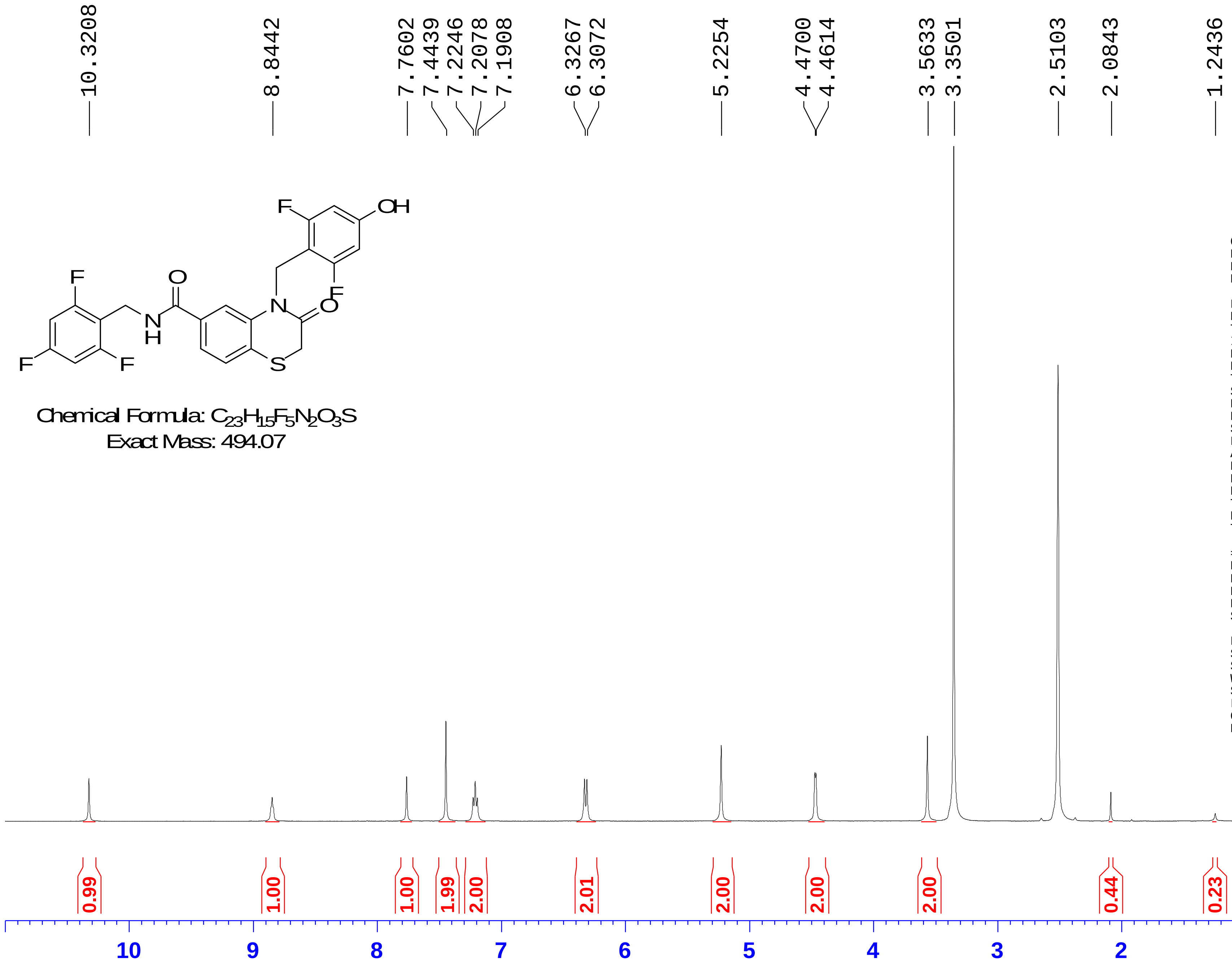


```
Current Data Parameters
NAME              03-04-2018
EXPNO                     67
PROCNO                     1

F2 - Acquisition Parameters
Date_               20180404
Time                    4.41
INSTRUM                spect
PROBHD     5 mm PABBO BB-
PULPROG                 zg30
TD                     65536
SOLVENT                 DMSO
NS                        24
DS                         2
SWH              10330.578 Hz
FIDRES            0.157632 Hz
AQ               3.1719425 sec
RG                       228
DW                    48.400 usec
DE                      6.50 usec
TE                    2719.5 K
D1              1.00000000 sec
TD0                        1

======== CHANNEL f1 ========
NUC1                      1H
P1                     10.90 usec
PL1                     1.10 dB
PL1W             18.99148560 W
SFO1             500.1330885 MHz

F2 - Processing parameters
SI                     32768
SF               500.1300000 MHz
WDW                       EM
SSB      0
LB                      0.30 Hz
GB       0
PC                      1.00
```

Compound 7 (10a)

TCG Lifesciences Private Limited

Kolkata

NAME	CRD–3970
EXPNO	60
PROCNO	1
Date_	20200613
Time	18.22
INSTRUM	spect
PROBHD	5 mm PABBO BB–
PULPROG	zgpg30
TD	65536
SOLVENT	DMSO
NS	30000
DS	4
SWH	25252.525 Hz
FIDRES	0.385323 Hz
AQ	1.2976629 sec
RG	2050
DW	19.800 usec
DE	6.50 usec
TE	299.9 K
D1	2.00000000 sec
D11	0.03000000 sec
TD0	1
======== CHANNEL f1 ========	
NUC1	13C
P1	6.75 usec
PL1	0.00 dB
SFO1	100.6404331 MHz
======== CHANNEL f2 ========	
CPDPRG2	waltz16
NUC2	1H
PCPD2	80.00 usec
PL2	0.00 dB
PL12	13.45 dB
PL13	17.00 dB
SFO2	400.2016008 MHz
SI	32768
SF	100.6304299 MHz
WDW	EM
SSB	0
LB	1.00 Hz
GB	0
PC	1.40

CRD–3970 IN DMSO–13C

TCGLS/ARD/NMR02/K02

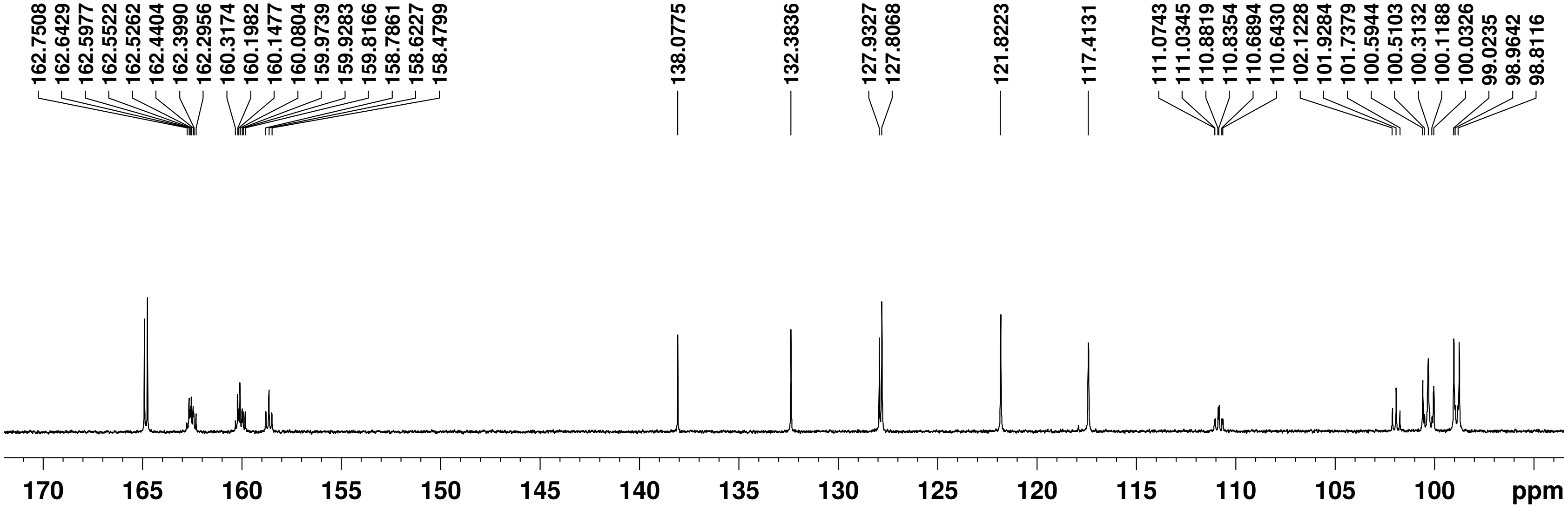

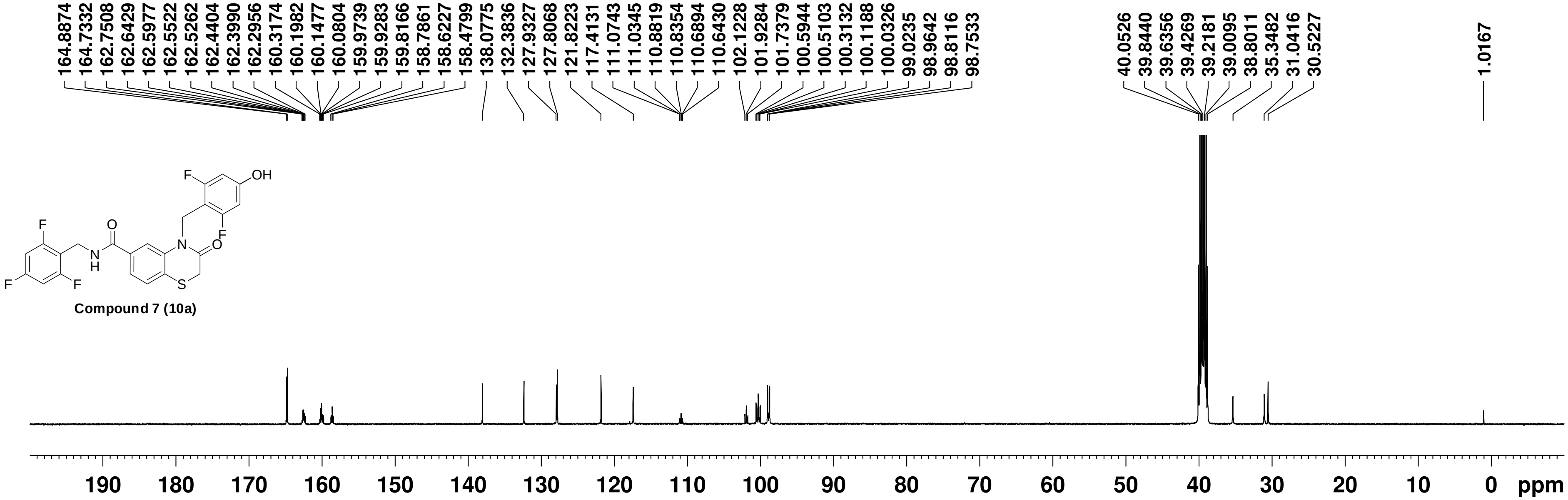

Compound 7 (10a)

Compound 7 (10a)

TCG Lifesciences Private Limited
Kolkata

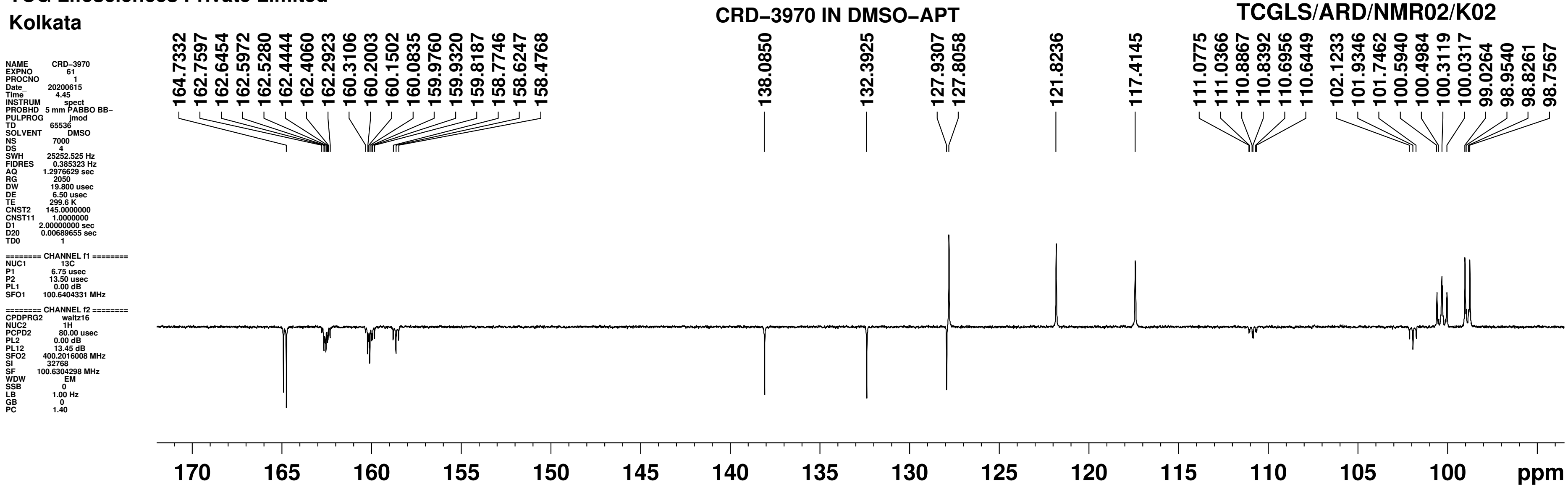

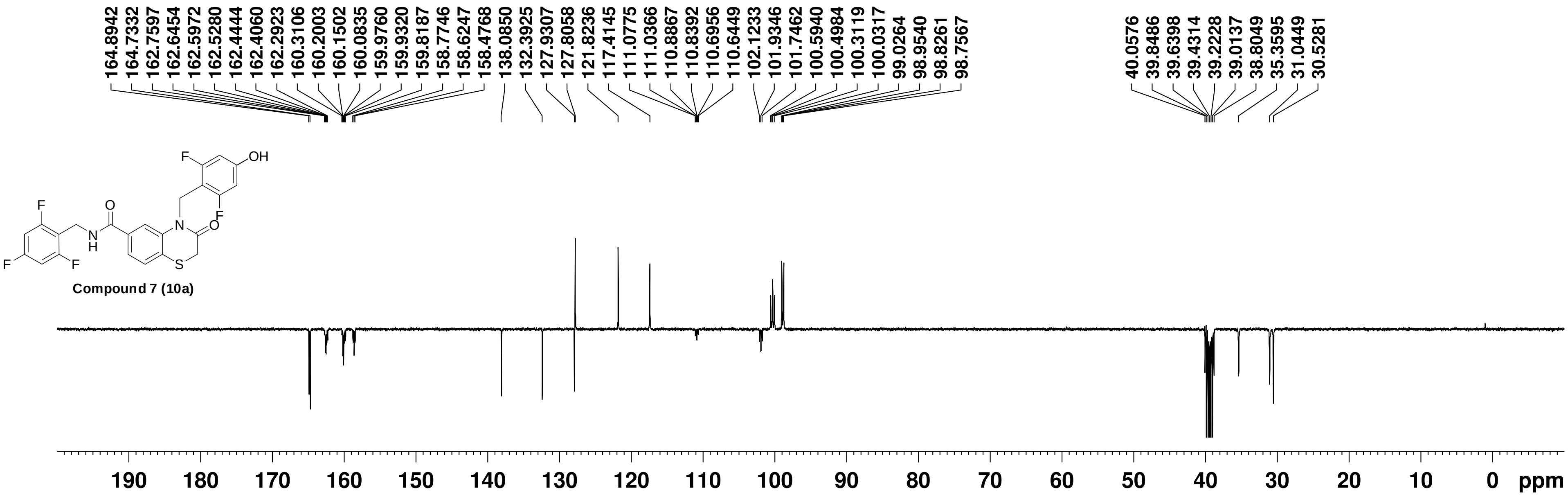

Compound 7 (10a)

Qualitative Analysis Report

Compound 7 (10a)

**Data Filename**	AS-CRD-3970.d	**Sample Name**	AS-CRD-3970
**Sample Type**	Sample	**Position**	Vial 71
**Instrument Name**	Instrument 1	**User Name**	
**Acq Method**	Direct Mass-2017.m	**Acquired Time**	6/16/2020 12:31:19 PM
**IRM Calibration Status**	Some Ions Missed	**DA Method**	Default.m
**Comment**			

Sample Group **Info.**

Acquisition SW Version 6200 series TOF/6500 series Q-TOF B.05.00 (B5042.0)

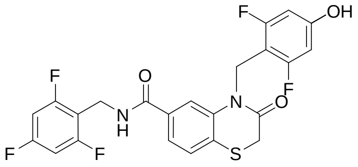

Compound 7 (10a)

Chemical Formula: $C_{23}H_{15}F_5N_2O_3S$

Exact Mass: 494.0724

User Chromatograms

Fragmentor Voltage 118 **Collision Energy** 0 **Ionization Mode** ESI

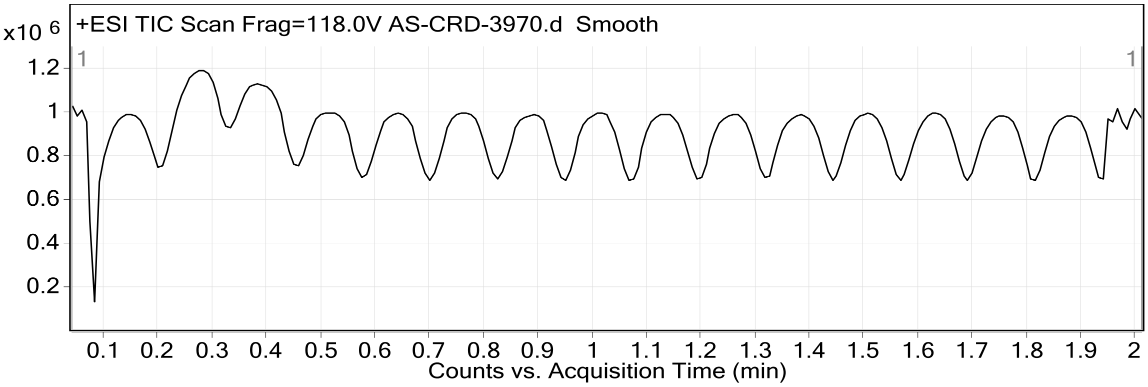

User Spectra

Fragmentor Voltage	Collision Energy	Ionization Mode
118	0	ESI

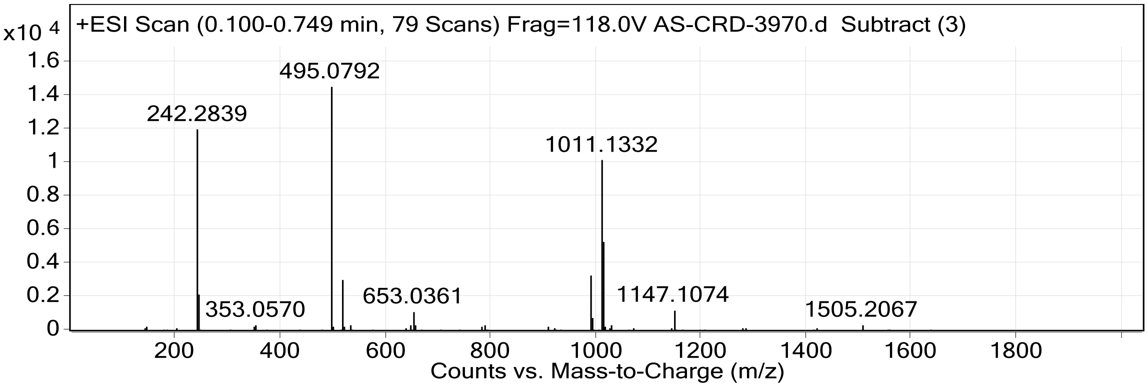

Peak List

m/z	z	Abund
242.2839	1	11973.97
243.287	1	2192.73
495.0792	1	14519.7
496.0824	1	3918.08
517.0612	1	3037.6
989.1512	1	3269.83

990.1536	1	1747.73
1011.1332	1	10137.59
1012.1361	1	5326.68
1013.1355	1	2485.3

Compound 7 (10a)

Compounds

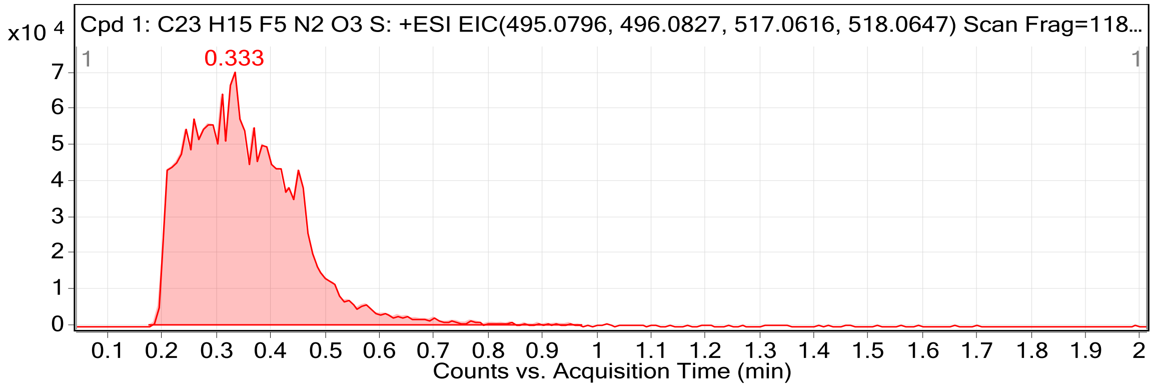

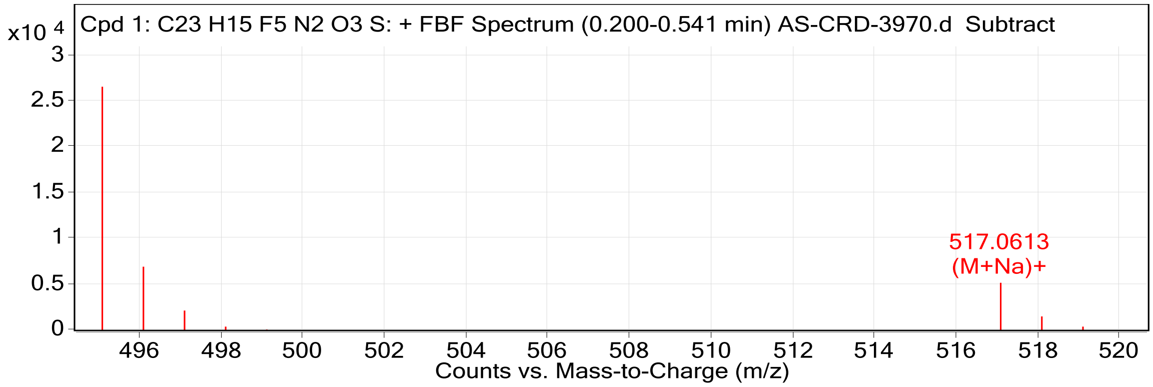

Peak List

m/z	z	Abund	Formula	Ion
495.0792	1	26647.47	C23H16F5N2O3S	(M+H)+
496.0824	1	7069.1	C23H16F5N2O3S	(M+H)+
497.0804	1	2270.76	C23H16F5N2O3S	(M+H)+
498.0797	1	462.33	C23H16F5N2O3S	(M+H)+
499.0778	1	81.46	C23H16F5N2O3S	(M+H)+
517.0613	1	5268.43	C23H15F5N2NaO3S	(M+Na)+
518.0636	1	1526.35	C23H15F5N2NaO3S	(M+Na)+
519.0612	1	461.82	C23H15F5N2NaO3S	(M+Na)+
520.0592	1	51.72	C23H15F5N2NaO3S	(M+Na)+

MASS

Compound 7 (10a)

SAMPLE INFORMATION

Sample Name:	ND-CA262-53	Acquired By:	UPLC_MS_01 System
Vial:	1:B,4	Sample Set Name:	SAMPLE_FA
Injection #:	1	Acq. Method Set:	FA_C18_6min_N
Injection Volume:	1.20 ul	Processing Method:	MASS
Run Time:	6.0 Minutes	Channel Name:	495.2Da
Date Acquired:	03-04-2018 12:21:22 IST	Date Processed:	03-04-2018 18:20:39 IST

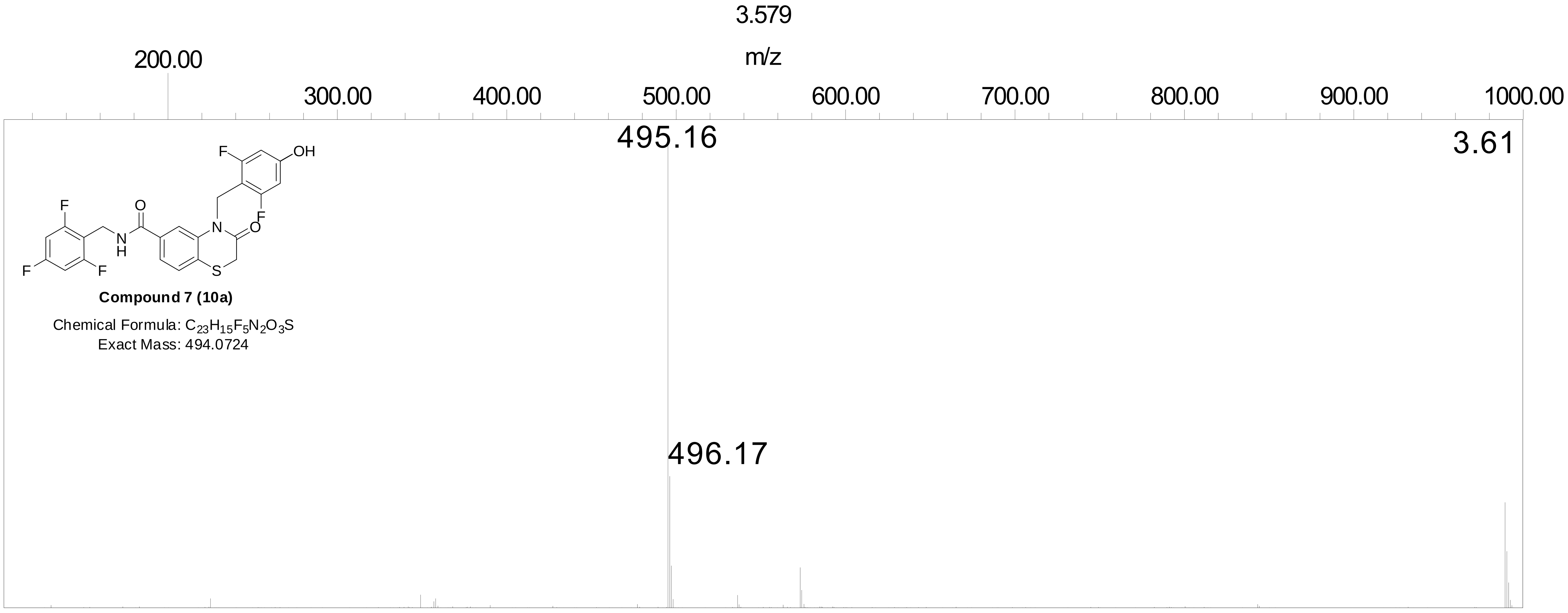

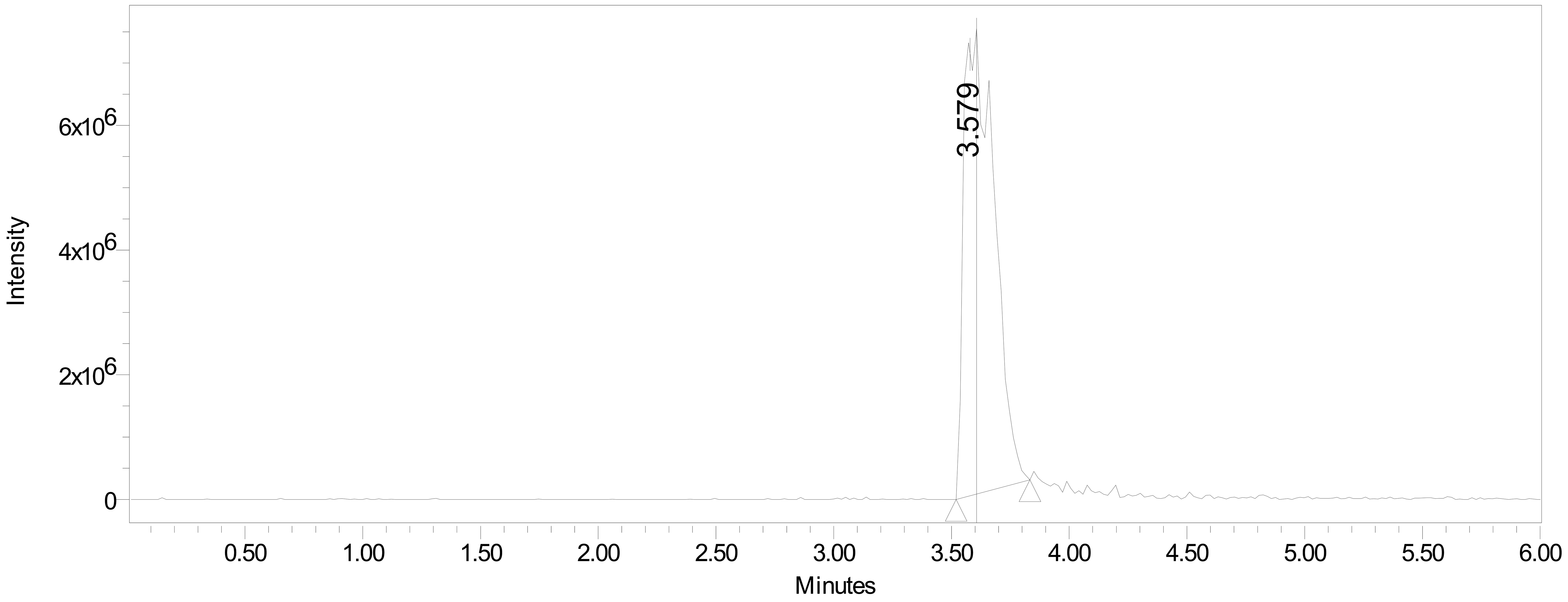

Channel Description 1: 100.00-1000.00 ES+, Centroid, CV=Tune; Processed Channel Descr. W3100 1: MS Scan MS 495.17 m/z Peak Separation: 1.0000 (1: 100.00-1000.00 ES+, Centroid, CV=Tune)

Empower 3 SOFTWARE

Compound 7 (10a)

SAMPLE INFORMATION

Sample Name:	ND-CA262-53	Acquired By:	UPLC_MS_01 System
Vial:	1:B,4	Sample Set Name:	SAMPLE_FA
Injection #:	1	Acq. Method Set:	FA_C18_6min_N
Injection Volume:	1.20 ul	Processing Method:	UPLC
Run Time:	6.0 Minutes	Channel Name:	235.0nm@2
Date Acquired:	03-04-2018 12:21:22 IST	Date Processed:	03-04-2018 18:19:59 IST
Column	KINETEX_EVO_C-18	Mobile Phase	0.1% FA in Water/ACN

Auto-Scaled Chromatogram

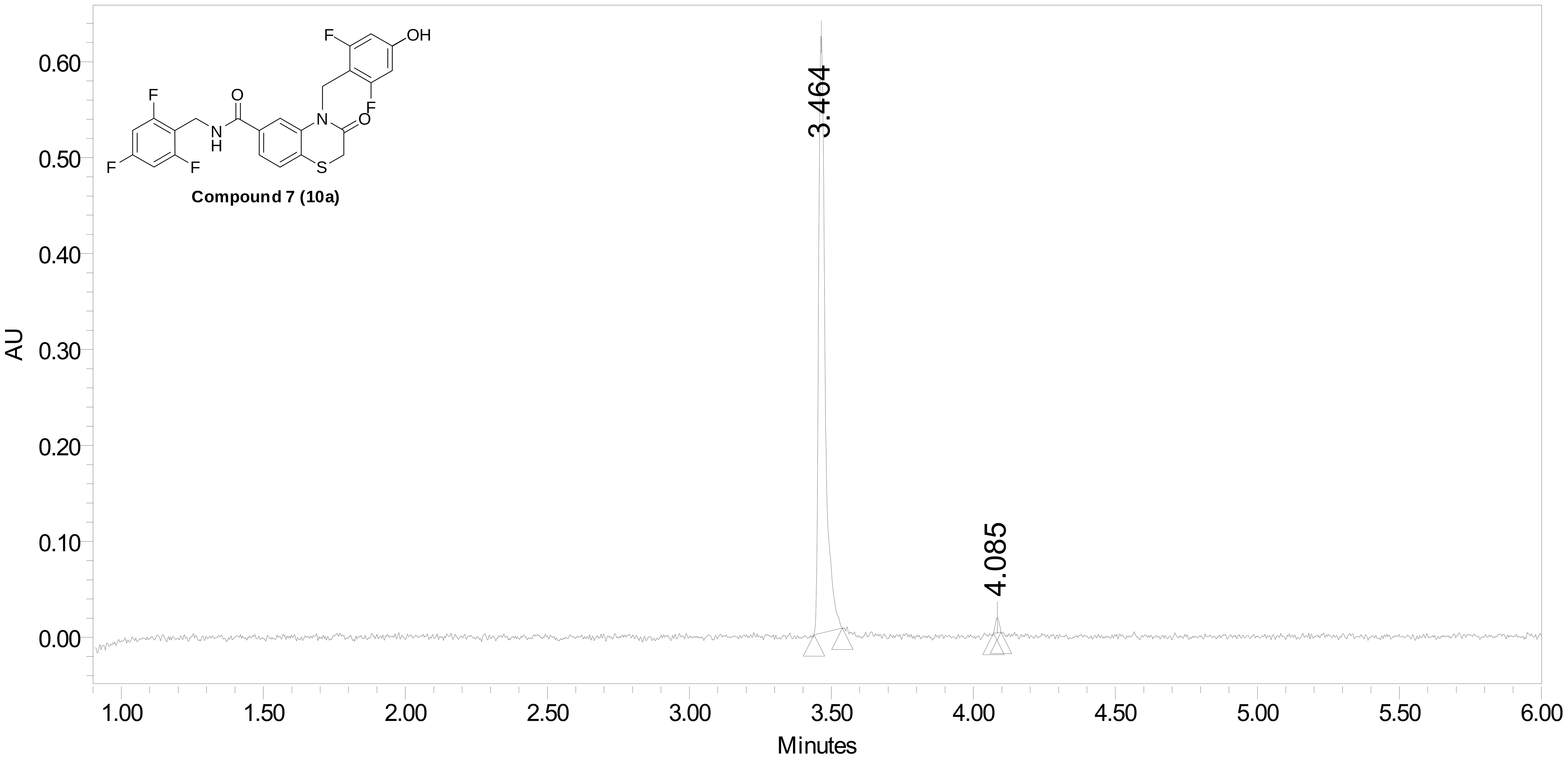

Processed Channel Descr. PDA 235.0 nm (PDA Spectrum (210-400)nm) Blank Subtracted from BLANK_DMSO, Vial 1:F,7 Inj. 1

Peak Results

	Name	RT	Area	% Area	Height
1		3.464	1002054	98.53	622378
2		4.085	14931	1.47	15655

Compound 8 (9g)

GB-CA218-140

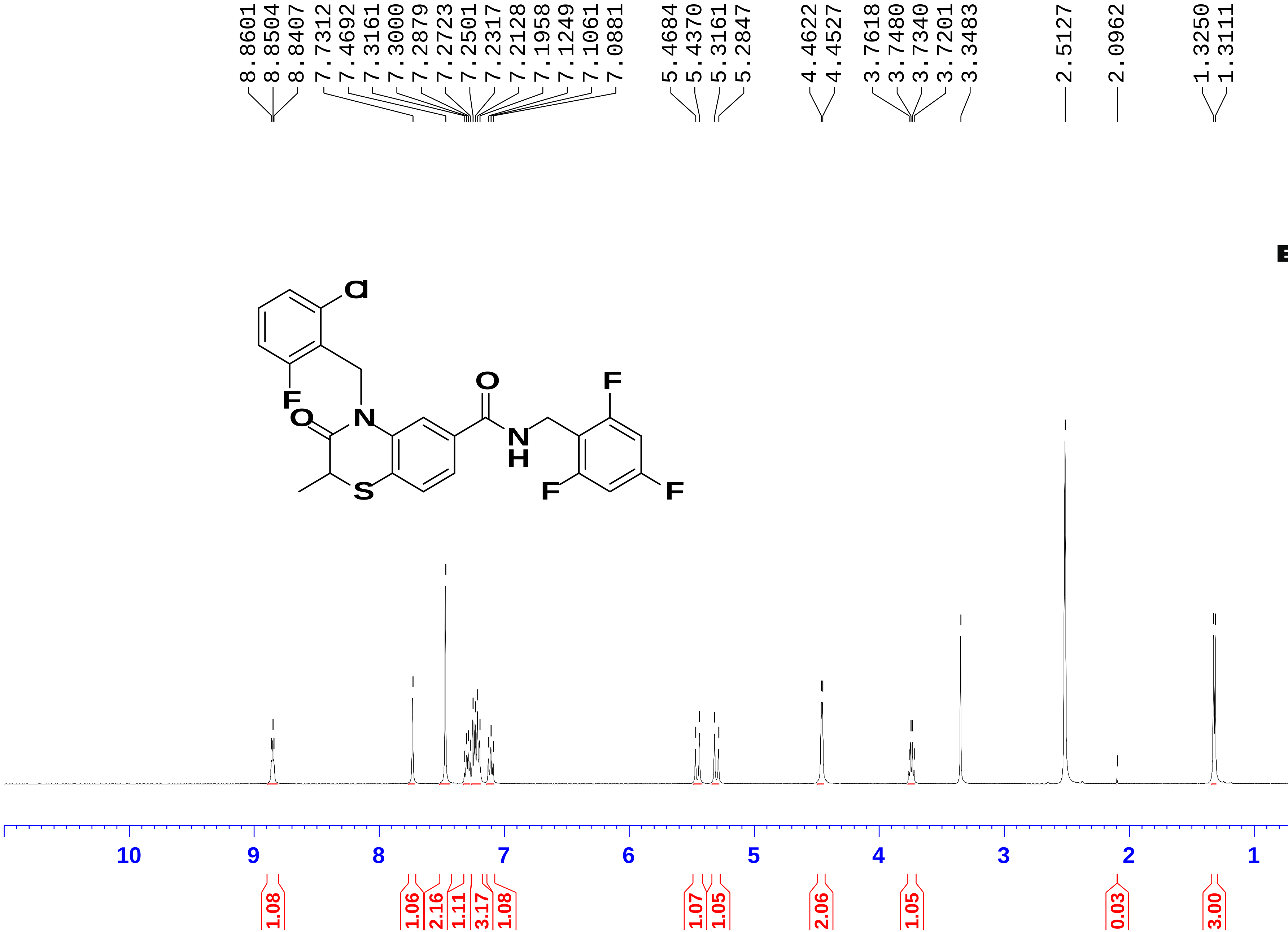


```
Current Data Parameters
NAME              826-33
EXPNO                826
PROCNO                 1

F2 - Acquisition Parameters
Date_           20170422
Time                4.35
INSTRUM            spect
PROBHD   5 mm PABBO BB-
PULPROG             zg30
TD                 65536
SOLVENT             DMSO
NS                    12
DS                     2
SWH            10330.578 Hz
FIDRES          0.157632 Hz
AQ             3.1719425 sec
RG                   181
DW                48.400 usec
DE                  6.50 usec
TE                 367.9 K
D1            1.00000000 sec
TD0                    1

======== CHANNEL f1 ========
NUC1                  1H
P1                 10.90 usec
PL1                 1.10 dB
PL1W         18.99148560 W
SFO1         500.1330885 MHz

F2 - Processing parameters
SI                 32768
SF           500.1300000 MHz
WDW                   EM
SSB      0
LB                  0.30 Hz
GB       0
PC                  1.00
```

Compound 8 (9g)

TCG Lifesciences Private Limited
Kolkata

NAME	CRD−2830
EXPNO	60
PROCNO	1
Date_	20200628
Time	9.12 h
INSTRUM	spect
PROBHD	Z8246_0048 (PH
PULPROG	zgpg30
TD	32768
SOLVENT	DMSO
NS	20000
DS	2
SWH	25252.525 Hz
FIDRES	1.541292 Hz
AQ	0.6488564 sec
RG	64
DW	19.800 usec
DE	6.50 usec
TE	297.2 K
D1	2.00000000 sec
D11	0.03000000 sec
TD0	1
SFO1	100.6152855 MHz
NUC1	13C
P1	9.10 usec
SI	16384
SF	100.6052822 MHz
WDW	EM
SSB	0
LB	1.00 Hz
GB	0
PC	1.40

CRD−2830 IN DMSO−13C

TCGLS/ARD/NMR02/K02

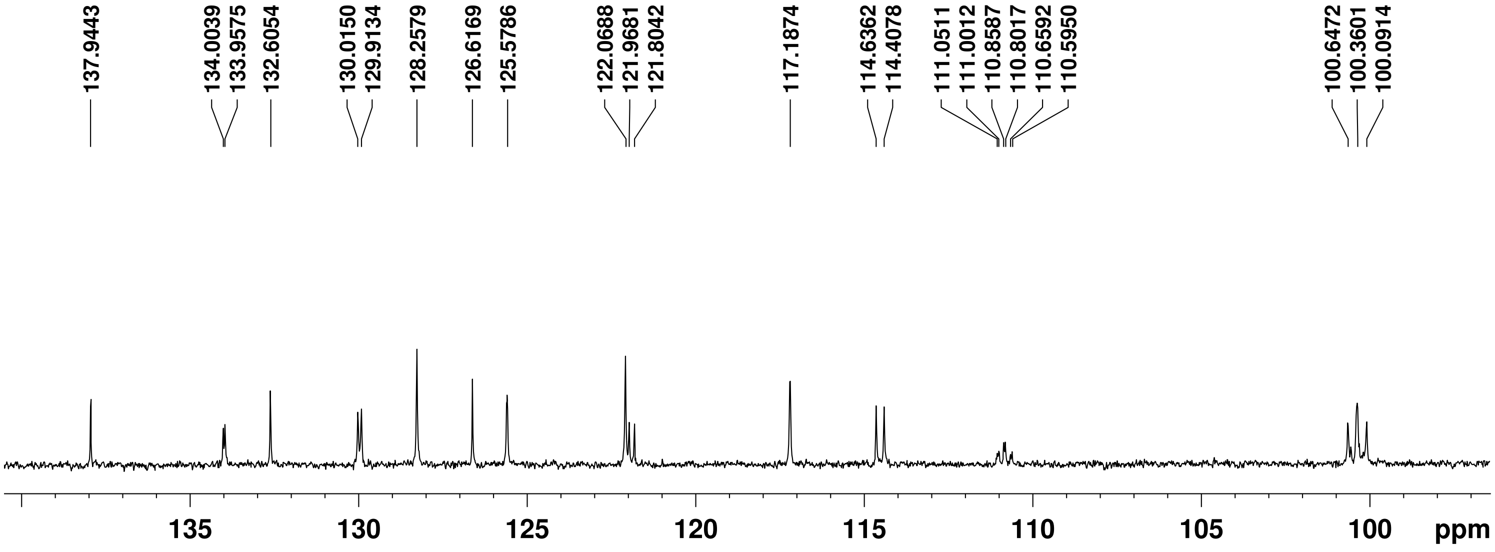

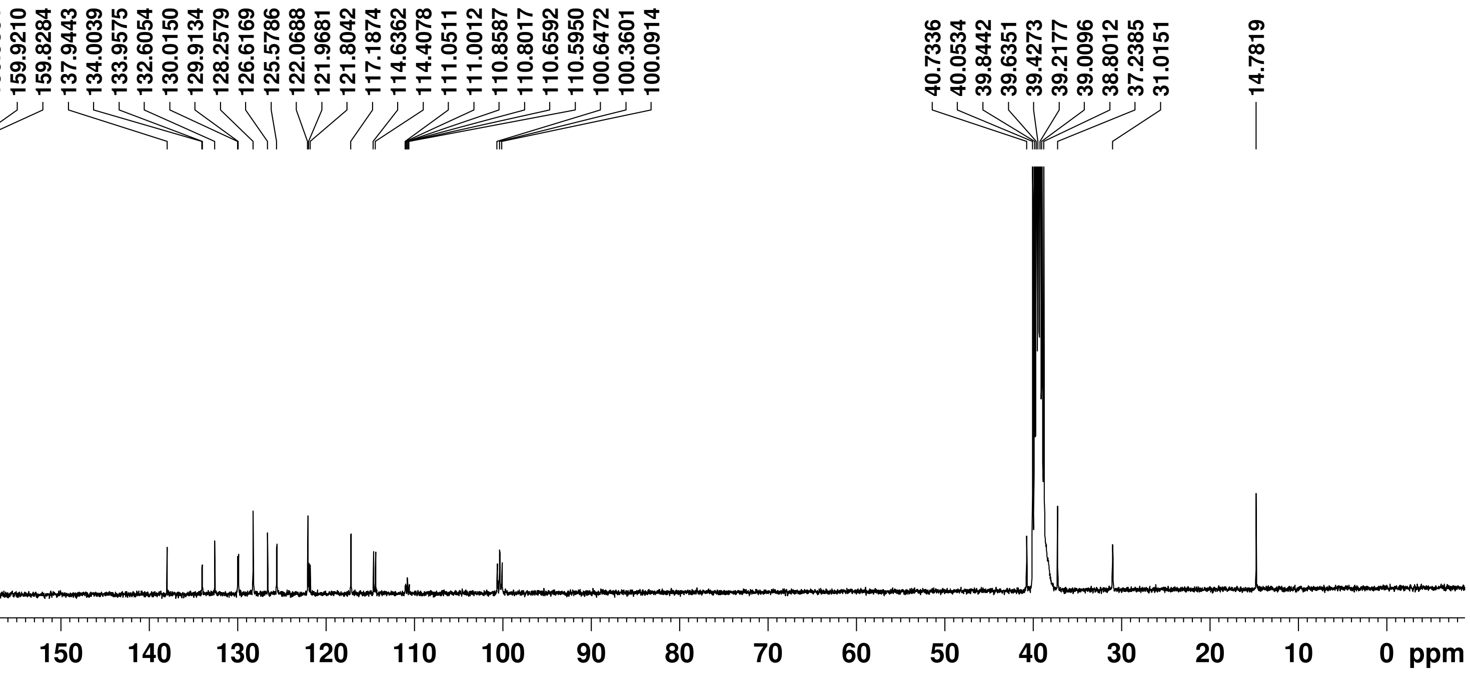

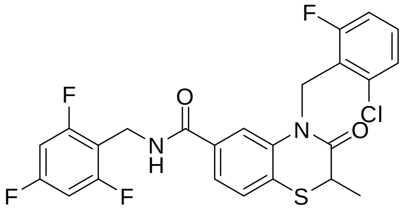

Compound 8 (9g)

Compound 8 (9g)

TCG Lifesciences Private Limited
Kolkata

NAME	CRD-2830
EXPNO	61
PROCNO	1
Date_	20200628
Time	22.41 h
INSTRUM	spect
PROBHD	Z8246_0048 (PH
PULPROG	jmod
TD	32768
SOLVENT	DMSO
NS	18000
DS	4
SWH	25252.525 Hz
FIDRES	1.541292 Hz
AQ	0.6488564 sec
RG	64
DW	19.800 usec
DE	6.50 usec
TE	297.2 K
CNST2	145.0000000
CNST11	1.0000000
D1	2.00000000 sec
D20	0.00689655 sec
TD0	1
SFO1	100.6152855 MHz
NUC1	13C
P1	9.10 usec
P2	18.20 usec
SI	16384
SF	100.6052824 MHz
WDW	EM
SSB	0
LB	1.00 Hz
GB	0
PC	1.40

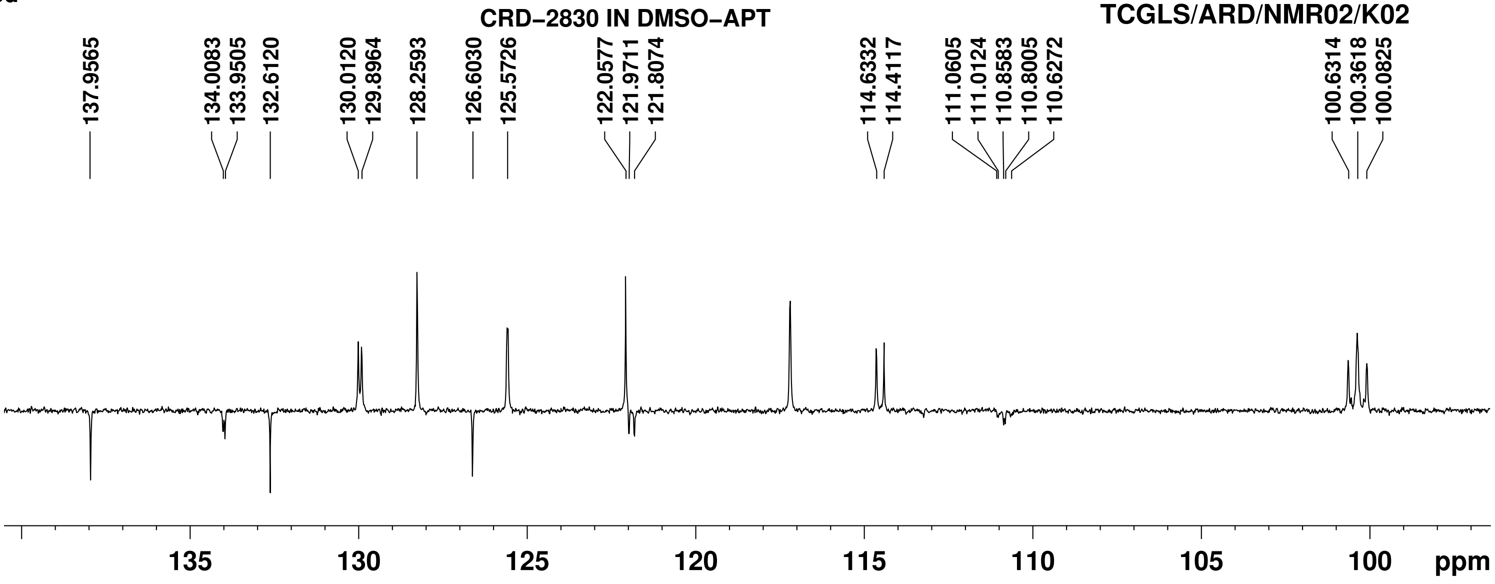

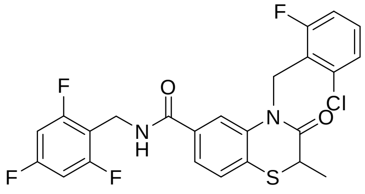

Compound 8 (9g)

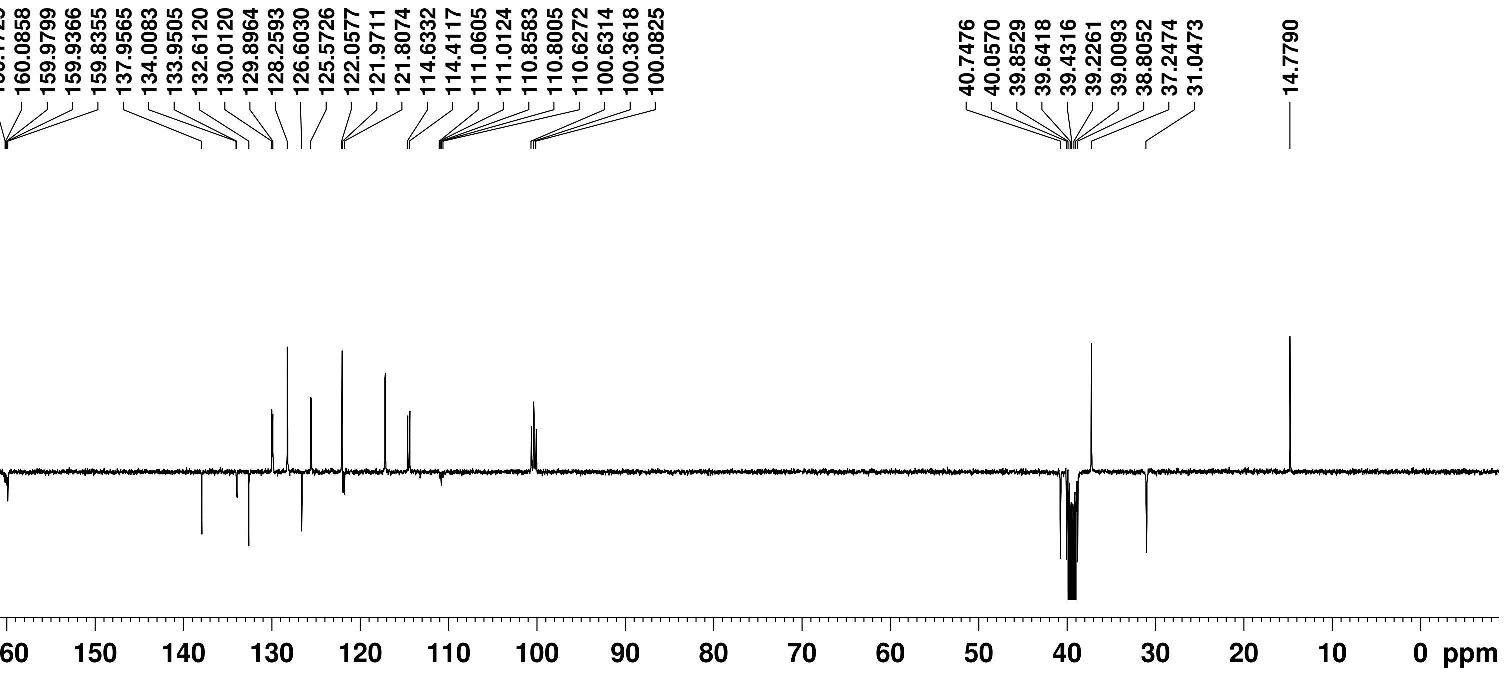

Qualitative Analysis Report

Compound 8 (9g)

**Data Filename**	AS-CRD-2830.d	**Sample Name**	AS-CRD-2830
**Sample Type**	Sample	**Position**	Vial 66
**Instrument Name**	Instrument 1	**User Name**	
**Acq Method**	Direct Mass-2017.m	**Acquired Time**	6/16/2020 12:17:06 PM
**IRM Calibration Status**	Some Ions Missed	**DA Method**	Default.m
**Comment**			

Sample Group **Info.**

Acquisition SW Version 6200 series TOF/6500 series Q-TOF B.05.00 (B5042.0)

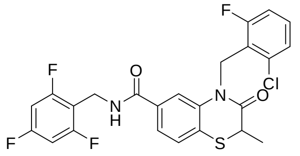

Compound 8 (9g)

Chemical Formula: $C_{24}H_{17}ClF_4N_2O_2S$
Exact Mass: 508.06
Molecular Weight: 508.92

User Chromatograms

Fragmentor Voltage 118 **Collision Energy** 0 **Ionization Mode** ESI

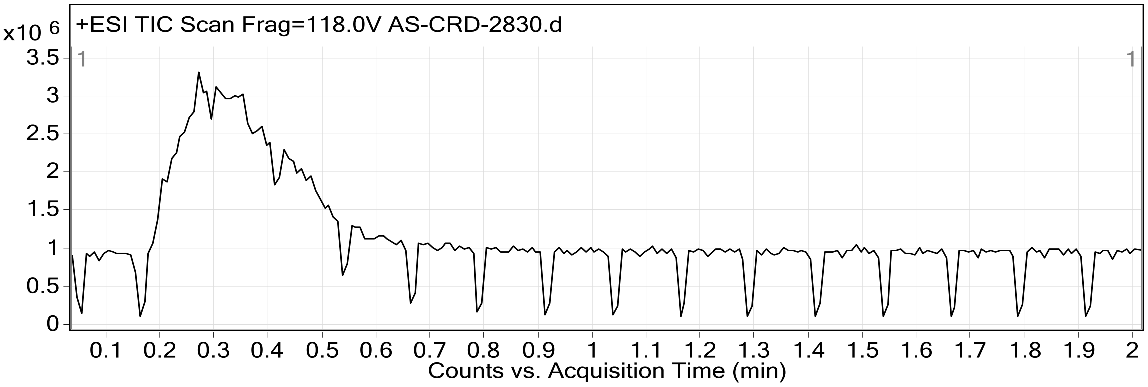

User Spectra

**Fragmentor Voltage**	**Collision Energy**	**Ionization Mode**
118	0	ESI

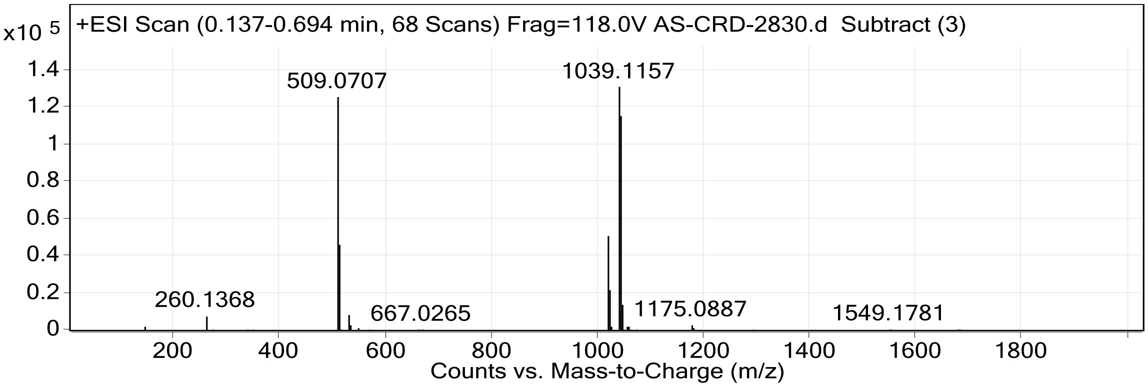

Peak List

m/z	z	Abund
509.0707	1	125857
510.0736	1	32097.83
511.0683	1	46548.43
1017.1336	1	50882.92
1019.1324	1	44839.89
1039.1157	1	131146.39

Qualitative Analysis Report

1040.1188	1	71466.97
1041.1142	1	115307.97
1042.1162	1	56239.38
1043.1134	1	35408.56

Compound 8 (9g)

Compounds

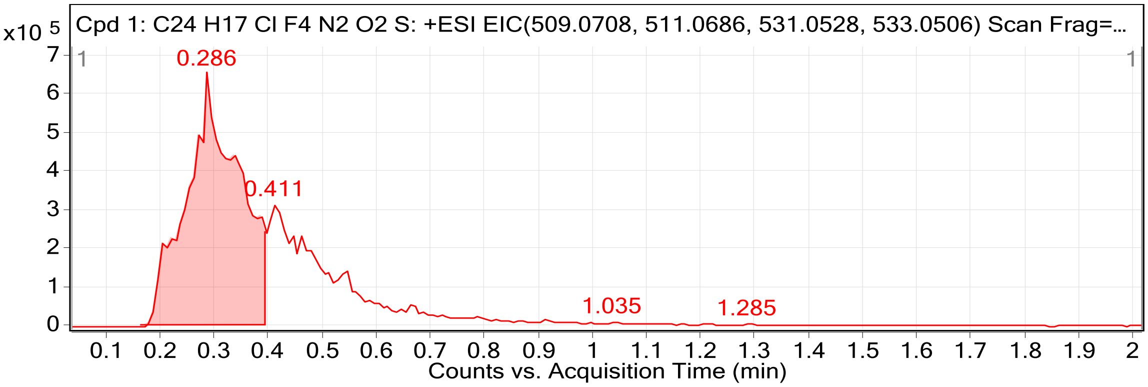

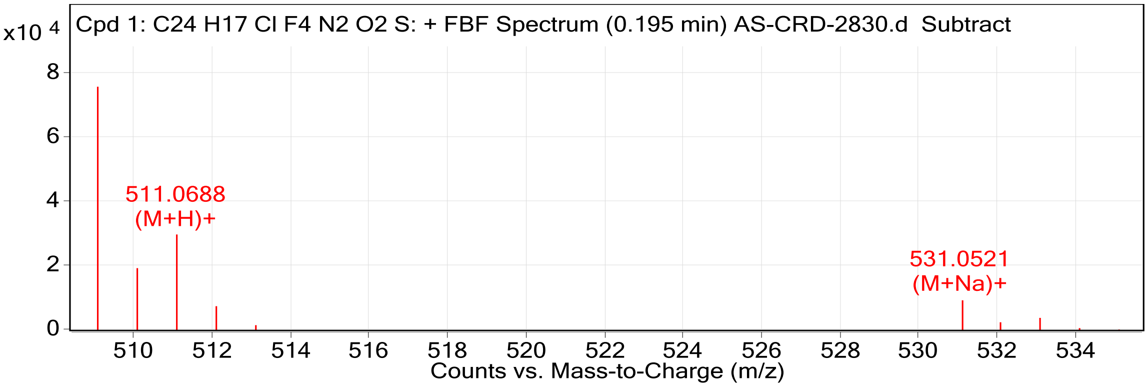

Peak List

m/z	z	Abund	Formula	Ion
509.0704	1	75824.38	C24H18ClF4N2O2S	(M+H)+
510.0745	1	19564.69	C24H18ClF4N2O2S	(M+H)+
511.0688	1	29779.45	C24H18ClF4N2O2S	(M+H)+
512.0695	1	7781.82	C24H18ClF4N2O2S	(M+H)+
513.0682	1	1819.18	C24H18ClF4N2O2S	(M+H)+
531.0521	1	9655.4	C24H17ClF4N2NaO2S	(M+Na)+
532.0555	1	2692.37	C24H17ClF4N2NaO2S	(M+Na)+
533.0493	1	4203.09	C24H17ClF4N2NaO2S	(M+Na)+
534.0505	1	1039.25	C24H17ClF4N2NaO2S	(M+Na)+
535.0484	1	305.61	C24H17ClF4N2NaO2S	(M+Na)+

MASS

Compound 8 (9g)

SAMPLE INFORMATION

Sample Name:	GB-CA218-140	Acquired By:	UPLC_MS_01 System
Vial:	1:A,8	Sample Set Name:	SAMPLE_FA
Injection #:	1	Acq. Method Set:	PH HEX_FA_6min
Injection Volume:	0.30 ul	Processing Method:	MASS
Run Time:	6.0 Minutes	Channel Name:	509.3Da
Date Acquired:	21-04-2017 12:49:22 IST	Date Processed:	24-04-2017 13:00:09 IST

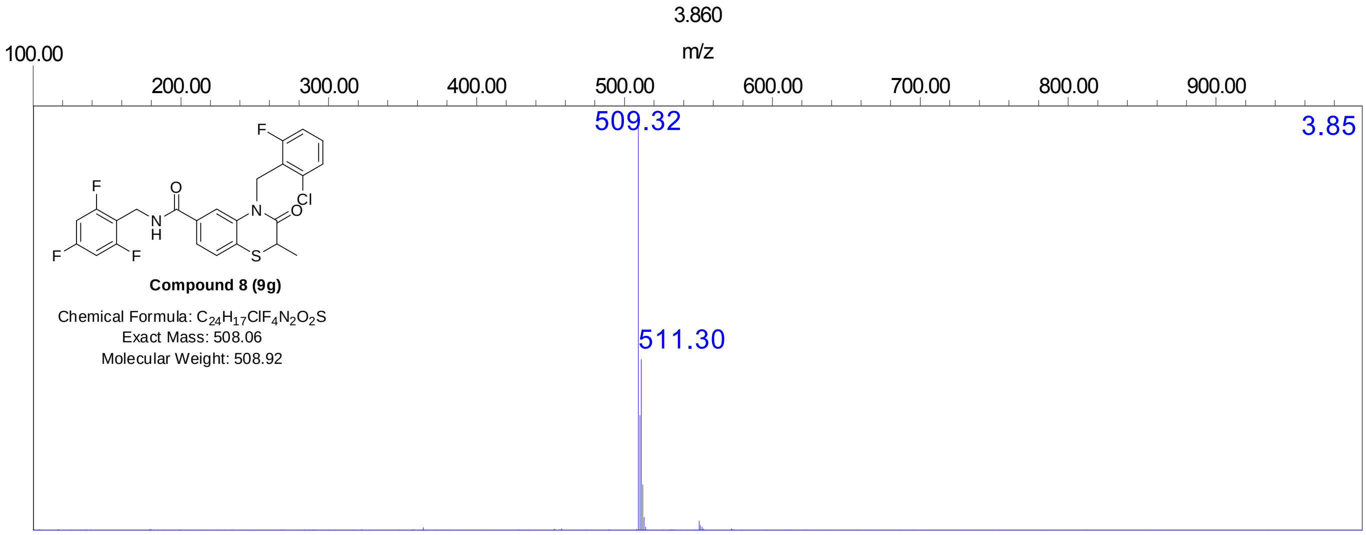

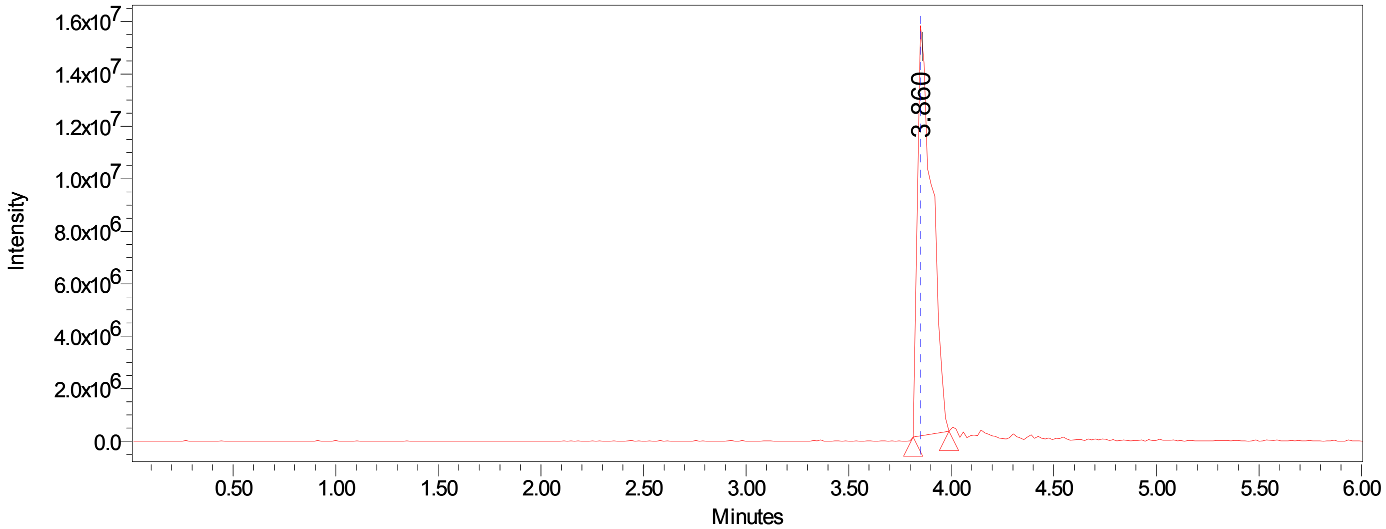

Channel Description 1: 100.00-1000.00 ES+, Centroid, CV=Tune; Processed Channel Descr. W3100 1: MS Scan MS 509.31 m/z Peak Separation: 1.0000 (1: 100.00-1000.00 ES+, Centroid, CV=Tune)

UPLC_FA

Compound 8 (9g)

SAMPLE INFORMATION

Sample Name:	GB-CA218-140	Acquired By:	UPLC_MS_01 System
Vial:	1:A,8	Sample Set Name:	SAMPLE_FA
Injection #:	1	Acq. Method Set:	PH HEX_FA_6min
Injection Volume:	0.30 ul	Processing Method:	UPLC
Run Time:	6.0 Minutes	Channel Name:	240.0nm@1
Date Acquired:	21-04-2017 12:49:22 IST	Date Processed:	24-04-2017 12:59:22 IST
Column	PHENYL HEXYL (2.1x100mm)	Mobile Phase	0.1% FA in Water/ACN

Auto-Scaled Chromatogram

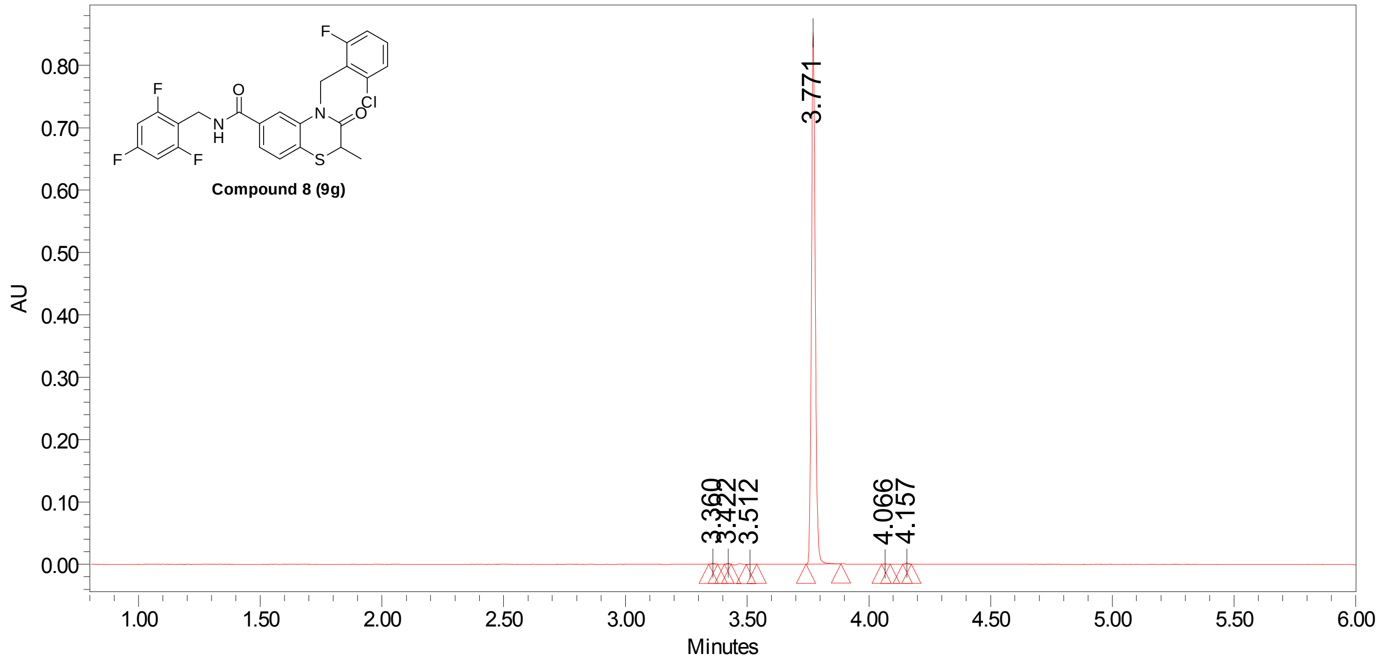

Processed Channel Descr. PDA 240.0 nm (PDA Spectrum (210-400)nm) Blank Subtracted from BLANK, Vial 1:F,8 Inj. 1

Peak Results

	Name	RT	Area	% Area	Height
1		3.360	1562	0.17	1466
2		3.422	1355	0.14	1607
3		3.512	645	0.07	510
4		3.771	930039	99.38	853797
5		4.066	530	0.06	475
6		4.157	1742	0.19	1796

Compound 9 (10b)

NP-CA264-75-A2

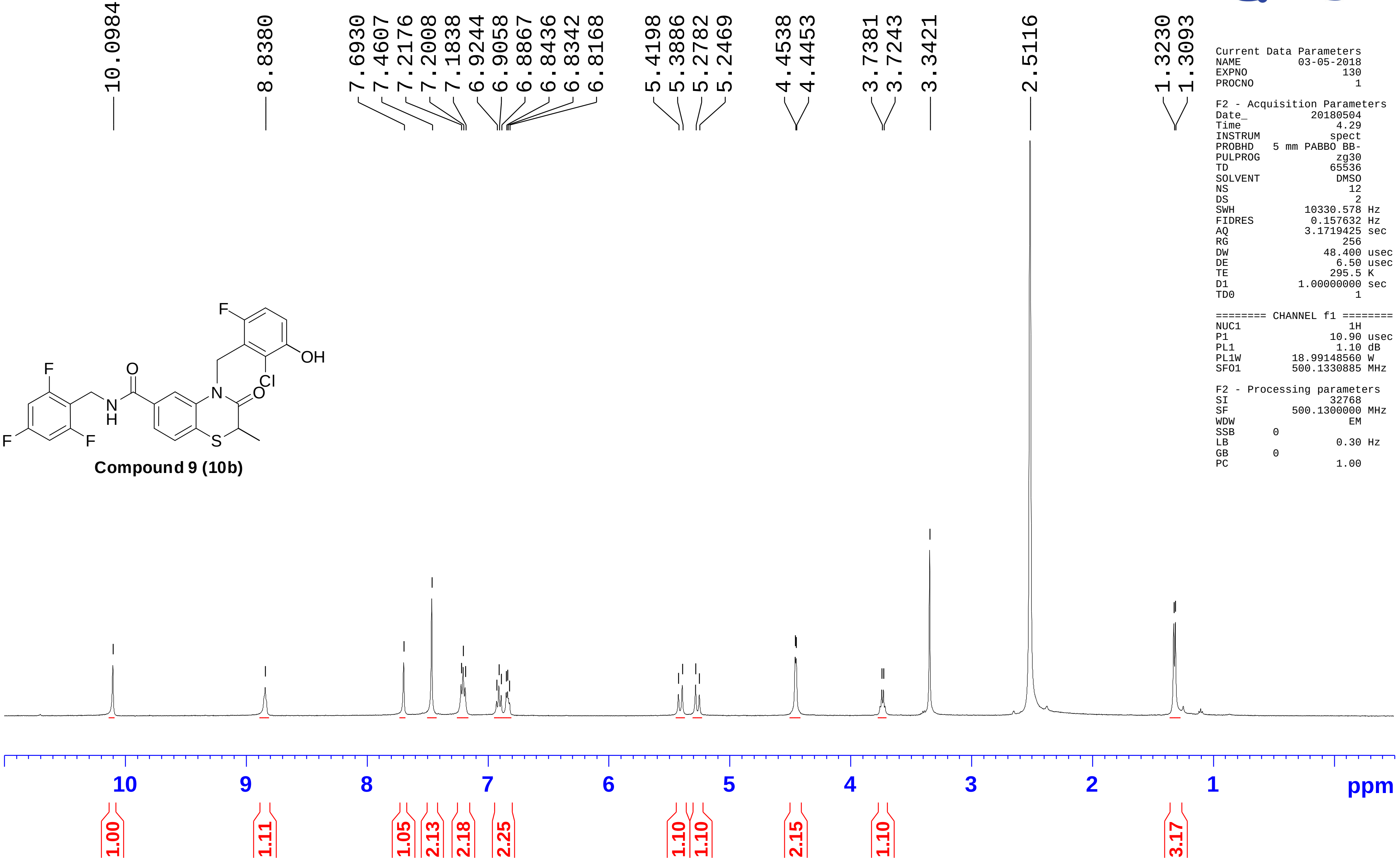

Compound 9 (10b)

```
Current Data Parameters
NAME          03-05-2018
EXPNO                130
PROCNO                 1

F2 - Acquisition Parameters
Date_           20180504
Time                4.29
INSTRUM            spect
PROBHD   5 mm PABBO BB-
PULPROG             zg30
TD                 65536
SOLVENT             DMSO
NS                    12
DS                     2
SWH           10330.578 Hz
FIDRES         0.157632 Hz
AQ            3.1719425 sec
RG                   256
DW                48.400 usec
DE                  6.50 usec
TE                 295.5 K
D1            1.00000000 sec
TD0                    1

======== CHANNEL f1 ========
NUC1                  1H
P1                 10.90 usec
PL1                 1.10 dB
PL1W         18.99148560 W
SFO1         500.1330885 MHz

F2 - Processing parameters
SI                 32768
SF           500.1300000 MHz
WDW                   EM
SSB      0
LB                  0.30 Hz
GB       0
PC                  1.00
```

Compound 9 (10b)

TCG Lifesciences Private Limited

Kolkata

CRD−3998 IN DMSO−13C

TCGLS/ARD/NMR02/K02

NAME CRD−3998
EXPNO 60
PROCNO 1
Date_ 20200614
Time 18.22 h
INSTRUM spect
PROBHD Z8246_0048 (PH
PULPROG zgpg30
TD 32768
SOLVENT DMSO
NS 32000
DS 2
SWH 25252.525 Hz
FIDRES 1.541292 Hz
AQ 0.6488564 sec
RG 64
DW 19.800 usec
DE 6.50 usec
TE 297.2 K
D1 2.00000000 sec
D11 0.03000000 sec
TD0 1
SFO1 100.6152855 MHz
NUC1 13C
P0 3.03 usec
P1 9.10 usec
SI 16384
SF 100.6052806 MHz
WDW EM
SSB 0
LB 1.00 Hz
GB 0
PC 1.40

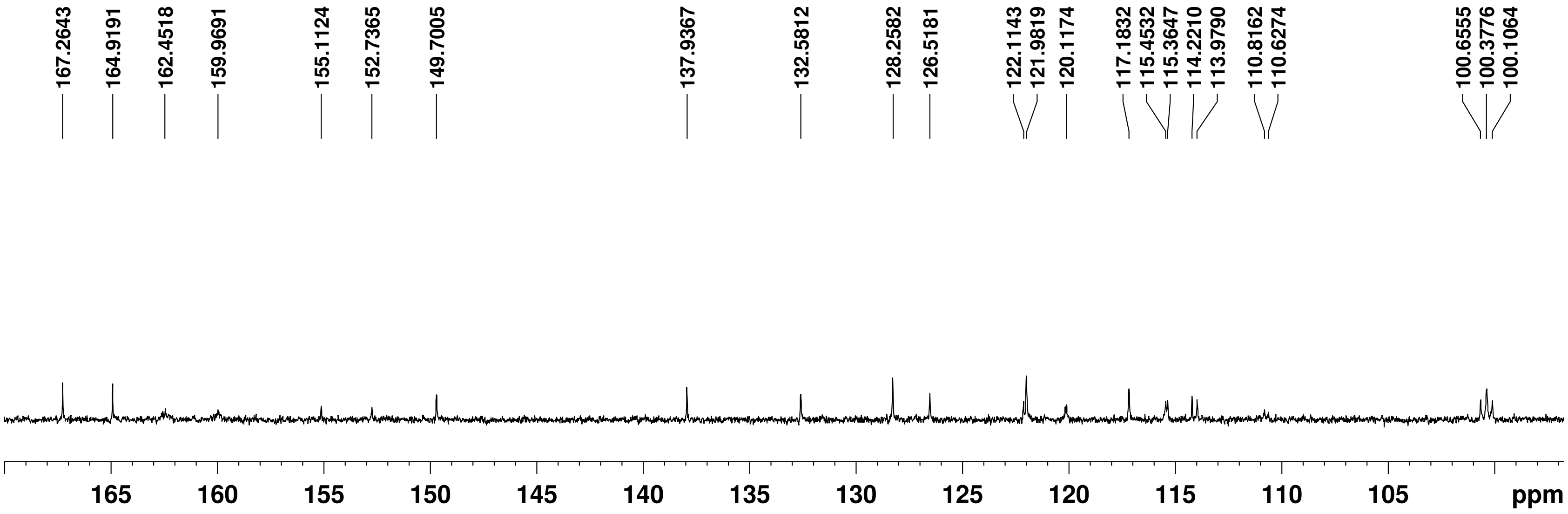

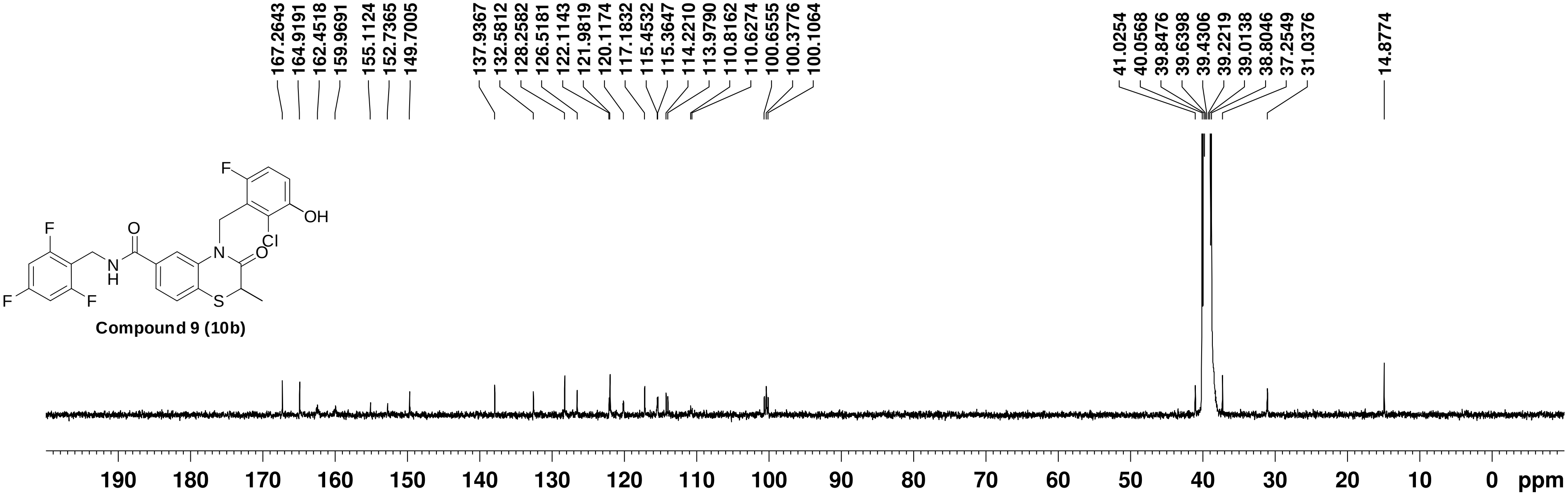

Compound 9 (10b)

Compound 9 (10b)

TCG Lifesciences Private Limited
Kolkata

CRD−3998 IN DMSO−APT

TCGLS/ARD/NMR02/K02

NAME	CRD−3998
EXPNO	61
PROCNO	1
Date_	20200615
Time	0.22 h
INSTRUM	spect
PROBHD	Z8246_0048 (PH
PULPROG	jmod
TD	32768
SOLVENT	DMSO
NS	8000
DS	4
SWH	25252.525 Hz
FIDRES	1.541292 Hz
AQ	0.6488564 sec
RG	64
DW	19.800 usec
DE	6.50 usec
TE	297.2 K
CNST2	145.0000000
CNST11	1.0000000
D1	2.00000000 sec
D20	0.00689655 sec
TD0	1
SFO1	100.6152855 MHz
NUC1	13C
P1	9.10 usec
P2	18.20 usec
SI	16384
SF	100.6052806 MHz
WDW	EM
SSB	0
LB	1.00 Hz
GB	0
PC	1.40

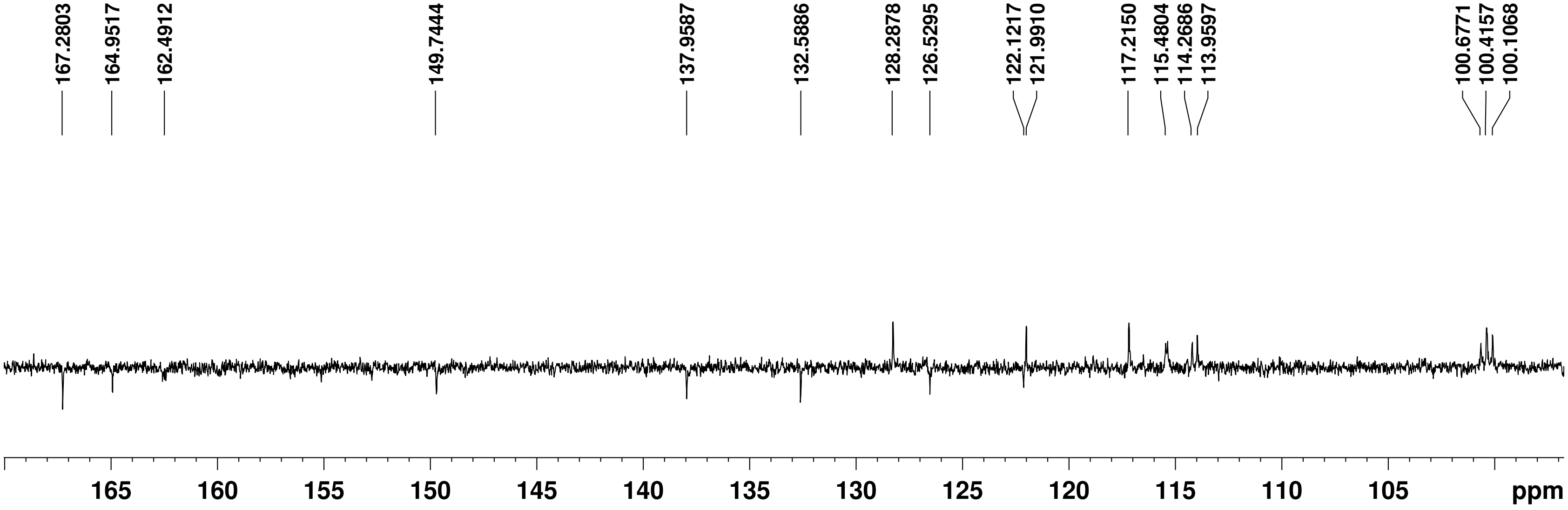

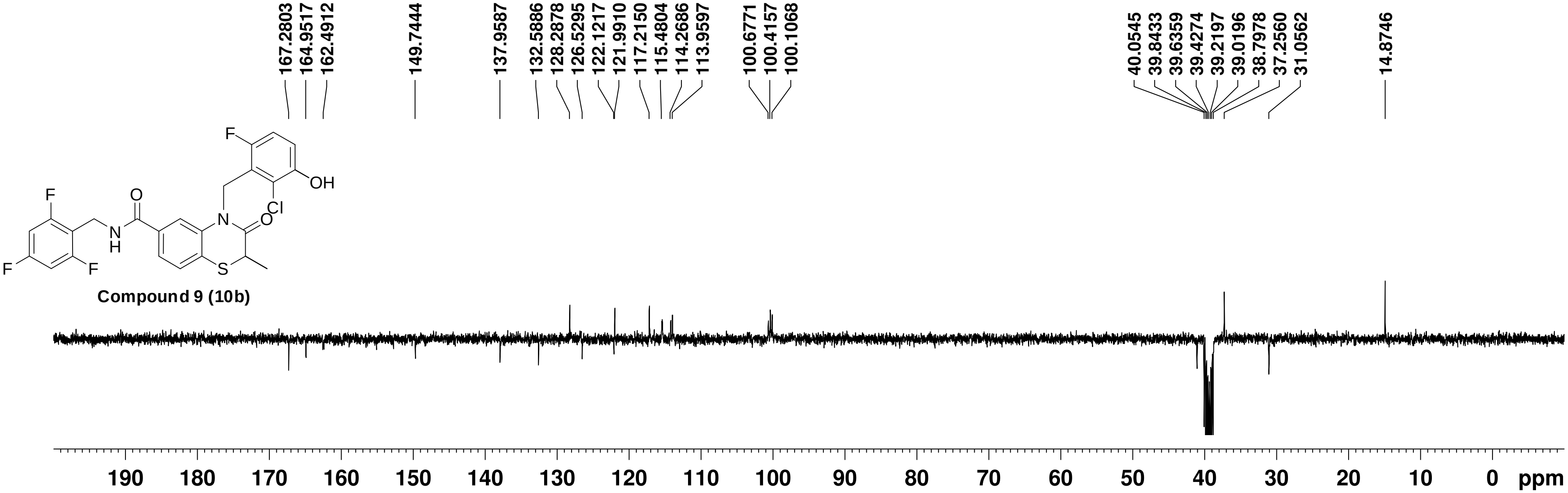

Compound 9 (10b)

Qualitative Analysis Report

Compound 9 (10b)

**Data Filename**	AS-CRD-3998.d	**Sample Name**	AS-CRD-3998
**Sample Type**	Sample	**Position**	Vial 72
**Instrument Name**	Instrument 1	**User Name**	
**Acq Method**	Direct Mass-2017.m	**Acquired Time**	6/16/2020 12:34:43 PM
**IRM Calibration Status**	Some Ions Missed	**DA Method**	Default.m
**Comment**			

Sample Group **Info.**

Acquisition SW Version 6200 series TOF/6500 series Q-TOF B.05.00 (B5042.0)

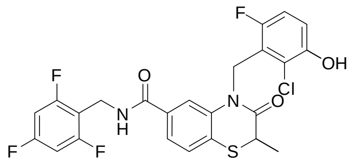

Compound 9 (10b)

Chemical Formula: $C_{24}H_{17}ClF_4N_2O_3S$
Exact Mass: 524.0585

User Chromatograms

Fragmentor Voltage 118 **Collision Energy** 0 **Ionization Mode** ESI

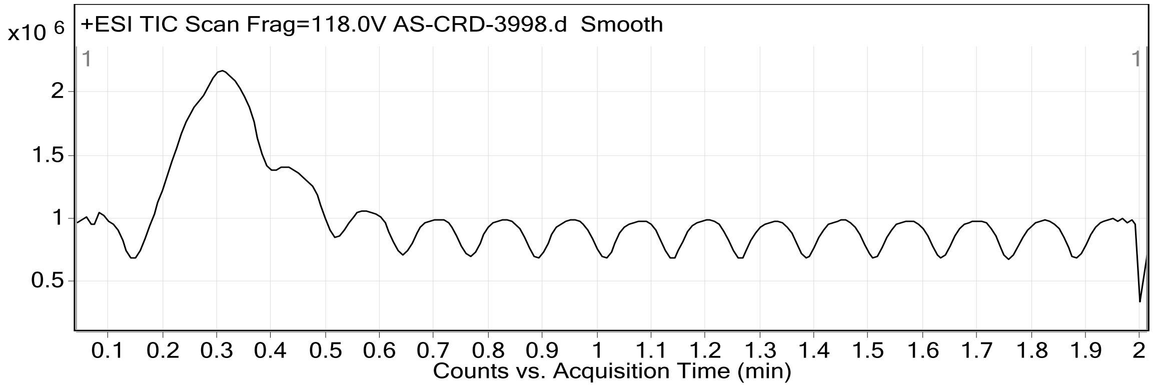

User Spectra

Fragmentor Voltage	Collision Energy	Ionization Mode
118	0	ESI

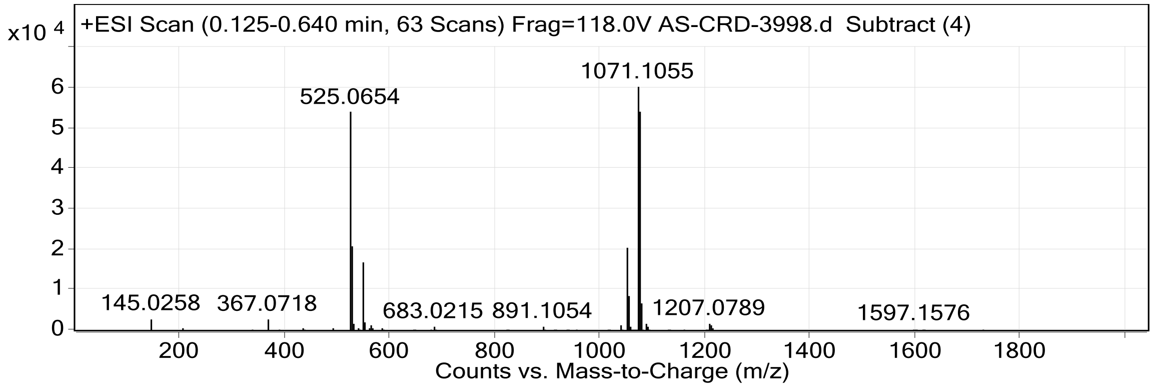

Peak List

m/z	z	Abund
525.0654	1	54036.87
527.0631	1	20971.18
547.0473	1	16822.48
1049.1209	1	20701.78
1051.1207	1	18178.87
1071.1055	1	60383.63

1072.1086	1	33367.96
1073.1043	1	54042.46
1074.106	1	26131.9
1075.1033	1	16867.12

Compound 9 (10b)

Compounds

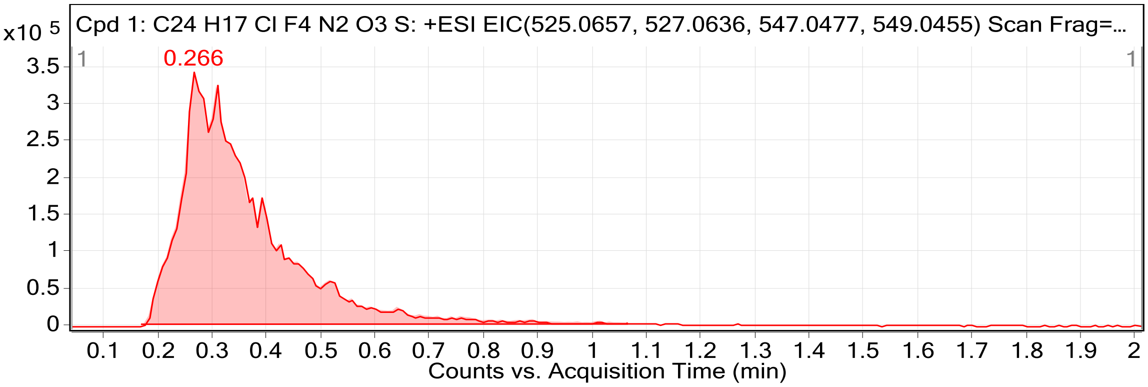

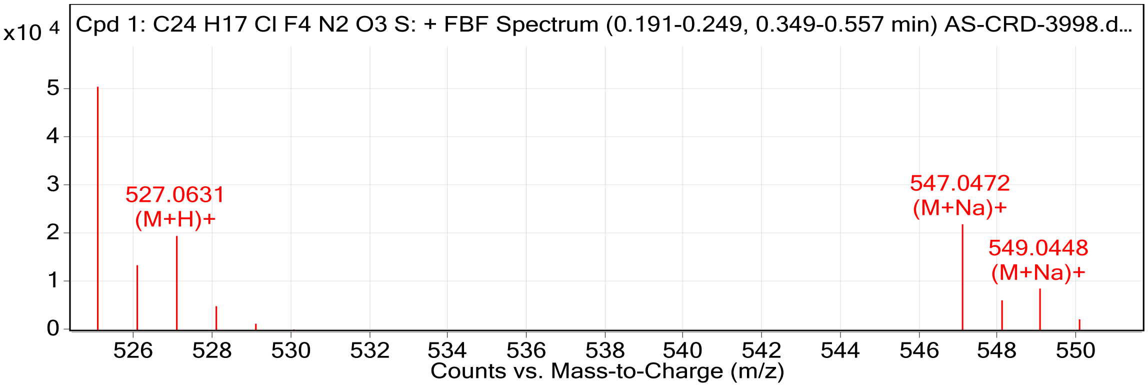

Peak List

m/z	z	Abund	Formula	Ion
525.0653	1	50624.63	C24H18ClF4N2O3S	(M+H)+
526.0685	1	13669.19	C24H18ClF4N2O3S	(M+H)+
527.0631	1	19795.75	C24H18ClF4N2O3S	(M+H)+
528.0657	1	5142.39	C24H18ClF4N2O3S	(M+H)+
529.0629	1	1541.26	C24H18ClF4N2O3S	(M+H)+
530.0651	1	323.04	C24H18ClF4N2O3S	(M+H)+
547.0472	1	22175.64	C24H17ClF4N2NaO3S	(M+Na)+
548.0498	1	6232.94	C24H17ClF4N2NaO3S	(M+Na)+
549.0448	1	8728.67	C24H17ClF4N2NaO3S	(M+Na)+
550.0475	1	2354.4	C24H17ClF4N2NaO3S	(M+Na)+

Compound 9 (10b)

SAMPLE INFORMATION

Sample Name:	NP-CA264-75-A1	Acquired By:	UPLC_MS_01 System
Vial:	1:E,4	Sample Set Name:	AA
Injection #:	1	Acq. Method Set:	AA_C18_6min_N
Injection Volume:	0.30 ul	Processing Method:	MASS
Run Time:	6.0 Minutes	Channel Name:	525.1Da
Date Acquired:	03-05-2018 11:27:24 IST	Date Processed:	03-05-2018 12:12:37 IST

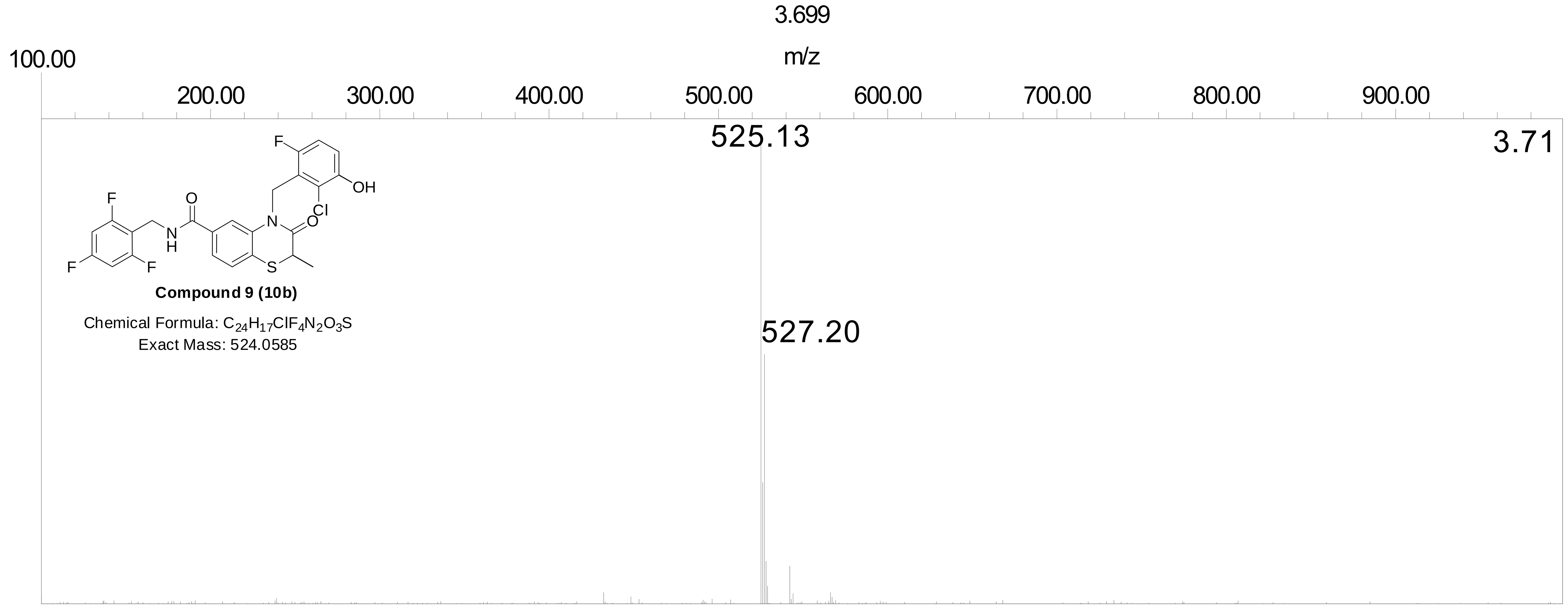

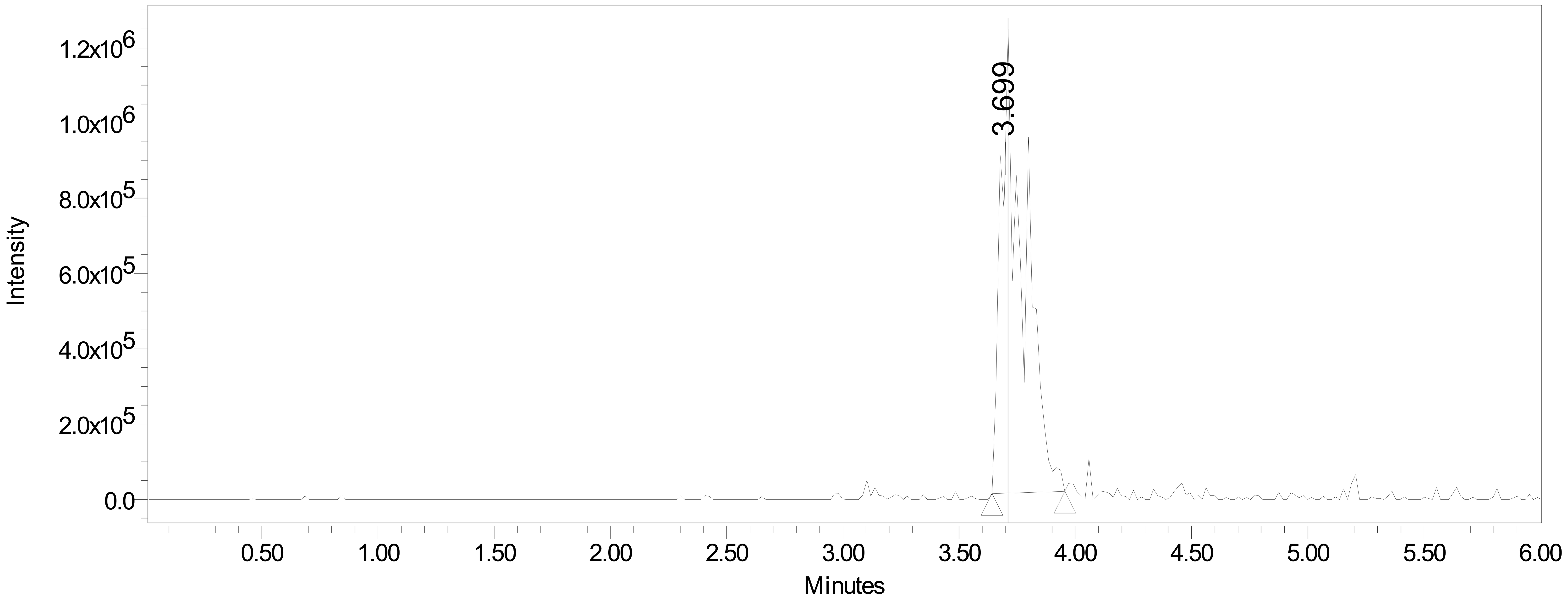

Channel Description 1: 100.00-1000.00 ES+, Centroid, CV=Tune; Processed Channel Descr. W3100 1: MS Scan MS 525.13 m/z Peak Separation: 1.0000 (1: 100.00-1000.00 ES+, Centroid, CV=Tune)

Empower 3 SOFTWARE

Compound 9 (10b)

SAMPLE INFORMATION

Sample Name:	NP-CA264-75-A1	Acquired By:	UPLC_MS_01 System
Vial:	1:E,4	Sample Set Name:	AA
Injection #:	1	Acq. Method Set:	AA_C18_6min_N
Injection Volume:	0.30 ul	Processing Method:	UPLC
Run Time:	6.0 Minutes	Channel Name:	240.0nm@4
Date Acquired:	03-05-2018 11:27:24 IST	Date Processed:	03-05-2018 12:39:31 IST
Column	KINETEX_EVO_C-18	Mobile Phase	1mM A.A. in Water/ACN

Auto-Scaled Chromatogram

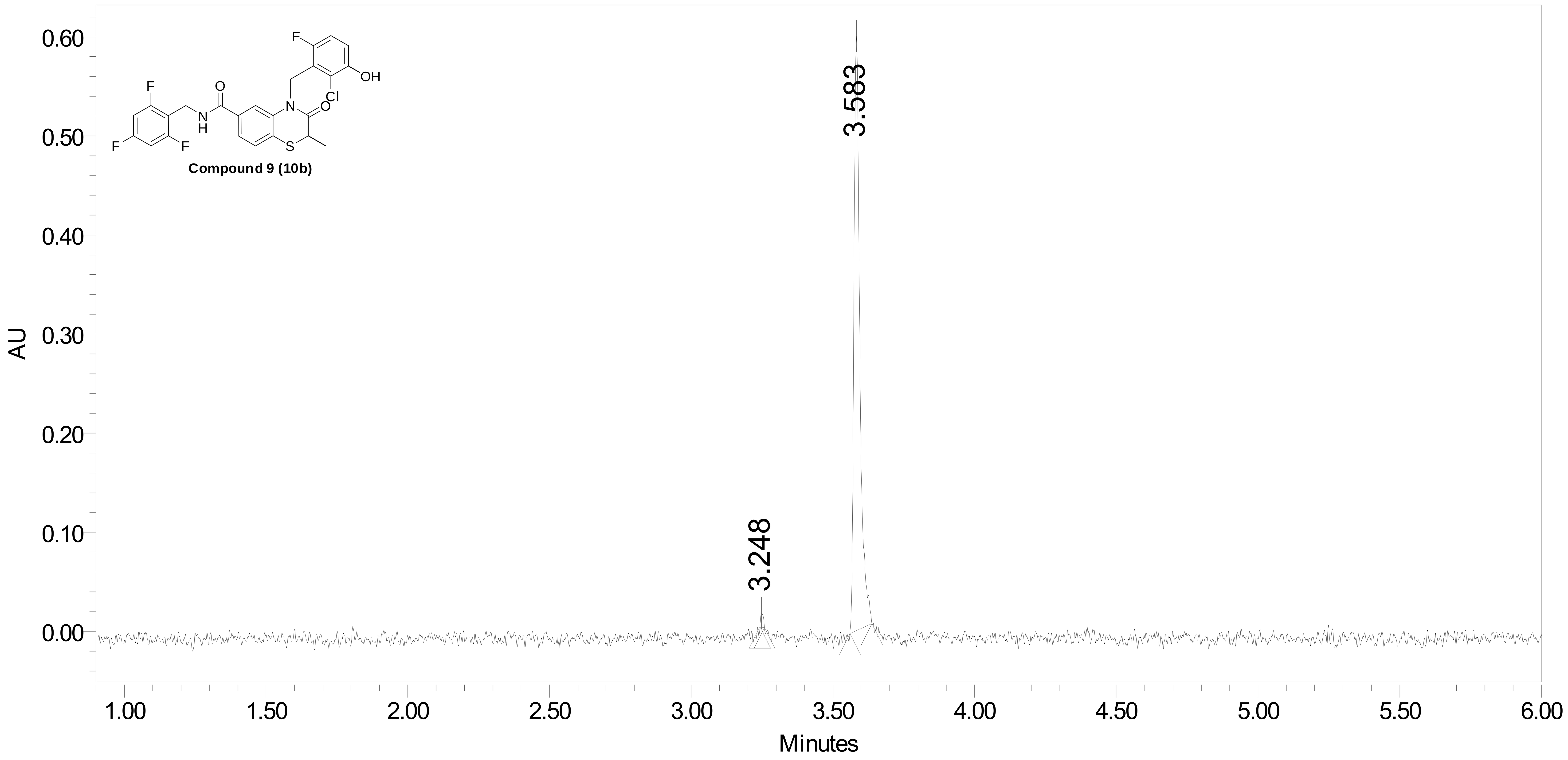

Processed Channel Descr. PDA 240.0 nm (PDA Spectrum (210-400)nm) Blank Subtracted from , Vial Inj. 115080592

Peak Results

	Name	RT	Area	% Area	Height
1		3.248	9401	1.06	14214
2		3.583	878474	98.94	600838

Compound 10 (9i)

CHEMBIOTEK, A TCG Lifesciences Private Limited

Kolkata

CR240−CA187−119−P1_GB−CA218−145 IN DMSO

TCGLS/ARD/NMR01/K01

Current Data Parameters
NAME CR240−CA187−119−P1_GB−CA218−145
EXPNO 10
PROCNO 1

F2 − Acquisition Parameters
Date_ 20170428
Time 13.22
INSTRUM spect
PROBHD 5 mm DUL 13C−1
PULPROG zg30
TD 24036
SOLVENT DMSO
NS 32
DS 0
SWH 8012.820 Hz
FIDRES 0.333367 Hz
AQ 1.4998964 sec
RG 45.2
DW 62.400 usec
DE 6.50 usec
TE 300.0 K
D1 1.00000000 sec
TD0 1

======== CHANNEL f1 ========
NUC1 1H
P1 14.50 usec
PL1 −1.00 dB
PL1W 9.92955208 W
SFO1 400.1024654 MHz

F2 − Processing parameters
SI 16384
SF 400.1000041 MHz
WDW EM
SSB 0
LB 0.30 Hz
GB 0
PC 1.00

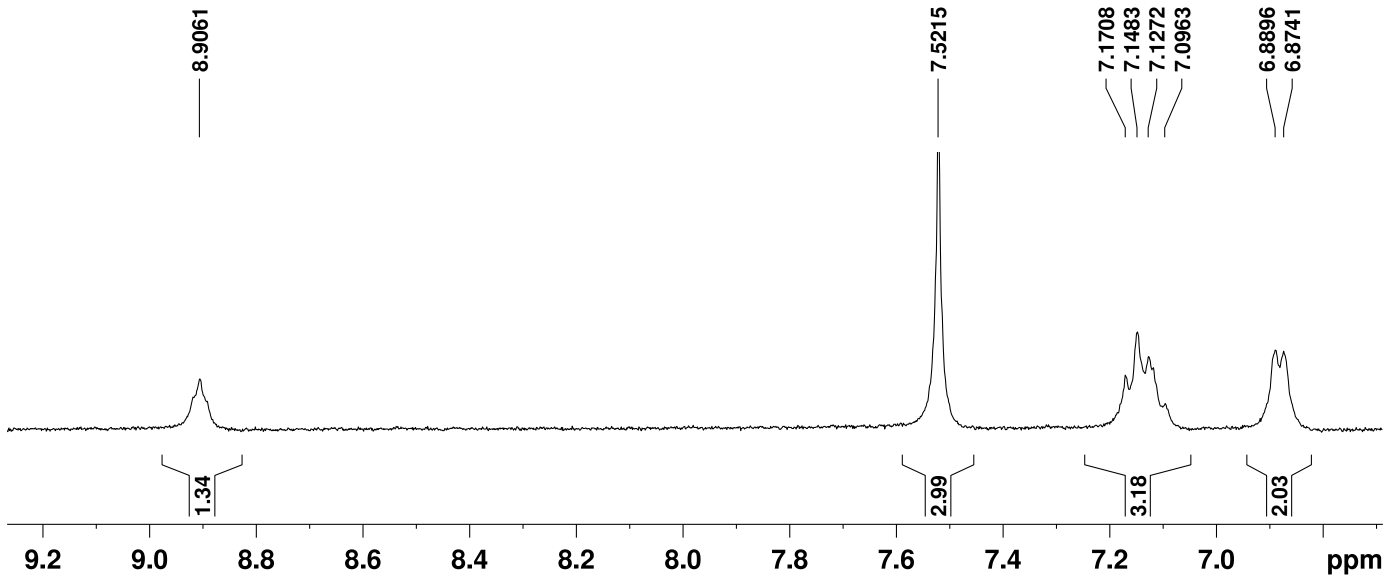

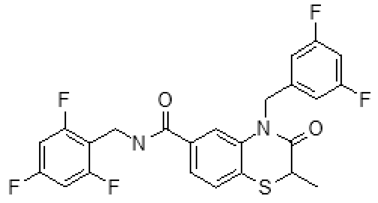

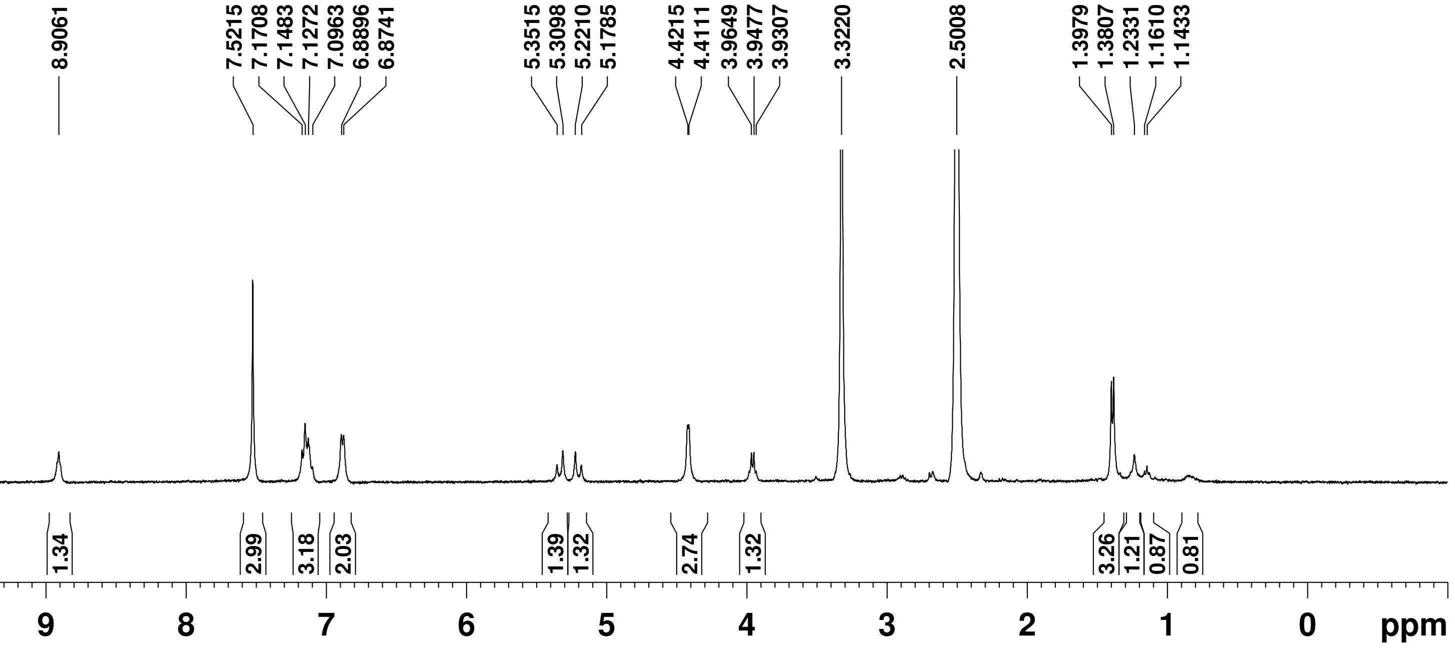

Compound 10 (9i)

CHEMBIOTEK, A TCG Lifesciences Private Limited

Kolkata

CR240−CA187−119−P1_GB−CA218−145 D2O EXCHANGE

TCGLS/ARD/NMR01/K01

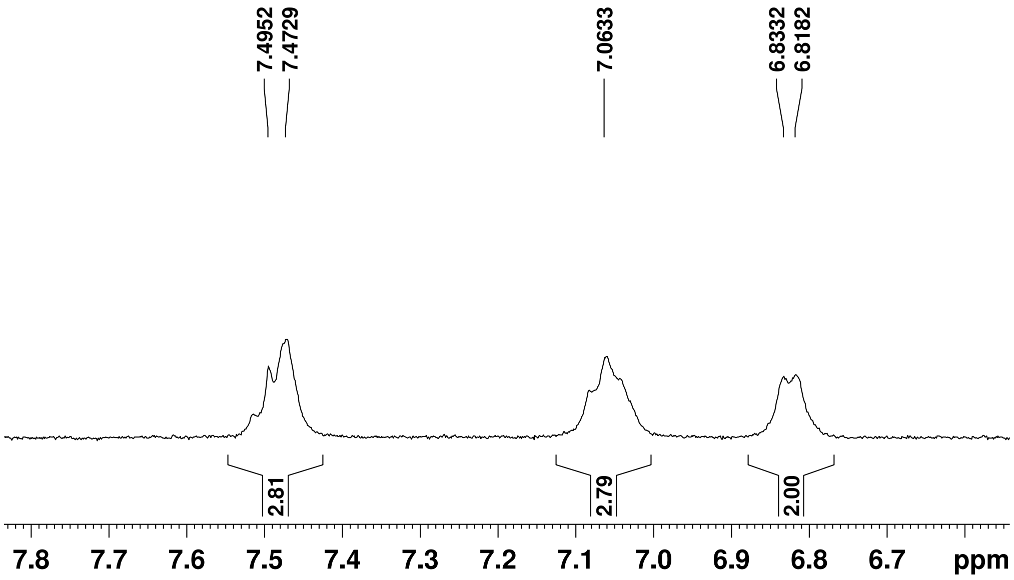

Current Data Parameters
NAME CR240−CA187−119−P1_GB−CA218−145
EXPNO 10
PROCNO 1

F2 − Acquisition Parameters
Date_ 20170429
Time 11.02
INSTRUM spect
PROBHD 5 mm DUL 13C−1
PULPROG zg30
TD 24036
SOLVENT DMSO
NS 32
DS 0
SWH 8012.820 Hz
FIDRES 0.333367 Hz
AQ 1.4998964 sec
RG 181
DW 62.400 usec
DE 6.50 usec
TE 300.0 K
D1 1.00000000 sec
TD0 1

======== CHANNEL f1 ========
NUC1 1H
P1 14.50 usec
PL1 −1.00 dB
PL1W 9.92955208 W
SFO1 400.1024654 MHz

F2 − Processing parameters
SI 16384
SF 400.1000046 MHz
WDW EM
SSB 0
LB 0.30 Hz
GB 0
PC 1.00

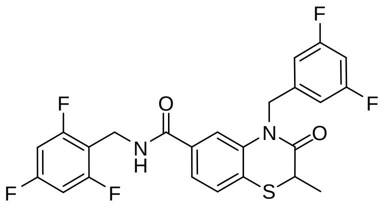

Compound 10 (9i)

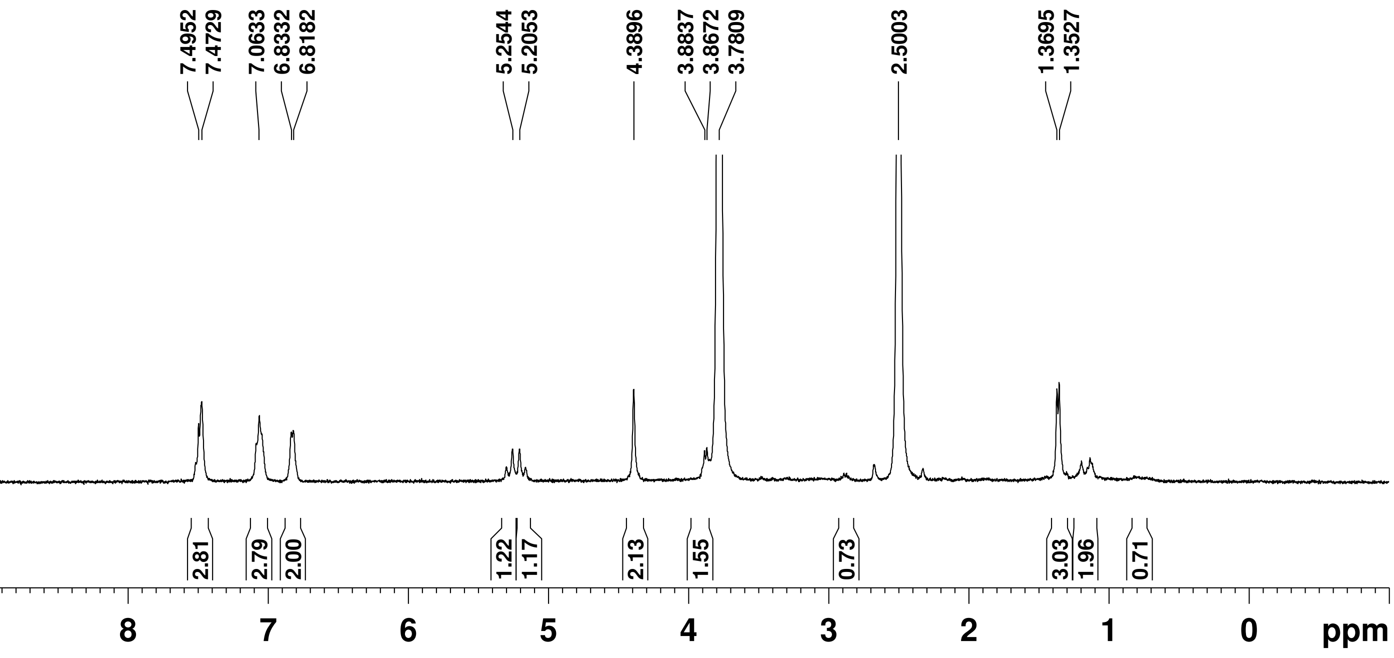

Compound 10 (9i)

===

Sample Name :CR240-CA187-119-Racemic_GB-CA218-145

Seq Line-> 11

Location : Vial 82

Acq. Operator : SOUMEN Inj. No. : 1

Injection Date : 5/2/2017 4:15:28 PM Inj. Vol. : 5 µl

Acq. Method : C:\Chem32\1\DATA\MAY-2017\020517 2017-05-02 11-01-07->

Analysis Method : C:\CHEM32\1\METHODS\WASH-2.M

Last Changed : Tue, 7. Mar. 2017, 06:37:19 pm

Sample ID : CR240-CA187-119-Racemic_GB-CA218-145

Column Name : Chiralcel OD-H (4.6 x 250 mm),5µ
ARD/K/7323

Mobile Phase : Hexane/EtOH/DEA : 80/20/0.1

Flow Rate : 1.0 ml/min

Solubility : MeOH

Ref:- SP/02.05.17/5

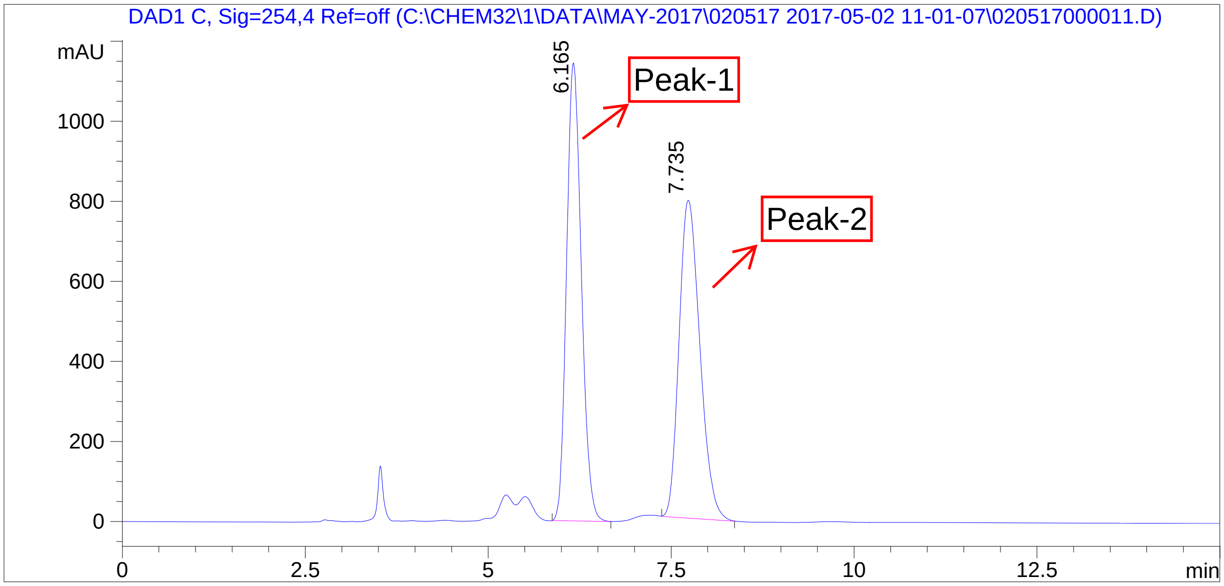

===

Signal 1: DAD1 C, Sig=254,4 Ref=off

Peak #	RT [min]	Area	Area %
1	6.16	16036.07	50.50
2	7.73	15715.84	49.50

===

*** End of Report ***

Compound 10 (9i)

===

Sample Name : CR240-CA187-119-P1_GB-CA218-145

Seq Line-> 12
Location : Vial 83
Acq. Operator : SOUMEN
Inj. No. : 1
Injection Date : 5/2/2017 4:34:47 PM
Inj. Vol. : 10 µl
Acq. Method : C:\Chem32\1\DATA\MAY-2017\020517 2017-05-02 11-01-07->
Analysis Method : C:\CHEM32\1\METHODS\WASH-2.M
Last Changed : Tue, 7. Mar. 2017, 06:37:19 pm

Sample ID : CR240-CA187-119-P1_GB-CA218-145

Column Name : Chiralcel OD-H (4.6 x 250 mm),5µ
ARD/K/7323
Mobile Phase : Hexane/EtOH/DEA : 80/20/0.1
Flow Rate : 1.0 ml/min
Solubility : MeOH

Ref:- SP/02.05.17/5

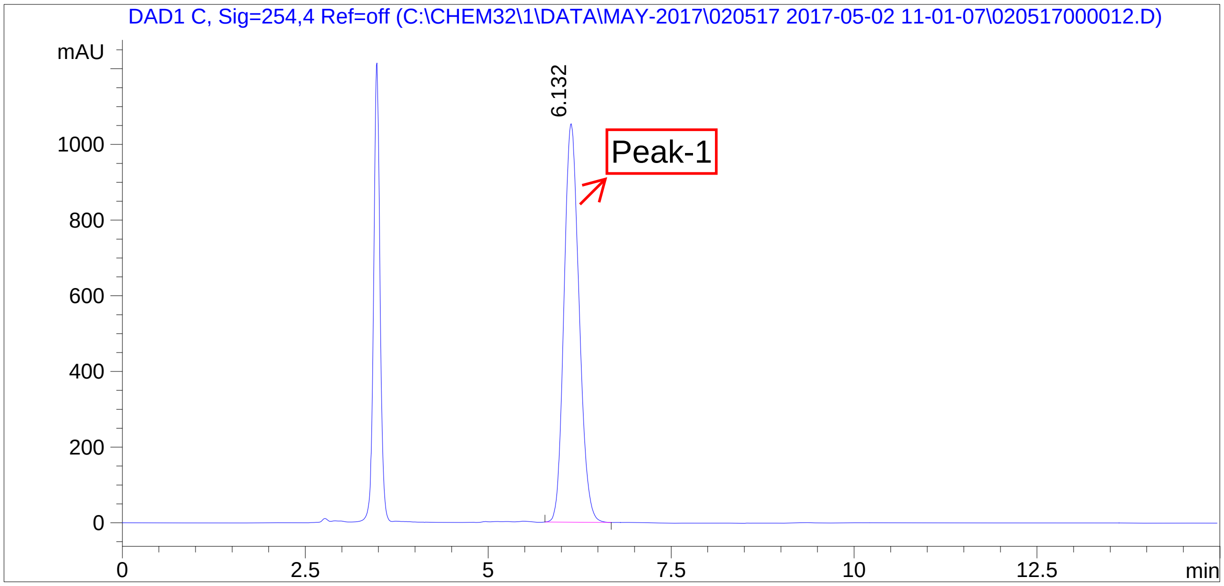

===

Signal 1: DAD1 C, Sig=254,4 Ref=off

Peak #	RT [min]	Area	Area %
1	6.13	15131.49	100.00

===

*** End of Report ***

Compound 10 (9i)

BATCH NO. **CHEMBIOTEK,TCG LIFESCIENCES ENTERPRISE** **28-Apr-201714:09:38**
CR240-CA187-119-P1_GB-CA218-145 **KOLKATA,INDIA**
Inst. No.-TCGLS/ARD/LCMS17/K71 **METHOD: MONI-CV25 (HCOOH:ACN) 3min**

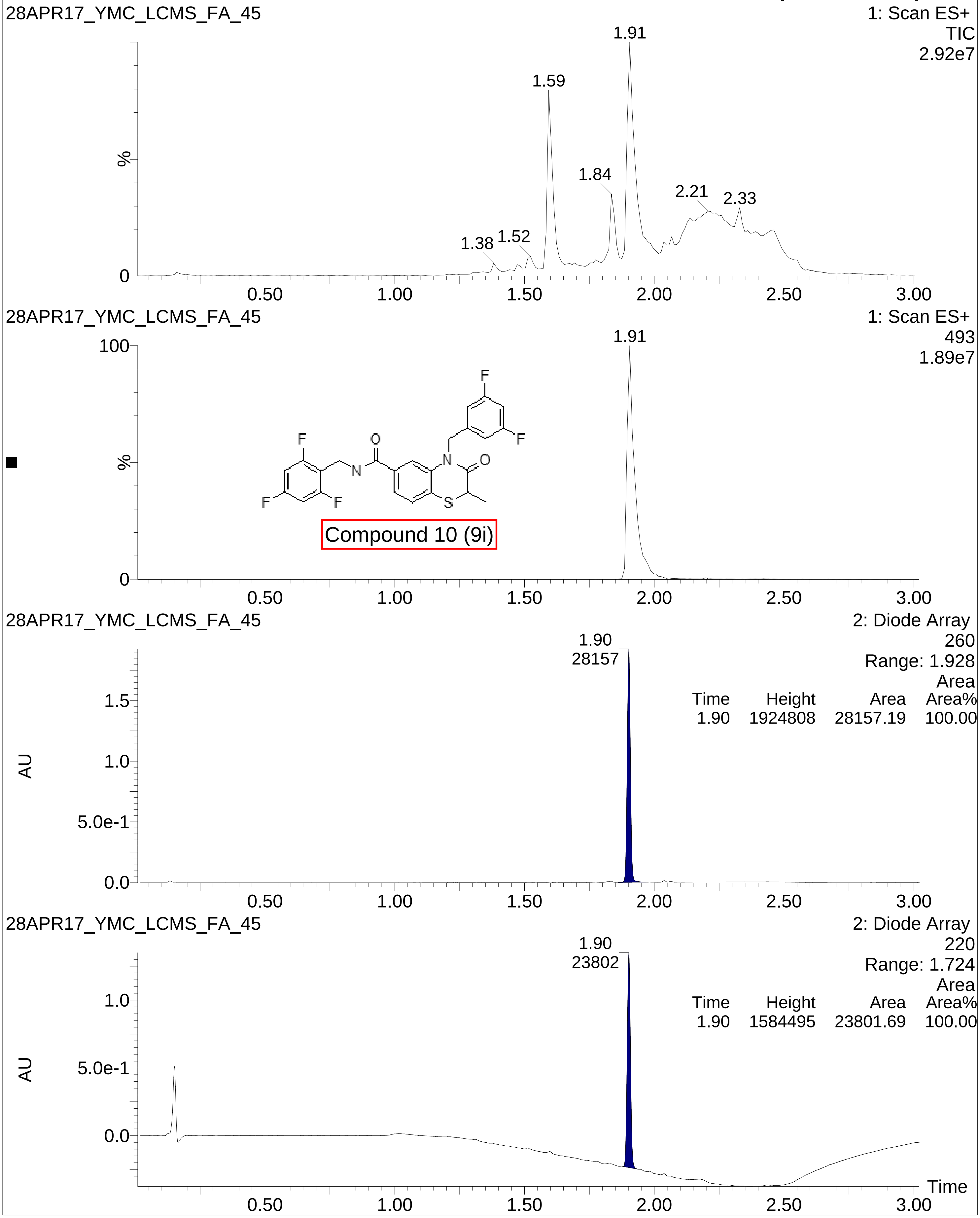

Compound 10 (9i)

BATCH NO. **CHEMBIOTEK,TCG LIFESCIENCES ENTERPRISE** **28-Apr-201714:09:38**

CR240-CA187-119-P1_GB-CA218-145 **KOLKATA,INDIA**

Inst.No.-TCGLS/ARD/LCMS17/K71

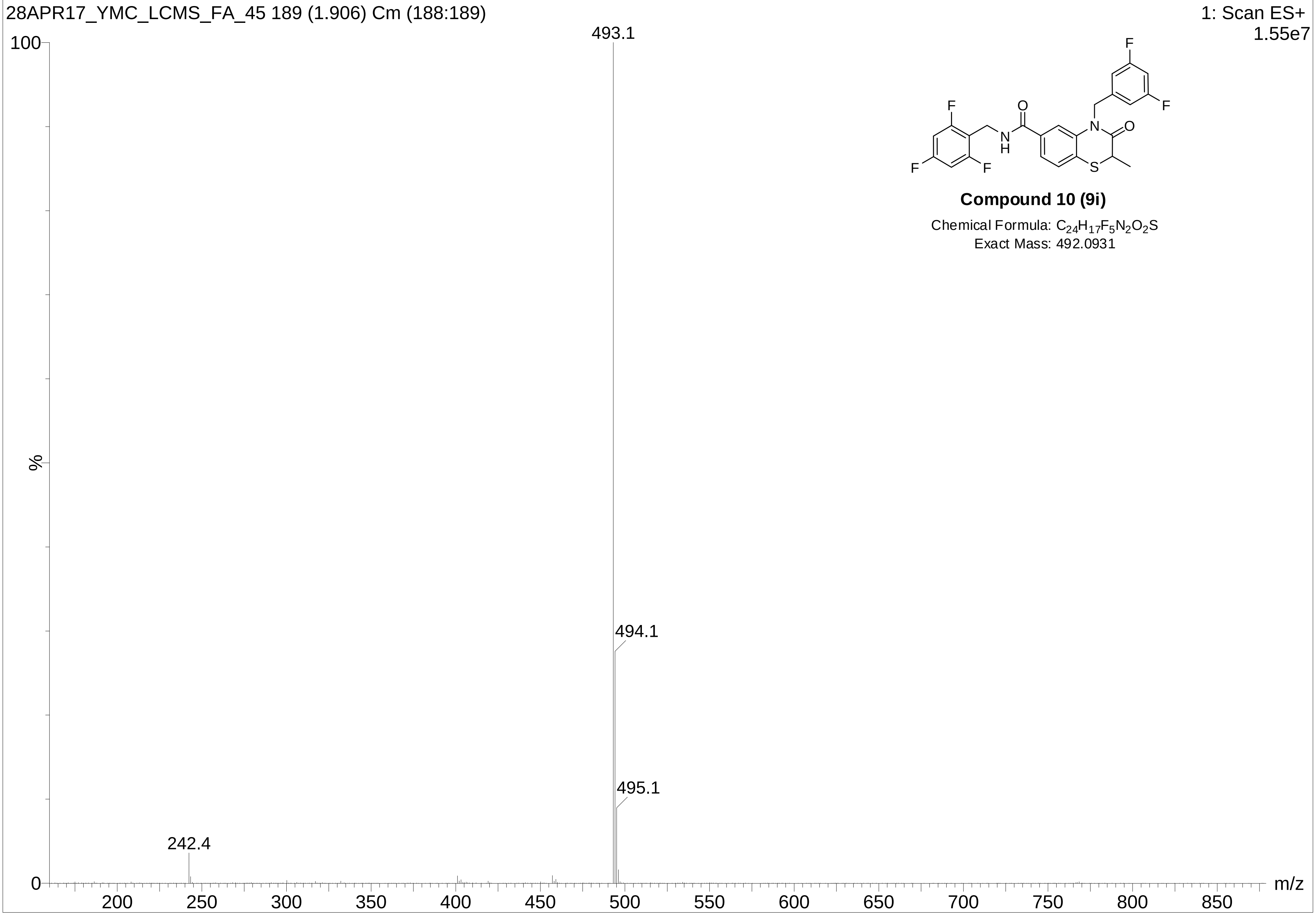

Compound 10 (9i)

TCG Lifesciences Private Limited
Kolkata

CRD−3093 IN DMSO−13C

TCGLS/ARD/NMR02/K02

NAME	CRD−3093
EXPNO	60
PROCNO	1
Date_	20200627
Time	18.08
INSTRUM	spect
PROBHD	5 mm PABBO BB−
PULPROG	zgpg30
TD	65536
SOLVENT	DMSO
NS	20000
DS	4
SWH	25252.525 Hz
FIDRES	0.385323 Hz
AQ	1.2976629 sec
RG	2050
DW	19.800 usec
DE	6.50 usec
TE	299.5 K
D1	2.00000000 sec
D11	0.03000000 sec
TD0	1

======== CHANNEL f1 ========

NUC1	13C
P1	6.75 usec
PL1	0.00 dB
SFO1	100.6404331 MHz

======== CHANNEL f2 ========

CPDPRG2	waltz16
NUC2	1H
PCPD2	80.00 usec
PL2	0.00 dB
PL12	13.45 dB
PL13	17.00 dB
SFO2	400.2016008 MHz
SI	32768
SF	100.6304307 MHz
WDW	EM
SSB	0
LB	1.00 Hz
GB	0
PC	1.40

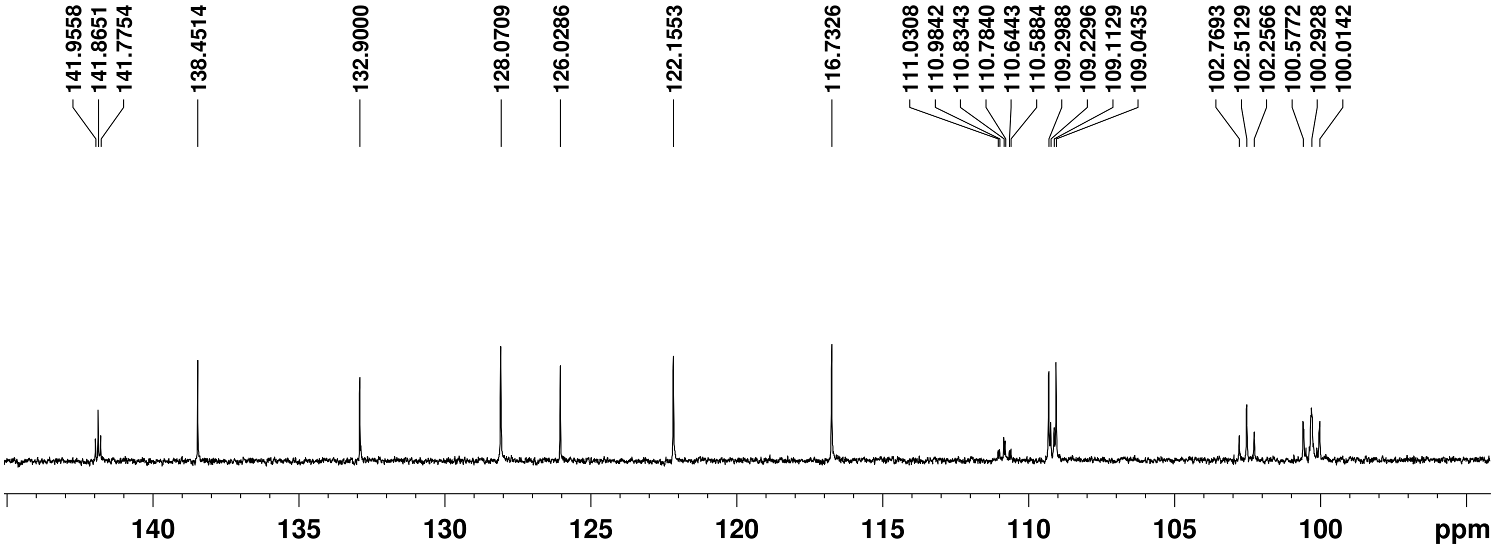

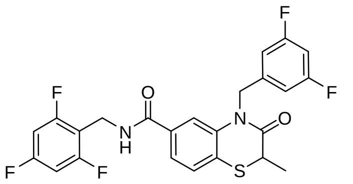

Compound 10 (9i)

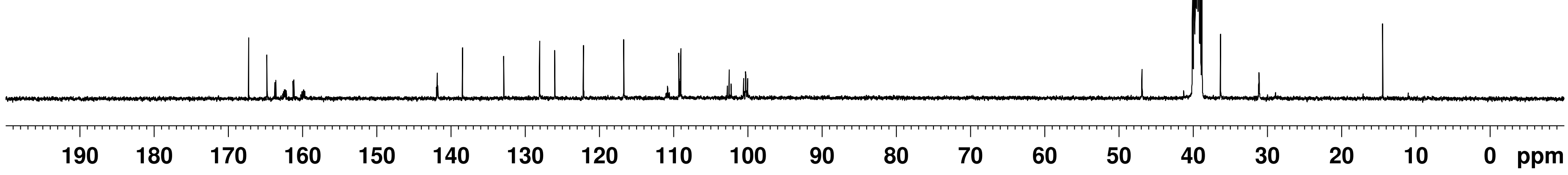

Compound 10 (9i)

TCG Lifesciences Private Limited

Kolkata

```
NAME          CRD-3093
EXPNO               61
PROCNO               1
Date_         20200629
Time              5.28
INSTRUM          spect
PROBHD   5 mm PABBO BB-
PULPROG           jmod
TD               65536
SOLVENT           DMSO
NS               18000
DS                   4
SWH        25252.525 Hz
FIDRES      0.385323 Hz
AQ         1.2976629 sec
RG                2050
DW            19.800 usec
DE              6.50 usec
TE             300.7 K
CNST2      145.0000000
CNST11       1.0000000
D1        2.00000000 sec
D20       0.00689655 sec
TD0                  1

======== CHANNEL f1 ========
NUC1               13C
P1                6.75 usec
P2               13.50 usec
PL1               0.00 dB
SFO1       100.6404331 MHz

======== CHANNEL f2 ========
CPDPRG2        waltz16
NUC2                1H
PCPD2            80.00 usec
PL2               0.00 dB
PL12             13.45 dB
SFO2       400.2016008 MHz
SI               32768
SF         100.6304314 MHz
WDW                 EM
SSB                  0
LB                1.00 Hz
GB                   0
PC                1.40
```

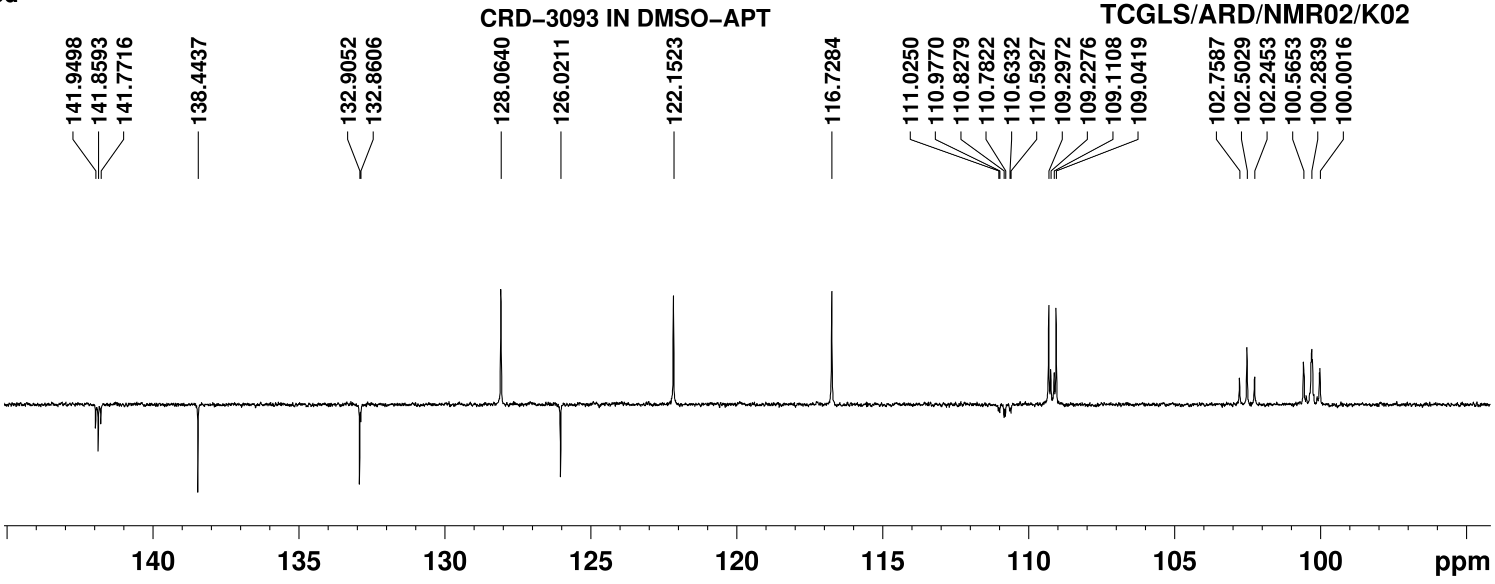

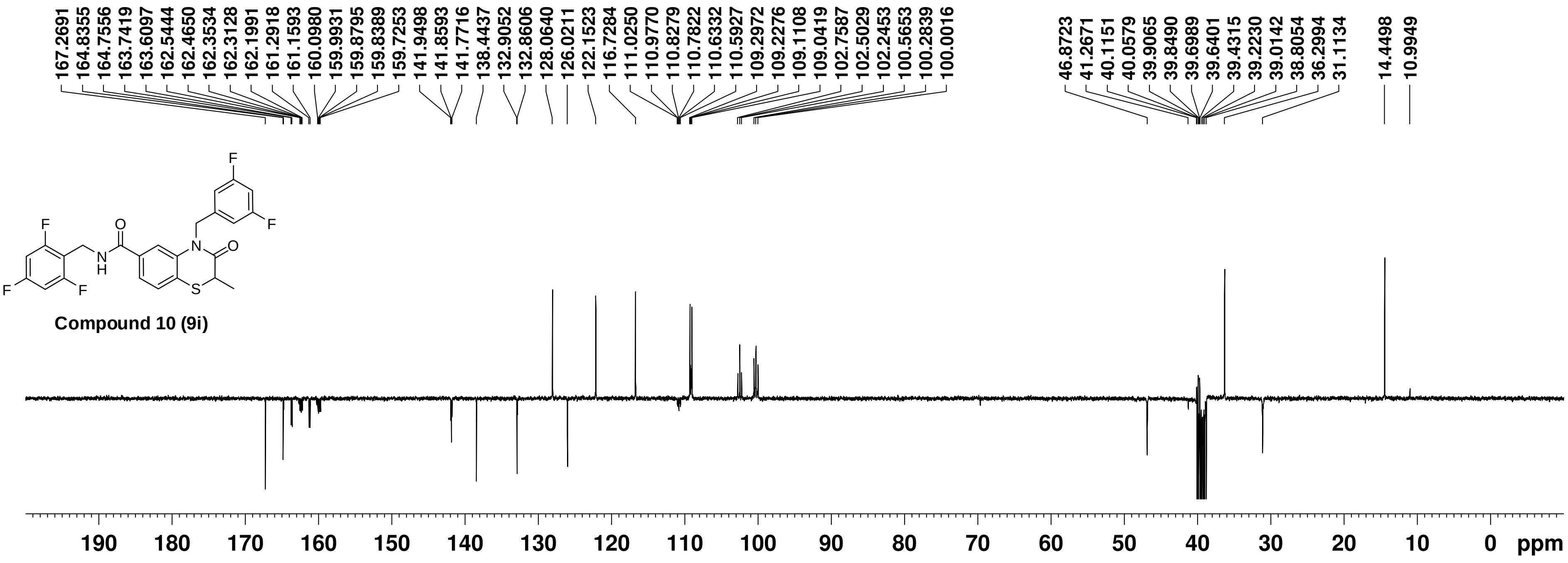

Compound 10 (9i)

Qualitative Analysis Report

Compound 10 (9i)

**Data Filename**	AS-CRD-3093.d	**Sample Name**	AS-CRD-3093
**Sample Type**	Sample	**Position**	Vial 67
**Instrument Name**	Instrument 1	**User Name**	
**Acq Method**	Direct Mass-2017.m	**Acquired Time**	6/16/2020 12:20:44 PM
**IRM Calibration Status**	Some Ions Missed	**DA Method**	Default.m
**Comment**			

**Sample Group**		**Info.**
**Acquisition SW Version**	6200 series TOF/6500 series Q-TOF B.05.00 (B5042.0)	

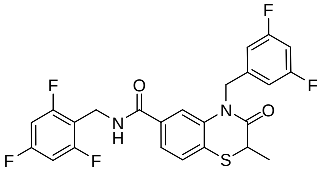

Compound 10 (9i)

Chemical Formula: $C_{24}H_{17}F_5N_2O_2S$

Exact Mass: 492.0931

User Chromatograms

Fragmentor Voltage 118 **Collision Energy** 0 **Ionization Mode** ESI

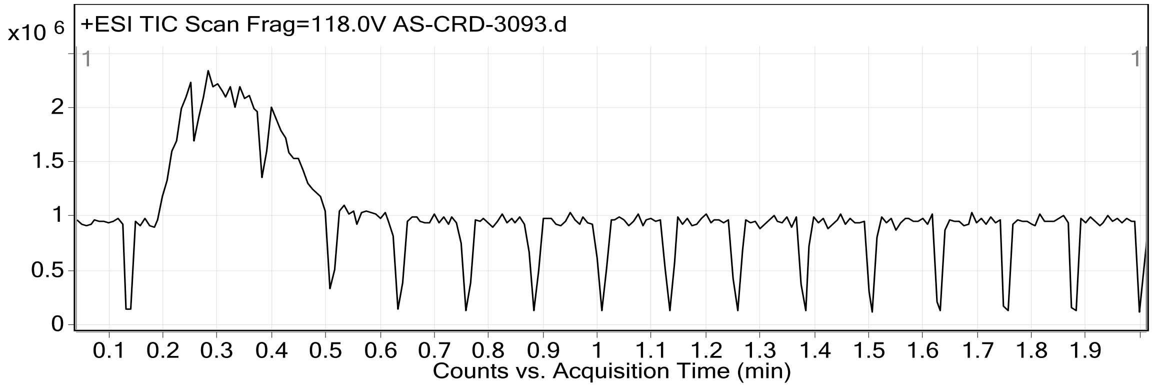

User Spectra

**Fragmentor Voltage**	**Collision Energy**	**Ionization Mode**
118	0	ESI

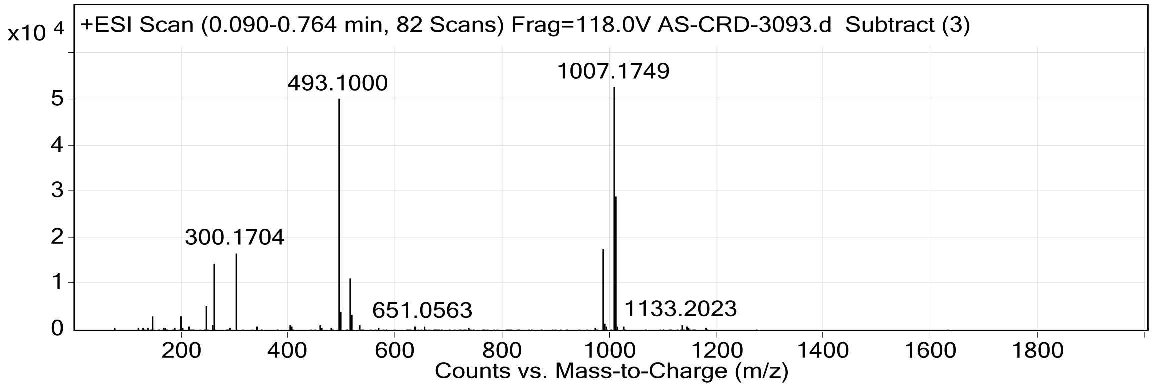

Peak List

m/z	z	Abund
260.1367		14672.59
300.1704	1	16790.25
493.1	1	50299.2
494.1032	1	13691.62
515.0819	1	11449.09
985.1925	1	17789.87

Qualitative Analysis Report

986.1958	1	9828.53
1007.1749	1	52891.24
1008.1778	1	29033.69
1009.1771	1	12747.23

Compound 10 (9i)

Compounds

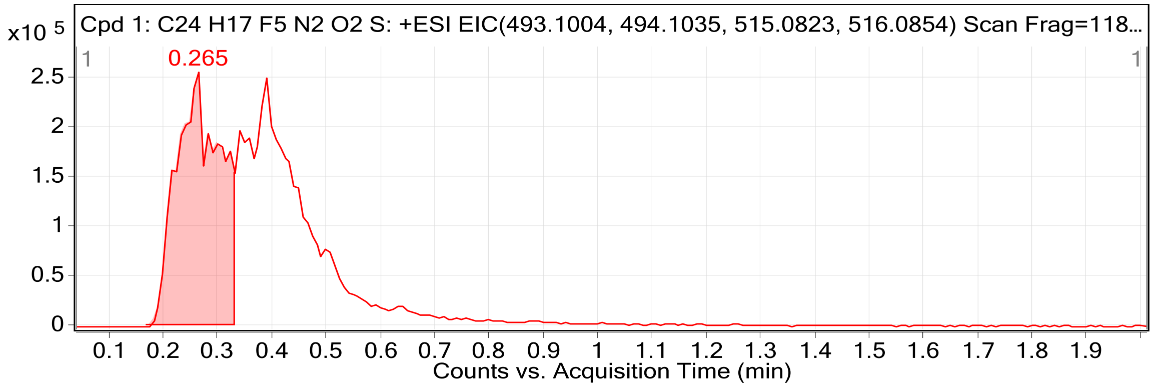

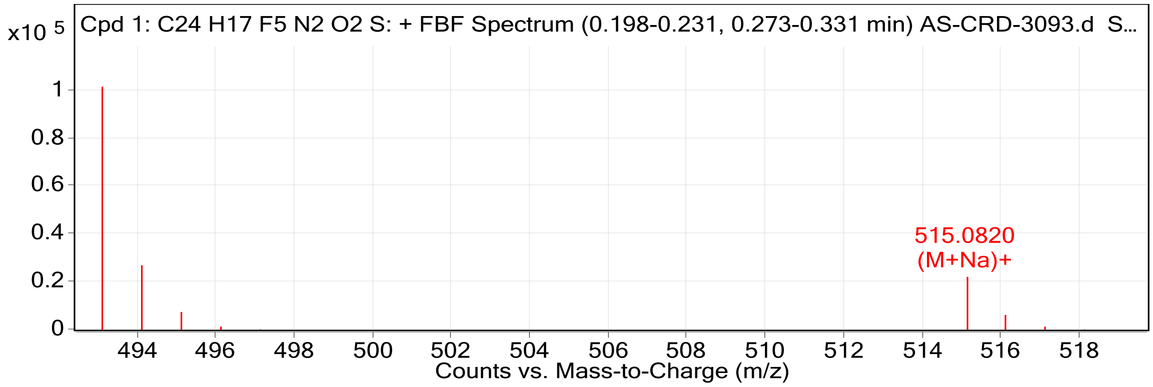

Peak List

m/z	z	Abund	Formula	Ion
493.1001	1	101776.13	C24H18F5N2O2S	(M+H)+
494.1031	1	27249.13	C24H18F5N2O2S	(M+H)+
495.1006	1	7807.96	C24H18F5N2O2S	(M+H)+
496.1001	1	1639.92	C24H18F5N2O2S	(M+H)+
497.1012	1	342.97	C24H18F5N2O2S	(M+H)+
515.082	1	22766.25	C24H17F5N2NaO2S	(M+Na)+
516.0843	1	6425.29	C24H17F5N2NaO2S	(M+Na)+
517.0831	1	1901.34	C24H17F5N2NaO2S	(M+Na)+
518.0835	1	482.97	C24H17F5N2NaO2S	(M+Na)+
519.0784	1	35.57	C24H17F5N2NaO2S	(M+Na)+

Compound 11 (9j)

CHEMBIOTEK, A TCG Lifesciences Private Limited

Kolkata

CR240–CA187–119–P2_GB–CA218–145 IN DMSO

TCGLS/ARD/NMR01/K01

Current Data Parameters
NAME CR240–CA187–119–P2_GB–CA218–145
EXPNO 10
PROCNO 1

F2 – Acquisition Parameters
Date_ 20170428
Time 13.25
INSTRUM spect
PROBHD 5 mm DUL 13C–1
PULPROG zg30
TD 24036
SOLVENT DMSO
NS 32
DS 0
SWH 8012.820 Hz
FIDRES 0.333367 Hz
AQ 1.4998964 sec
RG 45.2
DW 62.400 usec
DE 6.50 usec
TE 300.0 K
D1 1.00000000 sec
TD0 1

======== CHANNEL f1 ========
NUC1 1H
P1 14.50 usec
PL1 –1.00 dB
PL1W 9.92955208 W
SFO1 400.1024654 MHz

F2 – Processing parameters
SI 16384
SF 400.1000041 MHz
WDW EM
SSB 0
LB 0.30 Hz
GB 0
PC 1.00

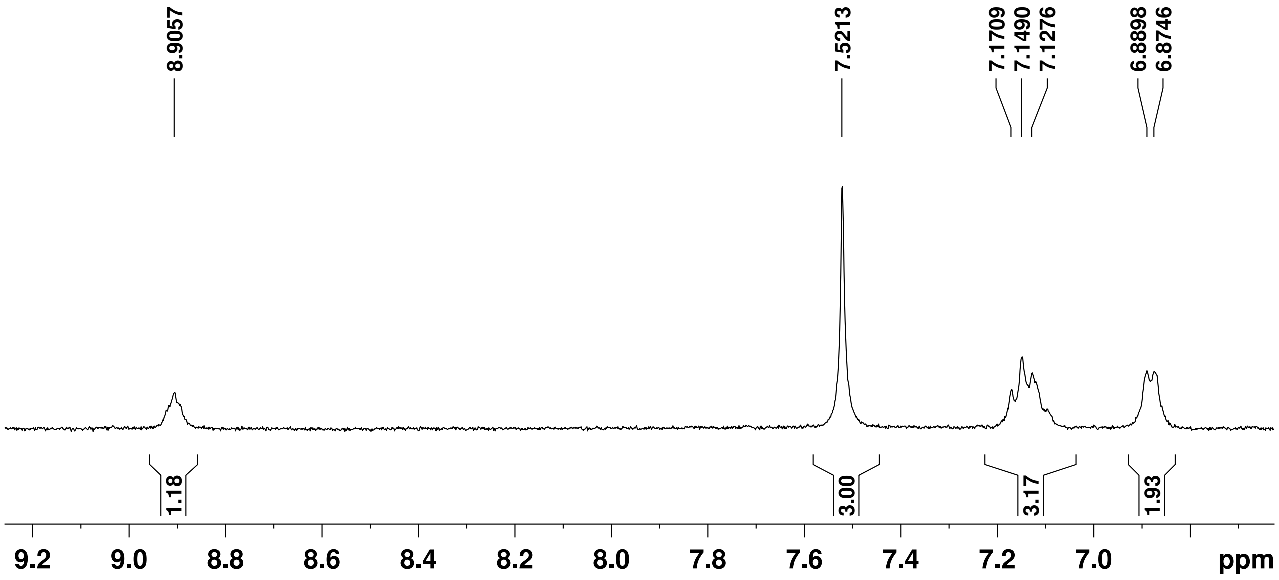

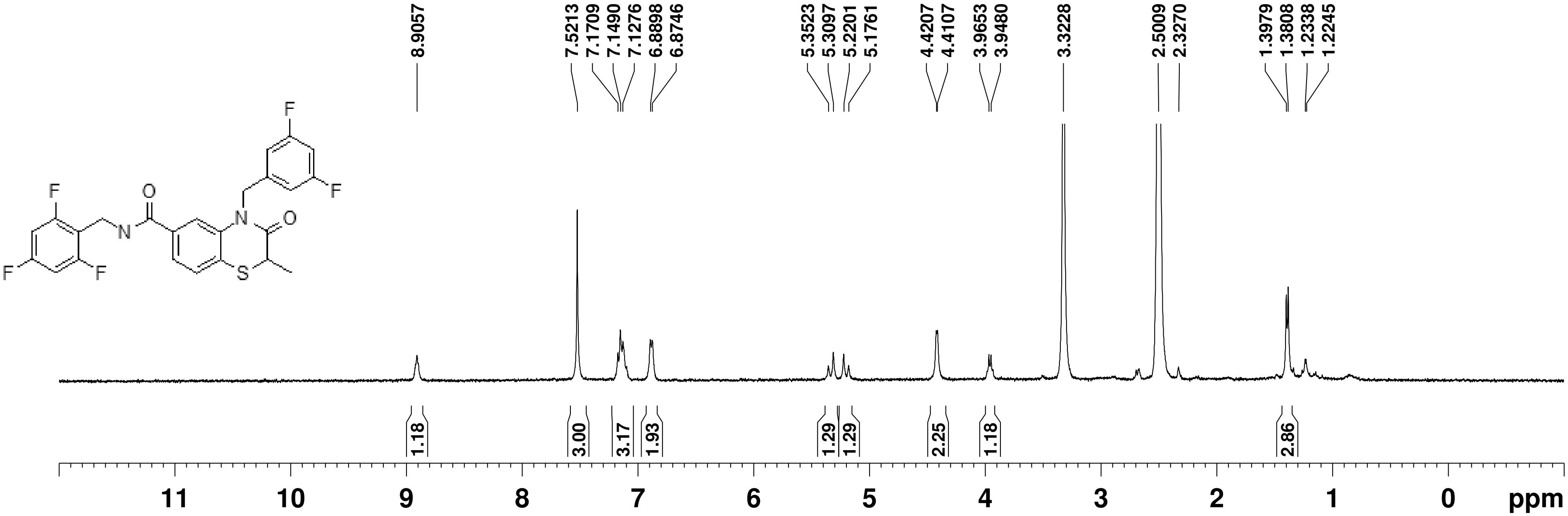

Compound 11 (9j)

BATCH NO. **CHEMBIOTEK,TCG LIFESCIENCES ENTERPRISE** **28-Apr-201714:01:43**
CR240-CA187-119-P2_GB-CA218-145 **KOLKATA,INDIA**
Inst. No.-TCGLS/ARD/LCMS17/K71 **METHOD: MONI-CV25 (HCOOH:ACN) 3min**

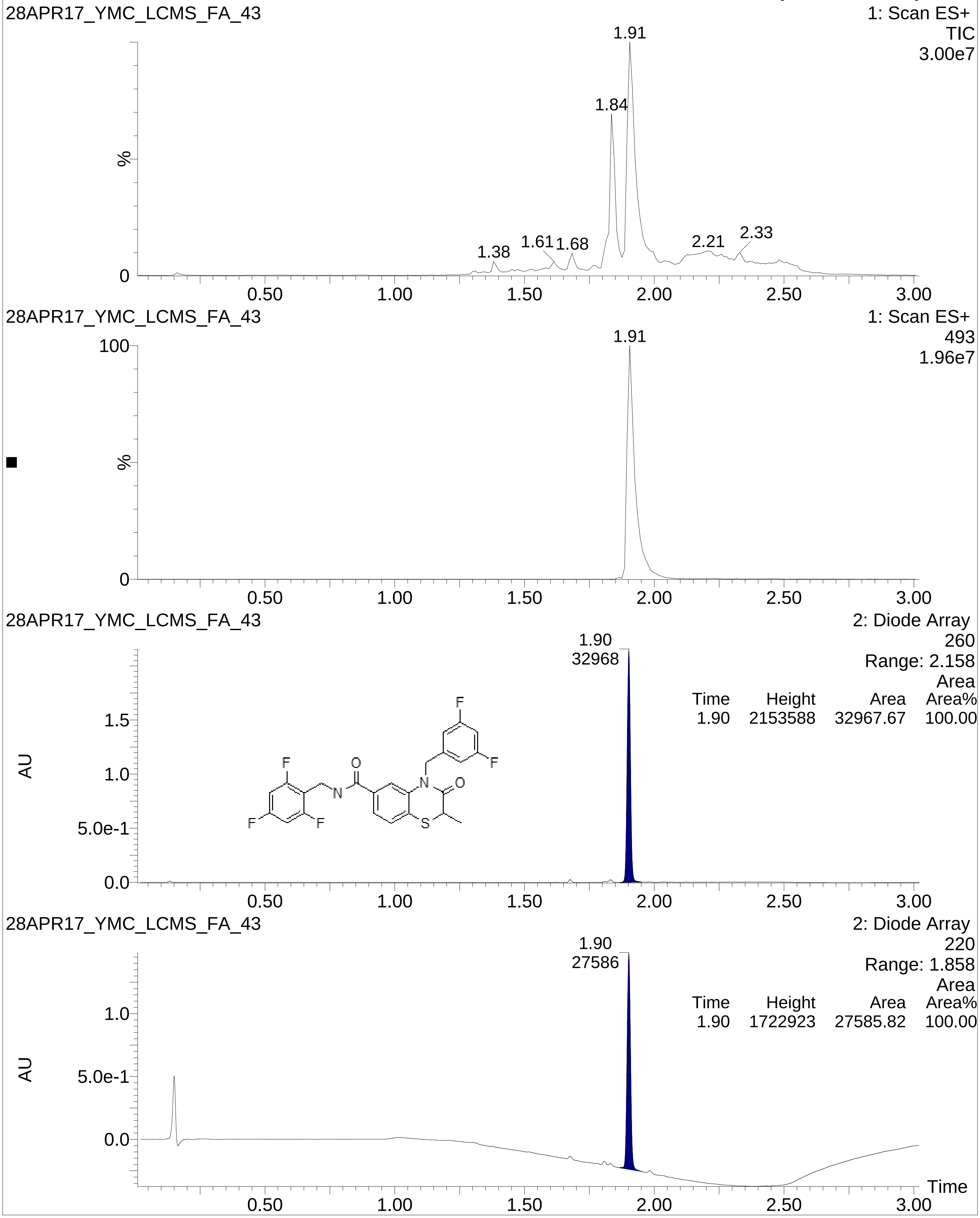

Compound 11 (9j)

BATCH NO. **CHEMBIOTEK,TCG LIFESCIENCES ENTERPRISE** **28-Apr-201714:01:43**

CR240-CA187-119-P2_GB-CA218-145 **KOLKATA,INDIA**

Inst.No.-TCGLS/ARD/LCMS17/K71

28APR17_YMC_LCMS_FA_43 189 (1.906) Cm (189)

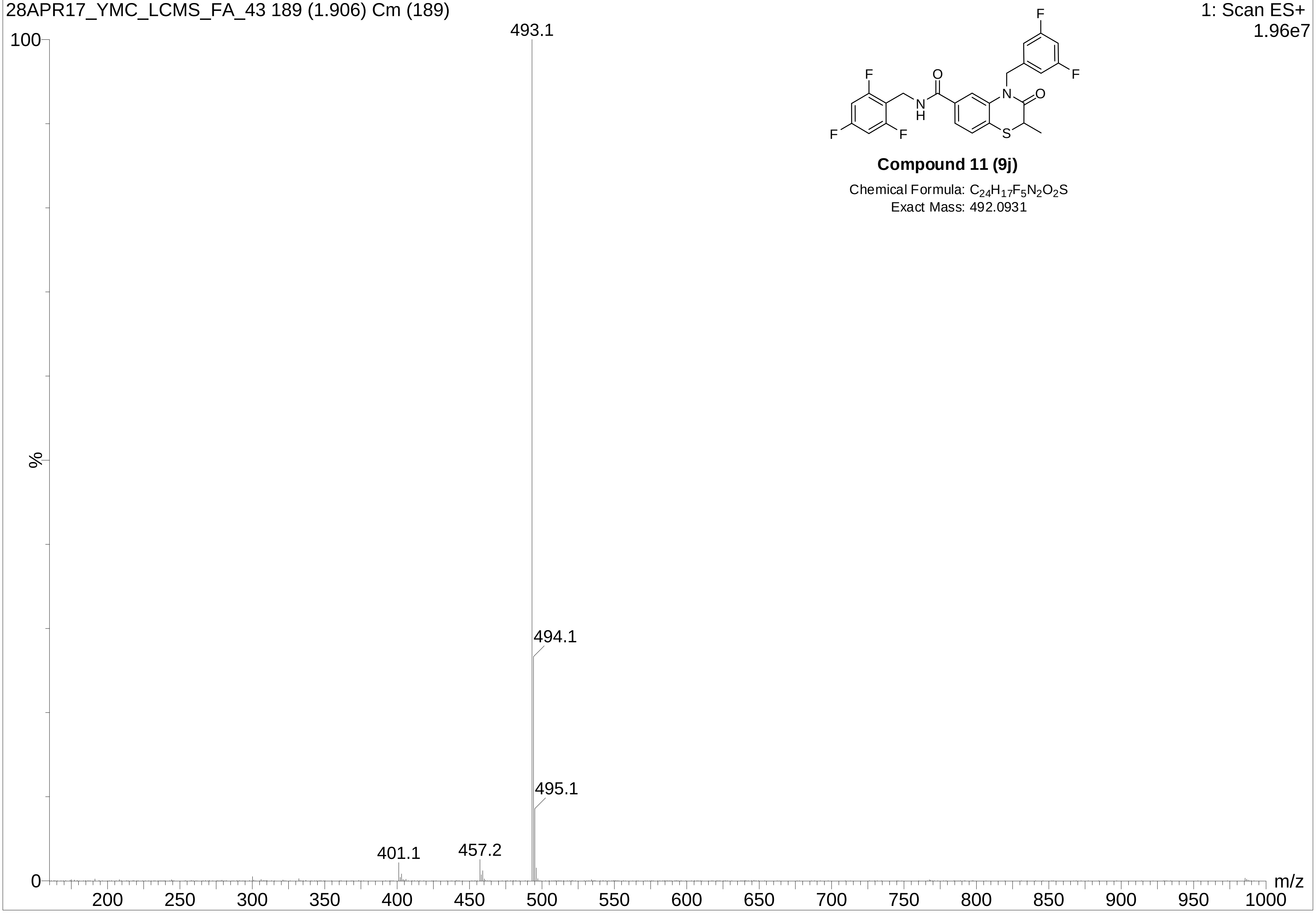

Compound 11 (9j)

Sample Name :CR240-CA187-119-P2_GB-CA218-145

Seq Line-> 13

Location : Vial 84

Acq. Operator : SOUMEN   Inj. No. : 1

Injection Date : 5/2/2017 4:52:00 PM   Inj. Vol. : 12 µl

Acq. Method : C:\Chem32\1\DATA\MAY-2017\020517 2017-05-02 11-01-07->

Analysis Method : C:\CHEM32\1\METHODS\WASH-2.M

Last Changed : Tue, 7. Mar. 2017, 06:37:19 pm

Sample ID : CR240-CA187-119-P2_GB-CA218-145

Column Name : Chiralcel OD-H (4.6 x 250 mm),5µ
ARD/K/7323
Mobile Phase : Hexane/EtOH/DEA : 80/20/0.1
Flow Rate : 1.0 ml/min
Solubility : MeOH

Ref:- SP/02.05.17/6

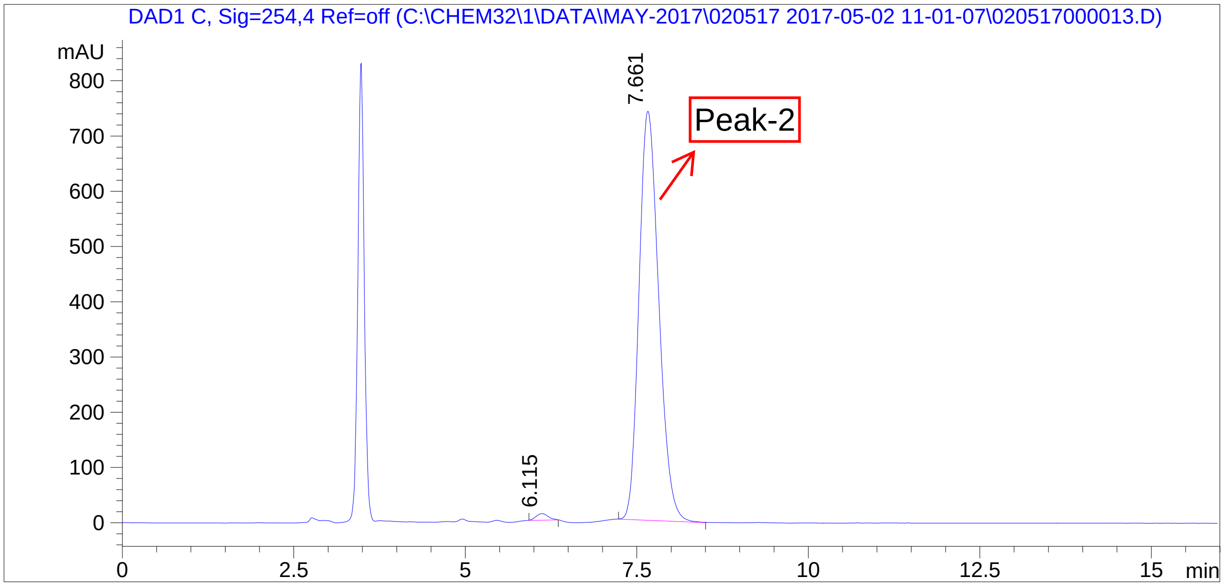

Signal 1: DAD1 C, Sig=254,4 Ref=off

Peak #	RT [min]	Area	Area %
1	6.12	146.12	0.98
2	7.66	14837.97	99.02

*** End of Report ***

Compound 11 (9j)

TCG Lifesciences Private Limited
Kolkata

VK–CA298–138–PEAK–2 IN DMSO–13C

TCGLS/ARD/NMR02/K02

NAME	VK–CA298–138–PEAK–2
EXPNO	60
PROCNO	1
Date_	20200703
Time	15.38 h
INSTRUM	spect
PROBHD	Z163739_0162 (
PULPROG	zgpg30
TD	65536
SOLVENT	DMSO
NS	1514
DS	4
SWH	24038.461 Hz
FIDRES	0.733596 Hz
AQ	1.3631988 sec
RG	208.6
DW	20.800 usec
DE	6.50 usec
TE	298.5 K
D1	2.00000000 sec
D11	0.03000000 sec
TD0	1
SFO1	100.6328888 MHz
NUC1	13C
P0	2.67 usec
P1	8.00 usec
SI	32768
SF	100.6228827 MHz
WDW	EM
SSB	0
LB	1.00 Hz
GB	0
PC	1.40

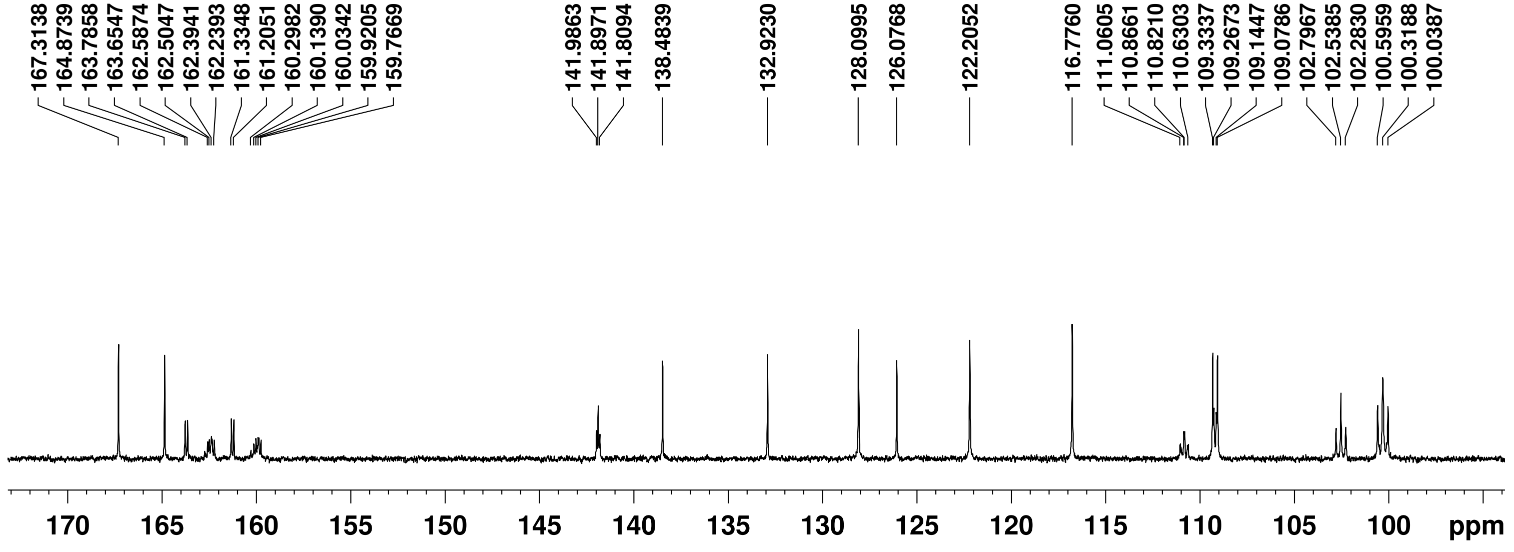

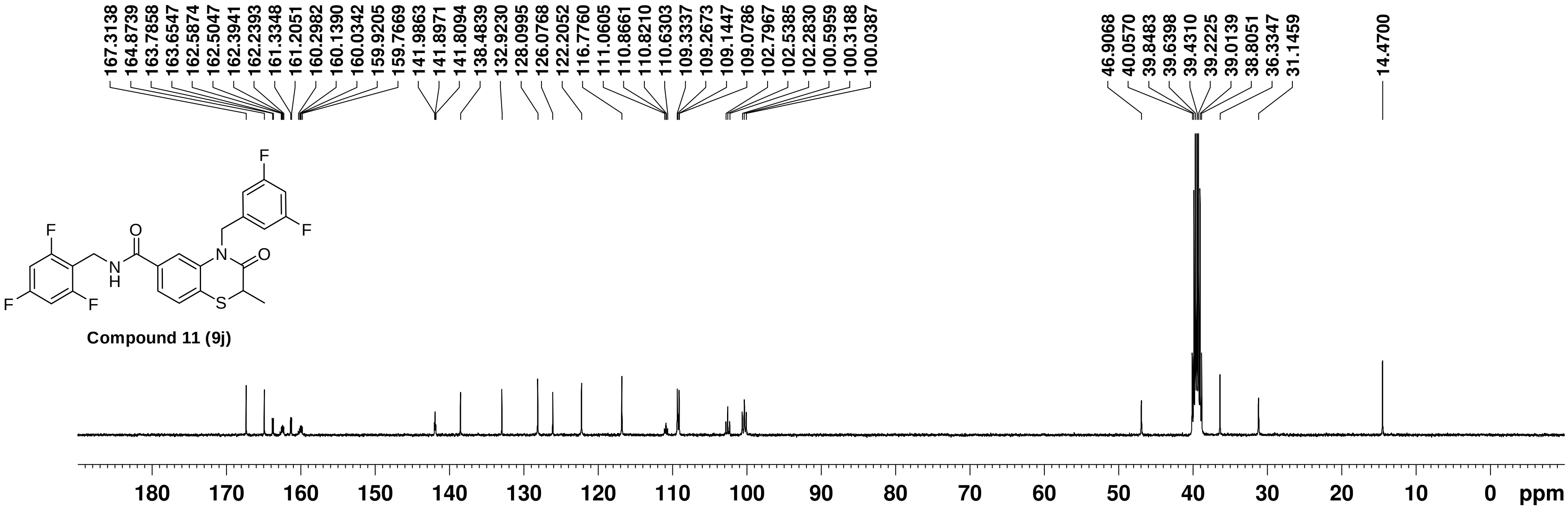

Compound 11 (9j)

Compound 11 (9j)

TCG Lifesciences Private Limited
Kolkata

VK–CA298–138–PEAK–2 IN DMSO–APT

TCGLS/ARD/NMR02/K02

NAME	VK–CA298–138–PEAK–2
EXPNO	61
PROCNO	1
Date_	20200703
Time	16.35 h
INSTRUM	spect
PROBHD	Z163739_0162 (
PULPROG	jmod
TD	65536
SOLVENT	DMSO
NS	1000
DS	4
SWH	24038.461 Hz
FIDRES	0.733596 Hz
AQ	1.3631988 sec
RG	208.6
DW	20.800 usec
DE	6.50 usec
TE	299.8 K
CNST2	145.0000000
CNST11	1.0000000
D1	2.00000000 sec
D20	0.00689655 sec
TD0	1
SFO1	100.6328888 MHz
NUC1	13C
P1	8.00 usec
P2	16.00 usec
SI	32768
SF	100.6228838 MHz
WDW	EM
SSB	0
LB	1.00 Hz
GB	0
PC	1.40

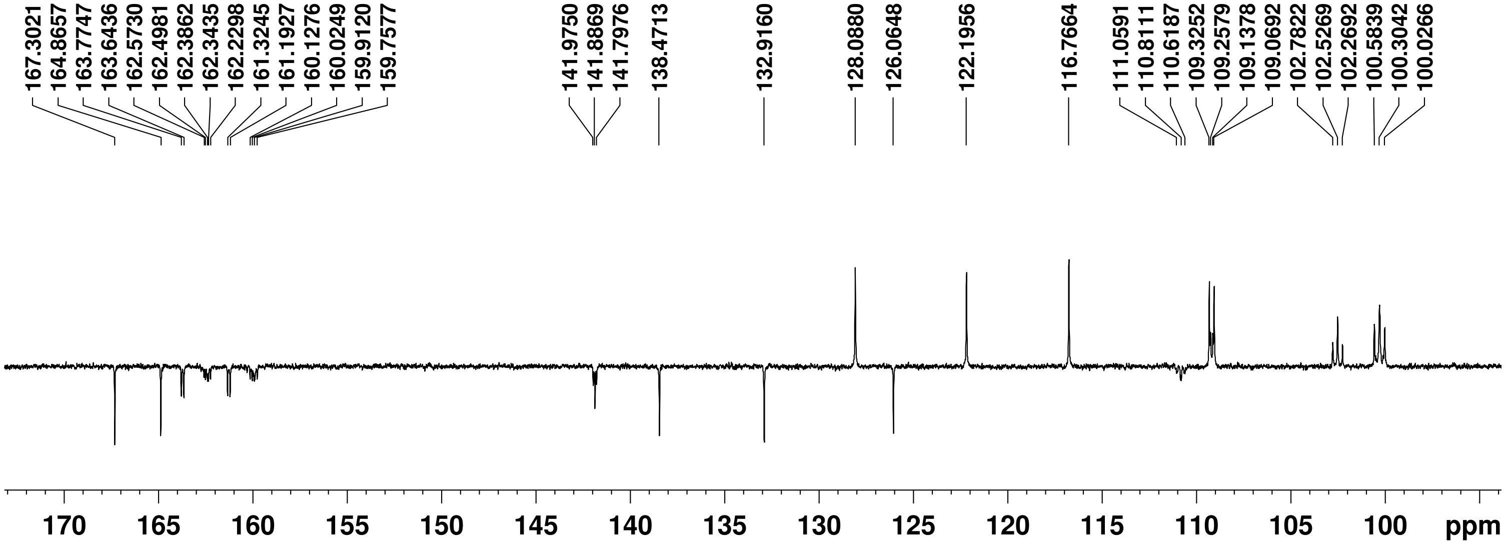

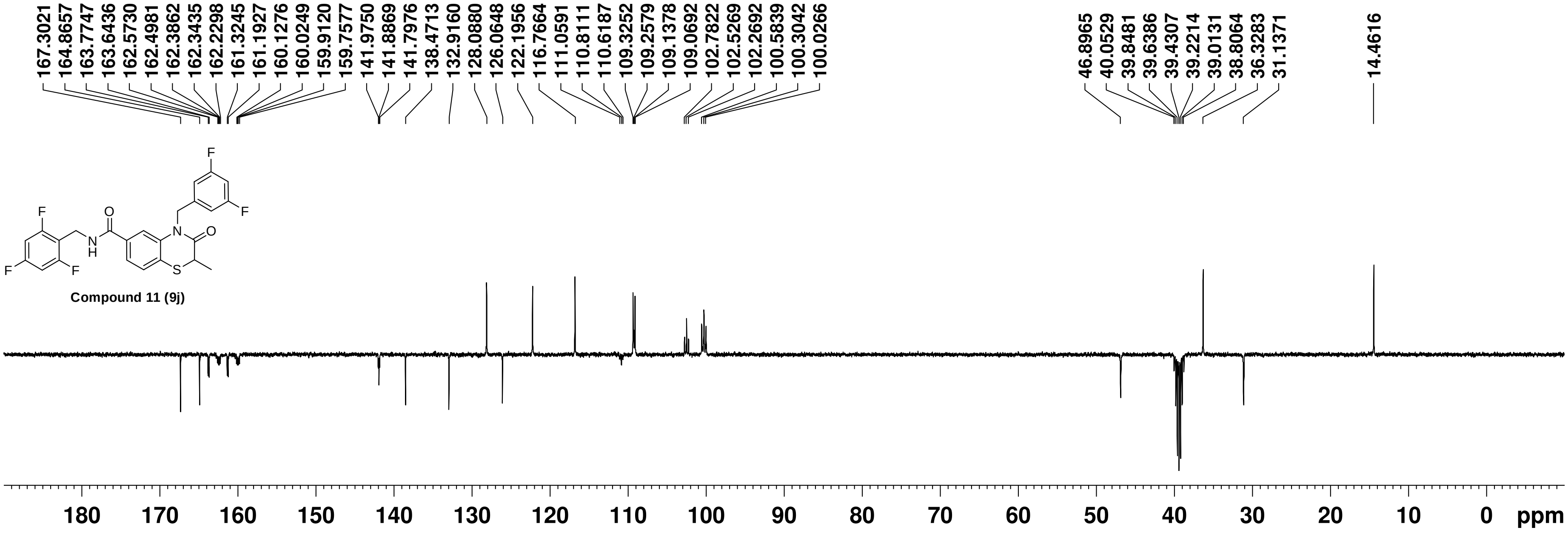

Compound 11 (9j)

Qualitative Analysis Report

Compound 11 (9j)

**Data Filename**	AS-CRD-3094.d	**Sample Name**	AS-CRD-3094
**Sample Type**	Sample	**Position**	Vial 68
**Instrument Name**	Instrument 1	**User Name**	
**Acq Method**	Direct Mass-2017.m	**Acquired Time**	6/16/2020 12:24:21 PM
**IRM Calibration Status**	Some Ions Missed	**DA Method**	Default.m
**Comment**			

**Sample Group**		**Info.**
**Acquisition SW Version**	6200 series TOF/6500 series Q-TOF B.05.00 (B5042.0)	

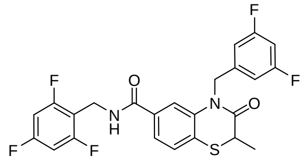

Compound 11 (9j)

Chemical Formula: $C_{24}H_{17}F_5N_2O_2S$

Exact Mass: 492.0931

User Chromatograms

Fragmentor Voltage 118 **Collision Energy** 0 **Ionization Mode** ESI

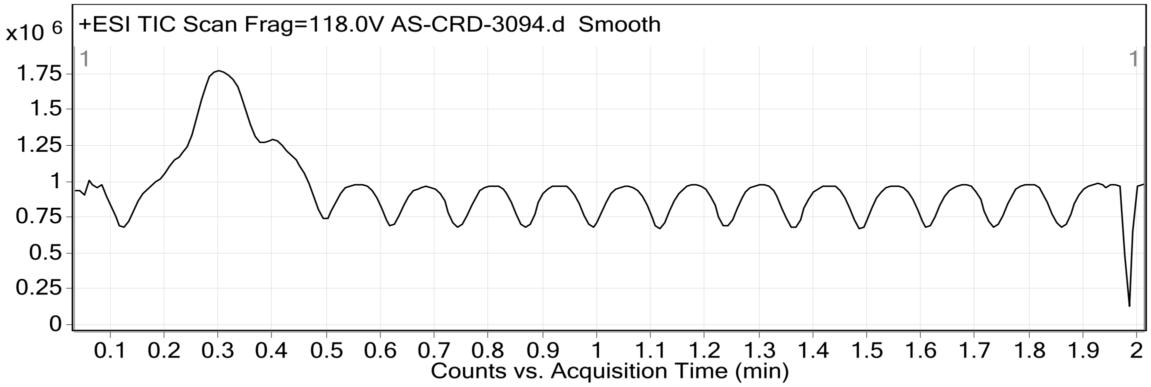

User Spectra

**Fragmentor Voltage**	**Collision Energy**	**Ionization Mode**
118	0	ESI

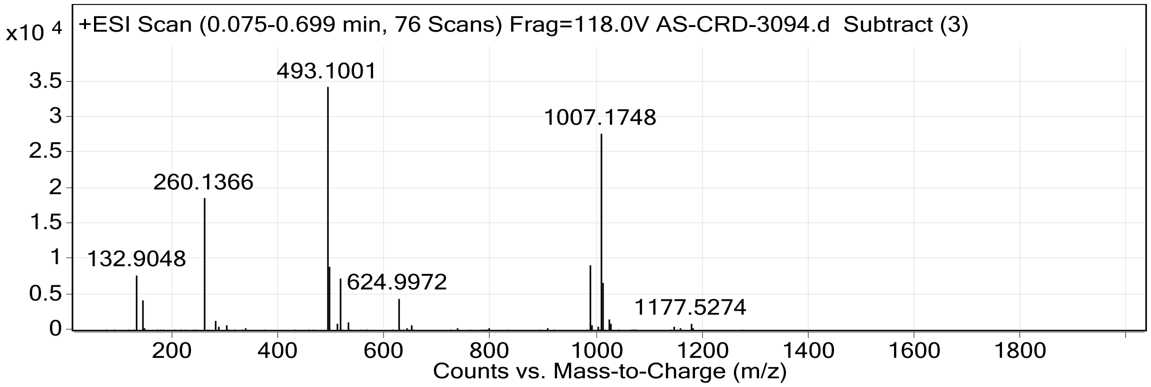

Peak List

m/z	z	Abund
132.9048		7860.23
260.1366	1	18676.36
493.1001	1	34344.59
494.103	1	9119.48
515.082	1	7485.64
985.1929	1	9164.06

986.1959	1	5071.79
1007.1748	1	27795.17
1008.1776	1	15367.84
1009.1767	1	6762.16

Compound 11 (9j)

Compounds

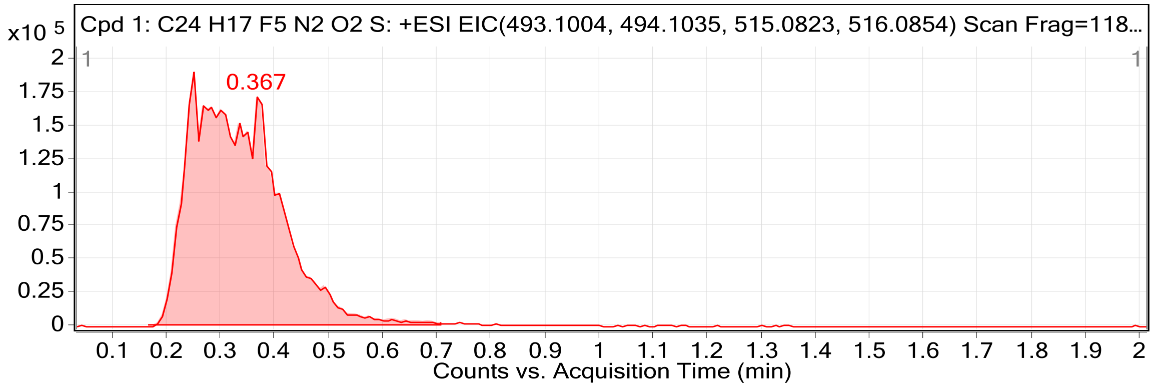

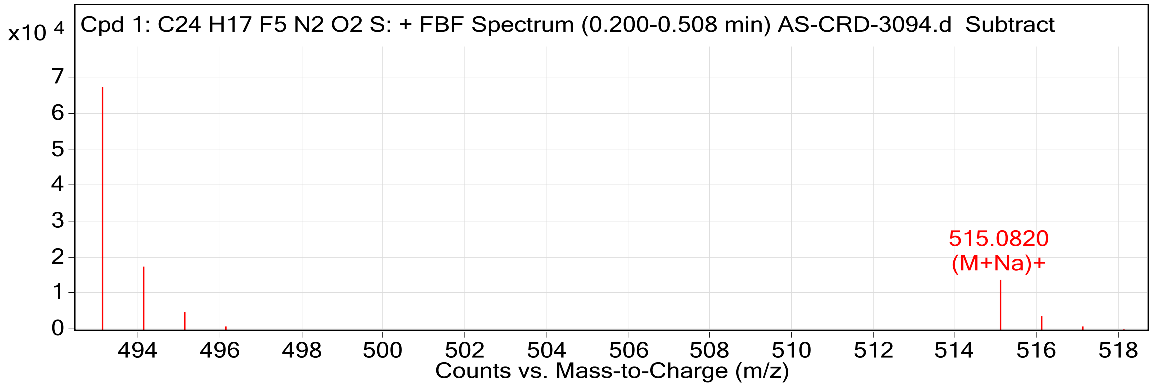

Peak List

m/z	z	Abund	Formula	Ion
493.1	1	67833.19	C24H18F5N2O2S	(M+H)+
494.103	1	17684.59	C24H18F5N2O2S	(M+H)+
495.1007	1	5320.02	C24H18F5N2O2S	(M+H)+
496.1025	1	1105.44	C24H18F5N2O2S	(M+H)+
497.1004	1	180.73	C24H18F5N2O2S	(M+H)+
515.082	1	14291.57	C24H17F5N2NaO2S	(M+Na)+
516.0846	1	3968.45	C24H17F5N2NaO2S	(M+Na)+
517.0832	1	1258.09	C24H17F5N2NaO2S	(M+Na)+
518.0829	1	275.1	C24H17F5N2NaO2S	(M+Na)+

Compound 12 (9k)

VK-CA223-80

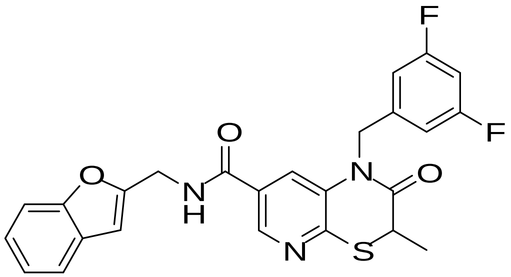

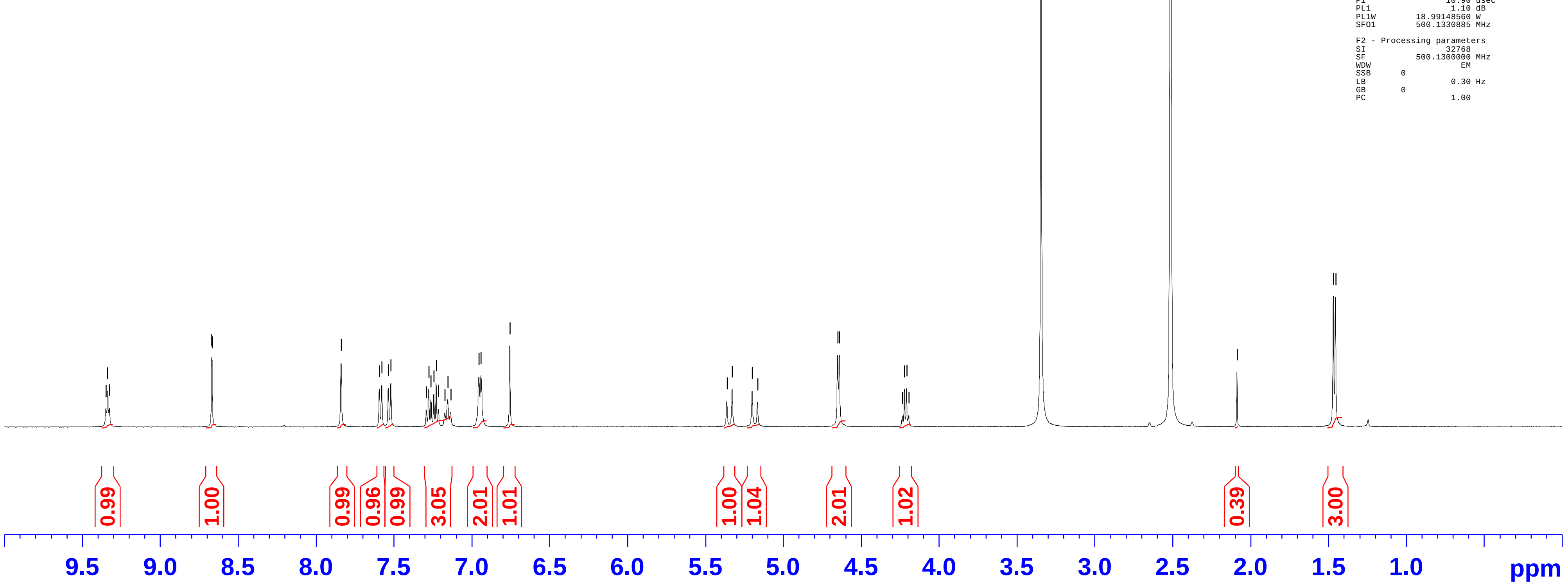


```
Current Data Parameters
NAME                 18-37
EXPNO                   32
PROCNO                   1

F2 - Acquisition Parameters
Date_             20170526
Time                  5.20
INSTRUM              spect
PROBHD   5 mm PABBO BB-
PULPROG               zg30
TD                   65536
SOLVENT               DMSO
NS                      12
DS                       2
SWH             10330.578 Hz
FIDRES           0.157632 Hz
AQ              3.1719425 sec
RG                     203
DW                  48.400 usec
DE                    6.50 usec
TE                   400.4 K
D1             1.00000000 sec
TD0                      1

======== CHANNEL f1 ========
NUC1                    1H
P1                   10.90 usec
PL1                   1.10 dB
PL1W           18.99148560 W
SFO1           500.1330885 MHz

F2 - Processing parameters
SI                   32768
SF             500.1300000 MHz
WDW                     EM
SSB      0
LB                    0.30 Hz
GB       0
PC                    1.00
```

Compound 12 (9k)

TCG Lifesciences Private Limited

Kolkata

CRD3182B3 IN DMSO−13C

TCGLS/ARD/NMR02/K02

NAME	CRD3182B3
EXPNO	60
PROCNO	1
Date_	20200709
Time	2.14 h
INSTRUM	spect
PROBHD	Z8246_0048 (PH
PULPROG	zgpg30
TD	32768
SOLVENT	DMSO
NS	5000
DS	2
SWH	25252.525 Hz
FIDRES	1.541292 Hz
AQ	0.6488564 sec
RG	64
DW	19.800 usec
DE	6.50 usec
TE	297.2 K
D1	2.00000000 sec
D11	0.03000000 sec
TD0	1
SFO1	100.6152855 MHz
NUC1	13C
P0	3.03 usec
P1	9.10 usec
SI	16384
SF	100.6052822 MHz
WDW	EM
SSB	0
LB	1.00 Hz
GB	0
PC	1.40

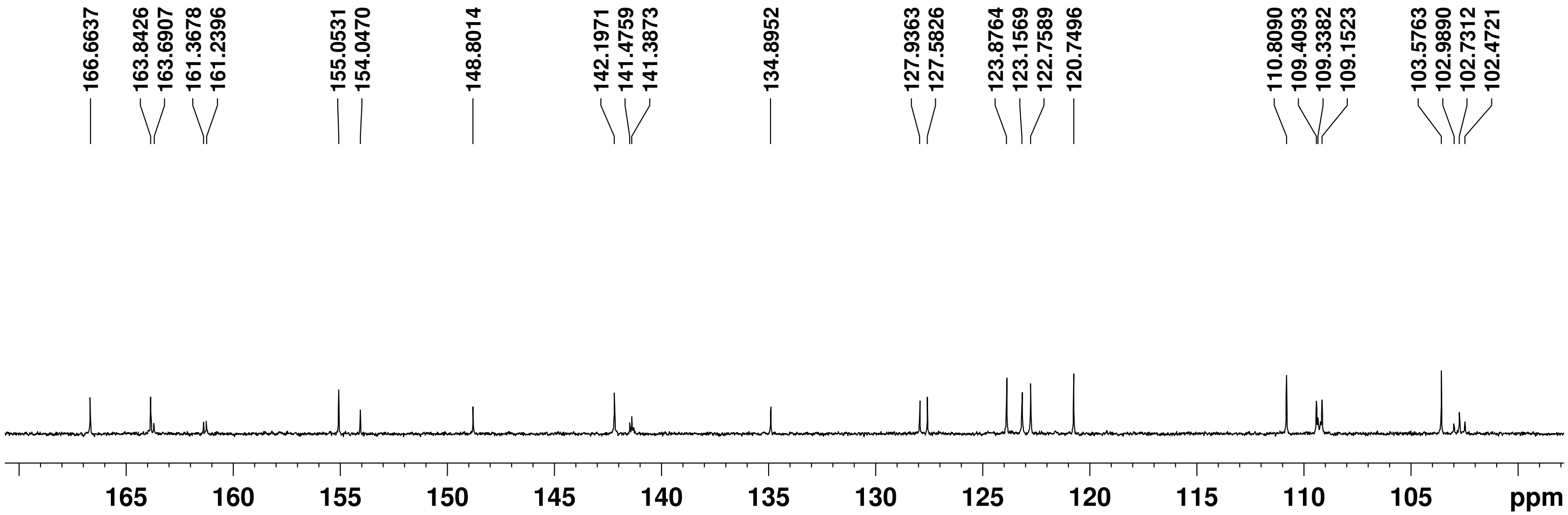

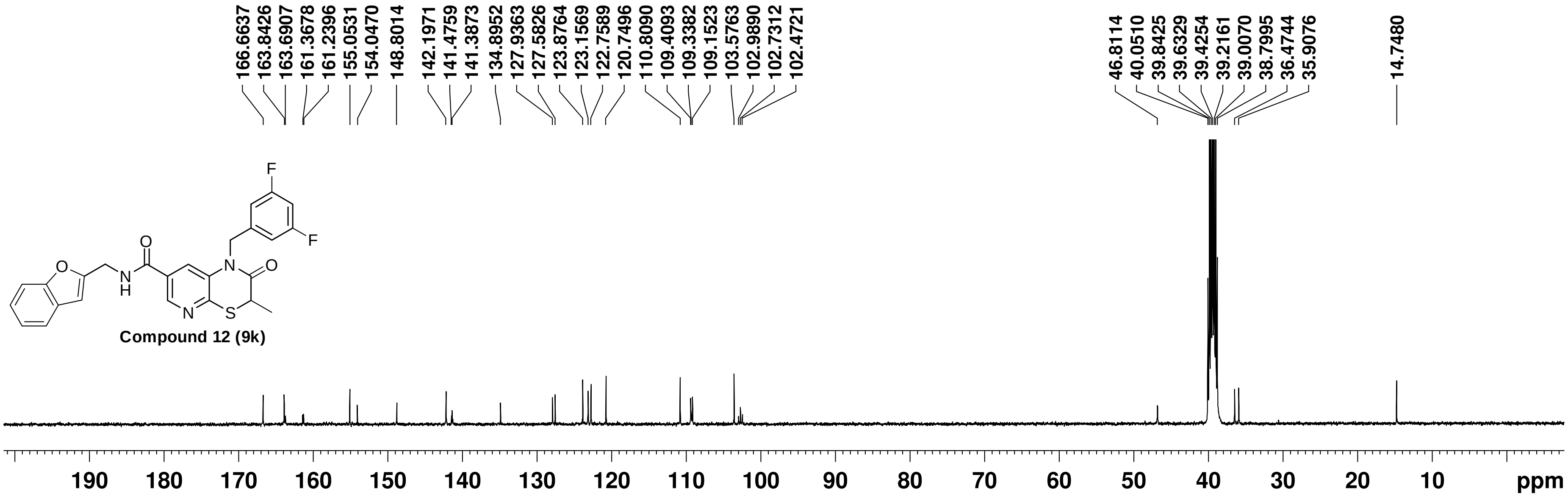

Compound 12 (9k)

Compound 12 (9k)

TCG Lifesciences Private Limited
Kolkata

CRD3182B3 IN DMSO−APT

TCGLS/ARD/NMR02/K02

NAME	CRD3182B3
EXPNO	61
PROCNO	1
Date_	20200709
Time	5.48 h
INSTRUM	spect
PROBHD	Z8246_0048 (PH
PULPROG	jmod
TD	32768
SOLVENT	DMSO
NS	4771
DS	4
SWH	25252.525 Hz
FIDRES	1.541292 Hz
AQ	0.6488564 sec
RG	64
DW	19.800 usec
DE	6.50 usec
TE	297.2 K
CNST2	145.0000000
CNST11	1.0000000
D1	2.00000000 sec
D20	0.00689655 sec
TD0	1
SFO1	100.6152855 MHz
NUC1	13C
P1	9.10 usec
P2	18.20 usec
SI	16384
SF	100.6052824 MHz
WDW	EM
SSB	0
LB	1.00 Hz
GB	0
PC	1.40

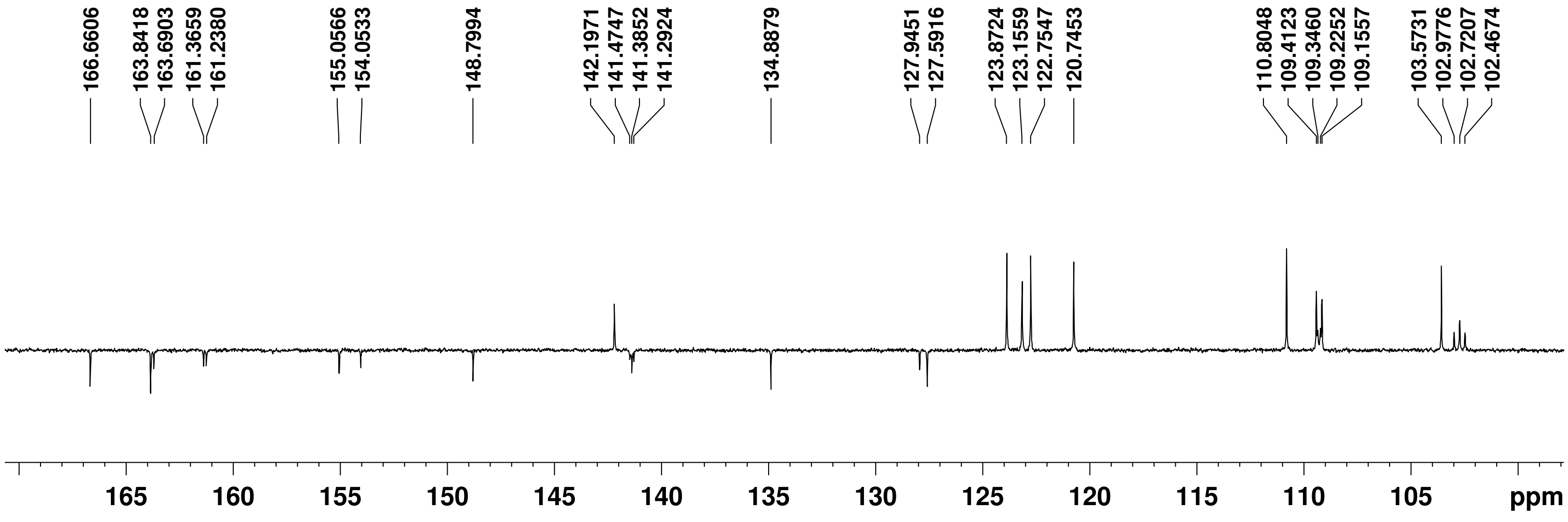

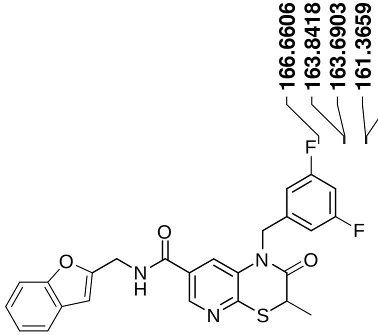

Compound 12 (9k)

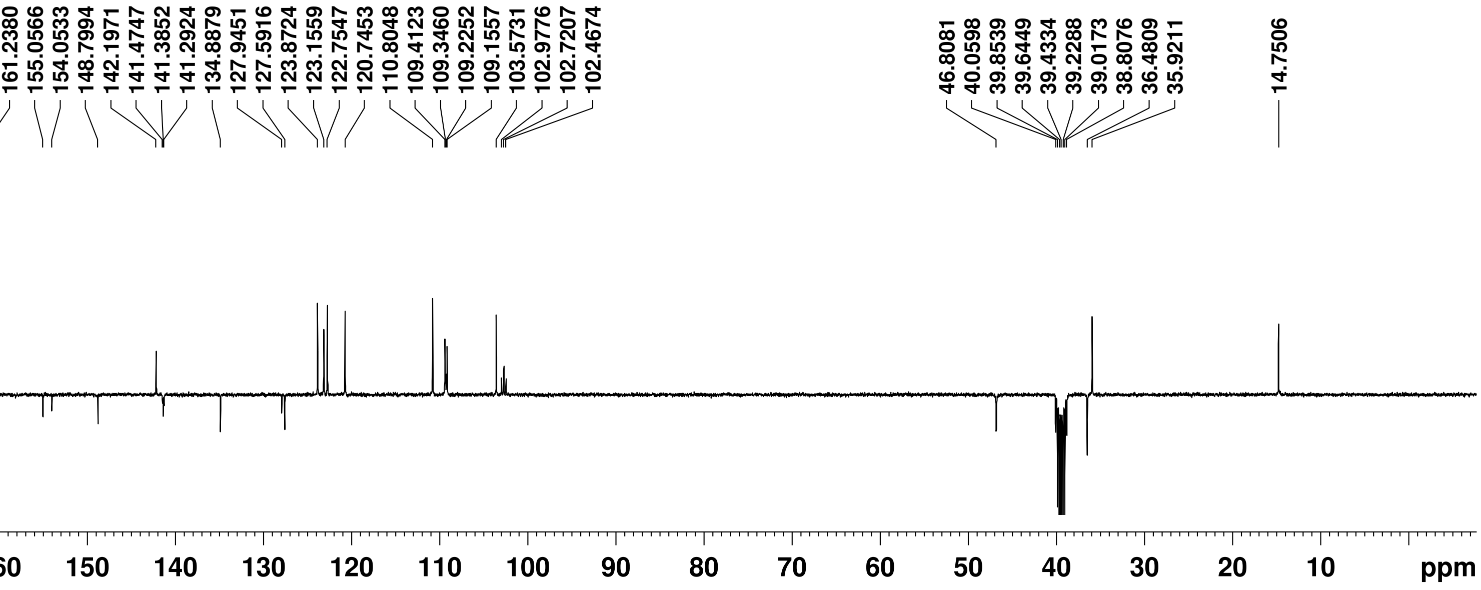

Qualitative Analysis Report

Compound 12 (9k)

**Data Filename**	AS-CRD-3182-B3.d	**Sample Name**	AS-CRD-3182-B3
**Sample Type**	Sample	**Position**	Vial 34
**Instrument Name**	Instrument 1	**User Name**	
**Acq Method**	Direct Mass-2017.m	**Acquired Time**	6/30/2020 9:28:28 PM
**IRM Calibration Status**	Some Ions Missed	**DA Method**	Default.m
**Comment**			

**Sample Group**		**Info.**
**Acquisition SW Version**	6200 series TOF/6500 series Q-TOF B.05.00 (B5042.0)	

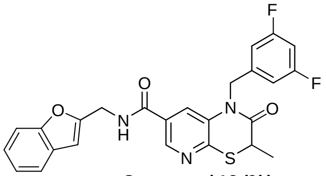

Compound 12 (9k)

Chemical Formula: $C_{25}H_{19}F_2N_3O_3S$
Exact Mass: 479.1115

User Chromatograms

Fragmentor Voltage 118 **Collision Energy** 0 **Ionization Mode** ESI

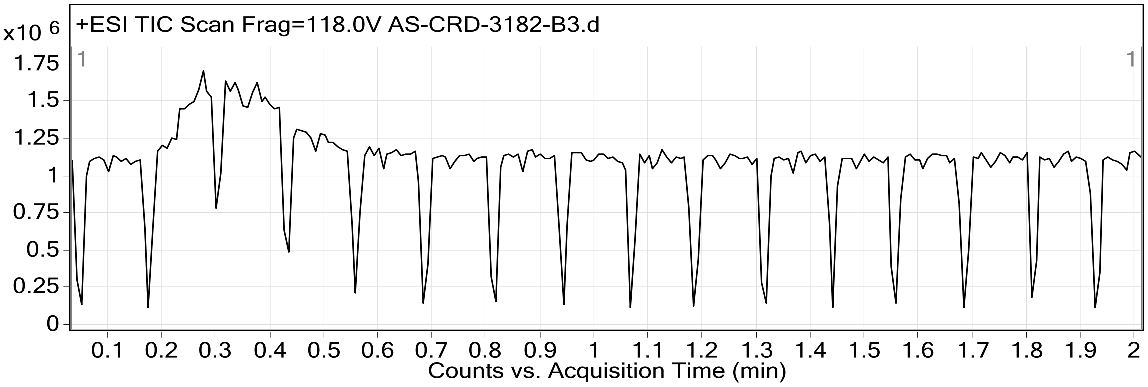

User Spectra

Fragmentor Voltage	Collision Energy	Ionization Mode
118	0	ESI

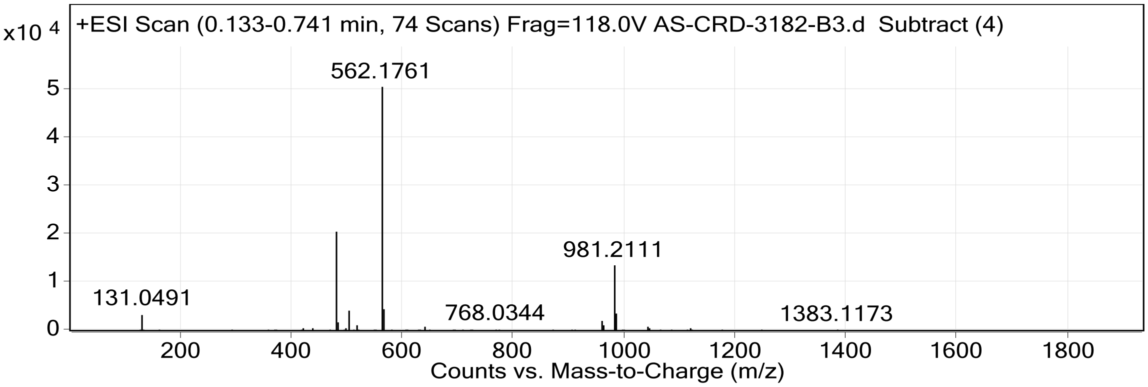

Peak List

m/z	z	Abund
131.0491	1	3471.48
480.1188	1	20453.03
481.1218	1	5954.81
502.1007	1	4183.08
562.1761	1	50510.48
563.1793	1	19675.7

564.1829	1	4617.77
981.2111	1	13475.04
982.2142	1	7824.97
983.2137	1	3602.07

Compound 12 (9k)

Compounds

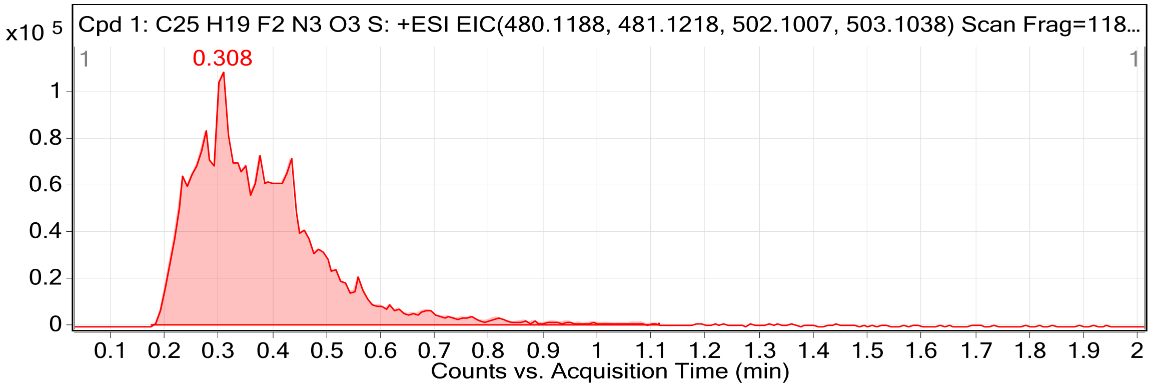

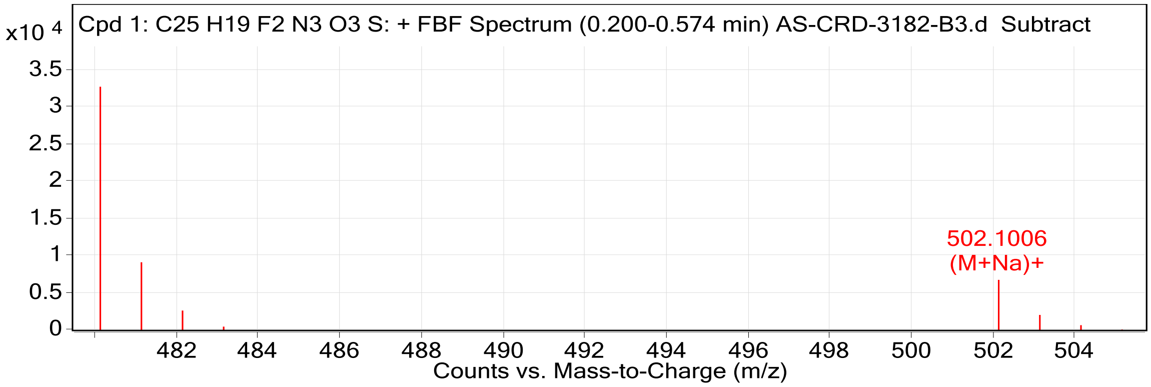

Peak List

m/z	z	Abund	Formula	Ion
480.1188	1	32890.11	C25H20F2N3O3S	(M+H)+
481.1218	1	9280.26	C25H20F2N3O3S	(M+H)+
482.1194	1	2751.34	C25H20F2N3O3S	(M+H)+
483.1199	1	678.93	C25H20F2N3O3S	(M+H)+
502.1006	1	6942.26	C25H19F2N3NaO3S	(M+Na)+
503.1031	1	2083.98	C25H19F2N3NaO3S	(M+Na)+
504.1013	1	708.3	C25H19F2N3NaO3S	(M+Na)+
505.1043	1	104.96	C25H19F2N3NaO3S	(M+Na)+

Compound 12 (9k)

SAMPLE INFORMATION

Sample Name:	VK-CA223-80	Acquired By:	UPLC_MS_01 System
Vial:	1:C,5	Sample Set Name:	SAMPLE_FA
Injection #:	1	Acq. Method Set:	PH HEX_FA_6min
Injection Volume:	0.20 ul	Processing Method:	MASS
Run Time:	6.0 Minutes	Channel Name:	480.4Da
Date Acquired:	25-05-2017 13:57:05 IST	Date Processed:	25-05-2017 17:45:14 IST

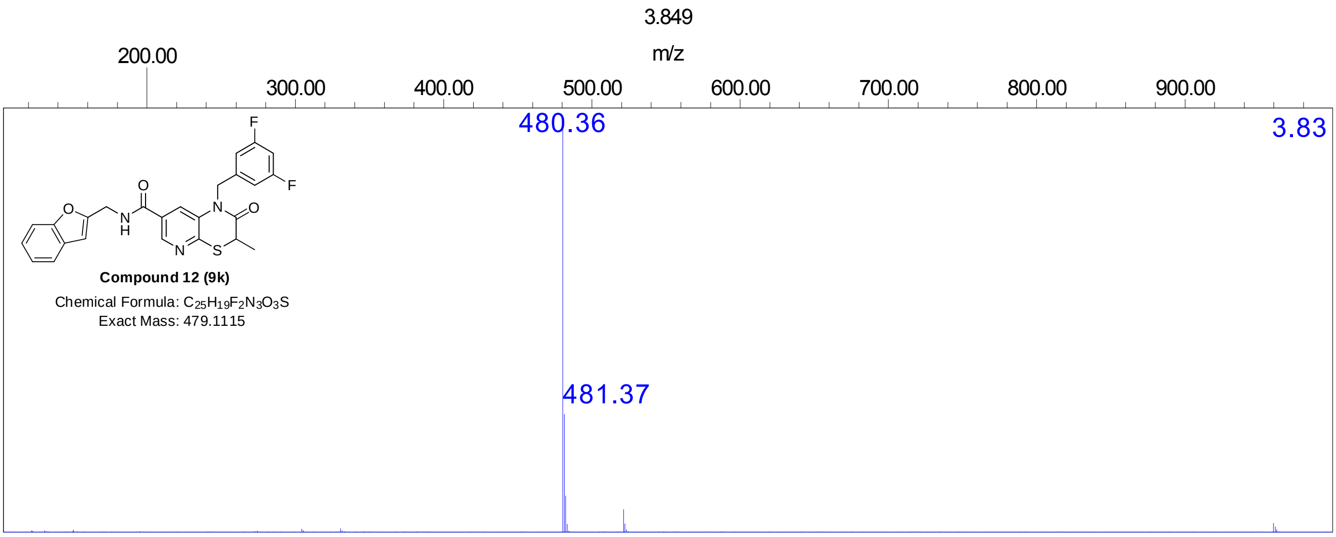

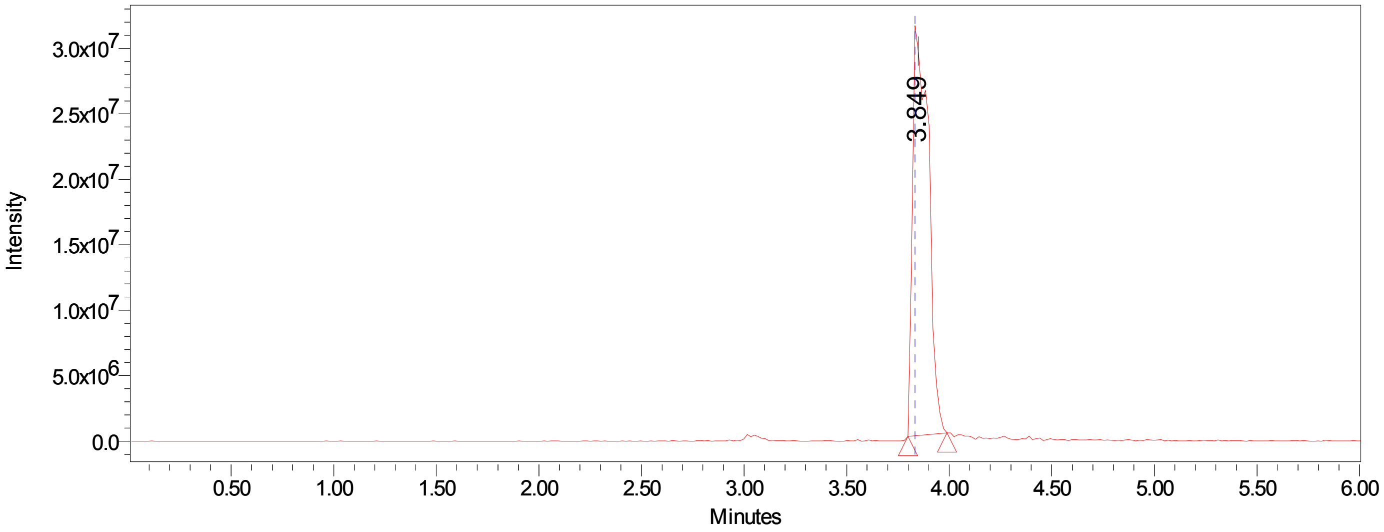

Channel Description 1: 100.00-1000.00 ES+, Centroid, CV=Tune; Processed Channel Descr. W3100 1: MS Scan MS 480.36 m/z Peak Separation: 1.0000 (1: 100.00-1000.00 ES+, Centroid, CV=Tune)

Empower 3 SOFTWARE

UPLC_FA

Compound 12 (9k)

SAMPLE INFORMATION

Sample Name:	VK-CA223-80	Acquired By:	UPLC_MS_01 System
Vial:	1:C,5	Sample Set Name:	SAMPLE_FA
Injection #:	1	Acq. Method Set:	PH HEX_FA_6min
Injection Volume:	0.20 ul	Processing Method:	UPLC
Run Time:	6.0 Minutes	Channel Name:	240.0nm
Date Acquired:	25-05-2017 13:57:05 IST	Date Processed:	25-05-2017 17:44:38 IST
Column	PHENYL HEXYL (2.1x100mm)	Mobile Phase	0.1% FA in Water/ACN

Auto-Scaled Chromatogram

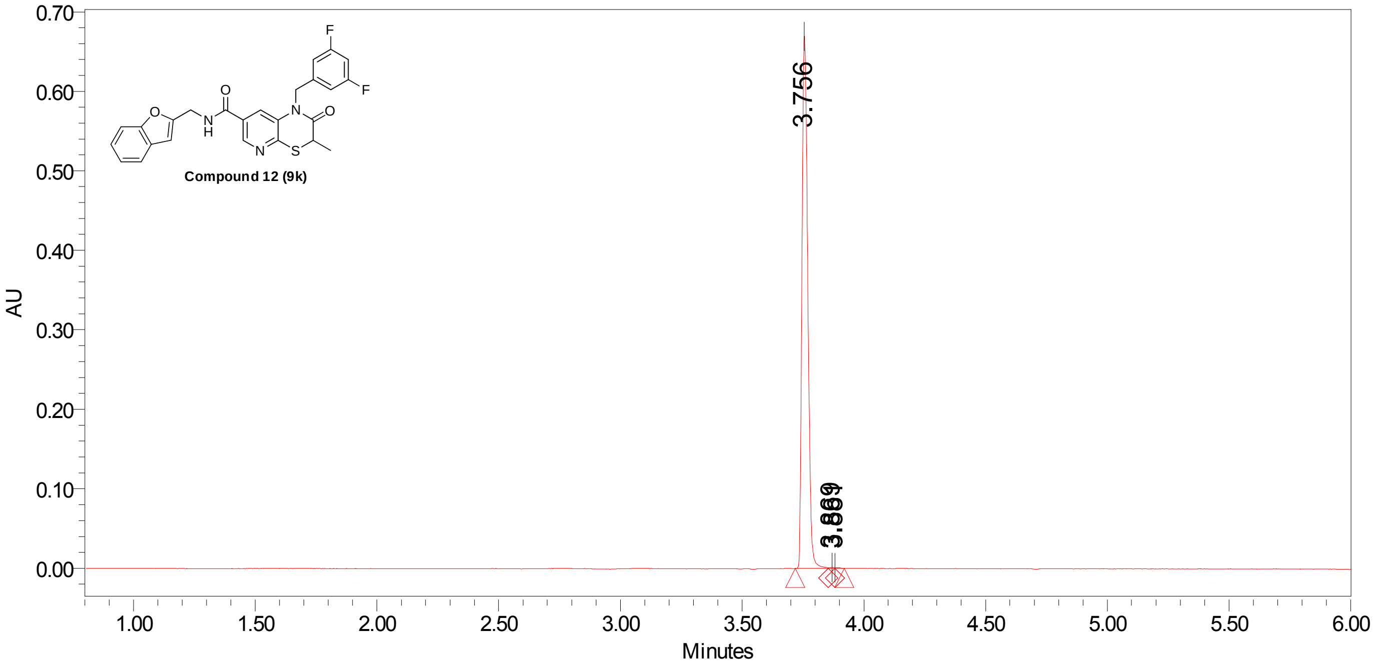

Processed Channel Descr. PDA 240.0 nm (PDA Spectrum (210-400)nm) Blank Subtracted from BLANK, Vial 1:F,8 Inj. 1

Peak Results

	Name	RT	Area	% Area	Height
1		3.756	1113123	99.79	669699
2		3.869	1422	0.13	1019
3		3.881	909	0.08	624

Compound 13 (9h)

VK-CA204-99

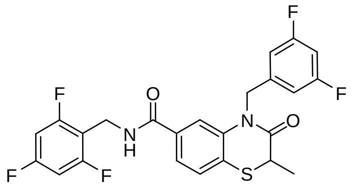

Compound 13 (9h)

Chemical Formula: $C_{24}H_{17}F_5N_2O_2S$

Exact Mass: 492.0931

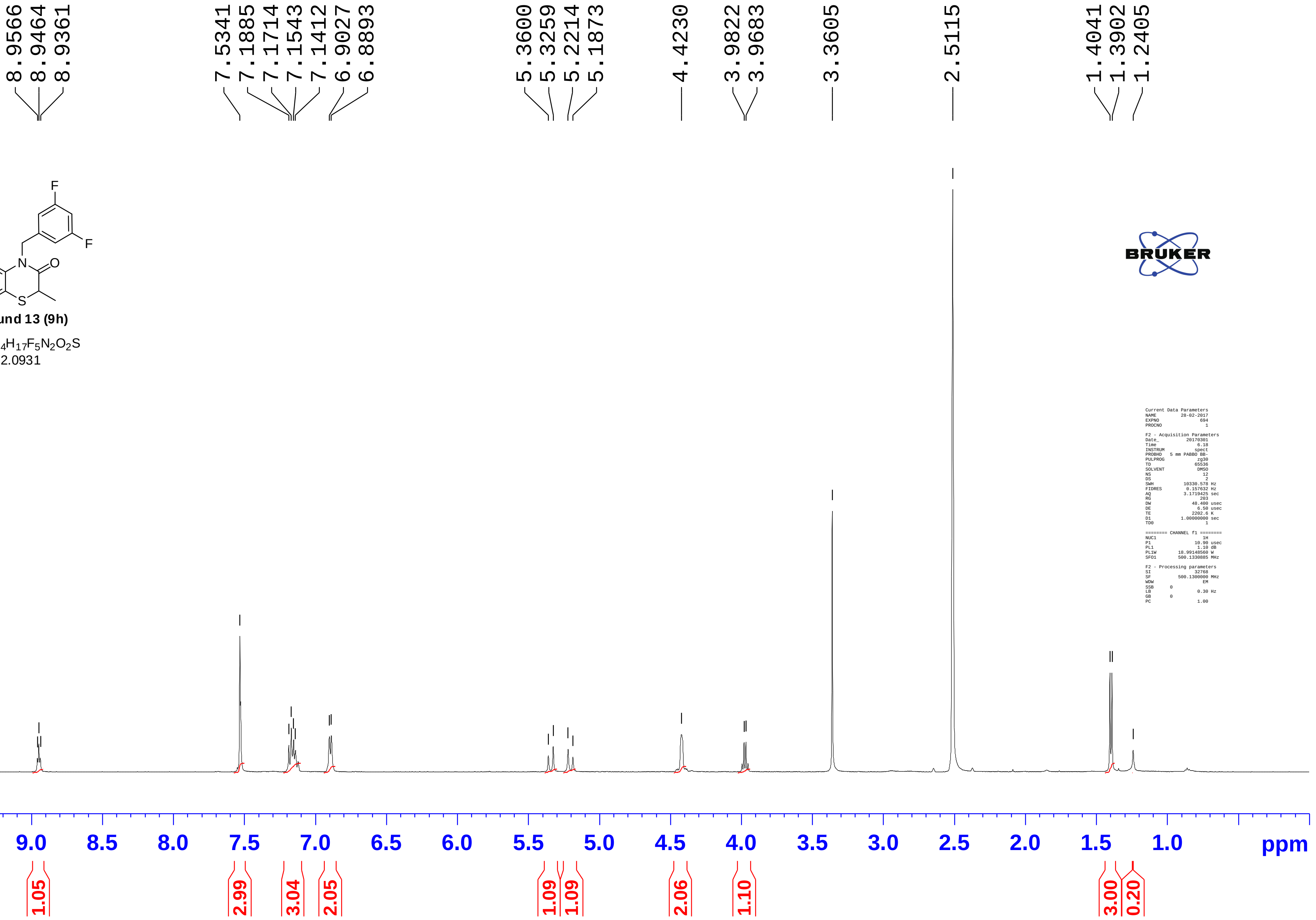

Qualitative Analysis Report

Compound 13 (9h)

**Data Filename**	AS-CRD-2973-B2.d	**Sample Name**	AS-CRD-2973-B2
**Sample Type**	Sample	**Position**	Vial 35
**Instrument Name**	Instrument 1	**User Name**	
**Acq Method**	Direct Mass-2017.m	**Acquired Time**	6/30/2020 9:31:59 PM
**IRM Calibration Status**	Some Ions Missed	**DA Method**	Default.m
**Comment**			

Sample Group **Info.**

Acquisition SW Version 6200 series TOF/6500 series Q-TOF B.05.00 (B5042.0)

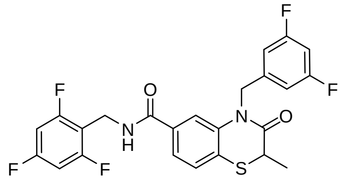

Compound 13 (9h)

Chemical Formula: $C_{24}H_{17}F_5N_2O_2S$

Exact Mass: 492.0931

User Chromatograms

Fragmentor Voltage 118 **Collision Energy** 0 **Ionization Mode** ESI

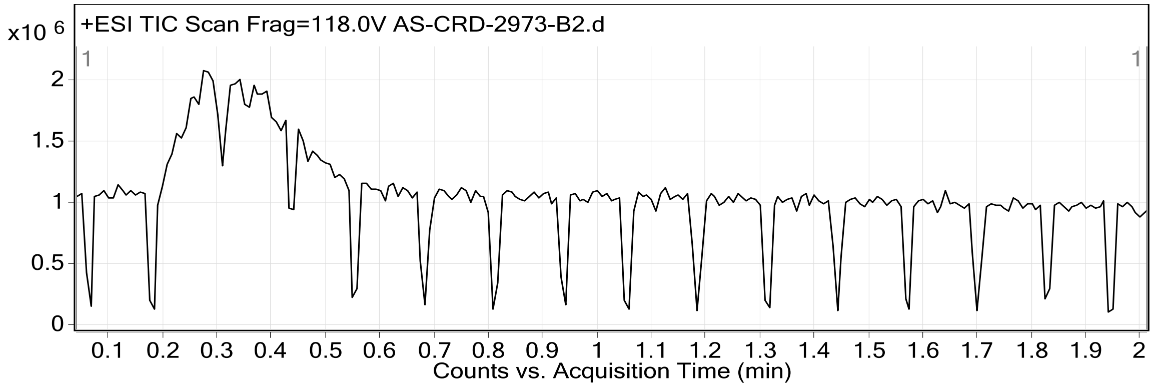

User Spectra

Fragmentor Voltage	Collision Energy	Ionization Mode
118	0	ESI

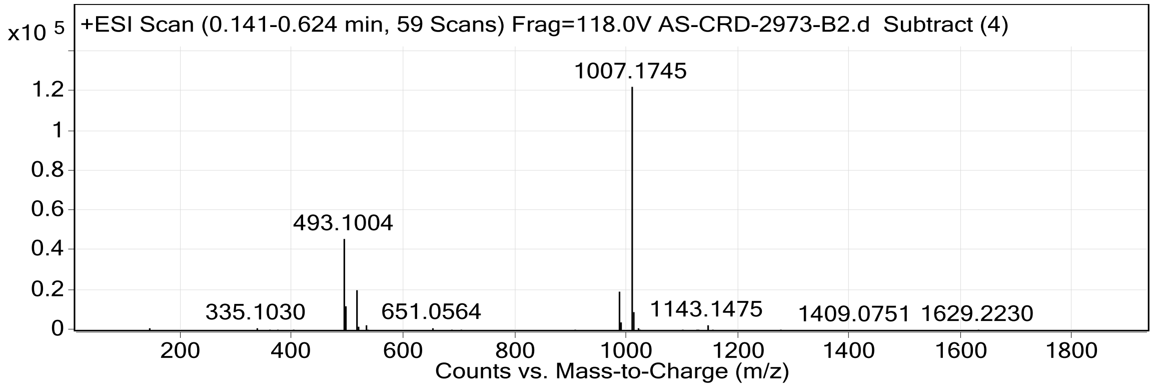

Peak List

m/z	z	Abund
493.1004	1	46126.13
494.1033	1	12187.93
515.0821	1	20805.63
516.0854	1	5918.15
985.1923	1	19718.57
986.1954	1	10839.6

1007.1745	1	122535.01
1008.1775	1	66105.42
1009.1772	1	28478.76
1010.1772	1	9209.25

Compound 13 (9h)

Compounds

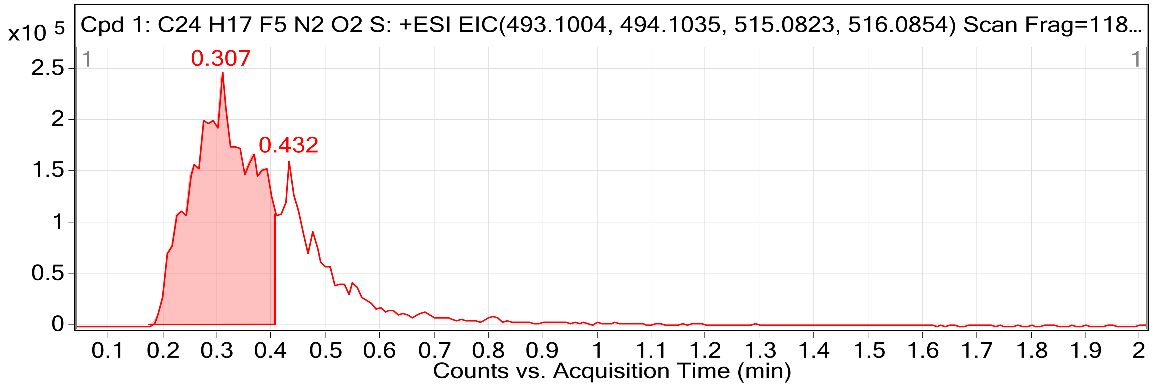

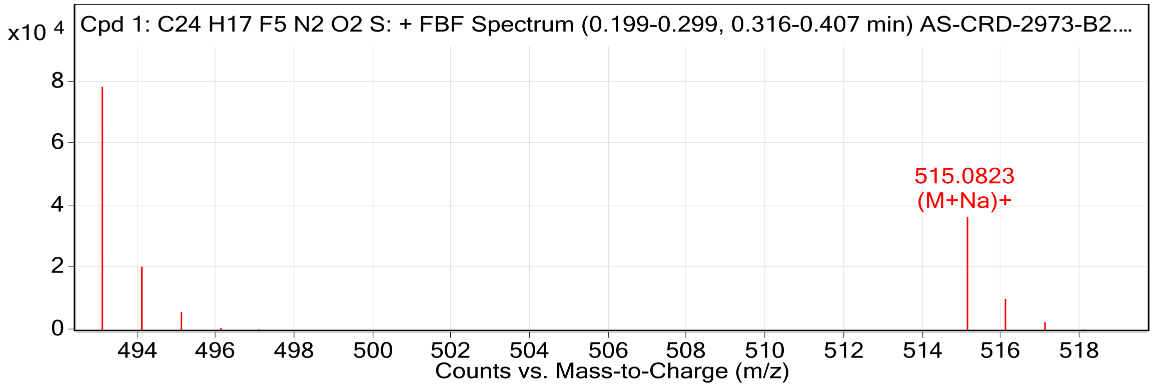

Peak List

m/z	z	Abund	Formula	Ion
493.1004	1	78599.25	C24H18F5N2O2S	(M+H)+
494.1033	1	20455.28	C24H18F5N2O2S	(M+H)+
495.1013	1	6108.03	C24H18F5N2O2S	(M+H)+
496.1019	1	1172.95	C24H18F5N2O2S	(M+H)+
497.0967	1	241.01	C24H18F5N2O2S	(M+H)+
515.0823	1	36768.59	C24H17F5N2NaO2S	(M+Na)+
516.0854	1	10221.49	C24H17F5N2NaO2S	(M+Na)+
517.0831	1	3000.48	C24H17F5N2NaO2S	(M+Na)+
518.0847	1	701.13	C24H17F5N2NaO2S	(M+Na)+
519.0791	1	79.88	C24H17F5N2NaO2S	(M+Na)+

MASS

Compound 13 (9h)

S A M P L E   I N F O R M A T I O N

Sample Name:	VK-CA204-99	Acquired By:	UPLC_MS_01 System
Vial:	2:F,5	Sample Set Name:	SAMPLE_FA
Injection #:	1	Acq. Method Set:	PH HEX_FA_6min
Injection Volume:	0.20 ul	Processing Method:	MASS
Run Time:	6.0 Minutes	Channel Name:	493.3Da
Date Acquired:	28-02-2017 14:24:04 IST	Date Processed:	28-02-2017 15:43:00 IST

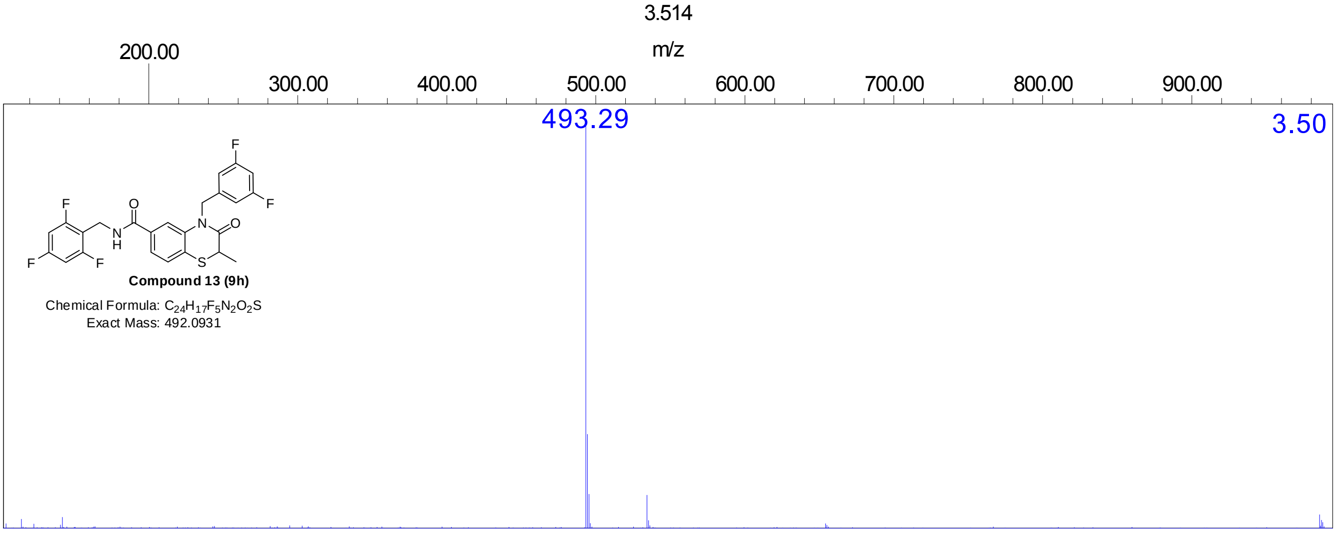

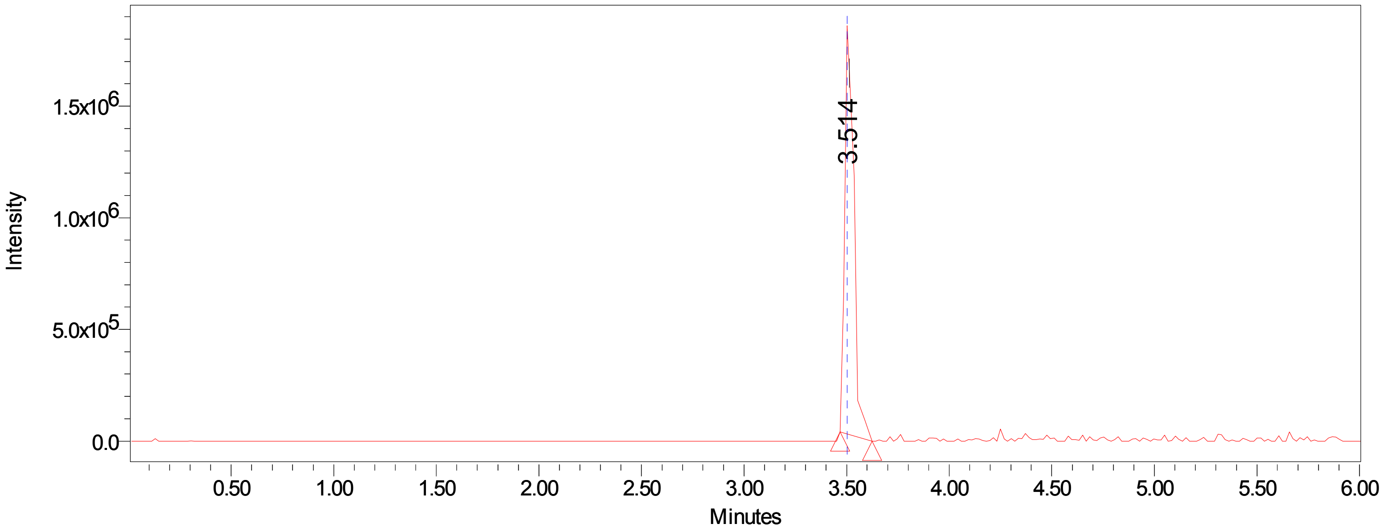

Channel Description 1: 100.00-1000.00 ES+, Centroid, CV=Tune; Processed Channel Descr. W3100 1: MS Scan MS 493.29 m/z Peak Separation: 1.0000 (1: 100.00-1000.00 ES+, Centroid, CV=Tune)

Compound 13 (9h)

SAMPLE INFORMATION

Sample Name:	VK-CA298-138	Acquired By:	UPLC_MS_01 System
Vial:	1:B,3	Sample Set Name:	AA
Injection #:	1	Acq. Method Set:	AA_C18_6min_N
Injection Volume:	0.80 ul	Processing Method:	UPLC1
Run Time:	6.0 Minutes	Channel Name:	220.0nm@3
Date Acquired:	12-06-2020 14:12:56 IST	Date Processed:	13-06-2020 16:24:19 IST
Column	Kinetex C18 (2.1x100mm)	Mobile Phase	1mM AA in Water/ACN

Auto-Scaled Chromatogram

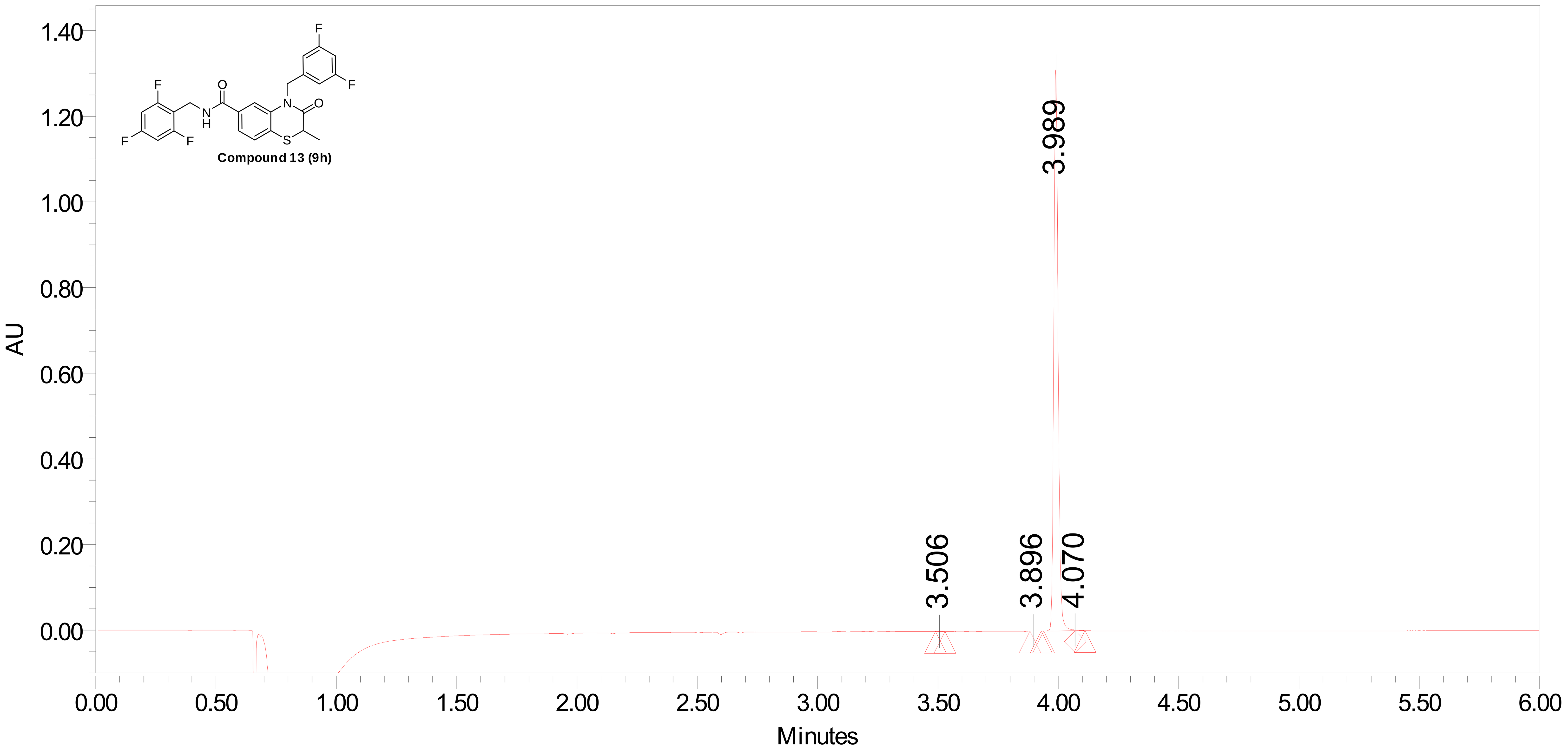

Processed Channel Descr. PDA 220.0 nm (PDA Spectrum (210-400)nm) Blank Subtracted from , Vial Inj. 144945840

Peak Results

	Name	RT	Area	% Area	Height
1		3.506	582	0.04	478
2		3.896	386	0.02	359
3		3.989	1640311	99.83	1310970
4		4.070	1796	0.11	1785

Compound 13 (9h)

SAMPLE INFORMATION

Sample Name:	VK-CA204-99	Acquired By:	UPLC_MS_01 System
Vial:	2:F,5	Sample Set Name:	SAMPLE_FA
Injection #:	1	Acq. Method Set:	PH HEX_FA_6min
Injection Volume:	0.20 ul	Processing Method:	UPLC
Run Time:	6.0 Minutes	Channel Name:	220.0nm@1
Date Acquired:	28-02-2017 14:24:04 IST	Date Processed:	28-02-2017 15:42:13 IST
Column	PHENYL HEXYL (2.1x100mm)	Mobile Phase	0.1% FA in Water/ACN

Auto-Scaled Chromatogram

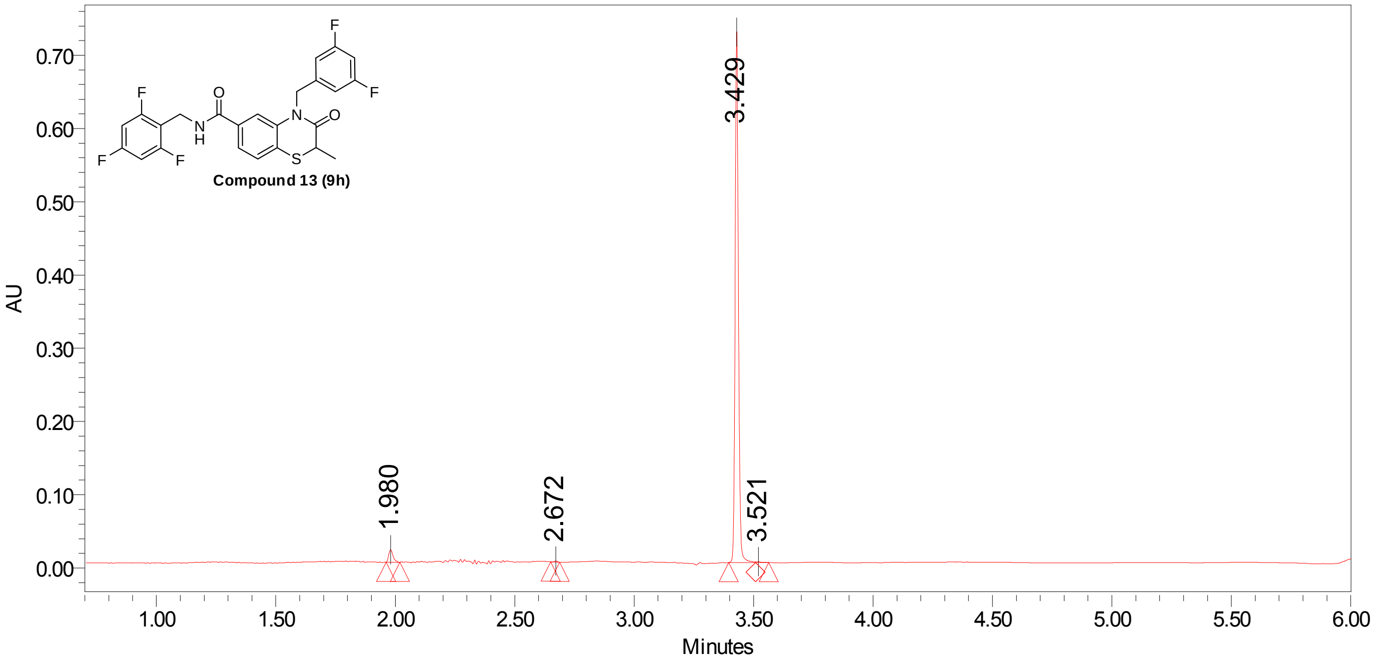

Processed Channel Descr. PDA 220.0 nm (PDA Spectrum (210-400)nm) Blank Subtracted from BLANK_ACN, Vial 1:F,8 Inj. 1

Peak Results

	Name	RT	Area	% Area	Height
1		1.980	21965	3.02	17068
2		2.672	1298	0.18	1387
3		3.429	702148	96.54	724828
4		3.521	1936	0.27	1220

SA-CA285-30

Compound 14 (9m)

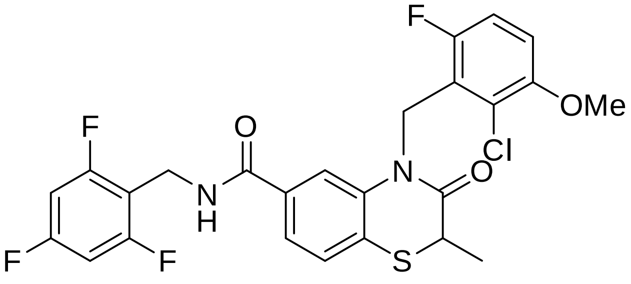

Chemical Formula: $C_{25}H_{19}ClF_4N_2O_3S$

Exact Mass: 538.07

Molecular Weight: 538.94

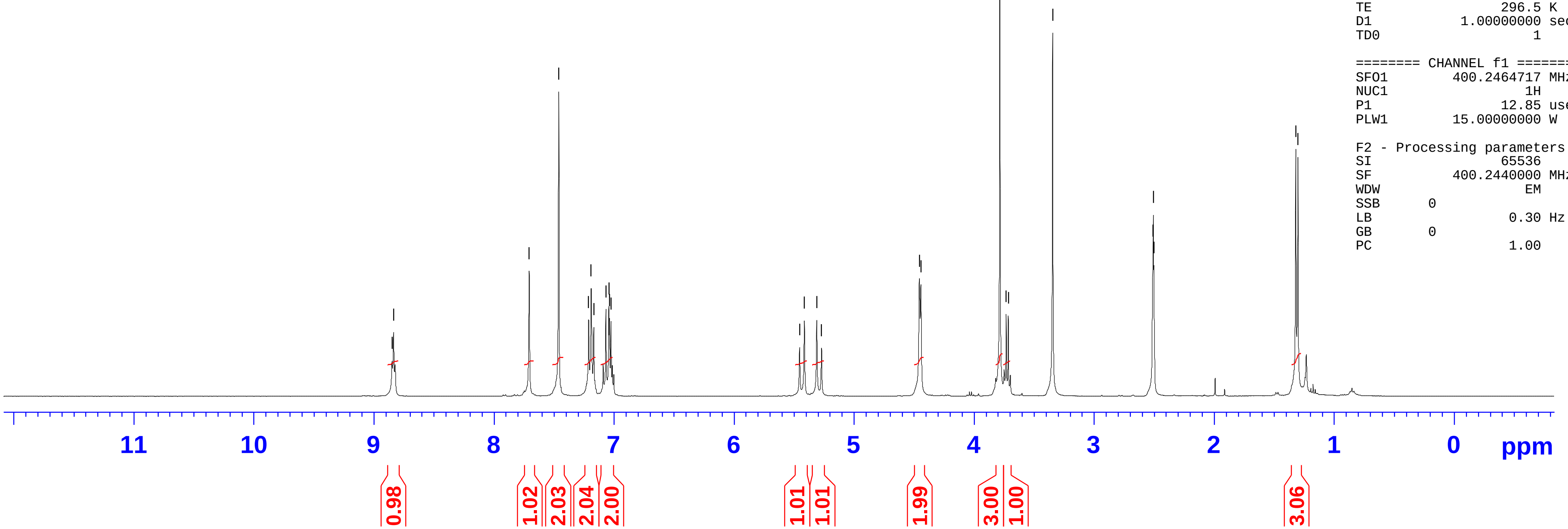


```
Current Data Parameters
NAME          10-06-2019
EXPNO                110
PROCNO                 1

F2 - Acquisition Parameters
Date_           20190610
Time               15.20
INSTRUM            spect
PROBHD   5 mm PABBO BB/
PULPROG             zg30
TD                 65536
SOLVENT             DMSO
NS                    16
DS                     2
SWH             8012.820 Hz
FIDRES          0.122266 Hz
AQ             4.0894465 sec
RG                144.37
DW                62.400 usec
DE                  6.50 usec
TE                 296.5 K
D1            1.00000000 sec
TD0                    1

======== CHANNEL f1 ========
SFO1         400.2464717 MHz
NUC1                  1H
P1                 12.85 usec
PLW1         15.00000000 W

F2 - Processing parameters
SI                 65536
SF           400.2440000 MHz
WDW                   EM
SSB      0
LB                  0.30 Hz
GB       0
PC                  1.00
```

Qualitative Analysis Report

Compound 14 (9m)

**Data Filename**	SA-CA-285-30.d	**Sample Name**	SA-CA-285-30
**Sample Type**	Sample	**Position**	Vial 70
**Instrument Name**	Instrument 1	**User Name**	
**Acq Method**	Direct Mass-2017.m	**Acquired Time**	6/16/2020 12:38:21 PM
**IRM Calibration Status**	Some Ions Missed	**DA Method**	Default.m
**Comment**			

Sample Group **Info.**

Acquisition SW Version 6200 series TOF/6500 series Q-TOF B.05.00 (B5042.0)

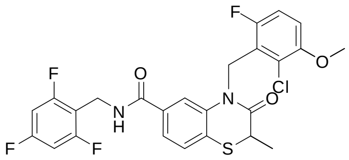

Chemical Formula: $C_{25}H_{19}ClF_4N_2O_3S$
Exact Mass: 538.0741

User Chromatograms

Fragmentor Voltage 118 **Collision Energy** 0 **Ionization Mode** ESI

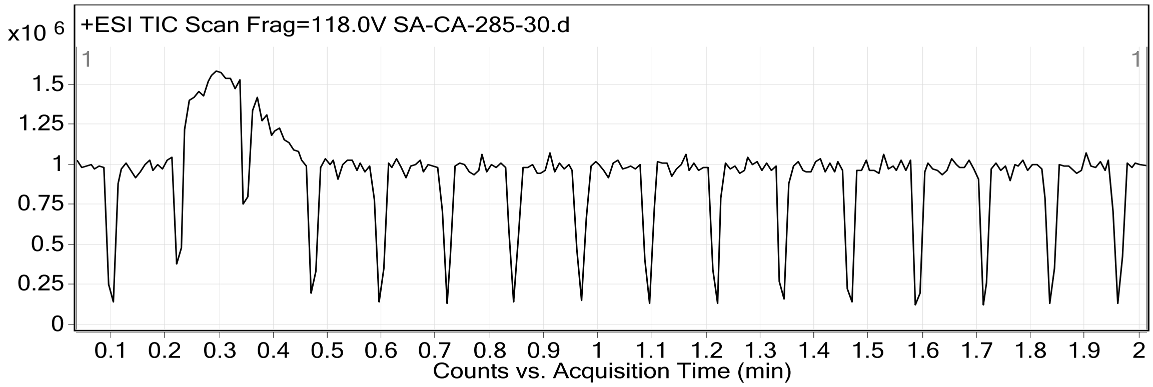

User Spectra

**Fragmentor Voltage**	**Collision Energy**	**Ionization Mode**
118	0	ESI

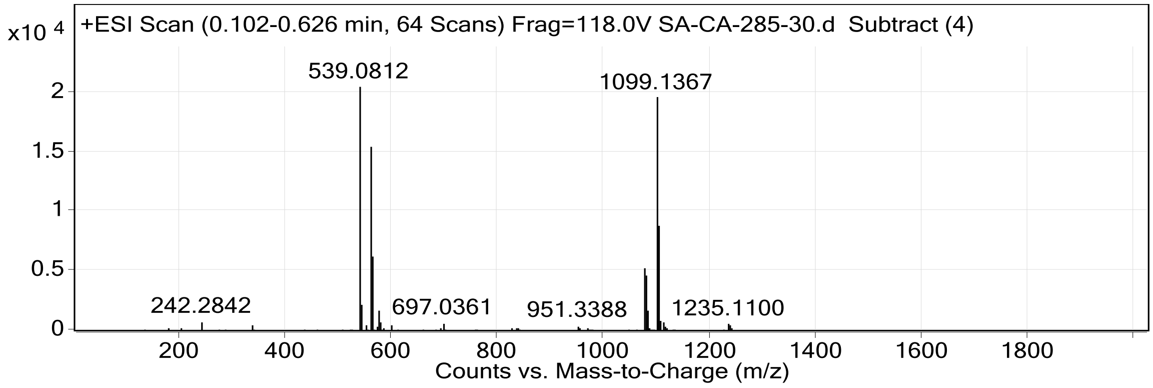

Peak List

m/z	z	Abund
539.0812	1	20484.19
540.0842	1	5833.02
541.0789	1	8048.69
561.063	1	15397.42
563.0607	1	6306.87
1099.1367	1	19566.97

1100.1397	1	11265.94
1101.1356	1	17613.41
1102.1374	1	8876.65
1103.1345	1	5655.08

Compound 14 (9m)

Compounds

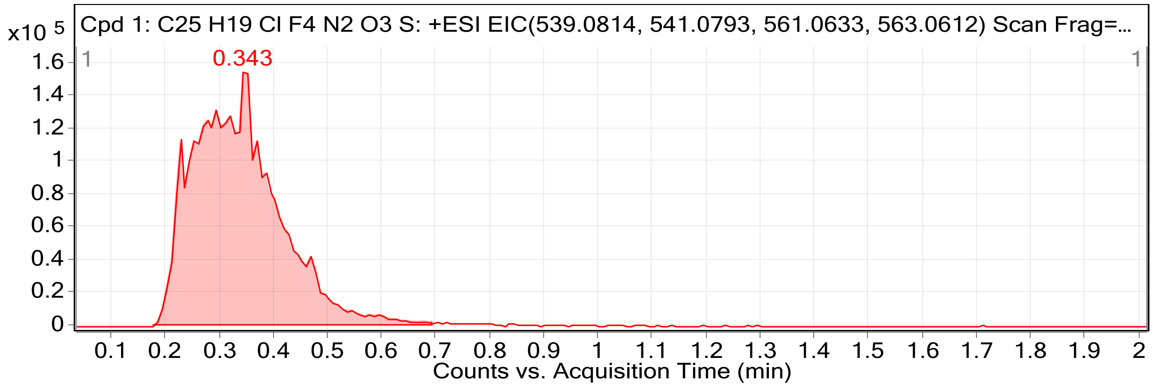

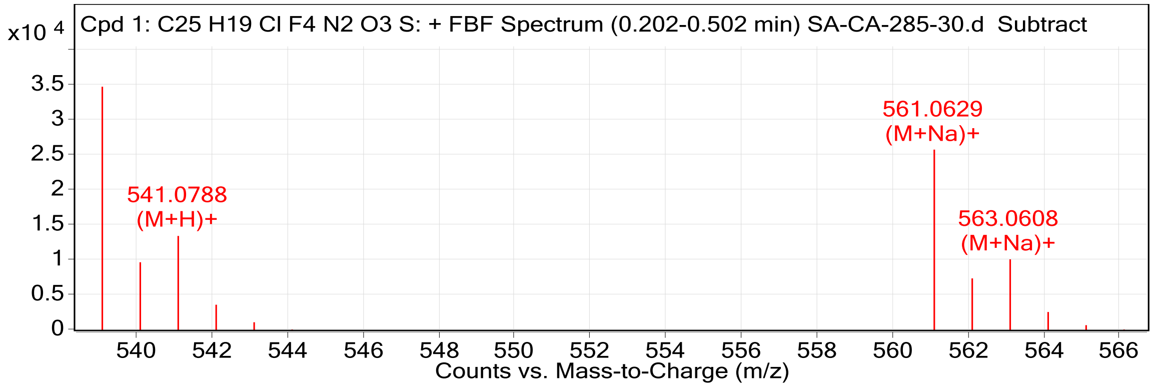

Peak List

m/z	z	Abund	Formula	Ion
539.0811	1	34743.85	C25H20ClF4N2O3S	(M+H)+
540.0842	1	9837.06	C25H20ClF4N2O3S	(M+H)+
541.0788	1	13576.8	C25H20ClF4N2O3S	(M+H)+
542.0814	1	3706.13	C25H20ClF4N2O3S	(M+H)+
543.0783	1	1154.98	C25H20ClF4N2O3S	(M+H)+
561.0629	1	25793.13	C25H19ClF4N2NaO3S	(M+Na)+
562.066	1	7377.54	C25H19ClF4N2NaO3S	(M+Na)+
563.0608	1	10185.91	C25H19ClF4N2NaO3S	(M+Na)+
564.0635	1	2796.5	C25H19ClF4N2NaO3S	(M+Na)+
565.0614	1	884.24	C25H19ClF4N2NaO3S	(M+Na)+

Multi MASS Report

Compound 14 (9m)

SAMPLE INFORMATION

Sample Name:	NP-CA264-74-A2	Acquired By:	System
Sample Type:	Unknown	Sample Set Name:	AA
Vial:	1:E,6	Acq. Method Set:	AA_C18_6min_N
Injection #:	1	Processing Method:	MASS
Injection Volume:	0.50 ul	Channel Name:	539.1Da@2, 539.1Da, 365.1Da@1
Run Time:	6.0 Minutes	Date Acquired:	28-04-2018 14:10:14 IST

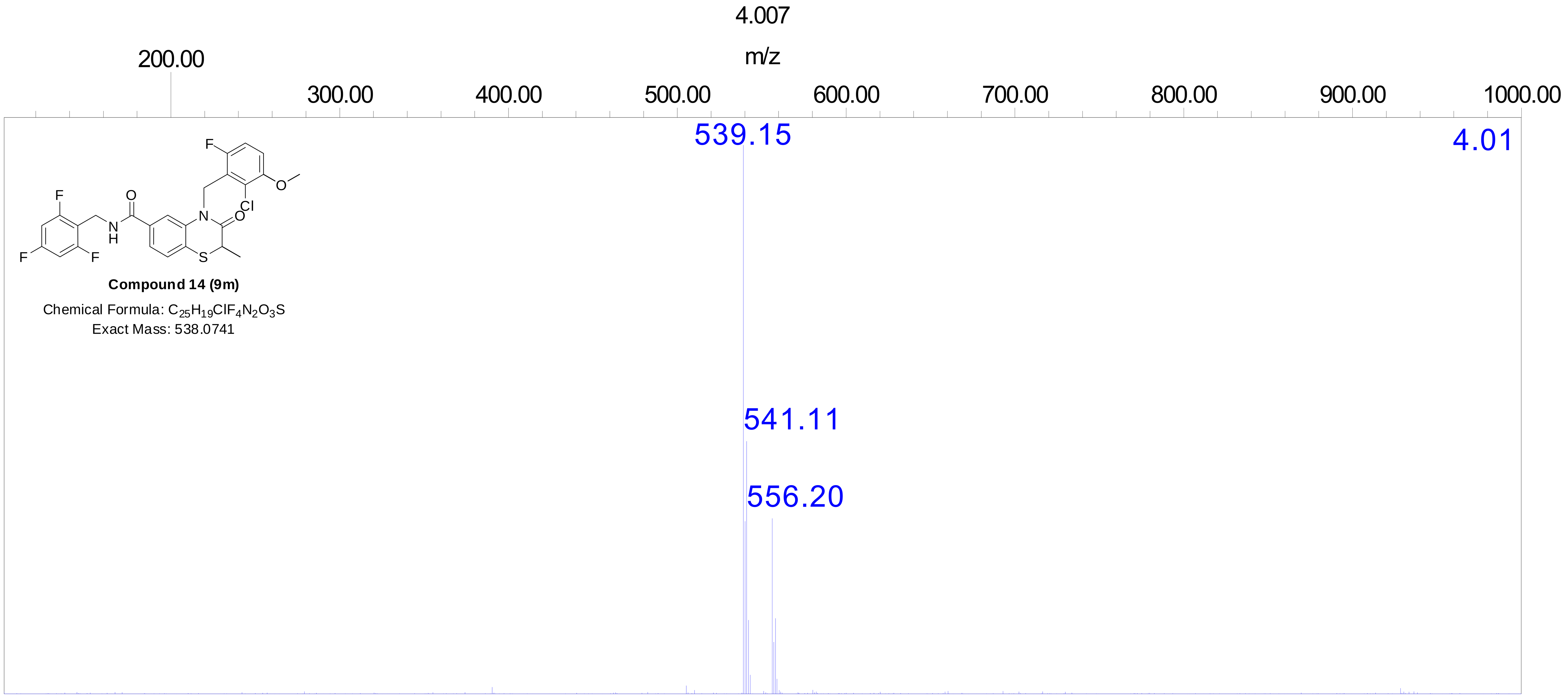

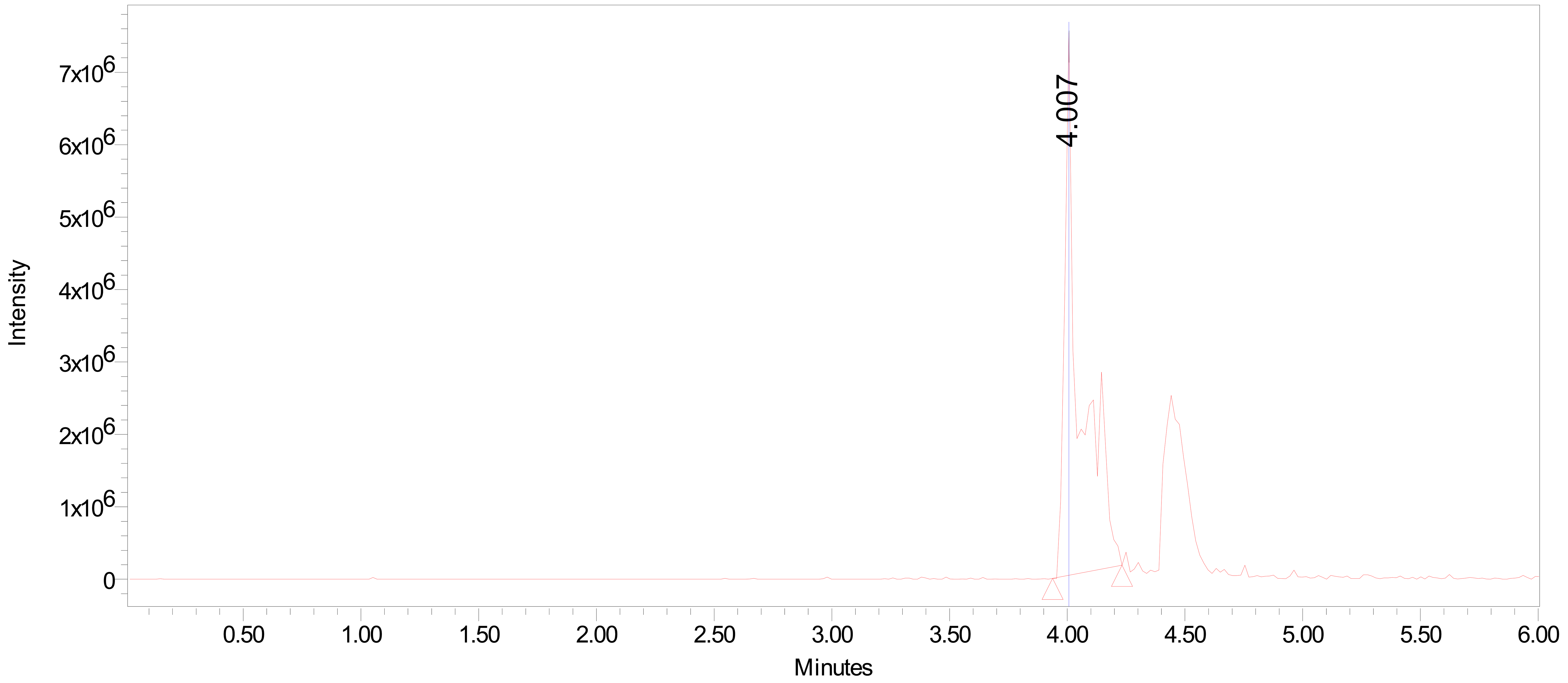

Processed Channel Descr. W3100 1: MS Scan MS 539.15 m/z Peak Separation: 1.0000 (1: 100.00-1000.00 ES+, Centroid, CV=Tune)

Compound 14 (9m)

SAMPLE INFORMATION

Sample Name:	SA-CA285-30	Acquired By:	UPLC_MS_01 System
Vial:	1:E,4	Sample Set Name:	SAMPLE_FA
Injection #:	1	Acq. Method Set:	FA_C18_6min_N
Injection Volume:	3.00 ul	Processing Method:	UPLC1
Run Time:	6.0 Minutes	Channel Name:	220.0nm
Date Acquired:	11-06-2020 17:19:00 IST	Date Processed:	11-06-2020 17:36:24 IST
Column	KINETEX_EVO_C-18	Mobile Phase	0.1% FA in Water/ACN

Auto-Scaled Chromatogram

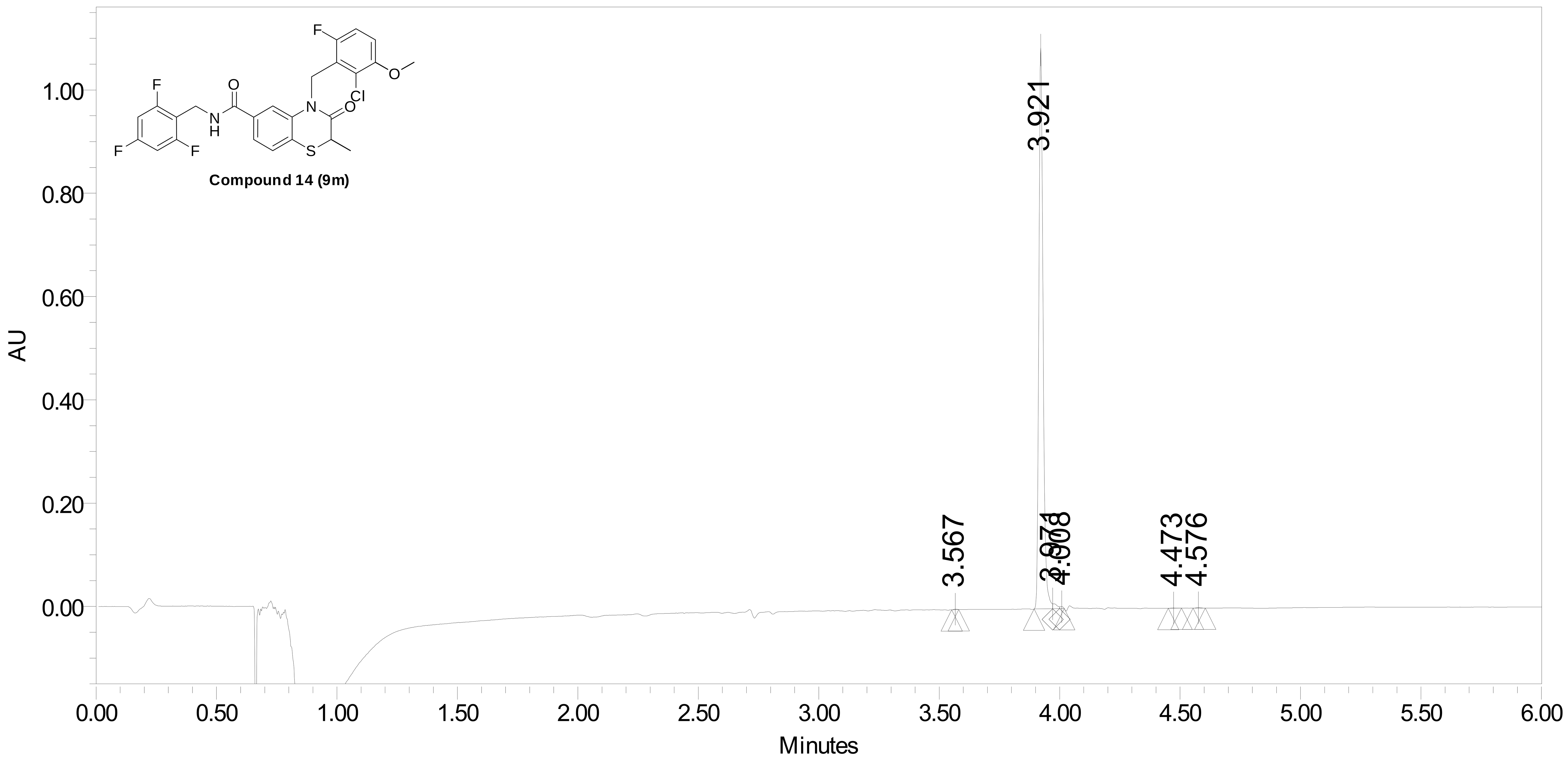

Processed Channel Descr. PDA 220.0 nm (PDA Spectrum (210-400)nm) Blank Subtracted from BLANK_DMSO, Vial 1:F,7 Inj. 1

Peak Results

	Name	RT	Area	% Area	Height
1		3.567	1063	0.08	1194
2		3.921	1330534	98.60	1086845
3		3.971	12009	0.89	9456
4		4.008	3746	0.28	3865
5		4.473	694	0.05	524
6		4.576	1353	0.10	1041

Compound 14 (9m)

SAMPLE INFORMATION

Sample Name:	NP-CA264-74-A2	Acquired By:	UPLC_MS_01 System
Vial:	1:E,6	Sample Set Name:	AA
Injection #:	1	Acq. Method Set:	AA_C18_6min_N
Injection Volume:	0.50 ul	Processing Method:	UPLC
Run Time:	6.0 Minutes	Channel Name:	250.0nm@1
Date Acquired:	28-04-2018 14:10:14 IST	Date Processed:	16-03-2019 11:42:25 IST
Column	Kinetex C18 (2.1x100mm)	Mobile Phase	5mM AA in Water/ACN

Auto-Scaled Chromatogram

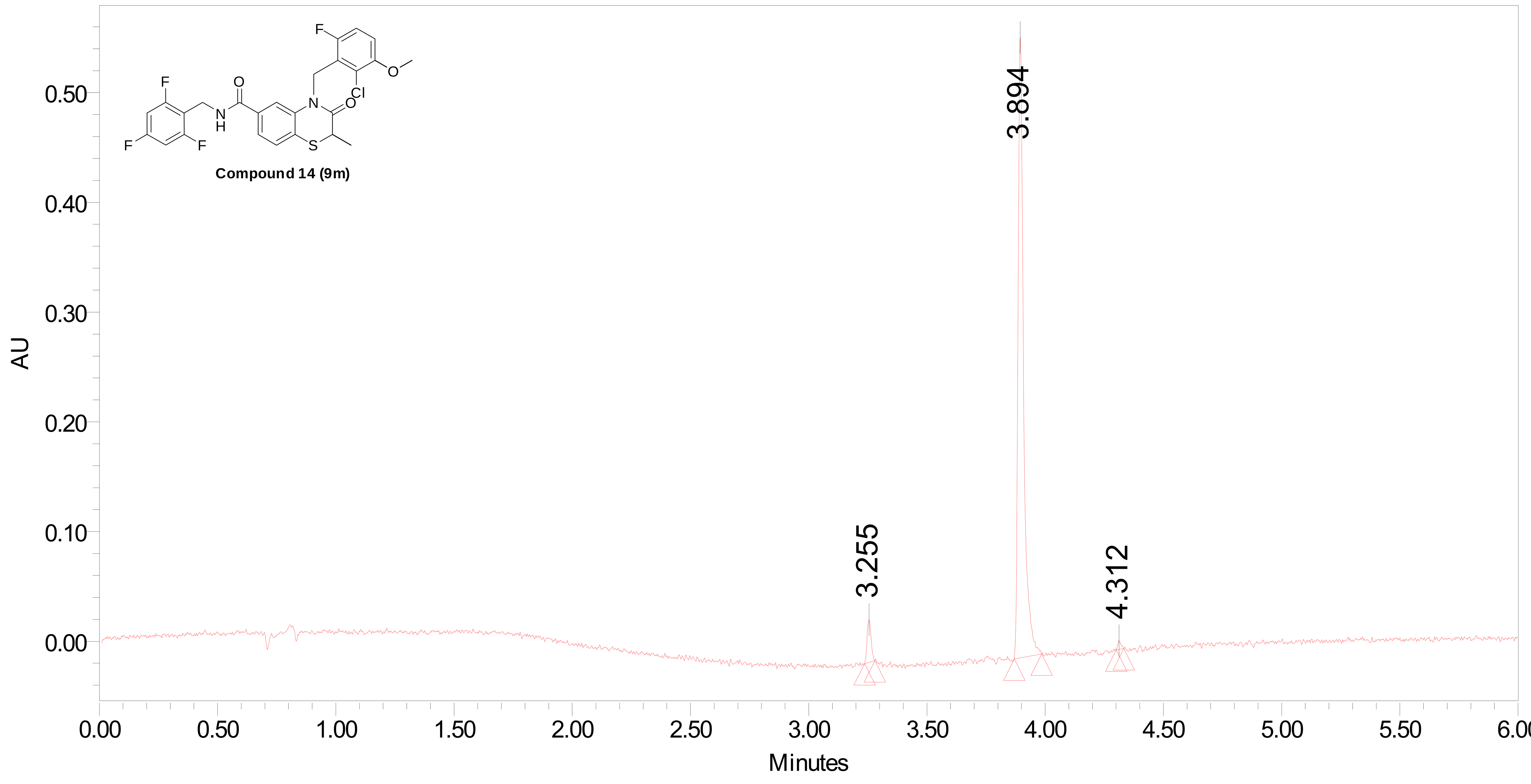

Peak Results

	Name	RT	Area	% Area	Height
1		3.255	46061	4.71	38931
2		3.894	923758	94.52	565973
3		4.312	7458	0.76	8117
