# Supplementary material for: G10 is a direct activator of human STING
Source: PLoS One. 2020 Sep 10;15(9):e0237743. doi: 10.1371/journal.pone.0237743 (PMC7482845; doi:10.1371/journal.pone.0237743)
Supplement: S1 Raw images — (PDF) [file pone.0237743.s015.pdf]

FIGURE 2

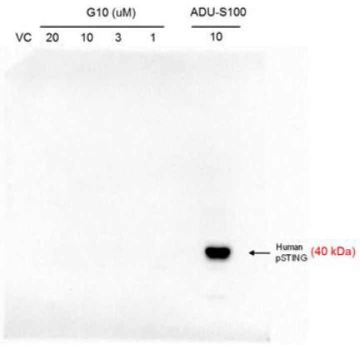

Used for pSTING in the main figure 2

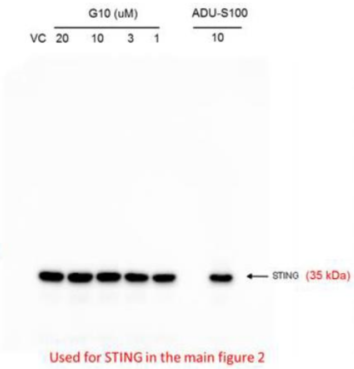

Used for STING in the main figure 2

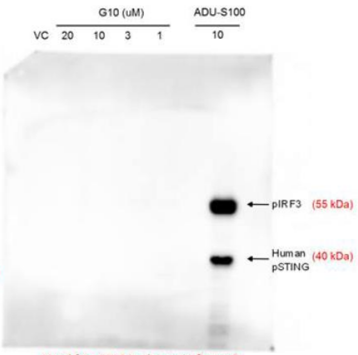

Used for pIRF3 in the main figure 2

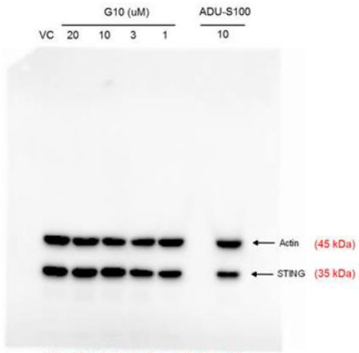

Used for Actin in the main figure 2

FIGURE 4

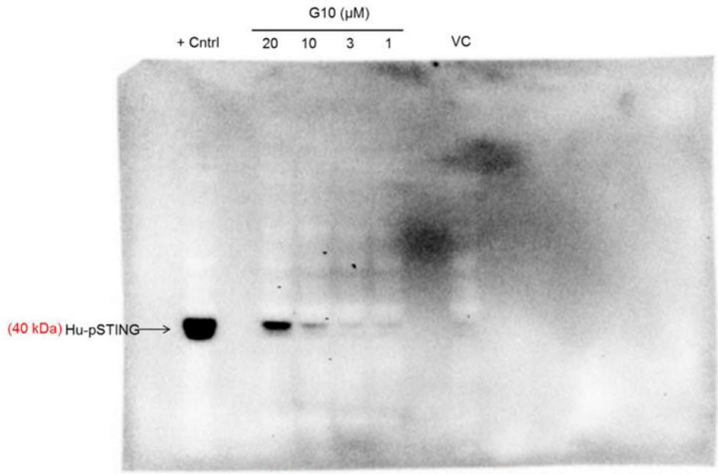

Used for human pSTING in the main figure 4

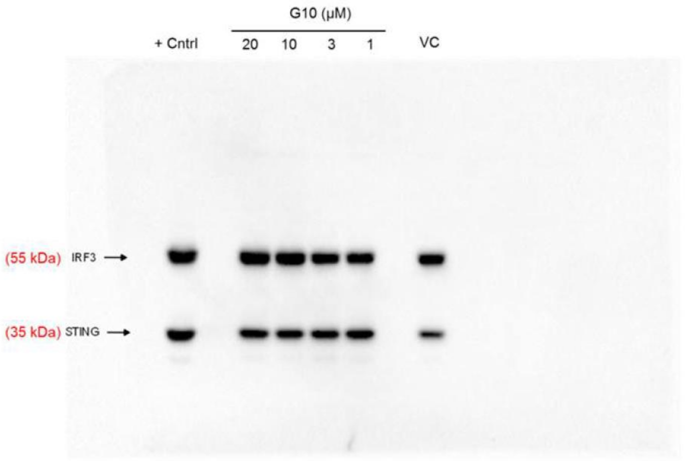

Used for human STING in the main figure 4

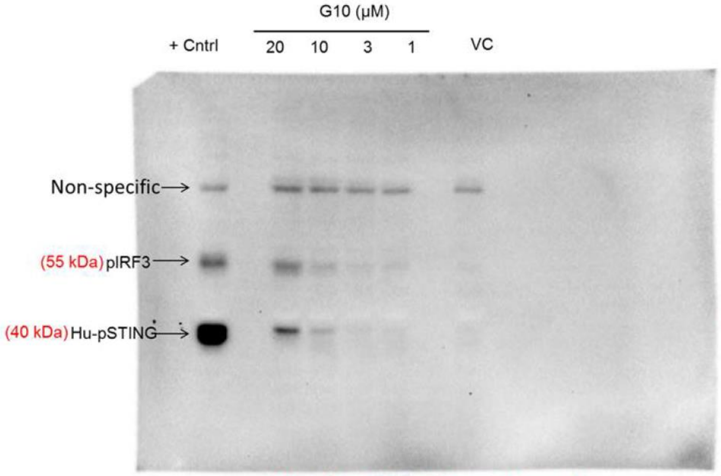

Used for pIRF3 in the main figure 4

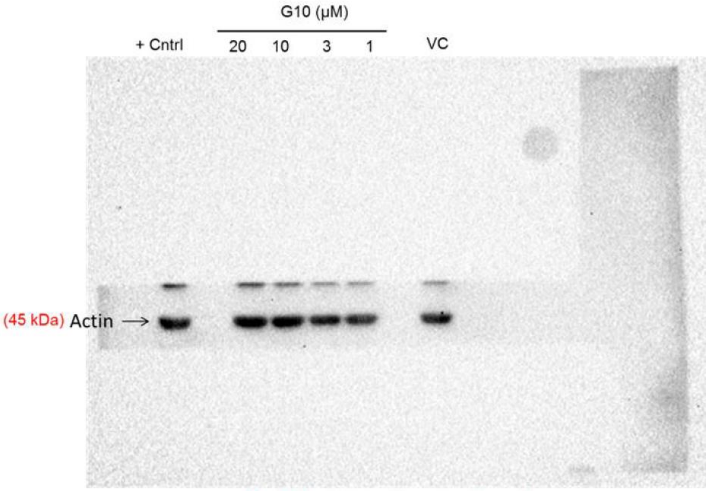

Used for Actin in the main figure 4

FIGURE 5

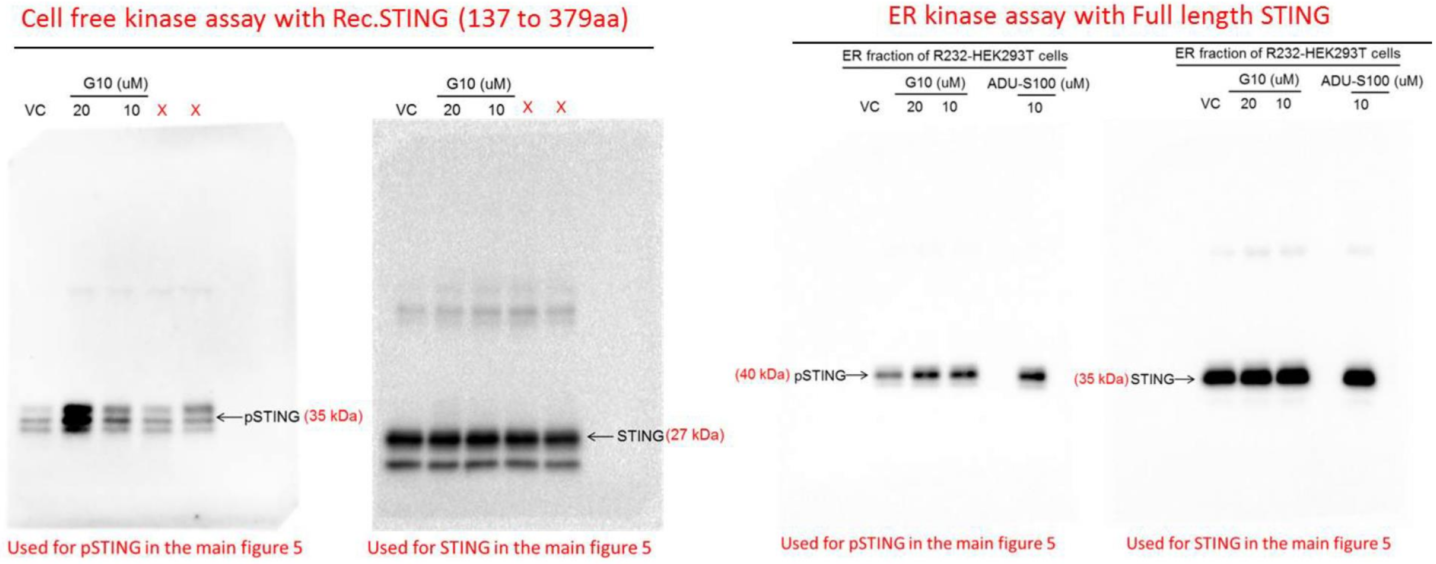

FIGURE 7

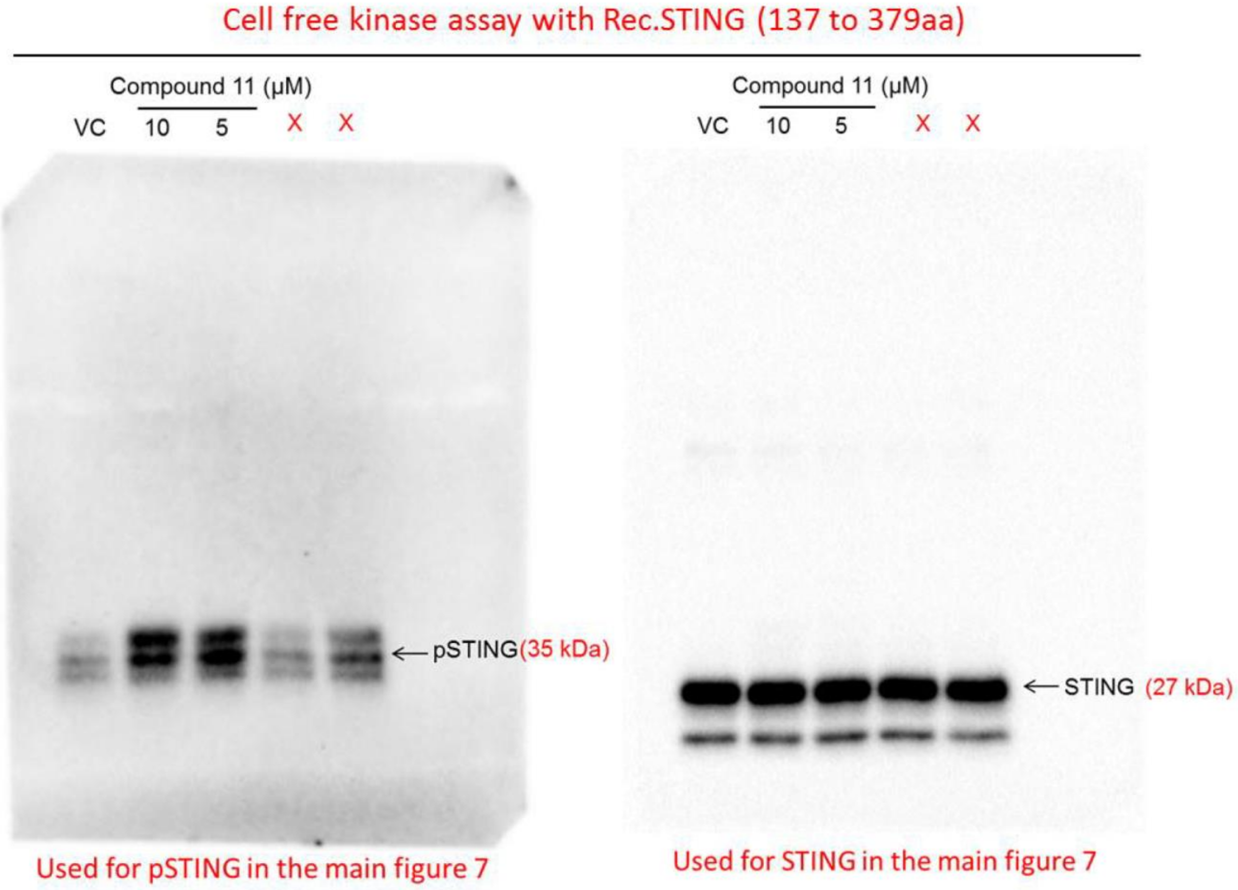

FIGURE 8

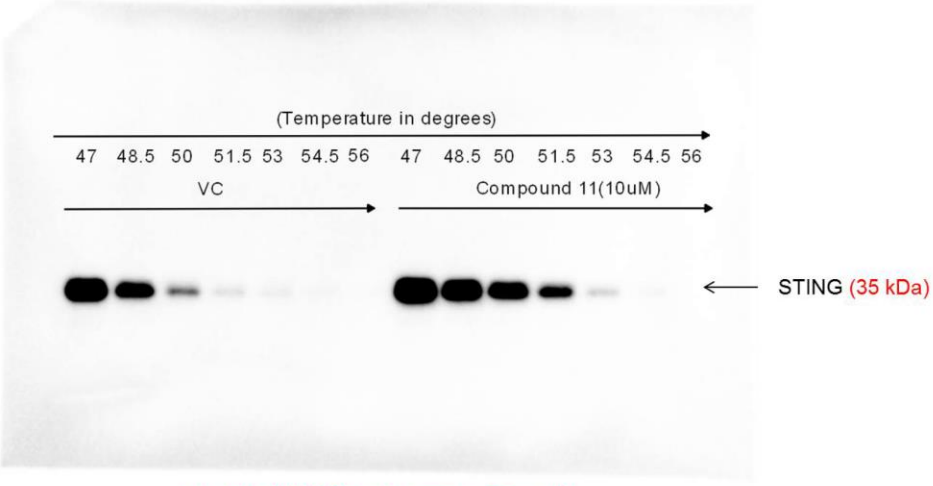

Used for STING in the main figure 8

FIGURE 9

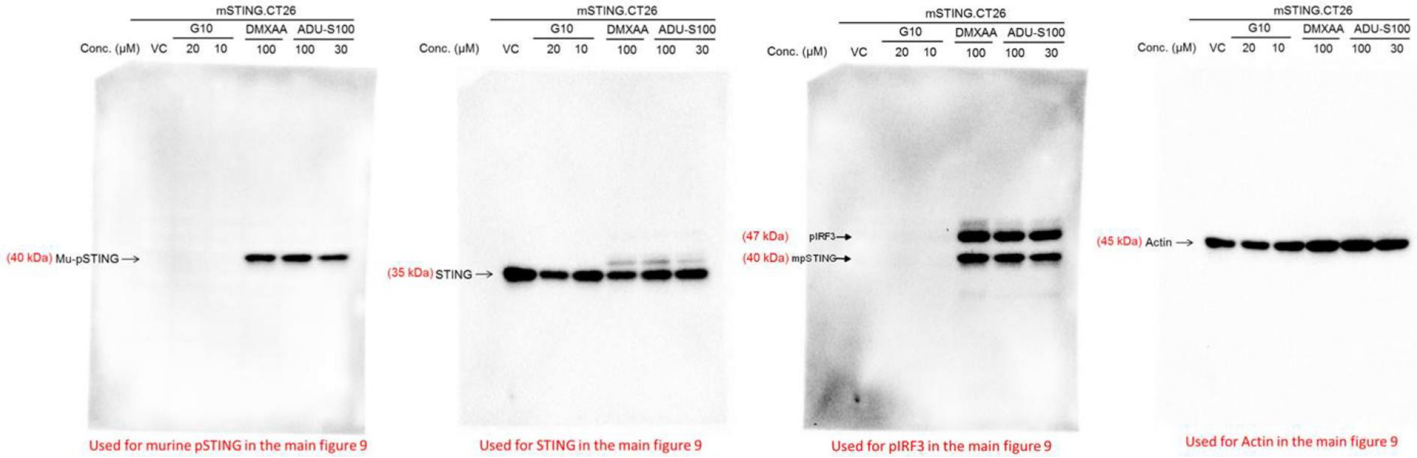

Used for murine pSTING in the main figure 9

Used for STING in the main figure 9

Used for pIRF3 in the main figure 9

Used for Actin in the main figure 9

FIGURE 10

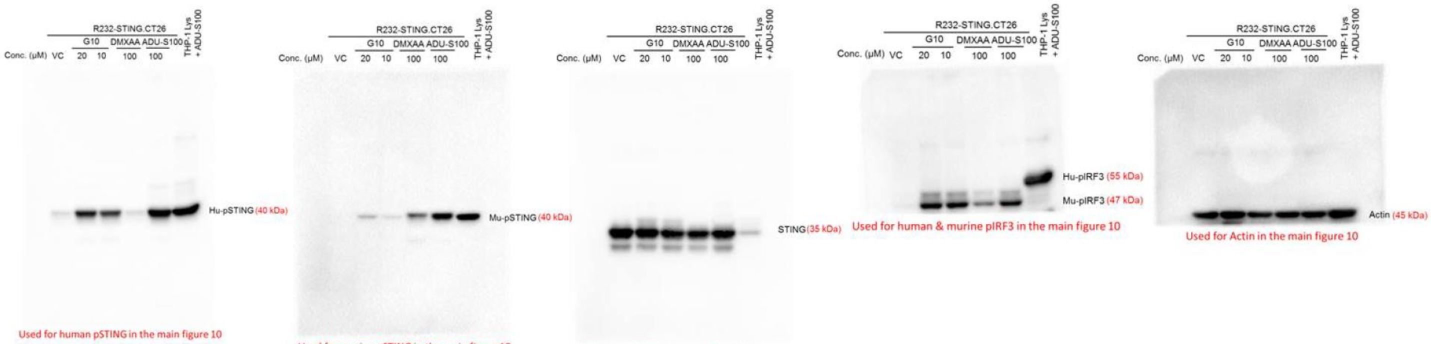

Used for human & murine pIRF3 in the main figure 10

Used for Actin in the main figure 10

Used for human pSTING in the main figure 10

Used for murine pSTING in the main figure 10

Used for STING in the main figure 10

SUPPLEMENTARY FIGURE S2

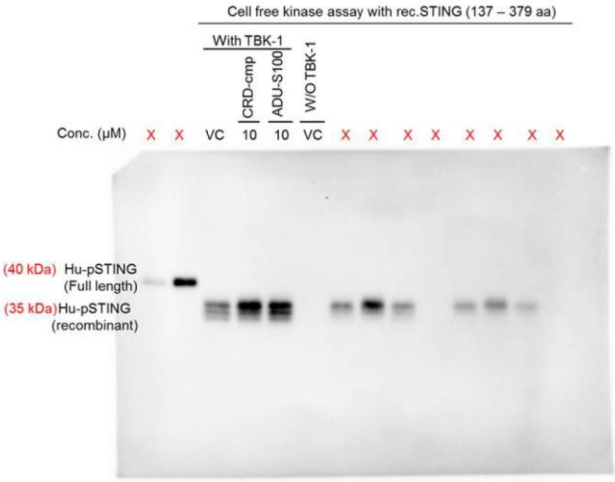

Used for human pSTING in the main figure S2

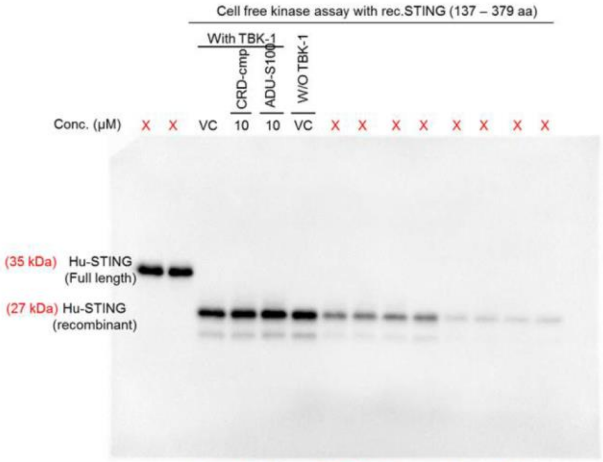

Used for human STING in the main figure S2

SUPPLEMENTARY FIGURE S3

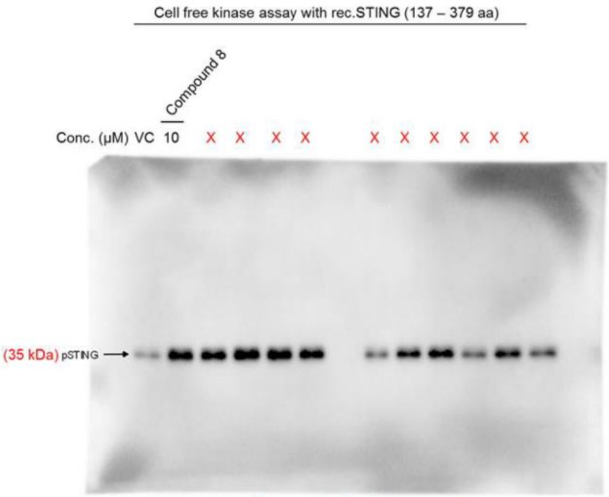

Used for human pSTING in the main figure S3

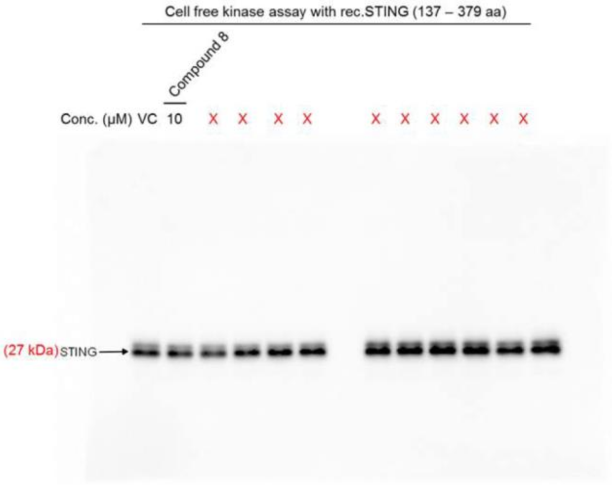

Used for human STING in the main figure S3

SUPPLEMENTARY FIGURE S5

R232.293T

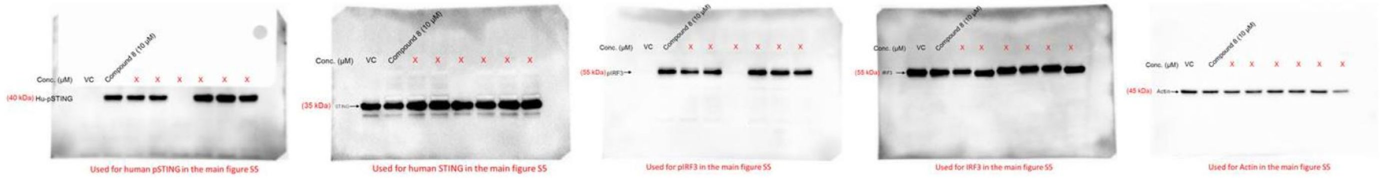

H232.293T

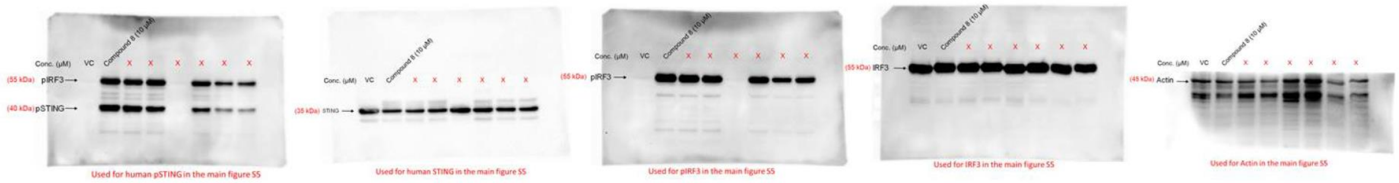

HAQ.293T

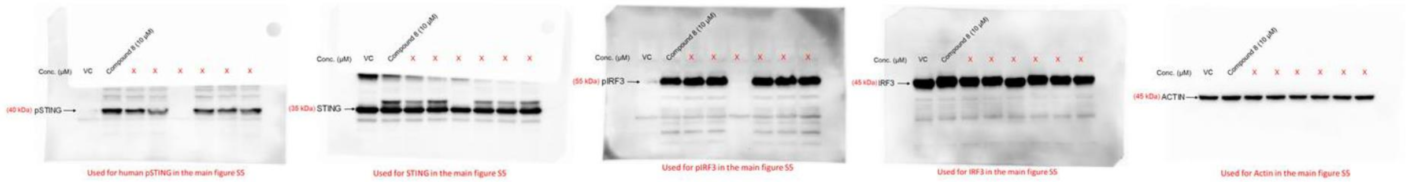

AQ.293T

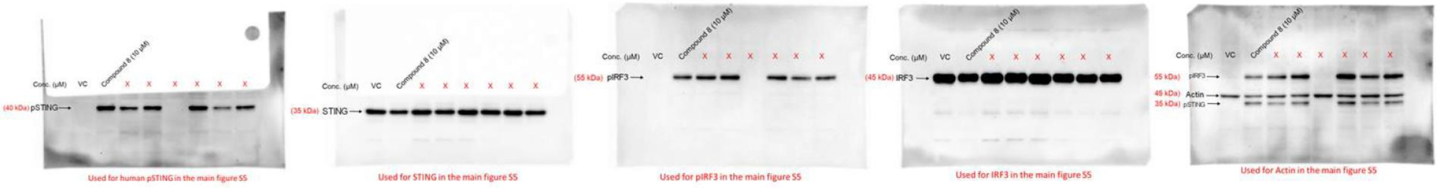

SUPPLEMENTARY FIGURE S6

D1:R232/R232

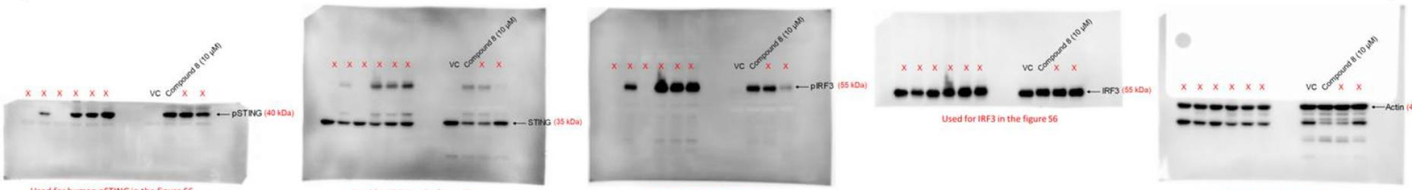

Used for human pSTING in the figure S6 | Used for STING in the figure S6 | Used for pIRF3 in the figure S6 | Used for IRF3 in the figure S6 | Used for Actin in the figure S6

D2:R232/H232

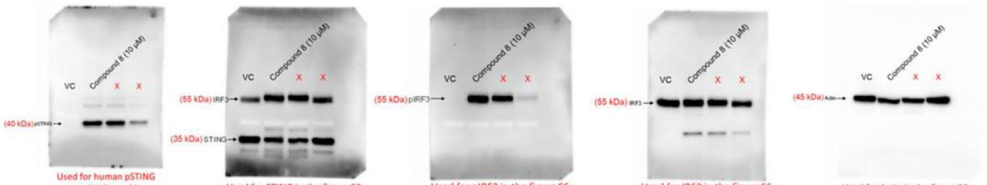

Used for human pSTING in the figure S6 | Used for STING in the figure S6 | Used for pIRF3 in the figure S6 | Used for IRF3 in the figure S6 | Used for Actin in the figure S6

D3:R232/HAQ

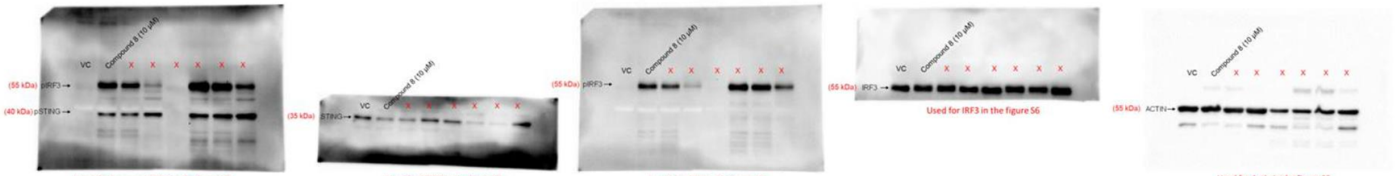

Used for human pSTING in the figure S6 | Used for STING in the figure S6 | Used for pIRF3 in the figure S6 | Used for IRF3 in the figure S6 | Used for Actin in the figure S6

SUPPLEMENTARY FIGURE S7

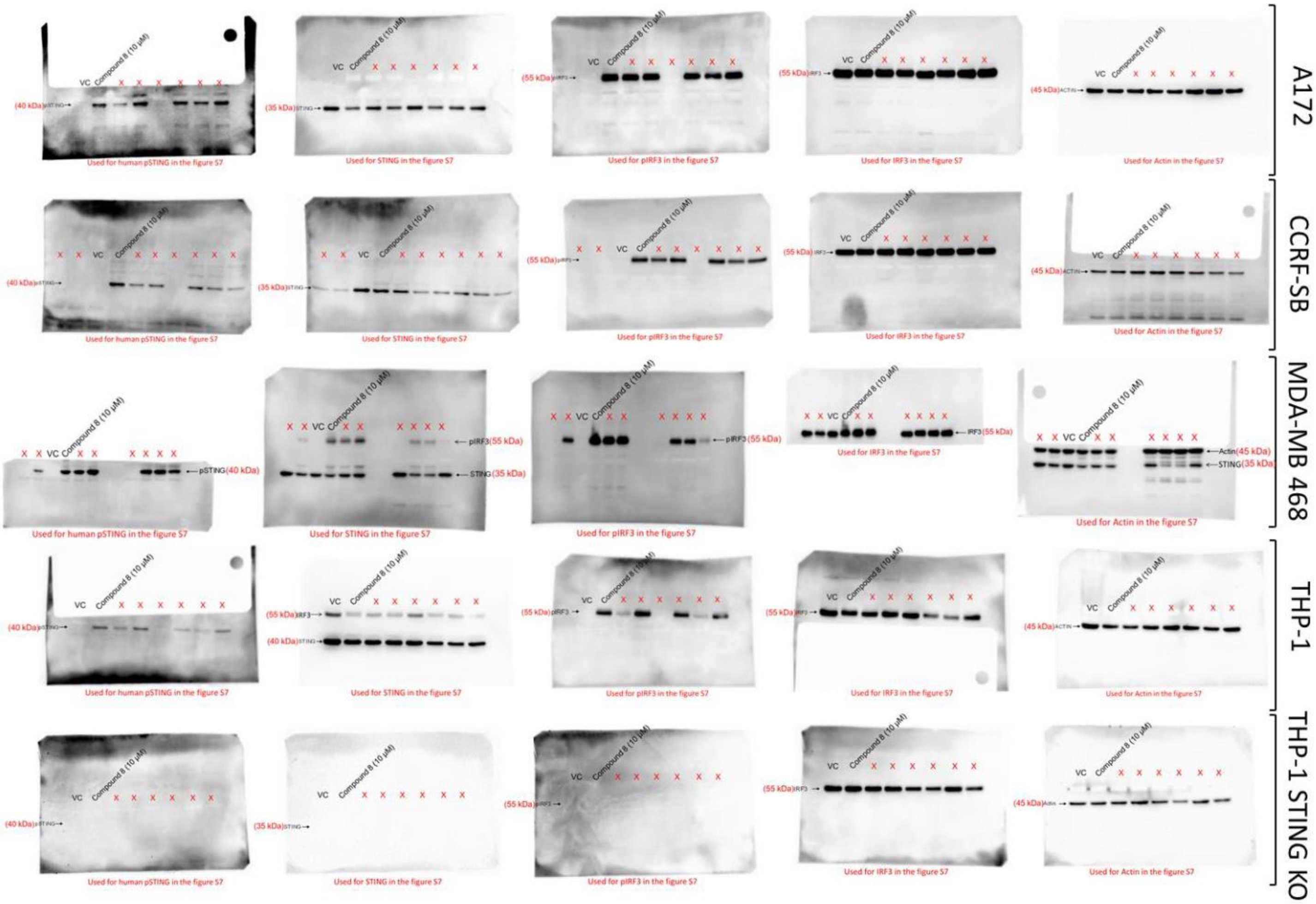

SUPPLEMENTARY FIGURE S8

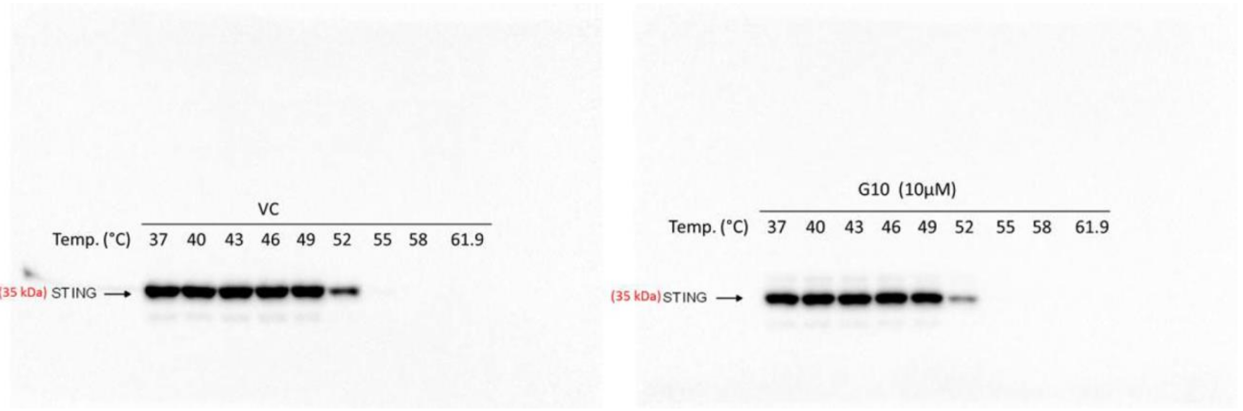

SUPPLEMENTARY FIGURE S9

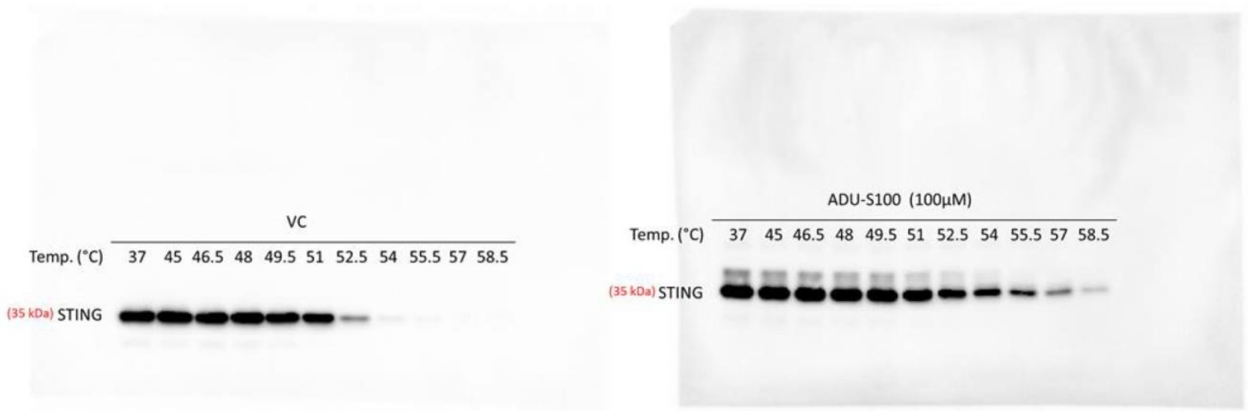

SUPPLEMENTARY FIGURE S10

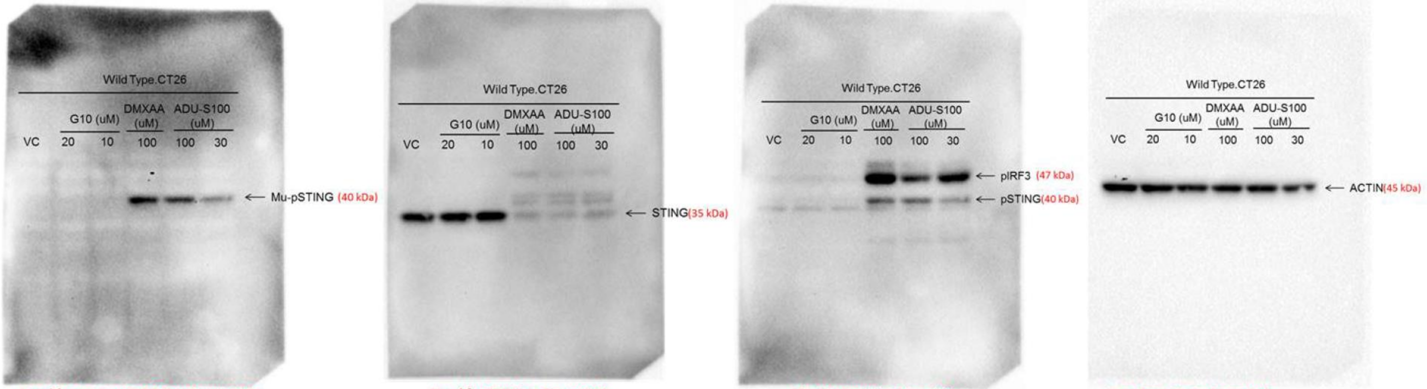

Used for murine pSTING in Figure S10 Used for STING in Figure S10 Used for pIRF3 in Figure S10 Used for Actin in Figure S10
